# Supplementary material for: Deep learning-based behavioral profiling of rodent stroke recovery
Source: BMC Biol. 2022 Oct 15;20:232. doi: 10.1186/s12915-022-01434-9 (PMC9571460; doi:10.1186/s12915-022-01434-9)
Supplement: Supplementary file 1 — Additional file 1: Fig. S1. Walking profile of mice following DeepLabCut tracking. Fig. S2. Tracking of body parts in injured mice and mice with different genotypes. Fig. S3. Kinematic changes in spontaneous walk after stroke. Fig. S4. Angular variability between body center and front and hind paws. Fig. S5. Subgroup analysis for random forest classification and principal component analysis. Fig. S6. Principal component analysis and random forest classification of uninjured control mice. Fig. S7. Correlation plots between human annotators and DeepLabCut evaluation of the ladder rung test. Fig. S8. Overview of overhead for establishing DLC-assisted analysis. Fig. S9. Functional assessment of recovery after stroke using conventional behavioral tests. Fig. S10. Manual labeling of joints and body parts for DLC. [file 12915_2022_1434_MOESM1_ESM.pdf]

Supplementary material

Deep learning based behavioral profiling of rodent stroke recovery

Rebecca Z Weber[1,2], Geertje Mulders[3], Julia Kaiser[4], Christian Tackenberg[1,2,#,*], Ruslan Rust[1,2,#,*]

[1] Institute for Regenerative Medicine, University of Zurich, 8952 Schlieren, Switzerland,

[2] Neuroscience Center Zurich, University of Zurich and ETH Zurich, Zurich, Switzerland

[3] Department of Health Sciences and Technology, ETH Zurich, Zurich, Switzerland

[4] Burke Neurological Institute, White Plains, New York, USA

[#] equal contribution, * correspondence authors

Correspondence to:

Ruslan Rust

Institute for Regenerative Medicine (IREM)

University of Zurich, Campus Schlieren

Wagistrasse 12

8952 Schlieren / Zurich, Switzerland

ruslan.rust@irem.uzh.ch, +41 44 63 53215

Christian Tackenberg

Institute for Regenerative Medicine (IREM)

University of Zurich, Campus Schlieren

Wagistrasse 12

8952 Schlieren / Zurich, Switzerland

christian.tackenberg@irem.uzh.ch, +41 44 6340929

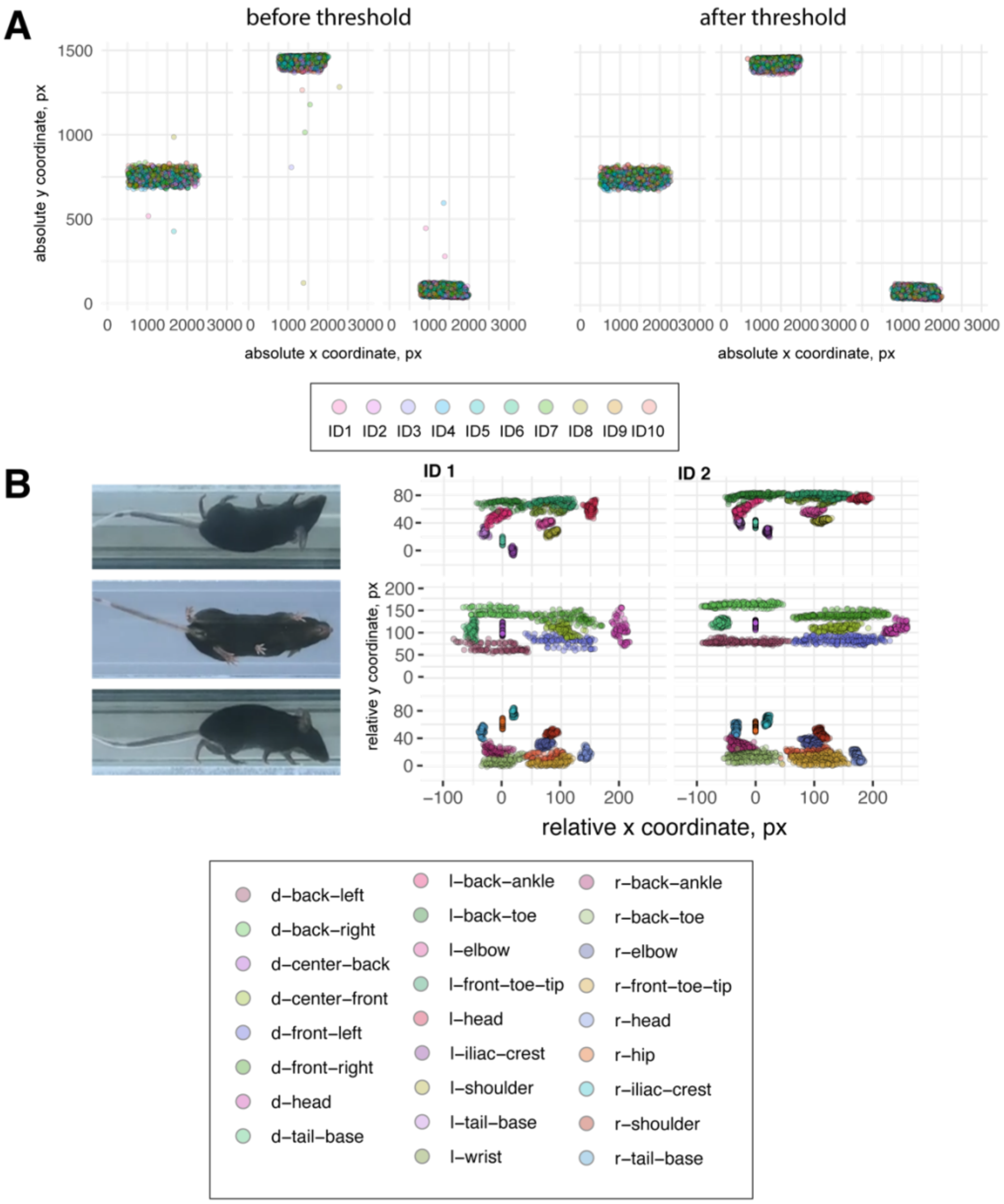

Suppl. Fig. 1: Walking profile of mice following DeepLabCut tracking. (**A**) Randomly sampled raw data plot of original x and y coordinates before pre-processing and after pre-processing. (**B**) Walking profile normalized to the hip coordinates of randomly selected individual non-injured mice (ID 1, ID2). Each dot represents an anatomical landmark.

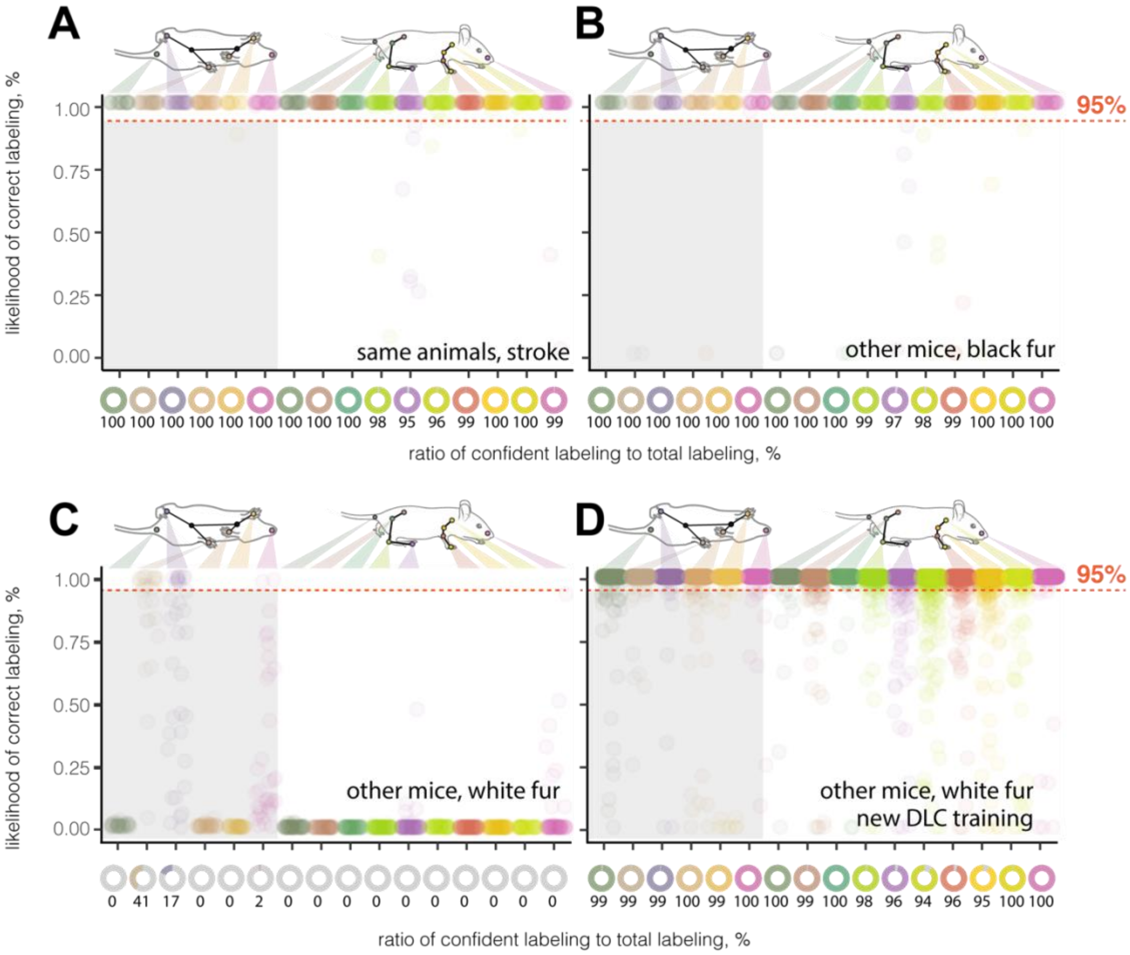

Suppl. Fig. 2: Tracking of body parts in injured mice and mice with different genotypes. (A) Likelihood of a confident labeling for individual body parts in the runway in stroked mice **(B)** stains with a different genotype but same (black) fur color and **(C)** different genotype but white fur color **(D)** Likelihood of a confident labeling for new training set in mice with different genotype and white fur. Each dot represents an anatomical landmark of individual image frames in a video. The red dotted line represents the confidence threshold of 95% likelihood for reliable labeling.

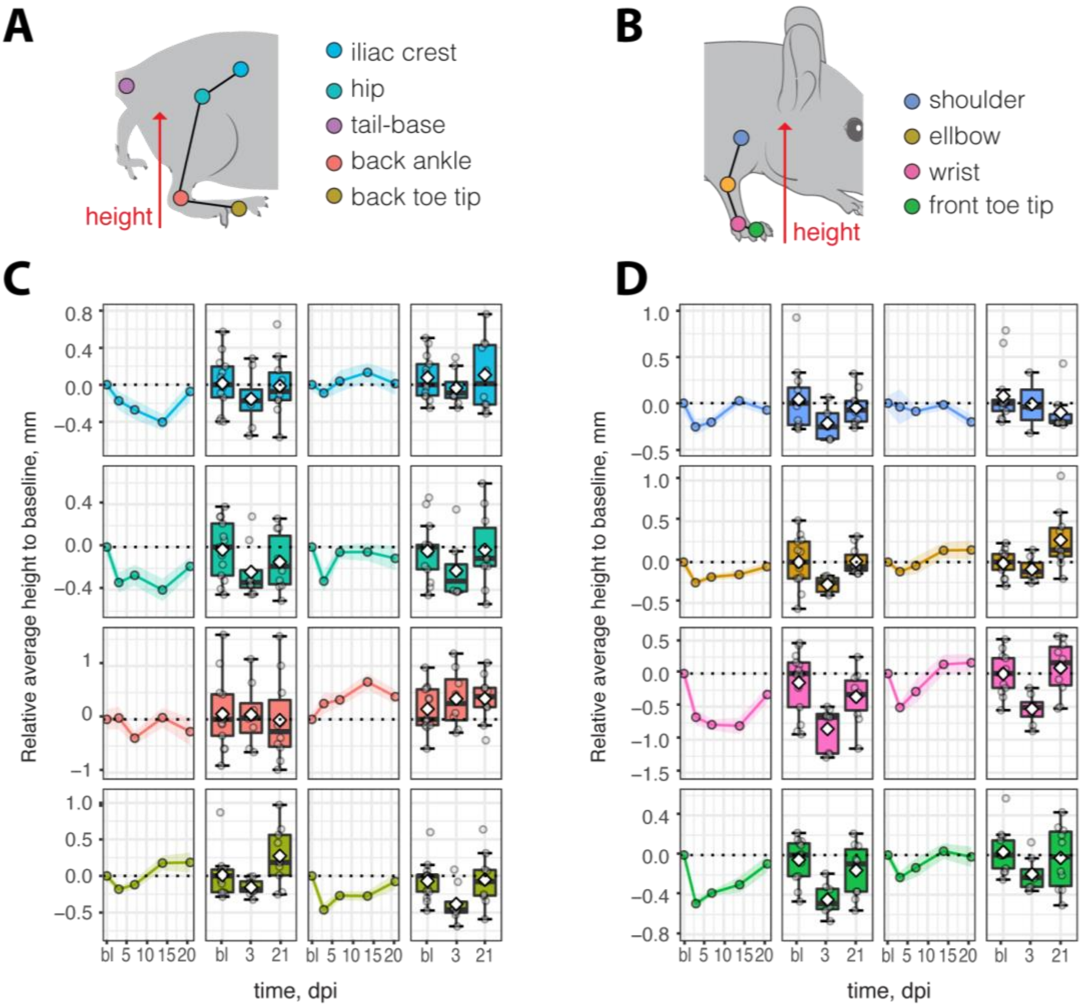

Suppl. Fig 3: Kinematic changes in spontaneous walk after stroke. (A, B) Schematic overview of tracked parameters (C, D) Average height of selected joints at 3,7, 14, 21 dpi compared to baseline. Data are shown as mean distributions where the white dot represents the mean. Boxplots indicate the 25% to 75% quartiles of the data. For boxplots: each dot in the plots represents one animal. Line graphs are plotted as mean ± sem. For line graphs: the dots represent the mean of the data. Significance of mean differences between the groups was assessed using Tukey's HSD. Asterisks indicate significance: * P < 0.05, ** P < 0.01, *** P < 0.001

A

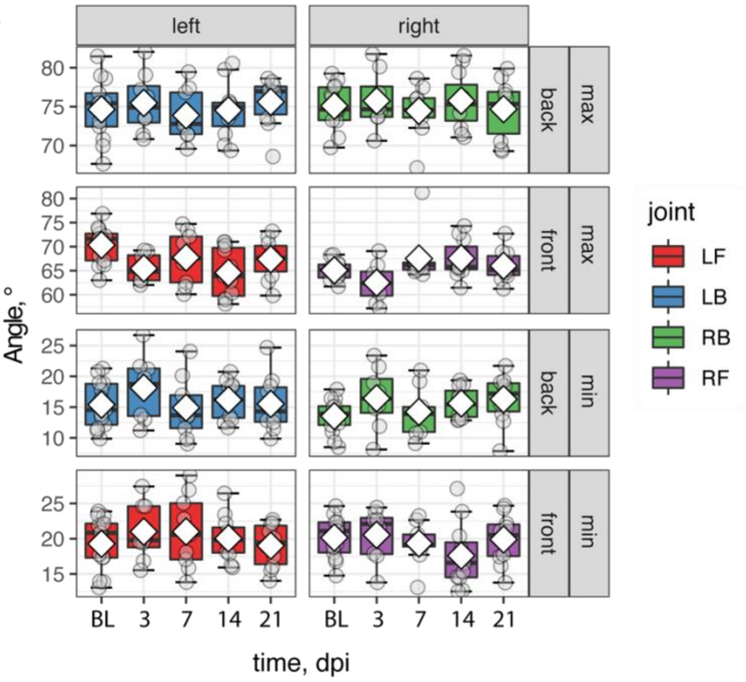

Suppl. Fig 4: Angular variability between body center and front and hind paws. (A) Comparison of angles of individual paws to body center in a time course. Data are shown as mean distributions where the white dot represents the mean. Boxplots indicate the 25% to 75% quartiles of the data. Each dot in the plots represents one animal and significance of mean differences between the groups was assessed using Tukey's HSD.

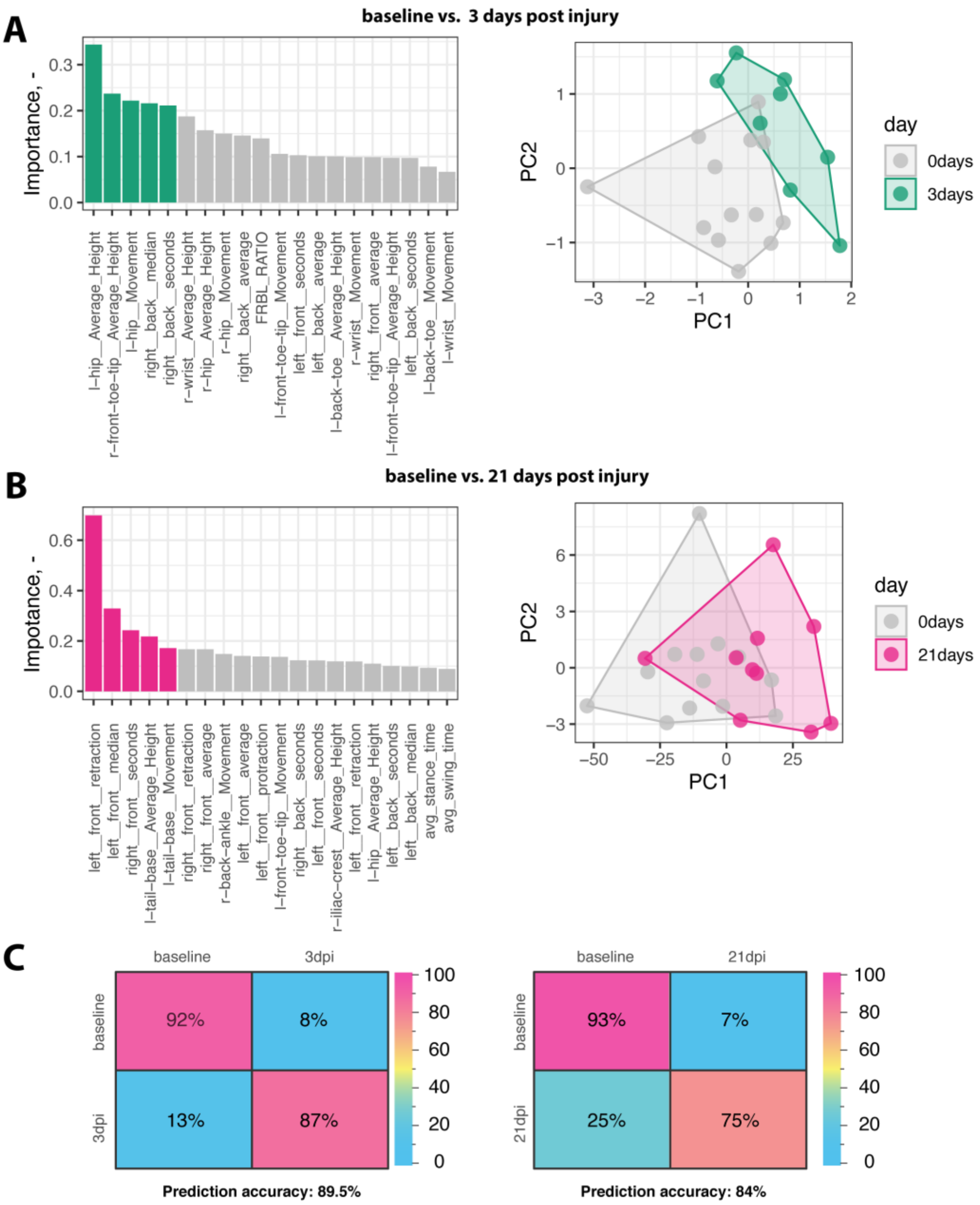

Suppl. Fig 5: Subgroup analysis for random forest classification and principal component analysis. (A) Random Forest classification of most important parameters (Gini impurity-based feature importance) between baseline and 3 dpi and principal component plot. (**B**) Random Forest classification of most important parameters Gini impurity-based feature importance between baseline and 21 dpi and principal component plot. (C) Confusion matrix and prediction accuracy of these models. Each dot in principal component analysis represents a video of individual animals.

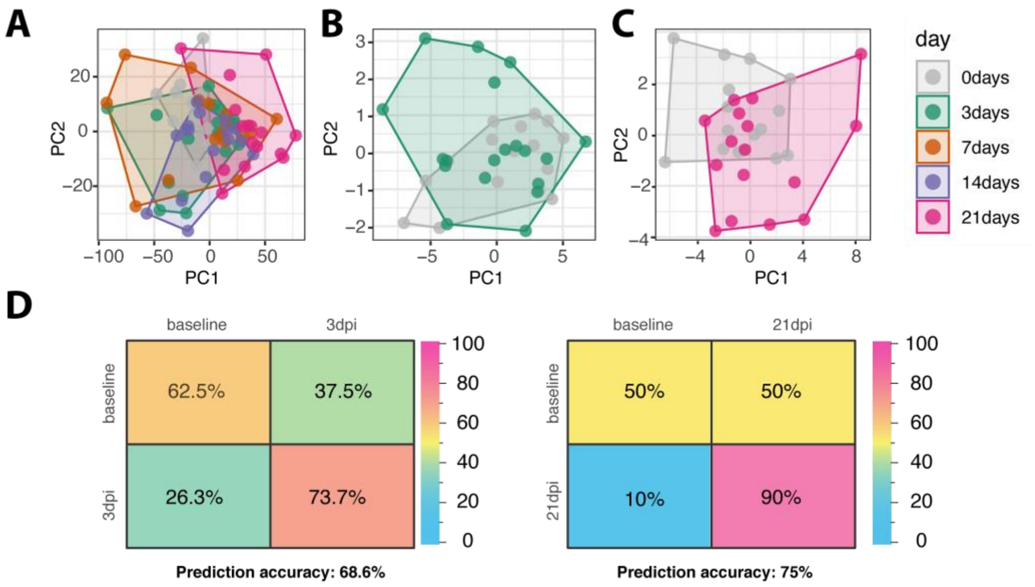

Suppl. Fig 6: Principal component analysis and random forest classification of uninjured control mice. (**A**) Principal component analysis of intact mice at day 0, 3, 7, 14, 21 and subgroup analysis of (B) 0 days and 3 days or (**C**) 0 days and 21 days with parameters previously generated from stroked mice. (**D**) Confusion matrix and prediction accuracy of a random forest classification in uninjured mice from 0 days and 3 days (left) and 0 days and 21 days (right). Each dot in principal component analysis represents a video of individual animals.

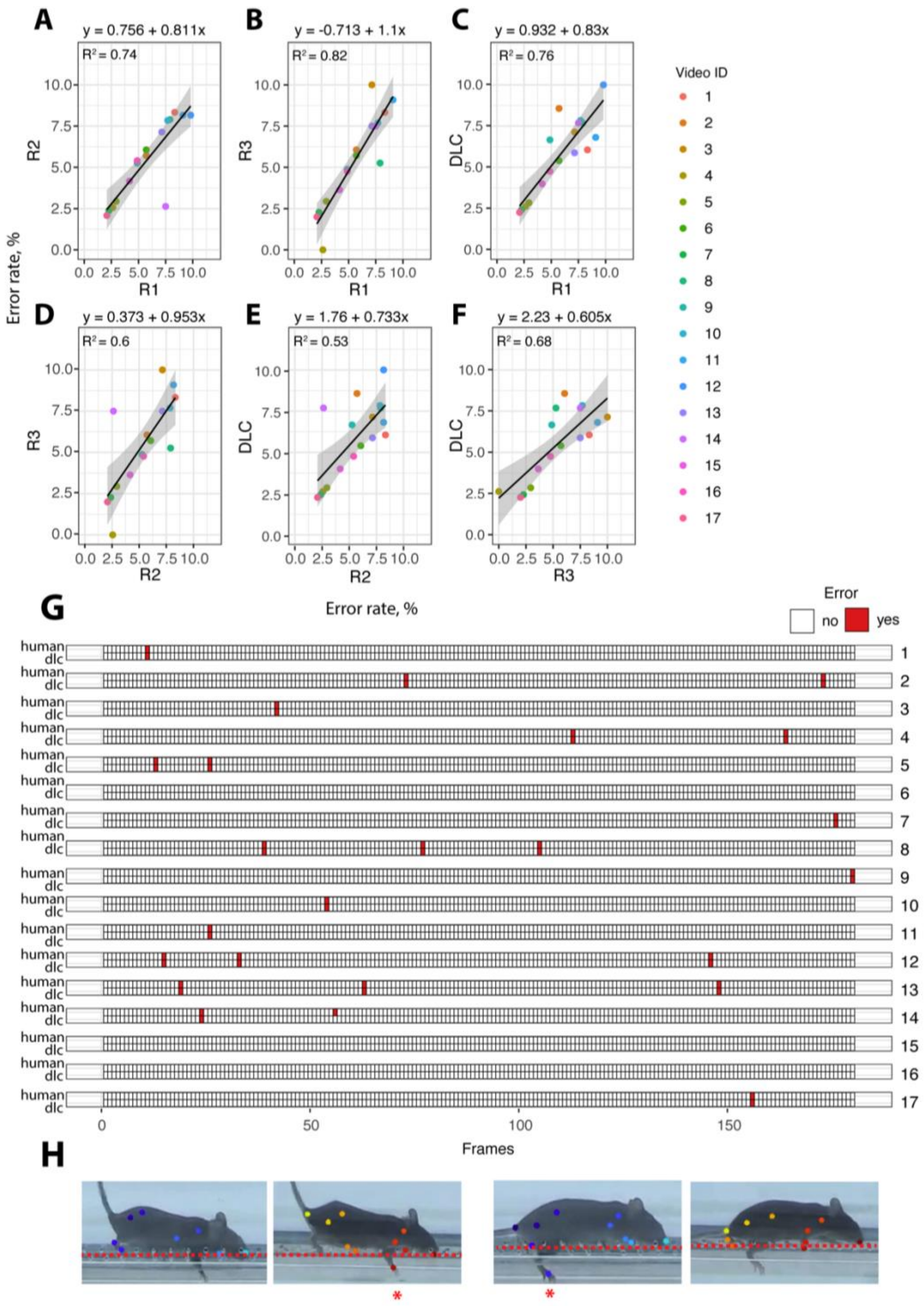

Suppl. Fig 7: Correlation plots between human annotators and DeepLabCut evaluation of the ladder rung test. (A) Correlation plot between Rater 1 and Rater 2. (B) Correlation plot between rater 1 and rater 3. (C) Correlation plot between rater 1 and rater 3. (D) Correlation plot between rater 2 and rater 3. (E) Correlation plot between DLC and rater 2. (F) Correlation plot between rater 3 and rater DLC. Individual dots represent randomly selected videos of both injured and non-injured mice. (G) Frame-by-Frame evaluation of missteps in selected videos (1-17) between human annotator and DLC-assisted tracking. (H) Representative recognition of missteps by DLC-assisted tracking.

A

initial overhead for DLC-assisted analysis

Process	Estimated time
optimising hardware set-up and camera settings	2-3 h
installation and set-up of DLC software	2 h
labeling and training neural network	12-48 h
refinement and validation of network	2 h
customizing post-hoc analysis	4 -6 h

Suppl Fig. 8: Overview of overhead for establishing DLC-assisted analysis.

A

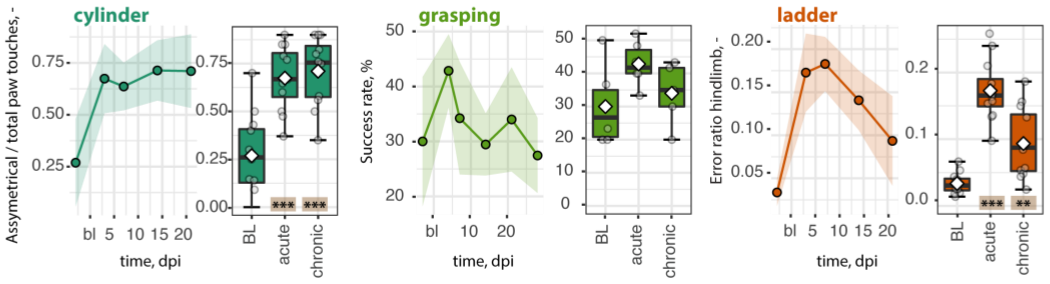

B

Parameter \ Criteria	neurol. score	rota-rod	cylinder test	pellet grasping	ladder rung test	ladder + DLC	runway test	runway + DLC
duration to perform task	10	8	7	1	4	4	3	8
sensitivity of task	2	2	5	8	6	6	10	10
# of readouts	1	2	3	5	3	3	10	10
objectivity / reproducibility	1	3	3	3	5	9.5	6	6
detection of long-term deficits	0	0	3	10	8	8	10	10
duration of post-hoc analysis	10	5	2	3	3	7	1	10
intensity of pre-training	10	3	7	1	5	5	9	9
low costs for set-up	10	8	9.5	9	8	8	0.5	8

C

Parameter \ Criteria	low (0-2)	average (3-7)	high (8-10)
duration to perform task	> 10 min / mouse	2 - 10 min / mouse	< 2 min / mouse
sensitivity of task	obvious deficits, only	moderate deficits	fine-motor deficits
# of readouts	1 readout	1-5 readout(s)	> 5 readouts
objectivity / reproducibility	highly variable, user-dependent	requires precise guidelines to ensure reproducibility	user-independent
detection of long-term deficits	no	only in big strokes, some animals	yes
duration of post-hoc analysis	> 10 min / mouse	2 - 10 min / mouse	< 2 min / mouse
intensity of pre-training	days-weeks of pre-training	several, short pre-trainings	no pre-training required
low costs for set-up	> 1500 USD	500 - 1500 USD	< 500 USD

Suppl. Fig 9: Functional assessment of recovery after stroke using conventional behavioral tests (**A**) Time course of recovery after stroke for asymmetry in the cylinder test (left), successful grasp and retrieval of the pellet in the single pellet grasping task (middle) and hindlimb errors in the ladder rung test (right). (**B**) Semi-quantitative scoring of assessment criteria including duration, sensitivity, number of readouts, reproducibility, detection of long-term deficits, duration of post-hoc analysis, intensity of pre-training and overall costs. (**C**) Description of scoring criteria for behavioral test comparison.

A

Label ID	Label name	Label description
1	Tail	Base of the tail; ventral to the caudal vertebrae.
2	Hip	Anterior to tail base; 15° angle from tail-shoulder-connecting line / straight above knee
3	Iliac crest	Anterior/dorsal to hip
4	Back ankle	Bend in left/right hind leg
5	Back toe tip	Toes on left/right hind paw
6	Shoulder	At level of tail base; anterior or posterior to elbow depending on movement.
7	Elbow	Underneath shoulder; depending on position of animal.
8	Wrist	Slight bend between left/right-front-paw and left/right-elbow.
9	Front toe tip	Toes on left/right front paw
10	Head	Nose tip

B

Label ID	Label name	Label description
1	Tail	Base of the tail; ventral to the caudal vertebrae.
2	Back right paw	Middle of the paw inside; hind right.
3	Back left paw	Middle of the paw inside; hind left.
4	Front right paw	Middle of the paw inside; front right.
5	Front left paw	Middle of the paw inside; front left.
6	Center back	Center of the connecting line back-right-paw and back-left-paw.
7	Center front	Center of the connecting line front-right-paw and front-left-paw.
8	Head	Nose tip

C

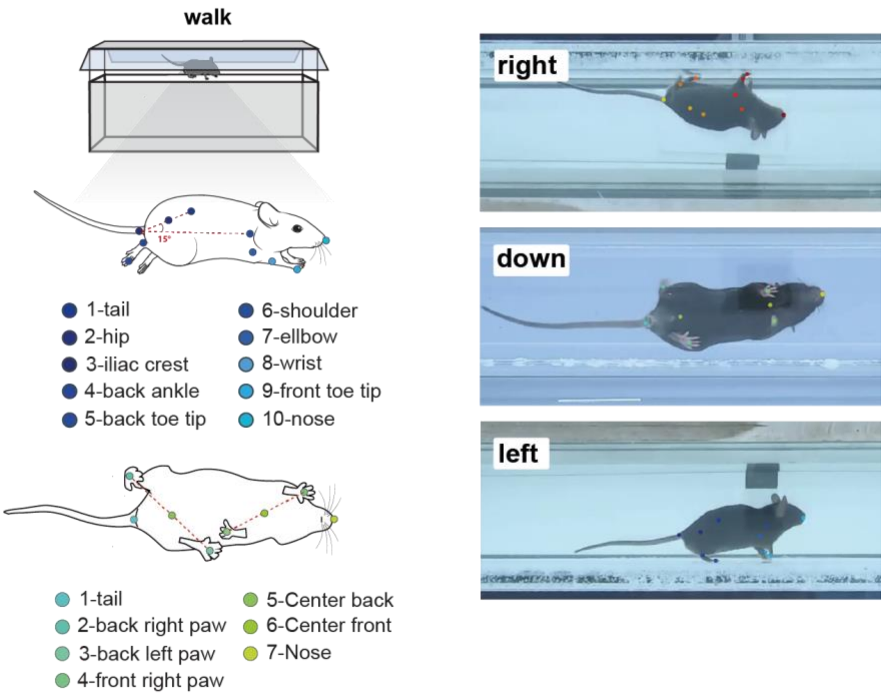

Suppl. Fig. 10: Manual labeling of joints and body parts for DLC. (A) Label name and description from the side (left and right) perspective (B) Label name and description from the down perspective (C) Schematic overview of labeling from right, down and bottom perspective.
