# Supplementary material for: Deep learning-based behavioral profiling of rodent stroke recovery
Source: BMC Biol. 2022 Oct 15;20:232. doi: 10.1186/s12915-022-01434-9 (PMC9571460; doi:10.1186/s12915-022-01434-9)
Supplement: Supplementary file 2 — Additional file 2: Table S1. Name and description of all generated parameters following runway performance. Table S2. Overview of sample size for each experiment. Tables S3-S37. Raw data, summarized data sets, and statistical data of data sets generated in this study. [file 12915_2022_1434_MOESM2_ESM.pdf]

Suppl. Table 1: Name and description of all generated parameters following runway performance

No	Prameter Cluster	Parameter Name	Parameter Description	Calculation	Formula Explanation
1	**synchronization and temporal features**	average duration	average duration to perform a total step with all four paws, (s)	average(time(Step(X)) - time(Step(X-1)))	Average time between Step X and the previous Step X-1
2		average stance time	average stance time during a step, (s)	average(time(StanceEnd(X)) - time(StanceBegin(X)))	Average time required for a stance (StanceEnd - StanceBegin) during one step
3		average swing time	average swing time during a step, (s)	average(time(SwingEnd(X)) - time(SwingBegin(X)))	Average time required for a swing (SwingEnd - SwingBegin) during one step
4		left back duration	average duration of a step in the left back paw (LB), (s)	average(time(StepLB(X)) - time(StepLB (X-1)))	Average time between Step X and the previous Step X-1 of left back paw
5		left front duration	average duration of a step in the left front paw (LF), (s)	average(time(StepLF(X)) - time(StepLF (X-1)))	Average time between Step X and the previous Step X-1 of left front paw
6		right back duration	average duration of a step in the right back paw (RB), (s)	average(time(StepRB(X)) - time(StepRB (X-1)))	Average time between Step X and the previous Step X-1 of right back paw
7		right front duration	average duration of a step in the right front paw (RF), (s)	average(time(StepRF(X)) - time(StepRF (X-1)))	Average time between Step X and the previous Step X-1 of right front paw
8		average stride length	average length of a step from down view, (mm)	average(length(Step(X)) - length(Step(X-1)))	Average distance covered between Step X and the previous Step X-1
9		left front back right synchronization	asynchronization ratio between left front paw (LF) and right back paw (RB), 0 = complete symmetry, normal gait (-)	asyncLFRB = 1 - time(syncLFRB/time(syncLFRB+asyncLFRB)	Normal gait: synchronization of stance phase LF+RB and RF+LB. Asynchronization ratio is 1- the time stance of LFRB was synchronized divided by total stance time (syncLFRB + asyncLFRB)
10		right front back left synchronization	asynchronization ratio between right front paw (RF) and left back paw (LB), 0 = complete symmetry, normal gait (-)	asyncRFLB = 1 - time(syncRFLB/time(syncRFLB+asyncRFLB)	Normal gait: synchronization of stance phase LF+RB and RF+LB. Asynchronization ratio is 1 - the time stance of RFLB was synchronized divided by total stance time (syncRFLB + asyncRFLB)

11	**vertical joint movement and heights**	left back ankle average height	average height changes during a step of the left back ankle compared to baseline = 0 , (mm)	average((height of body part)) / Step	Average relative height /y-axis coordinate of body part per step. (normalized to baseline)
12		left back ankle vertical movement	total vertical movement changes during a step of the left back ankle compared to baseline = 0, (mm)	max(height of body part) - min(height of body part) / Step	Vertical movement is the highest position of the body part during a step subtracted by the lowest position. (normalized to baseline)
13		left back toe tip average height	average height changes during a step of the left back toe tip compared to baseline = 0, (mm)	average((height of body part)) / Step	Average relative height /y-axis coordinate of body part per step. (normalized to baseline)
14		left back toe tip vertical movement	total vertical movement changes during a step of the left back toe tip compared to baseline = 0, (mm)	max(height of body part) - min(height of body part) / Step	Vertical movement is the highest position of the body part during a step subtracted by the lowest position. (normalized to baseline)
15		left front toe tip average height	average height changes during a step of the left front toe tip compared to baseline = 0, (mm)	average((height of body part)) / Step	Average relative height /y-axis coordinate of body part per step. (normalized to baseline)
16		left front toe tip vertical movement	total vertical movement changes during a step of the left front toe tip compared to baseline = 0, (mm)	max(height of body part) - min(height of body part) / Step	Vertical movement is the highest position of the body part during a step subtracted by the lowest position. (normalized to baseline)
17		left head average height	average height changes during a step of the head from left side compared to baseline = 0, (mm)	average((height of body part)) / Step	Average relative height /y-axis coordinate of body part per step. (normalized to baseline)
18		left head vertical movement	total vertical movement changes during a step of the head from left side compared to baseline = 0, (mm)	max(height of body part) - min(height of body part) / Step	Vertical movement is the highest position of the body part during a step subtracted by the lowest position. (normalized to baseline)
19		left hip average height	average height changes during a step of the left hip compared to baseline = 0, (mm)	average((height of body part)) / Step	Average relative height /y-axis coordinate of body part per step. (normalized to baseline)
20		left hip vertical movement	total vertical movement changes during a step of the left hip compared to baseline = 0, (mm)	max(height of body part) - min(height of body part) / Step	Vertical movement is the highest position of the body part during a step subtracted by the lowest position. (normalized to baseline)
21		left iliac crest average height	average height changes during a step of the left iliac crest compared to baseline = 0, (mm)	average((height of body part)) / Step	Average relative height /y-axis coordinate of body part per step. (normalized to baseline)
22		left iliac crest vertical movement	total vertical movement changes during a step of the left iliac crest compared to baseline = 0, (mm)	max(height of body part) - min(height of body part) / Step	Vertical movement is the highest position of the body part during a step subtracted by the lowest position. (normalized to baseline)

23	**vertical joint movement and heights**	left tail base average height	average height changes during a step of the left tail base compared to baseline = 0, (mm)	average((height of body part)) / Step	Average relative height /y-axis coordinate of body part per step. (normalized to baseline)
24		left tail base vertical movement	total vertical movement changes during a step of the left tail base compared to baseline = 0, (mm)	max(height of body part) - min(height of body part) / Step	Vertical movement is the highest position of the body part during a step subtracted by the lowest position. (normalized to baseline)
25		left wrist average height	average height changes during a step of the left wrist compared to baseline = 0, (mm)	average((height of body part)) / Step	Average relative height /y-axis coordinate of body part per step. (normalized to baseline)
26		left wrist vertical movement	total vertical movement changes during a step of the left wrist compared to baseline = 0, (mm)	max(height of body part) - min(height of body part) / Step	Vertical movement is the highest position of the body part during a step subtracted by the lowest position. (normalized to baseline)
27		right back ankle average height	average height changes during a step of the right back ankle compared to baseline = 0, (mm)	average((height of body part)) / Step	Average relative height /y-axis coordinate of body part per step. (normalized to baseline)
28		right back ankle vertical movement	total vertical movement changes during a step of the left back ankle compared to baseline = 0, (mm)	max(height of body part) - min(height of body part) / Step	Vertical movement is the highest position of the body part during a step subtracted by the lowest position. (normalized to baseline)
29		right back toe tip average height	average height changes during a step of the right back toe tip compared to baseline = 0, (mm)	average((height of body part)) / Step	Average relative height /y-axis coordinate of body part per step. (normalized to baseline)
30		right back toe tip vertical movement	total vertical movement changes during a step of the right back toe tip compared to baseline = 0, (mm)	max(height of body part) - min(height of body part) / Step	Vertical movement is the highest position of the body part during a step subtracted by the lowest position. (normalized to baseline)
31		right front toe tip average height	average height changes during a step of the right front toe tip compared to baseline = 0, (mm)	average((height of body part)) / Step	Average relative height /y-axis coordinate of body part per step. (normalized to baseline)
32		right front toe tip vertical movement	total vertical movement changes during a step of the right front toe tip compared to baseline = 0, (mm)	max(height of body part) - min(height of body part) / Step	Vertical movement is the highest position of the body part during a step subtracted by the lowest position. (normalized to baseline)
33		right head average height	average height changes during a step of the head from right side compared to baseline = 0, (mm)	average((height of body part)) / Step	Average relative height /y-axis coordinate of body part per step. (normalized to baseline)
34		right head vertical movement	total vertical movement changes during a step of the head from right side compared to baseline = 0, (mm)	max(height of body part) - min(height of body part) / Step	Vertical movement is the highest position of the body part during a step subtracted by the lowest position. (normalized to baseline)
35		right hip average height	average height changes during a step of the right hip compared to baseline = 0, (mm)	average((height of body part)) / Step	Average relative height /y-axis coordinate of body part per step. (normalized to baseline)

36	**vertical joint movement and heights**	right hip vertical movement	total vertical movement changes during a step of the right hip compared to baseline = 0, (mm)	max(height of body part) - min(height of body part) / Step	Vertical movement is the highest position of the body part during a step subtracted by the lowest position. (normalized to baseline)
37		right iliac crest average height	average height changes during a step of the right iliac crest compared to baseline = 0, (mm)	average((height of body part)) / Step	Average relative height /y-axis coordinate of body part per step. (normalized to baseline)
38		right iliac crest vertical movement	total vertical movement changes during a step of the right iliac crest compared to baseline = 0, (mm)	max(height of body part) - min(height of body part) / Step	Vertical movement is the highest position of the body part during a step subtracted by the lowest position. (normalized to baseline)
39		right tail base average height	average height changes during a step of the right tail base compared to baseline = 0, (mm)	average((height of body part)) / Step	Average relative height /y-axis coordinate of body part per step. (normalized to baseline)
40		right tail base vertical movement	total vertical movement changes during a step of the right tail base compared to baseline = 0, (mm)	max(height of body part) - min(height of body part) / Step	Vertical movement is the highest position of the body part during a step subtracted by the lowest position. (normalized to baseline)
41		right wrist average height	average height changes during a step of the right wrist compared to baseline = 0, (mm)	average((height of body part)) / Step	Average relative height /y-axis coordinate of body part per step. (normalized to baseline)
42		right wrist vertical movement	total vertical movement changes during a step of the right wrist compared to baseline = 0, (mm)	max(height of body part) - min(height of body part) / Step	Vertical movement is the highest position of the body part during a step subtracted by the lowest position. (normalized to baseline)
43	**step length, protraction and retraction**	left back average length	average length of a step of the left back paw (LB) compared to baseline = 0, (mm)	average(distance(EndStepLB-BeginnStepLB))	Average distance/x-axis coordinates covered by left back paw during one step
44		left back median length	median length of a step of the left back paw (LB) compared to baseline = 0, (mm)	median(distance(EndStepLB-BeginnStepLB))	Median distance/x-axis coordinates covered by left back paw during one step
45		left back total horizontal movement	total horizontal movement during a step of the left back paw (LB) compared to baseline = 0, (mm)	max(distance(EndStepLB-BeginnStepLB))	Max distance/x-axis coordinates covered by left back paw during one step
46		left back protraction max	maximum protractive movement during a step of the left back paw (LB) compared to baseline = 0, (mm)	max(distance(EndProtractStepLB-BeginnProtractStepLB))	Protraction is defined when x-coordinates between 2 frames was positive. Max distance covered by left back paw during protraction of step
47		left back retraction max	maximum retractive movement during a step of the left back paw (LB) compared to baseline = 0, (mm)	max(distance(EndRetractStepLB-BeginnRetractStepLB))	Retraction is defined when x-coordinates between 2 frames was negative. Max distance covered by left back paw during retraction of step
48		left front average length	average length of a step of the left front paw (LF) compared to baseline = 0, (mm)	average(distance(EndStepLF-BeginnStepLF))	Average distance/x-axis coordinates covered by left front paw during one step

49	**step length, protraction and retraction**	left front median length	median length of a step of the left front paw (LF) compared to baseline = 0, (mm)	median(distance(EndStepLF-BeginnStepLF))	Median distance/x-axis coordinates covered by left front paw during one step
50		left front total horizontal movement	total horizontal movement during a step of the left front paw (LF) compared to baseline = 0, (mm)	max(distance(EndStepLF-BeginnStepLF))	Max distance/x-axis coordinates covered by left front paw during one step
51		left front protraction max	maximum protractive movement during a step of the left front paw (LF) compared to baseline = 0, (mm)	max(distance(EndProtractStepLF-BeginnProtractStepLF))	Protraction is defined when x-coordinates between 2 frames was positive. Max distance covered by left front paw during protraction of step
52		left front retraction max	maximum retractive movement during a step of the left front paw (LF) compared to baseline = 0, (mm)	max(distance(EndRetractStepLF-BeginnRetractStepLF))	Retraction is defined when x-coordinates between 2 frames was negative. Max distance covered by left front paw during retraction of step
53		right back average length	average length of a step of the right back paw (RB) compared to baseline = 0, (mm)	average(distance(EndStepRB-BeginnStepLF))	Average distance/x-axis coordinates covered by right back paw during one step
54		right back median length	median length of a step of the right back paw (RB) compared to baseline = 0, (mm)	median(distance(EndStepRB-BeginnStepRB))	Median distance/x-axis coordinates covered by right back paw during one step
55		right back total horizontal movement	total horizontal movement during a step of the right back paw (RB) compared to baseline = 0, (mm)	max(distance(EndStepRB-BeginnStepRB))	Max distance/x-axis coordinates covered by right back paw during one step
56		right back protraction max	maximum protractive movement during a step of the right back paw (RB) compared to baseline = 0, (mm)	max(distance(EndProtractStepRB-BeginnProtractStepRB))	Protraction is defined when x-coordinates between 2 frames was positive. Max distance covered by right back paw during protraction of step
57		right back retraction max	maximum retractive movement during a step of the right back paw (RB) compared to baseline = 0, (mm)	max(distance(EndRetractStepRB-BeginnRetractStepRB))	Retraction is defined when x-coordinates between 2 frames was negative. Max distance covered by right back paw during retraction of step
58		right front average length	average length of a step of the right front paw (RF) compared to baseline = 0, (mm)	average(distance(EndStepRF-BeginnStepRF))	Average distance/x-axis coordinates covered by right front paw during one step
59		right front median length	median length of a step of the right front paw (RF) compared to baseline = 0, (mm)	median(distance(EndStepRF-BeginnStepRF))	Median distance/x-axis coordinates covered by right front paw during one step
60		right front total horizontal movement	total horizontal movement during a step of the right front paw (RF) compared to baseline = 0, (mm)	max(distance(EndStepRF-BeginnStepRF))	Max distance/x-axis coordinates covered by right front paw during one step
61		right front protraction max	maximum protractive movement during a step of the right front paw (RF) compared to baseline = 0, (mm)	max(distance(EndProtractStepRF-BeginnProtractStepRF))	Protraction is defined when x-coordinates between 2 frames was positive. Max distance covered by right front paw during protraction of step

62		right front retraction max	maximum retractive movement during a step of the right front paw (RF) compared to baseline = 0, (mm)	max(distance(EndRetractStepRF-BeginnRetractStepRF))	Retraction is defined when x-coordinates between 2 frames was negative. Max distance covered by right front paw during retraction of step
63		left hip total horizontal movement	total horizontal movement during a step of the left hip compared to baseline = 0, (mm)	max(distance(EndStepLeftHip-BeginnStepLeftHip))	Max distance/x-axis coordinates covered by left hip during one step
64		left hip average length	average distance covered by the left hip during a step compared to baseline = 0, (mm)	average(distance(EndStepLeftHip-BeginnStepLeftHip))	Average distance/x-axis coordinates covered by left hip during one step
65		left tail base total horizontal movement	total horizontal movement during a step of the left tail base compared to baseline = 0, (mm)	max(distance(EndStepLeftTail-BeginnStepLeftTail))	Max distance/x-axis coordinates covered by left tail base during one step
66		left tail base average length	average distance covered by the left tail base during a step compared to baseline = 0, (mm)	average(distance(EndStepLeftTail-BeginnStepLeftTail))	Average distance/x-axis coordinates covered by left tail base during one step
67		right hip total horizontal movement	total horizontal movement during a step of the right hip compared to baseline = 0, (mm)	max(distance(EndStepRightHip-BeginnStepRightHip))	Max distance/x-axis coordinates covered by right hip during one step
68		right hip average length	average distance covered by the right hip during a step compared to baseline = 0, (mm)	average(distance(EndStepRightHip-BeginnStepRightHip))	Average distance/x-axis coordinates covered by right hip during one step
69		right tail base total horizontal movement	total horizontal movement during a step of the right tail base compared to baseline = 0, (mm)	max(distance(EndStepRightTail-BeginnStepRightTail))	Max distance/x-axis coordinates covered by right tail base during one step
70		right tail base average length	average distance covered by the right tail base during a step compared to baseline = 0, (mm)	average(distance(EndStepRightTail-BeginnStepRightTail))	Average distance/x-axis coordinates covered by right tail base during one step
71	**paw to body center angles**	left back average angle	average angle between body center back (CB) and left back paw (LB) during a step from down view, (°)	average(atan2(LB.y - CB.y,LB.x - CB.x)) / Step	Arctan was used to calculate angle between the landmark coordinates (X/Y) of body center and paw. Average angle was calculated per step.
72		left back max angle	maximum angle between body center back (CB) and left back paw (LB) during a step from down view, (°)	max(atan2(LB.y - CB.y,LB.x - CB.x)) / Step	Arctan was used to calculate angle between the landmark coordinates (X/Y) of body center and paw. Max angle was calculated per step.
73		left back min angle	minimum angle between body center back (CB) and left back paw (LB) during a step from down view, (°)	min(atan2(LB.y - CB.y,LB.x - CB.x)) / Step	Arctan was used to calculate angle between the landmark coordinates (X/Y) of body center and paw. Min angle was calculated per step.
74		left front average angle	average angle between body center front (CF) and left front paw (LF) during a step from down view, (°)	average(atan2(LF.y - BC.y,LF.x - BC.x)) / Step	Arctan was used to calculate angle between the landmark coordinates (X/Y) of body center and paw. Average angle was calculated per step.

75	**paw to body center angles**	left front max angle	maximum angle between body center front (CF) and left front paw (LF) during a step from down view, (°)	max(atan2(LF.y - BC.y,LF.x - BC.x)) / Step	Arctan was used to calculate angle between the landmark coordinates (X/Y) of body center and paw. Max angle was calculated per step.
76		left front min angle	minimum angle between body center front (CF) and left front paw (LF) during a step from down view, (°)	min(atan2(LF.y - BC.y,LF.x - BC.x)) / Step	Arctan was used to calculate angle between the landmark coordinates (X/Y) of body center and paw. Min angle was calculated per step.
77		right back average angle	average angle between body center back (CB) and right back paw (RB) during a step from down view, (°)	average(atan2(RB.y - CB.y,RB.x - CB.x)) / Step	Arctan was used to calculate angle between the landmark coordinates (X/Y) of body center and paw. Average angle was calculated per step.
78		right back max angle	maximum angle between body center back (CB) and right back paw (RB) during a step from down view, (°)	max(atan2(RB.y - CB.y,RB.x - CB.x)) / Step	Arctan was used to calculate angle between the landmark coordinates (X/Y) of body center and paw. Max angle was calculated per step.
79		right back min angle	minimum angle between body center back (CB) and right back paw (RB) during a step from down view, (°)	min(atan2(RB.y - CB.y,RB.x - CB.x)) / Step	Arctan was used to calculate angle between the landmark coordinates (X/Y) of body center and paw. Min angle was calculated per step.
80		right front average angle	average angle between body center front (CF) and right front paw (RF) during a step from down view, (°)	average(atan2(RF.y - BC.y,RF.x - BC.x)) / Step	Arctan was used to calculate angle between the landmark coordinates (X/Y) of body center and paw. Average angle was calculated per step.
81		right front max angle	maximum angle between body center front (CF) and right front paw RF) during a step from down view, (°)	max(atan2(RF.y - BC.y,RF.x - BC.x)) / Step	Arctan was used to calculate angle between the landmark coordinates (X/Y) of body center and paw. Max angle was calculated per step.
82		right front min angle	minimum angle between body center front (CF) and right front paw (RF) during a step from down view, (°)	min(atan2(RF.y - BC.y,RF.x - BC.x)) / Step	Arctan was used to calculate angle between the landmark coordinates (X/Y) of body center and paw. Min angle was calculated per step.
83	**joint angles**	average angle left back hip ankle toe	average angle between left hip, left back ankle and left back toe tip during a step from side view, (°)	average(atan2((back-toe.y-back-ankle.y), (back-toe.x-back-ankle.x))- atan2((hip.y-back-ankle.y), (hip.x-back-ankle.x))) / Step	Arctan was used to calculate angle between the three left landmark coordinates (X/Y) of hip, back ankle and back toe tip. Average angle was calculated per [illegible]
84		average angle left back iliac hip ankle	average angle between left iliac crest, left hip and left back ankle during a step from side view, (°)	average(atan2((back-ankle.y-hip.y), (back-ankle.x-hip.x))- atan2((iliac-crest.y-hip.y), (iliac-crest.x-hip.x))) / Step	Arctan was used to calculate angle between the three left landmark coordinates (X/Y) of iliac-crest, hip, back ankle. Average angle was calculated per step.
85		average angle left front elbow wrist toetip	average angle between left elbow, left wrist and left front toe tip during a step from side view, (°)	average(atan2((front-toe-tip.y-wrist.y), (front-toe-tip.x-wrist.x))- atan2((elbow.y-wrist.y), (elbow.x-wrist.x))) / Step	Arctan was used to calculate angle between the three left landmark coordinates (X/Y) of elbow, wrist, front toe tip. Average angle was calculated per step.
86		average angle left front shoulder elbow wrist	average angle between left shuolder, left elbow and left wrist during a step from side view, (°)	average(atan2((wrist.y-elbow.y), (wrist.x-elbow.x))- atan2((shoulder.y-elbow.y), (shoulder.x-elbow.x))) / Step	Arctan was used to calculate angle between the three left landmark coordinates (X/Y) of shoulder, elbow, wrist. Average angle was calculated per step.
87		max left back hip ankle toe	maximum angle between left hip, left back ankle and left back toe tip during a step from side view, (°)	max(atan2((back-toe.y-back-ankle.y), (back-toe.x-back-ankle.x))- atan2((hip.y-back-ankle.y), (hip.x-back-ankle.x))) / Step	Arctan was used to calculate angle between the three left landmark coordinates (X/Y) of hip, back ankle and back toe tip. Max angle was calculated per step.

88	**joint angles**	max left back iliac hip ankle	maximum angle between left iliac crest, left hip and left back ankle during a step from side view, (°)	max(atan2((back-ankle.y-hip.y), (back-ankle.x-hip.x))- atan2((iliac-crest.y-hip.y), (iliac-crest.x-hip.x))) / Step	Arctan was used to calculate angle between the three left landmark coordinates (X/Y) of iliac-crest, hip, back ankle. Max angle was calculated per step.
89		max left front elbow wrist toetip	maximum angle between left elbow, left wrist and left front toe tip during a step from side view, (°)	max(atan2((front-toe-tip.y-wrist.y), (front-toe-tip.x-wrist.x))- atan2((elbow.y-wrist.y), (elbow.x-wrist.x)) / Step	Arctan was used to calculate angle between the three left landmark coordinates (X/Y) of elbow, wrist, front toe tip. Max angle was calculated per step.
90		max left front shoulder elbow wrist	maximum angle between left shuolder, left elbow and left wrist during a step from side view, (°)	max(atan2((wrist.y-elbow.y), (wrist.x-elbow.x))- atan2((shoulder.y-elbow.y), (shoulder.x-elbow.x)) / Step	Arctan was used to calculate angle between the three left landmark coordinates (X/Y) of shoulder, elbow, wrist. Max angle was calculated per step.
91		min left back hip ankle toe	minimum angle between left hip, left back ankle and left back toe tip during a step from side view, (°)	min(atan2((back-toe.y-back-ankle.y), (back-toe.x-back-ankle.x))- atan2((hip.y-back-ankle.y), (hip.x-back-ankle.x))) / Step	Arctan was used to calculate angle between the three left landmark coordinates (X/Y) of hip, back ankle and back toe tip. Min angle was calculated per step.
92		min left back iliac hip ankle	minimum angle between left iliac crest, left hip and left back ankle during a step from side view, (°)	min(atan2((back-ankle.y-hip.y), (back-ankle.x-hip.x))- atan2((iliac-crest.y-hip.y), (iliac-crest.x-hip.x))) / Step	Arctan was used to calculate angle between the three left landmark coordinates (X/Y) of iliac-crest, hip, back ankle. Min angle was calculated per step.
93		min left front elbow wrist toetip	minimum angle between left elbow, left wrist and left front toe tip during a step from side view, (°)	min(atan2((front-toe-tip.y-wrist.y), (front-toe-tip.x-wrist.x))- atan2((elbow.y-wrist.y), (elbow.x-wrist.x)) / Step	Arctan was used to calculate angle between the three left landmark coordinates (X/Y) of elbow, wrist, front toe tip. Min angle was calculated per step.
94		min left front shoulder elbow wrist	minimum angle between left shuolder, left elbow and left wrist during a step from side view, (°)	min(atan2((wrist.y-elbow.y), (wrist.x-elbow.x))- atan2((shoulder.y-elbow.y), (shoulder.x-elbow.x)) / Step	Arctan was used to calculate angle between the three left landmark coordinates (X/Y) of shoulder, elbow, wrist. Min angle was calculated per step.
95		average angle right back hip ankle toe	average angle between right hip, right back ankle and right back toe tip during a step from side view, (°)	average(atan2((back-toe.y-back-ankle.y), (back-toe.x-back-ankle.x))- atan2((hip.y-back-ankle.y), (hip.x-back-ankle.x))) / Step	Arctan was used to calculate angle between the three left landmark coordinates (X/Y) of hip, back ankle and back toe tip. Average angle was calculated per [illegible]
96		average angle right back iliac hip ankle	average angle between right iliac crest, right hip and right back ankle during a step from side view, (°)	average(atan2((back-ankle.y-hip.y), (back-ankle.x-hip.x))- atan2((iliac-crest.y-hip.y), (iliac-crest.x-hip.x))) / Step	Arctan was used to calculate angle between the three right landmark coordinates (X/Y) of iliac-crest, hip, back ankle. Average angle was calculated per step.
97		average angle right front elbow wrist toetip	average angle between right elbow, right wrist and right front toe tip during a step from side view, (°)	average(atan2((front-toe-tip.y-wrist.y), (front-toe-tip.x-wrist.x))- atan2((elbow.y-wrist.y), (elbow.x-wrist.x))) / Step	Arctan was used to calculate angle between the three right landmark coordinates (X/Y) of elbow, wrist, front toe tip. Average angle was calculated per step.
98		average angle right front shoulder elbow wrist	average angle between right shuolder, right elbow and right wrist during a step from side view, (°)	average(atan2((wrist.y-elbow.y), (wrist.x-elbow.x))- atan2((shoulder.y-elbow.y), (shoulder.x-elbow.x))) / Step	Arctan was used to calculate angle between the three right landmark coordinates (X/Y) of shoulder, elbow, wrist. Average angle was calculated per step.
99		max right back hip ankle toe	maximum angle between right hip, right back ankle and right back toe tip during a step from side view, (°)	max(atan2((back-toe.y-back-ankle.y), (back-toe.x-back-ankle.x))- atan2((hip.y-back-ankle.y), (hip.x-back-ankle.x))) / Step	Arctan was used to calculate angle between the three right landmark coordinates (X/Y) of hip, back ankle and back toe tip. Max angle was calculated per step.
100		max right back iliac hip ankle	maximum angle between right iliac crest, right hip and right back ankle during a step from side view, (°)	max(atan2((back-ankle.y-hip.y), (back-ankle.x-hip.x))- atan2((iliac-crest.y-hip.y), (iliac-crest.x-hip.x))) / Step	Arctan was used to calculate angle between the three right landmark coordinates (X/Y) of iliac-crest, hip, back ankle. Max angle was calculated per step.

101	**joint angles**	max right front elbow wrist toetip	maximum angle between right elbow, right wrist and right front toe tip during a step from side view, (°)	max(atan2((front-toe-tip.y-wrist.y), (front-toe-tip.x-wrist.x))- atan2((elbow.y-wrist.y), (elbow.x-wrist.x)) / Step	Arctan was used to calculate angle between the three right landmark coordinates (X/Y) of elbow, wrist, front toe tip. Max angle was calculated per step.
102		max right front shoulder elbow wrist	maximum angle between right shuolder, right elbow and right wrist during a step from side view, (°)	max(atan2((wrist.y-elbow.y), (wrist.x-elbow.x))- atan2((shoulder.y-elbow.y), (shoulder.x-elbow.x)) / Step	Arctan was used to calculate angle between the three right landmark coordinates (X/Y) of shoulder, elbow, wrist. Max angle was calculated per step.
103		min right back hip ankle toe	minimum angle between right hip, right back ankle and right back toe tip during a step from side view, (°)	min(atan2((back-toe.y-back-ankle.y), (back-toe.x-back-ankle.x))- atan2((hip.y-back-ankle.y), (hip.x-back-ankle.x))) / Step	Arctan was used to calculate angle between the three right landmark coordinates (X/Y) of hip, back ankle and back toe tip. Min angle was calculated per step.
104		min right back iliac hip ankle	minimum angle between right iliac crest, right hip and right back ankle during a step from side view, (°)	min(atan2((back-ankle.y-hip.y), (back-ankle.x-hip.x))- atan2((iliac-crest.y-hip.y), (iliac-crest.x-hip.x))) / Step	Arctan was used to calculate angle between the three right landmark coordinates (X/Y) of iliac-crest, hip, back ankle. Min angle was calculated per step.
105		min right front elbow wrist toetip	minimum angle between right elbow, right wrist and right front toe tip during a step from side view, (°)	min(atan2((front-toe-tip.y-wrist.y), (front-toe-tip.x-wrist.x))- atan2((elbow.y-wrist.y), (elbow.x-wrist.x)) / Step	Arctan was used to calculate angle between the three right landmark coordinates (X/Y) of elbow, wrist, front toe tip. Min angle was calculated per step.
106		min right front shoulder elbow wrist	minimum angle between right shuolder, right elbow and right wrist during a step from side view, (°)	min(atan2((wrist.y-elbow.y), (wrist.x-elbow.x))- atan2((shoulder.y-elbow.y), (shoulder.x-elbow.x)) / Step	Arctan was used to calculate angle between the three right landmark coordinates (X/Y) of shoulder, elbow, wrist. Min angle was calculated per step.

Suppl. Table 2: Overview of sample size for each experiment

Overview	Description of experiment	Sample size N	Sex
**Validation of DeepLabCut set-up**	C57Bl/6 (1st batch) for training, set-up	8	4M / 4F
	C57Bl/6 (2nd batch) for validation, stroked	13	7M / 6F
	NSG (white fur)	12	6M/6F
**Stroke confirmation and tissue analysis**	Laser Doppler Imaging	8	4M /4F
	Stroke size analysis	8	4M /4F
**DeepLabCut analysis**	runway analysis	8-13	7M /6F
	ladder rung analysis	8-13	7M /6F
**Conventional tests**	Neurological score	7*	3M /4F
	Rotarod	12*	6M /6F
	Cylinder test	12*	6M /6F
	ladder rung test	12*	6M /6F
	single pellet grasping	6*	6F

* different cohort

Suppl. Table 3: Accuracy of labeling different mice during runway test

Group	Parameter	Likeliehood	Training Session	Setup
Bl6, original mice	d-back-left	100	Original Session 1	runway
Bl6, original mice	d-back-right	100	Original Session 1	runway
Bl6, original mice	d-front-left	100	Original Session 1	runway
Bl6, original mice	d-front-right	100	Original Session 1	runway
Bl6, original mice	d-head	100	Original Session 1	runway
Bl6, original mice	d-tail-base	100	Original Session 1	runway
Bl6, original mice	l-back-ankle	96.77419355	Original Session 1	runway
Bl6, original mice	l-back-toe	93.63057325	Original Session 1	runway
Bl6, original mice	l-elbow	100	Original Session 1	runway
Bl6, original mice	l-front-toe-tip	98.125	Original Session 1	runway
Bl6, original mice	l-head	100	Original Session 1	runway
Bl6, original mice	l-hip	100	Original Session 1	runway
Bl6, original mice	l-iliac-crest	100	Original Session 1	runway
Bl6, original mice	l-shoulder	99.35483871	Original Session 1	runway
Bl6, original mice	l-tail-base	100	Original Session 1	runway
Bl6, original mice	l-wrist	100	Original Session 1	runway
Bl6, original mice	r-back-ankle	99.35483871	Original Session 1	runway
Bl6, original mice	r-back-toe	100	Original Session 1	runway
Bl6, original mice	r-elbow	100	Original Session 1	runway
Bl6, original mice	r-front-toe-tip	96.73202614	Original Session 1	runway
Bl6, original mice	r-head	98.03921569	Original Session 1	runway
Bl6, original mice	r-hip	100	Original Session 1	runway
Bl6, original mice	r-iliac-crest	100	Original Session 1	runway
Bl6, original mice	r-shoulder	100	Original Session 1	runway
Bl6, original mice	r-tail-base	100	Original Session 1	runway
Bl6, original mice	r-wrist	99.35897436	Original Session 1	runway
Bl6, new mice	d-back-left	100	Original Session 1	runway
Bl6, new mice	d-back-right	100	Original Session 1	runway
Bl6, new mice	d-front-left	100	Original Session 1	runway
Bl6, new mice	d-front-right	100	Original Session 1	runway
Bl6, new mice	d-head	100	Original Session 1	runway
Bl6, new mice	d-tail-base	100	Original Session 1	runway
Bl6, new mice	l-back-ankle	97.63313609	Original Session 1	runway
Bl6, new mice	l-back-toe	92.89940828	Original Session 1	runway
Bl6, new mice	l-elbow	100	Original Session 1	runway
Bl6, new mice	l-front-toe-tip	98.25581395	Original Session 1	runway
Bl6, new mice	l-head	100	Original Session 1	runway
Bl6, new mice	l-hip	100	Original Session 1	runway
Bl6, new mice	l-iliac-crest	100	Original Session 1	runway
Bl6, new mice	l-shoulder	100	Original Session 1	runway
Bl6, new mice	l-tail-base	100	Original Session 1	runway
Bl6, new mice	l-wrist	100	Original Session 1	runway
Bl6, new mice	r-back-ankle	100	Original Session 1	runway
Bl6, new mice	r-back-toe	100	Original Session 1	runway
Bl6, new mice	r-elbow	100	Original Session 1	runway
Bl6, new mice	r-front-toe-tip	96.49122807	Original Session 1	runway

Bl6, new mice	r-head	100	Original Session 1	runway
Bl6, new mice	r-hip	100	Original Session 1	runway
Bl6, new mice	r-iliac-crest	100	Original Session 1	runway
Bl6, new mice	r-shoulder	100	Original Session 1	runway
Bl6, new mice	r-tail-base	100	Original Session 1	runway
Bl6, new mice	r-wrist	99.42196532	Original Session 1	runway
NOD, white fur	d-back-left	41.11111111	Original Session 1	runway
NOD, white fur	d-back-right	17.87709497	Original Session 1	runway
NOD, white fur	d-front-left	0	Original Session 1	runway
NOD, white fur	d-front-right	0	Original Session 1	runway
NOD, white fur	d-head	1.886792453	Original Session 1	runway
NOD, white fur	d-tail-base	0	Original Session 1	runway
NOD, white fur	l-back-ankle	0	Original Session 1	runway
NOD, white fur	l-back-toe	0	Original Session 1	runway
NOD, white fur	l-elbow	0	Original Session 1	runway
NOD, white fur	l-front-toe-tip	0	Original Session 1	runway
NOD, white fur	l-head	0	Original Session 1	runway
NOD, white fur	l-hip	0	Original Session 1	runway
NOD, white fur	l-iliac-crest	0	Original Session 1	runway
NOD, white fur	l-shoulder	0	Original Session 1	runway
NOD, white fur	l-tail-base	0	Original Session 1	runway
NOD, white fur	l-wrist	0	Original Session 1	runway
NOD, white fur	r-back-ankle	0	Original Session 1	runway
NOD, white fur	r-back-toe	0	Original Session 1	runway
NOD, white fur	r-elbow	0	Original Session 1	runway
NOD, white fur	r-front-toe-tip	0	Original Session 1	runway
NOD, white fur	r-head	0	Original Session 1	runway
NOD, white fur	r-hip	0	Original Session 1	runway
NOD, white fur	r-iliac-crest	0	Original Session 1	runway
NOD, white fur	r-shoulder	0	Original Session 1	runway
NOD, white fur	r-tail-base	0	Original Session 1	runway
NOD, white fur	r-wrist	0	Original Session 1	runway
NOD, white fur	d-back-left	99.25925926	Modified Session 2	runway
NOD, white fur	d-back-right	99.25925926	Modified Session 2	runway
NOD, white fur	d-front-left	99.23664122	Modified Session 2	runway
NOD, white fur	d-front-right	99.24242424	Modified Session 2	runway
NOD, white fur	d-head	100	Modified Session 2	runway
NOD, white fur	d-tail-base	98.48484848	Modified Session 2	runway
NOD, white fur	l-back-ankle	99.25925926	Modified Session 2	runway
NOD, white fur	l-back-toe	95.45454545	Modified Session 2	runway
NOD, white fur	l-elbow	95.45454545	Modified Session 2	runway
NOD, white fur	l-front-toe-tip	96.92307692	Modified Session 2	runway
NOD, white fur	l-head	100	Modified Session 2	runway
NOD, white fur	l-hip	100	Modified Session 2	runway
NOD, white fur	l-iliac-crest	99.23664122	Modified Session 2	runway
NOD, white fur	l-shoulder	98.4962406	Modified Session 2	runway
NOD, white fur	l-tail-base	100	Modified Session 2	runway
NOD, white fur	l-wrist	96.96969697	Modified Session 2	runway

NOD, white fur	r-back-ankle	96.2962963	Modified Session 2	runway
NOD, white fur	r-back-toe	96.26865672	Modified Session 2	runway
NOD, white fur	r-elbow	89.3129771	Modified Session 2	runway
NOD, white fur	r-front-toe-tip	83.07692308	Modified Session 2	runway
NOD, white fur	r-head	100	Modified Session 2	runway
NOD, white fur	r-hip	100	Modified Session 2	runway
NOD, white fur	r-iliac-crest	98.46153846	Modified Session 2	runway
NOD, white fur	r-shoulder	99.23664122	Modified Session 2	runway
NOD, white fur	r-tail-base	100	Modified Session 2	runway
NOD, white fur	r-wrist	93.89312977	Modified Session 2	runway

Suppl. Table 3: Summary of accuracy during runway

Group	Likelihood	Training
Bl6, original mic	99.28344848	Original Session 1
Bl6, new mice	99.41159814	Original Session 1
NOD, white fur	2.341346098	Original Session 1
NOD, white fur	97.45471542	Modified Session 2

Suppl Table 4: Accuracy of labeling different mice during runway test

Group	Parameter	Likelihood	Training Session	Setup
Bl6, original mice	d-back-left	96.969697	Original Session 1	ladder rung
Bl6, original mice	d-back-right	98.2758621	Original Session 1	ladder rung
Bl6, original mice	d-front-left	98.6998917	Original Session 1	ladder rung
Bl6, original mice	d-front-right	95.9821429	Original Session 1	ladder rung
Bl6, original mice	d-head	99.6760259	Original Session 1	ladder rung
Bl6, original mice	d-tail-base	99.3576017	Original Session 1	ladder rung
Bl6, original mice	l-back-ankle	94.5790081	Original Session 1	ladder rung
Bl6, original mice	l-back-toe	91.1235955	Original Session 1	ladder rung
Bl6, original mice	l-elbow	99.1351351	Original Session 1	ladder rung
Bl6, original mice	l-front-toe-tip	81.6826411	Original Session 1	ladder rung
Bl6, original mice	l-head	99.6499417	Original Session 1	ladder rung
Bl6, original mice	l-hip	100	Original Session 1	ladder rung
Bl6, original mice	l-iliac-crest	100	Original Session 1	ladder rung
Bl6, original mice	l-shoulder	100	Original Session 1	ladder rung
Bl6, original mice	l-tail-base	100	Original Session 1	ladder rung
Bl6, original mice	l-wrist	89.5744681	Original Session 1	ladder rung
Bl6, original mice	r-back-ankle	88.3886256	Original Session 1	ladder rung
Bl6, original mice	r-back-toe	90.0816803	Original Session 1	ladder rung
Bl6, original mice	r-elbow	95.3362256	Original Session 1	ladder rung
Bl6, original mice	r-front-toe-tip	89.9022801	Original Session 1	ladder rung
Bl6, original mice	r-head	100	Original Session 1	ladder rung
Bl6, original mice	r-hip	100	Original Session 1	ladder rung
Bl6, original mice	r-iliac-crest	100	Original Session 1	ladder rung
Bl6, original mice	r-shoulder	97.4566474	Original Session 1	ladder rung
Bl6, original mice	r-tail-base	100	Original Session 1	ladder rung
Bl6, original mice	r-wrist	95.2329361	Original Session 1	ladder rung
Bl6, original mice	d-back-left	100	Original Session 1	ladder rung
Bl6, new mice	d-back-right	95.6018519	Original Session 1	ladder rung
Bl6, new mice	d-front-left	97.752809	Original Session 1	ladder rung
Bl6, new mice	d-front-right	94.5454545	Original Session 1	ladder rung
Bl6, new mice	d-head	99.7722096	Original Session 1	ladder rung
Bl6, new mice	d-tail-base	98.3758701	Original Session 1	ladder rung
Bl6, new mice	l-back-ankle	87.5	Original Session 1	ladder rung
Bl6, new mice	l-back-toe	82.5287356	Original Session 1	ladder rung
Bl6, new mice	l-elbow	95.1388889	Original Session 1	ladder rung
Bl6, new mice	l-front-toe-tip	74.0909091	Original Session 1	ladder rung
Bl6, new mice	l-head	98.6238532	Original Session 1	ladder rung
Bl6, new mice	l-hip	100	Original Session 1	ladder rung
Bl6, new mice	l-iliac-crest	100	Original Session 1	ladder rung
Bl6, new mice	l-shoulder	98.5781991	Original Session 1	ladder rung
Bl6, new mice	l-tail-base	100	Original Session 1	ladder rung
Bl6, new mice	l-wrist	86.0411899	Original Session 1	ladder rung
Bl6, new mice	r-back-ankle	87.0748299	Original Session 1	ladder rung
Bl6, new mice	r-back-toe	85.778781	Original Session 1	ladder rung
Bl6, new mice	r-elbow	82.4884793	Original Session 1	ladder rung
Bl6, new mice	r-front-toe-tip	86.4367816	Original Session 1	ladder rung

Bl6, new mice	r-head	99.3055556	Original Session 1	ladder rung
Bl6, new mice	r-hip	99.3243243	Original Session 1	ladder rung
Bl6, new mice	r-iliac-crest	99.7727273	Original Session 1	ladder rung
Bl6, new mice	r-shoulder	76.443418	Original Session 1	ladder rung
Bl6, new mice	r-tail-base	100	Original Session 1	ladder rung
Bl6, new mice	r-wrist	92.9864253	Original Session 1	ladder rung
NOD, white fur	d-back-left	0	Original Session 1	ladder rung
NOD, white fur	d-back-right	0	Original Session 1	ladder rung
NOD, white fur	d-front-left	0	Original Session 1	ladder rung
NOD, white fur	d-front-right	0	Original Session 1	ladder rung
NOD, white fur	d-head	0	Original Session 1	ladder rung
NOD, white fur	d-tail-base	0	Original Session 1	ladder rung
NOD, white fur	l-back-ankle	0	Original Session 1	ladder rung
NOD, white fur	l-back-toe	0	Original Session 1	ladder rung
NOD, white fur	l-elbow	0	Original Session 1	ladder rung
NOD, white fur	l-front-toe-tip	0	Original Session 1	ladder rung
NOD, white fur	l-head	0	Original Session 1	ladder rung
NOD, white fur	l-hip	0	Original Session 1	ladder rung
NOD, white fur	l-iliac-crest	0	Original Session 1	ladder rung
NOD, white fur	l-shoulder	0	Original Session 1	ladder rung
NOD, white fur	l-tail-base	0	Original Session 1	ladder rung
NOD, white fur	l-wrist	0	Original Session 1	ladder rung
NOD, white fur	r-back-ankle	0	Original Session 1	ladder rung
NOD, white fur	r-back-toe	0	Original Session 1	ladder rung
NOD, white fur	r-elbow	0	Original Session 1	ladder rung
NOD, white fur	r-front-toe-tip	0	Original Session 1	ladder rung
NOD, white fur	r-head	0	Original Session 1	ladder rung
NOD, white fur	r-hip	0	Original Session 1	ladder rung
NOD, white fur	r-iliac-crest	0	Original Session 1	ladder rung
NOD, white fur	r-shoulder	0	Original Session 1	ladder rung
NOD, white fur	r-tail-base	0	Original Session 1	ladder rung
NOD, white fur	r-wrist	0	Original Session 1	ladder rung
NOD, white fur	d-back-left	95.959596	Modified Session 2	ladder rung
NOD, white fur	d-back-right	96.7576792	Modified Session 2	ladder rung
NOD, white fur	d-front-left	89.2307692	Modified Session 2	ladder rung
NOD, white fur	d-front-right	88.0749574	Modified Session 2	ladder rung
NOD, white fur	d-head	99.6705107	Modified Session 2	ladder rung
NOD, white fur	d-tail-base	82	Modified Session 2	ladder rung
NOD, white fur	l-back-ankle	94.5392491	Modified Session 2	ladder rung
NOD, white fur	l-back-toe	90.6354515	Modified Session 2	ladder rung
NOD, white fur	l-elbow	98.6394558	Modified Session 2	ladder rung
NOD, white fur	l-front-toe-tip	80.952381	Modified Session 2	ladder rung
NOD, white fur	l-head	99.6557659	Modified Session 2	ladder rung
NOD, white fur	l-hip	100	Modified Session 2	ladder rung
NOD, white fur	l-iliac-crest	99.323181	Modified Session 2	ladder rung
NOD, white fur	l-shoulder	99.6539792	Modified Session 2	ladder rung
NOD, white fur	l-tail-base	99.6557659	Modified Session 2	ladder rung
NOD, white fur	l-wrist	86.0306644	Modified Session 2	ladder rung

NOD, white fur	r-back-ankle	94.8892675	Modified Session 2	ladder rung
NOD, white fur	r-back-toe	93.2773109	Modified Session 2	ladder rung
NOD, white fur	r-elbow	93.814433	Modified Session 2	ladder rung
NOD, white fur	r-front-toe-tip	75	Modified Session 2	ladder rung
NOD, white fur	r-head	99.6533795	Modified Session 2	ladder rung
NOD, white fur	r-hip	99.6655518	Modified Session 2	ladder rung
NOD, white fur	r-iliac-crest	98.3305509	Modified Session 2	ladder rung
NOD, white fur	r-shoulder	94.6826758	Modified Session 2	ladder rung
NOD, white fur	r-tail-base	98.9813243	Modified Session 2	ladder rung
NOD, white fur	r-wrist	80.4123711	Modified Session 2	ladder rung

Suppl. Table 5: Summary of accuracy during rung test

Group	Likelihood	Training
Bl6, original mice	96.3372002	Original Session 1
Bl6, new mice	93.0062036	Original Session 1
NOD, white fur	0	Original Session 1
NOD, white fur	93.4417797	Modified Session 2

Suppl Table 6: Relative laser Doppler intensities 24h after stroke injury, baseline is 1

Mouse_ID	Side	Relative LDI
ID_1	L	0.96964282
ID_2	L	0.85884438
ID_3	L	0.94832747
ID_4	L	0.93208403
ID_5	L	1.07793734
ID_6	L	1.06103588
ID_7	L	0.91017473
ID_1	R	0.38120362
ID_2	R	0.17035167
ID_3	R	0.31565163
ID_4	R	0.25427492
ID_5	R	0.25176975
ID_6	R	0.54153461
ID_7	R	0.22838815
ID_1	baseline	1.04581865
ID_2	baseline	0.87407882
ID_3	baseline	0.87236997
ID_4	baseline	1.00907829
ID_5	baseline	0.95524939
ID_6	baseline	0.97361957
ID_7	baseline	1.03513831
ID_8	baseline	1.23464701

Suppl. Table 7: Summary of LDI

Side	LDI	SD
L	0.96543524	0.0791983
R	0.30616776	0.12333259
baseline	1	0.11546168

Suppl Table 8: Statistical evaluation of data

Group1	Group2	padj	Sig	Stat test
Left side	baseline	0.872246	ns	Tukey multiple comparison
right side	baseline	5.2007E-10	****	Tukey multiple comparison
right side	left side	4.0138E-09	****	Tukey multiple comparison

Suppl Table 9:Stroke volume on left and right hemisphere at 21 dpi

Mouse_ID	Side	Storke Volume in mm3
ID_1	L	0
ID_2	L	0
ID_3	L	0
ID_4	L	0
ID_5	L	0
ID_6	L	0
ID_7	L	0
ID_1	R	1.299355407
ID_2	R	1.089375951
ID_3	R	1.329701471
ID_4	R	1.056641243
ID_5	R	1.382891652
ID_6	R	1.621320592
ID_7	R	1.34263095

Suppl Table 10: Summary of stroke volume on left and right hemisphere at 21 dpi

Side	Storke Volume in mm3	SD
L	0	0
R	1.303131038	0.189883864

Suppl Table 11: Stroke areas in cortical sections 21 dpi

Mouse_ID	Side	A-P axis relative to bregma in mm	Stroke area in mm2
ID_1	L	-2.5	0
ID_2	L	-2.5	0
ID_3	L	-2.5	0
ID_4	L	-2.5	0
ID_5	L	-2.5	0
ID_6	L	-2.5	0
ID_7	L	-2.5	0
ID_1	R	-2.5	0
ID_2	R	-2.5	0
ID_3	R	-2.5	0
ID_4	R	-2.5	0
ID_5	R	-2.5	0
ID_6	R	-2.5	0
ID_7	R	-2.5	0
ID_1	L	-1.5	0
ID_2	L	-1.5	0
ID_3	L	-1.5	0
ID_4	L	-1.5	0
ID_5	L	-1.5	0
ID_6	L	-1.5	0
ID_7	L	-1.5	0
ID_1	R	-1.5	0
ID_2	R	-1.5	0.62052749
ID_3	R	-1.5	0.395874101

ID_4	R	-1.5	0.269451822
ID_5	R	-1.5	0
ID_6	R	-1.5	0.807621874
ID_7	R	-1.5	0.546754634
ID_1	L	-0.5	0
ID_2	L	-0.5	0
ID_3	L	-0.5	0
ID_4	L	-0.5	0
ID_5	L	-0.5	0
ID_6	L	-0.5	0
ID_7	L	-0.5	0
ID_1	R	-0.5	0.237142277
ID_2	R	-0.5	0.722405361
ID_3	R	-0.5	0.4974167
ID_4	R	-0.5	0.590926211
ID_5	R	-0.5	0.548070937
ID_6	R	-0.5	0.757378657
ID_7	R	-0.5	0.736623045
ID_1	L	0.5	0
ID_2	L	0.5	0
ID_3	L	0.5	0
ID_4	L	0.5	0
ID_5	L	0.5	0
ID_6	L	0.5	0
ID_7	L	0.5	0
ID_1	R	0.5	0.913724914
ID_2	R	0.5	0.473883078
ID_3	R	0.5	0.768881997
ID_4	R	0.5	0.72641693
ID_5	R	0.5	0.838932186
ID_6	R	0.5	0.578163799
ID_7	R	0.5	0.434000051
ID_1	L	1.5	0
ID_2	L	1.5	0
ID_3	L	1.5	0
ID_4	L	1.5	0
ID_5	L	1.5	0
ID_6	L	1.5	0
ID_7	L	1.5	0
ID_1	R	1.5	0.434094674
ID_2	R	1.5	0.257521549
ID_3	R	1.5	0.286860314
ID_4	R	1.5	0.047268964
ID_5	R	1.5	0.607549084
ID_6	R	1.5	0.288816558
ID_7	R	1.5	0.296568696
ID_1	L	2.5	0
ID_2	L	2.5	0

ID_3	L	2.5	0
ID_4	L	2.5	0
ID_5	L	2.5	0
ID_6	L	2.5	0
ID_7	L	2.5	0
ID_1	R	2.5	0
ID_2	R	2.5	0
ID_3	R	2.5	0
ID_4	R	2.5	0
ID_5	R	2.5	0
ID_6	R	2.5	0
ID_7	R	2.5	0

Suppl Table 12: Summary of stroke areas in cortical sections 21 dpi

Side	A-P axis relative to bregma	Stroke area in mm2	SD
L	-2.5	0	0
R	-2.5	0	0
L	-1.5	0	0
R	-1.5	0.377175703	0.308149882
L	-0.5	0	0
R	-0.5	0.584280455	0.18332815
L	0.5	0	0
R	0.5	0.676286136	0.18403602
L	1.5	0	0
R	1.5	0.316954263	0.171533564
L	2.5	0	0
R	2.5	0	0

Suppl. Table 13: Raw data bottom analysis

day	ID	Parameter	Value
baseline	ID_1	average duration	0.25833333
baseline	ID_2	average duration	0.54166667
baseline	ID_3	average duration	0.36666667
baseline	ID_4	average duration	0.425
baseline	ID_5	average duration	0.59166667
baseline	ID_6	average duration	0.61666667
baseline	ID_7	average duration	0.25
baseline	ID_8	average duration	0.40833333
baseline	ID_9	average duration	0.33333333
baseline	ID_10	average duration	0.23333333
baseline	ID_11	average duration	0.75
baseline	ID_12	average duration	0.38333333
baseline	ID_13	average duration	0.23333333
3days	ID_1	average duration	0.61666667
3days	ID_2	average duration	0.33333333
3days	ID_3	average duration	0.3
3days	ID_4	average duration	0.6
3days	ID_5	average duration	0.71666667
3days	ID_6	average duration	0.65
3days	ID_7	average duration	0.99166667
3days	ID_8	average duration	0.66666667
7days	ID_1	average duration	0.38333333
7days	ID_2	average duration	0.53333333
7days	ID_3	average duration	0.36666667
7days	ID_4	average duration	0.23333333
7days	ID_5	average duration	0.25
7days	ID_6	average duration	0.40833333
7days	ID_7	average duration	0.65833333
7days	ID_8	average duration	0.45
7days	ID_9	average duration	0.95
14days	ID_1	average duration	0.35
14days	ID_2	average duration	0.3
14days	ID_3	average duration	0.38333333
14days	ID_4	average duration	0.48333333
14days	ID_5	average duration	0.31666667
14days	ID_6	average duration	0.56666667
14days	ID_7	average duration	0.4
14days	ID_8	average duration	0.50833333
14days	ID_9	average duration	0.56666667
14days	ID_10	average duration	0.8
14days	ID_11	average duration	0.33333333
21days	ID_1	average duration	0.48333333
21days	ID_2	average duration	0.425
21days	ID_3	average duration	0.40833333
21days	ID_4	average duration	0.16666667
21days	ID_5	average duration	0.36666667
21days	ID_6	average duration	0.31666667
21days	ID_7	average duration	0.24166667
21days	ID_8	average duration	0.525
21days	ID_9	average duration	0.31666667
21days	ID_10	average duration	0.275
baseline	ID_1	average stance time	0.13333333
baseline	ID_2	average stance time	0.15
baseline	ID_3	average stance time	0.13333333

baseline	ID_4	average stance time	0.13333333
baseline	ID_5	average stance time	0.26666667
baseline	ID_6	average stance time	0.13333333
baseline	ID_7	average stance time	0.11666667
baseline	ID_8	average stance time	0.1
baseline	ID_9	average stance time	0.13333333
baseline	ID_10	average stance time	0.1
baseline	ID_11	average stance time	0.21666667
baseline	ID_12	average stance time	0.11666667
baseline	ID_13	average stance time	0.1
3days	ID_1	average stance time	0.28333333
3days	ID_2	average stance time	0.16666667
3days	ID_3	average stance time	0.15
3days	ID_4	average stance time	0.28333333
3days	ID_5	average stance time	0.26666667
3days	ID_6	average stance time	0.18333333
3days	ID_7	average stance time	0.2
3days	ID_8	average stance time	0.16666667
7days	ID_1	average stance time	0.15
7days	ID_2	average stance time	0.14166667
7days	ID_3	average stance time	0.15
7days	ID_4	average stance time	0.11666667
7days	ID_5	average stance time	0.1
7days	ID_6	average stance time	0.2
7days	ID_7	average stance time	0.28333333
7days	ID_8	average stance time	0.16666667
7days	ID_9	average stance time	0.19166667
14days	ID_1	average stance time	0.1
14days	ID_2	average stance time	0.13333333
14days	ID_3	average stance time	0.13333333
14days	ID_4	average stance time	0.16666667
14days	ID_5	average stance time	0.13333333
14days	ID_6	average stance time	0.3
14days	ID_7	average stance time	0.19166667
14days	ID_8	average stance time	0.18333333
14days	ID_9	average stance time	0.16666667
14days	ID_10	average stance time	0.16666667
14days	ID_11	average stance time	0.15
21days	ID_1	average stance time	0.18333333
21days	ID_2	average stance time	0.15
21days	ID_3	average stance time	0.2
21days	ID_4	average stance time	0.06666667
21days	ID_5	average stance time	0.18333333
21days	ID_6	average stance time	0.15833333
21days	ID_7	average stance time	0.11666667
21days	ID_8	average stance time	0.1
21days	ID_9	average stance time	0.15
21days	ID_10	average stance time	0.13333333
baseline	ID_1	average swing time	0.125
baseline	ID_2	average swing time	0.13333333
baseline	ID_3	average swing time	0.13333333
baseline	ID_4	average swing time	0.10833333
baseline	ID_5	average swing time	0.15
baseline	ID_6	average swing time	0.15
baseline	ID_7	average swing time	0.13333333
baseline	ID_8	average swing time	0.1

baseline	ID_9	average swing time	0.13333333
baseline	ID_10	average swing time	0.13333333
baseline	ID_11	average swing time	0.13333333
baseline	ID_12	average swing time	0.13333333
baseline	ID_13	average swing time	0.13333333
3days	ID_1	average swing time	0.13333333
3days	ID_2	average swing time	0.13333333
3days	ID_3	average swing time	0.13333333
3days	ID_4	average swing time	0.11666667
3days	ID_5	average swing time	0.13333333
3days	ID_6	average swing time	0.13333333
3days	ID_7	average swing time	0.15
3days	ID_8	average swing time	0.11666667
7days	ID_1	average swing time	0.11666667
7days	ID_2	average swing time	0.11666667
7days	ID_3	average swing time	0.11666667
7days	ID_4	average swing time	0.13333333
7days	ID_5	average swing time	0.11666667
7days	ID_6	average swing time	0.15833333
7days	ID_7	average swing time	0.15
7days	ID_8	average swing time	0.16666667
7days	ID_9	average swing time	0.13333333
14days	ID_1	average swing time	0.11666667
14days	ID_2	average swing time	0.13333333
14days	ID_3	average swing time	0.11666667
14days	ID_4	average swing time	0.13333333
14days	ID_5	average swing time	0.13333333
14days	ID_6	average swing time	0.13333333
14days	ID_7	average swing time	0.16666667
14days	ID_8	average swing time	0.16666667
14days	ID_9	average swing time	0.11666667
14days	ID_10	average swing time	0.15
14days	ID_11	average swing time	0.15
21days	ID_1	average swing time	0.11666667
21days	ID_2	average swing time	0.16666667
21days	ID_3	average swing time	0.15
21days	ID_4	average swing time	0.13333333
21days	ID_5	average swing time	0.15
21days	ID_6	average swing time	0.14166667
21days	ID_7	average swing time	0.15
21days	ID_8	average swing time	0.14166667
21days	ID_9	average swing time	0.15
21days	ID_10	average swing time	0.13333333
baseline	ID_1	left front right back synchronization	0.10416667
baseline	ID_2	left front right back synchronization	0.17931034
baseline	ID_3	left front right back synchronization	0.15789474
baseline	ID_4	left front right back synchronization	0.28030303
baseline	ID_5	left front right back synchronization	0.28497409
baseline	ID_6	left front right back synchronization	0.10434783
baseline	ID_7	left front right back synchronization	0.12844037
baseline	ID_8	left front right back synchronization	0.22834646
baseline	ID_9	left front right back synchronization	0.17213115
baseline	ID_10	left front right back synchronization	0.0990099
baseline	ID_11	left front right back synchronization	0.37820513
baseline	ID_12	left front right back synchronization	0.13114754
baseline	ID_13	left front right back synchronization	0.13684211

3days	ID_1	left front right back synchronization	0.33163265
3days	ID_2	left front right back synchronization	0.21126761
3days	ID_3	left front right back synchronization	0.18656716
3days	ID_4	left front right back synchronization	0.4
3days	ID_5	left front right back synchronization	0.36065574
3days	ID_6	left front right back synchronization	0.16564417
3days	ID_7	left front right back synchronization	0.30120482
3days	ID_8	left front right back synchronization	0.26
7days	ID_1	left front right back synchronization	0.1884058
7days	ID_2	left front right back synchronization	0.1986755
7days	ID_3	left front right back synchronization	0.28985507
7days	ID_4	left front right back synchronization	0.20535714
7days	ID_5	left front right back synchronization	0.14159292
7days	ID_6	left front right back synchronization	0.17037037
7days	ID_7	left front right back synchronization	0.34027778
7days	ID_8	left front right back synchronization	0.15503876
7days	ID_9	left front right back synchronization	0.27333333
14days	ID_1	left front right back synchronization	0.24576271
14days	ID_2	left front right back synchronization	0.15
14days	ID_3	left front right back synchronization	0.15909091
14days	ID_4	left front right back synchronization	0.19708029
14days	ID_5	left front right back synchronization	0.15315315
14days	ID_6	left front right back synchronization	0.27338129
14days	ID_7	left front right back synchronization	0.15
14days	ID_8	left front right back synchronization	0.16666667
14days	ID_9	left front right back synchronization	0.26206897
14days	ID_10	left front right back synchronization	0.18
14days	ID_11	left front right back synchronization	0.15
21days	ID_1	left front right back synchronization	0.26751592
21days	ID_2	left front right back synchronization	0.14655172
21days	ID_3	left front right back synchronization	0.21481481
21days	ID_4	left front right back synchronization	0.09589041
21days	ID_5	left front right back synchronization	0.17391304
21days	ID_6	left front right back synchronization	0.15151515
21days	ID_7	left front right back synchronization	0.09345794
21days	ID_8	left front right back synchronization	0.16666667
21days	ID_9	left front right back synchronization	0.1171875
21days	ID_10	left front right back synchronization	0.15044248
baseline	ID_1	right front left back synchronization	0.0625
baseline	ID_2	right front left back synchronization	0.14482759
baseline	ID_3	right front left back synchronization	0.09022556
baseline	ID_4	right front left back synchronization	0.28030303
baseline	ID_5	right front left back synchronization	0.37823834
baseline	ID_6	right front left back synchronization	0.16521739
baseline	ID_7	right front left back synchronization	0.18348624
baseline	ID_8	right front left back synchronization	0.2519685
baseline	ID_9	right front left back synchronization	0.16393443
baseline	ID_10	right front left back synchronization	0.07920792
baseline	ID_11	right front left back synchronization	0.17307692
baseline	ID_12	right front left back synchronization	0.14754098
baseline	ID_13	right front left back synchronization	0.09473684
3days	ID_1	right front left back synchronization	0.48979592
3days	ID_2	right front left back synchronization	0.26760563
3days	ID_3	right front left back synchronization	0.17910448
3days	ID_4	right front left back synchronization	0.48717949
3days	ID_5	right front left back synchronization	0.40983607

3days	ID_6	right front left back synchronization	0.32515337
3days	ID_7	right front left back synchronization	0.21084337
3days	ID_8	right front left back synchronization	0.23333333
7days	ID_1	right front left back synchronization	0.31884058
7days	ID_2	right front left back synchronization	0.18543046
7days	ID_3	right front left back synchronization	0.28985507
7days	ID_4	right front left back synchronization	0.16071429
7days	ID_5	right front left back synchronization	0.20353982
7days	ID_6	right front left back synchronization	0.2
7days	ID_7	right front left back synchronization	0.43055556
7days	ID_8	right front left back synchronization	0.10852713
7days	ID_9	right front left back synchronization	0.30666667
14days	ID_1	right front left back synchronization	0.23728814
14days	ID_2	right front left back synchronization	0.1625
14days	ID_3	right front left back synchronization	0.16666667
14days	ID_4	right front left back synchronization	0.24087591
14days	ID_5	right front left back synchronization	0.17117117
14days	ID_6	right front left back synchronization	0.41726619
14days	ID_7	right front left back synchronization	0.2
14days	ID_8	right front left back synchronization	0.20138889
14days	ID_9	right front left back synchronization	0.27586207
14days	ID_10	right front left back synchronization	0.26666667
14days	ID_11	right front left back synchronization	0.15
21days	ID_1	right front left back synchronization	0.29936306
21days	ID_2	right front left back synchronization	0.11206897
21days	ID_3	right front left back synchronization	0.27407407
21days	ID_4	right front left back synchronization	0.10958904
21days	ID_5	right front left back synchronization	0.23188406
21days	ID_6	right front left back synchronization	0.13636364
21days	ID_7	right front left back synchronization	0.09345794
21days	ID_8	right front left back synchronization	0.06862745
21days	ID_9	right front left back synchronization	0.1953125
21days	ID_10	right front left back synchronization	0.22123894

Suppl. Table 14: Summary of bottom analysis

day	Parameter	Mean	SD
baseline	average dur	0.41474359	0.16534734
3days	average dur	0.609375	0.21864652
7days	average dur	0.47037037	0.22267749
14days	average dur	0.45530303	0.14992001
21days	average dur	0.3525	0.11097561
baseline	average star	0.141025641	0.04840769
3days	average star	0.2125	0.05616727
7days	average star	0.166666667	0.05416667
14days	average star	0.165909091	0.05170087
21days	average star	0.144166667	0.04121047
baseline	average swi	0.130769231	0.01377087
3days	average swi	0.13125	0.01068117
7days	average swi	0.134259259	0.01973982
14days	average swi	0.137878788	0.01839521
21days	average swi	0.143333333	0.0134944
baseline	left front rig	0.183470719	0.0856535
3days	left front rig	0.277121519	0.08526072
7days	left front rig	0.218100741	0.06769096
14days	left front rig	0.189745818	0.04802373
21days	left front rig	0.157795566	0.05319644
baseline	right front l	0.170404904	0.08919253
3days	right front l	0.325356458	0.12344471
7days	right front l	0.244903286	0.09919638
14days	right front l	0.226335063	0.07660734
21days	right front l	0.174197967	0.08079999

Suppl. Table 15: Statistical test of bottom analysis

Measure	group1	group2	p.adj	sig	Test
average dura	baseline	3days	0.04648307	*	Repeated ANOVA, PH test*
average dura	baseline	7days	1	ns	Repeated ANOVA, PH test*
average dura	baseline	14days	1	ns	Repeated ANOVA, PH test*
average dura	baseline	21days	1	ns	Repeated ANOVA, PH test*
average stan	baseline	3days	0.03937093	*	Repeated ANOVA, PH test*
average stan	baseline	7days	0.77236619	ns	Repeated ANOVA, PH test*
average stan	baseline	14days	0.77236619	ns	Repeated ANOVA, PH test*
average stan	baseline	21days	0.89167767	ns	Repeated ANOVA, PH test*
duration_sd_	baseline	3days	0.94665102	ns	Repeated ANOVA, PH test*
duration_sd_	baseline	7days	1	ns	Repeated ANOVA, PH test*
duration_sd_	baseline	14days	1	ns	Repeated ANOVA, PH test*
duration_sd_	baseline	21days	1	ns	Repeated ANOVA, PH test*
LFRB synch	baseline	3days	0.02116338	*	Repeated ANOVA, PH test*
LFRB synch	baseline	7days	0.47641714	ns	Repeated ANOVA, PH test*
LFRB synch	baseline	14days	0.93621763	ns	Repeated ANOVA, PH test*
LFRB synch	baseline	21days	0.93621763	ns	Repeated ANOVA, PH test*
RFBL synch	baseline	3days	8.8073E-05	****	Repeated ANOVA, PH test*
RFBL synch	baseline	7days	0.0288736	*	Repeated ANOVA, PH test*
RFBL synch	baseline	14days	0.03061719	*	Repeated ANOVA, PH test*
RFBL synch	baseline	21days	0.80513565	ns	Repeated ANOVA, PH test*

* p.adjust.method = "holm", ref group = baseline (for more details see Methods)

Suppl. Table 16: Raw data bottom angle analysis

day	Measure	Angle, °	ID
0days	LB__avg_Angle	50.4077012	ID_1
0days	LB__avg_Angle	46.2632675	ID_2
0days	LB__avg_Angle	51.8477229	ID_3
0days	LB__avg_Angle	43.6508697	ID_4
0days	LB__avg_Angle	54.2814501	ID_5
0days	LB__avg_Angle	47.6089647	ID_6
0days	LB__avg_Angle	47.3941644	ID_7
0days	LB__avg_Angle	42.1677037	ID_8
0days	LB__avg_Angle	48.8915014	ID_9
0days	LB__avg_Angle	49.6482824	ID_10
0days	LB__avg_Angle	48.4205062	ID_11
0days	LB__avg_Angle	44.9161935	ID_12
0days	LB__avg_Angle	45.3047359	ID_13
3days	LB__avg_Angle	56.7093179	ID_1
3days	LB__avg_Angle	49.7875511	ID_2
3days	LB__avg_Angle	49.6046545	ID_3
3days	LB__avg_Angle	48.5468763	ID_4
3days	LB__avg_Angle	45.5417375	ID_5
3days	LB__avg_Angle	46.4329682	ID_6
3days	LB__avg_Angle	56.8399674	ID_7
3days	LB__avg_Angle	47.7674786	ID_8
7days	LB__avg_Angle	47.4811774	ID_1
7days	LB__avg_Angle	45.6543481	ID_2
7days	LB__avg_Angle	52.4828104	ID_3
7days	LB__avg_Angle	48.945709	ID_4
7days	LB__avg_Angle	50.8862455	ID_5
7days	LB__avg_Angle	51.4478357	ID_6
7days	LB__avg_Angle	48.9761677	ID_7
7days	LB__avg_Angle	50.5034691	ID_8
7days	LB__avg_Angle	55.529131	ID_9
14days	LB__avg_Angle	50.3122777	ID_1
14days	LB__avg_Angle	51.9261004	ID_2
14days	LB__avg_Angle	47.8045234	ID_3
14days	LB__avg_Angle	49.6406405	ID_4
14days	LB__avg_Angle	52.3945749	ID_5
14days	LB__avg_Angle	55.6305889	ID_6
14days	LB__avg_Angle	48.4529191	ID_7
14days	LB__avg_Angle	48.9886127	ID_8
14days	LB__avg_Angle	48.0132903	ID_9
14days	LB__avg_Angle	52.781057	ID_10
14days	LB__avg_Angle	55.0773354	ID_11
21days	LB__avg_Angle	47.672133	ID_1
21days	LB__avg_Angle	54.1935514	ID_2
21days	LB__avg_Angle	55.6359358	ID_3
21days	LB__avg_Angle	53.8303683	ID_4
21days	LB__avg_Angle	51.5771797	ID_5
21days	LB__avg_Angle	47.7049767	ID_6
21days	LB__avg_Angle	49.3323145	ID_7
21days	LB__avg_Angle	42.3500748	ID_8
21days	LB__avg_Angle	48.4011887	ID_9
21days	LB__avg_Angle	46.693582	ID_10
0days	LB__max	75.6027361	ID_1
0days	LB__max	81.4634481	ID_2
0days	LB__max	74.069999	ID_3

0days	LB__max	70.774883	ID_4
0days	LB__max	72.4356346	ID_5
0days	LB__max	69.9449045	ID_6
0days	LB__max	78.6149721	ID_7
0days	LB__max	78.9854971	ID_8
0days	LB__max	73.0850971	ID_9
0days	LB__max	75.4969288	ID_10
0days	LB__max	67.6386185	ID_11
0days	LB__max	76.7281341	ID_12
0days	LB__max	75.4352644	ID_13
3days	LB__max	71.3561627	ID_1
3days	LB__max	73.4782816	ID_2
3days	LB__max	73.5486086	ID_3
3days	LB__max	70.8213052	ID_4
3days	LB__max	79.0749323	ID_5
3days	LB__max	76.2572046	ID_6
3days	LB__max	82.0311242	ID_7
3days	LB__max	77.1708438	ID_8
7days	LB__max	72.7784758	ID_1
7days	LB__max	76.8154719	ID_2
7days	LB__max	71.4535317	ID_3
7days	LB__max	73.435902	ID_4
7days	LB__max	79.4444907	ID_5
7days	LB__max	72.6221119	ID_6
7days	LB__max	69.5756072	ID_7
7days	LB__max	71.4153506	ID_8
7days	LB__max	77.3774398	ID_9
14days	LB__max	74.9646969	ID_1
14days	LB__max	71.473176	ID_2
14days	LB__max	74.1434788	ID_3
14days	LB__max	70.0281556	ID_4
14days	LB__max	75.6343805	ID_5
14days	LB__max	69.3589935	ID_6
14days	LB__max	79.7671195	ID_7
14days	LB__max	80.5561463	ID_8
14days	LB__max	73.4745868	ID_9
14days	LB__max	74.9809401	ID_10
14days	LB__max	75.3733273	ID_11
21days	LB__max	77.1749442	ID_1
21days	LB__max	68.5800134	ID_2
21days	LB__max	72.8488419	ID_3
21days	LB__max	77.1250917	ID_4
21days	LB__max	77.724208	ID_5
21days	LB__max	78.6170218	ID_6
21days	LB__max	76.8056567	ID_7
21days	LB__max	75.4825825	ID_8
21days	LB__max	78.1885294	ID_9
21days	LB__max	73.484921	ID_10
0days	LB__min	18.779951	ID_1
0days	LB__min	12.1393046	ID_2
0days	LB__min	14.5346713	ID_3
0days	LB__min	9.86731573	ID_4
0days	LB__min	21.2852704	ID_5
0days	LB__min	17.7882452	ID_6
0days	LB__min	13.2839772	ID_7
0days	LB__min	11.1572571	ID_8

0days	LB__min	20.7572374	ID_9
0days	LB__min	11.512426	ID_10
0days	LB__min	16.4456199	ID_11
0days	LB__min	13.8128615	ID_12
0days	LB__min	18.9384396	ID_13
3days	LB__min	26.6972495	ID_1
3days	LB__min	21.1645706	ID_2
3days	LB__min	21.601163	ID_3
3days	LB__min	13.0184654	ID_4
3days	LB__min	11.2072338	ID_5
3days	LB__min	13.7395223	ID_6
3days	LB__min	19.7260928	ID_7
3days	LB__min	17.8103329	ID_8
7days	LB__min	15.6142006	ID_1
7days	LB__min	9.04592594	ID_2
7days	LB__min	13.3531695	ID_3
7days	LB__min	11.5769437	ID_4
7days	LB__min	16.9383934	ID_5
7days	LB__min	20.0917916	ID_6
7days	LB__min	13.6707086	ID_7
7days	LB__min	9.80482036	ID_8
7days	LB__min	24.0422522	ID_9
14days	LB__min	18.2062696	ID_1
14days	LB__min	20.739789	ID_2
14days	LB__min	12.5164845	ID_3
14days	LB__min	13.4960615	ID_4
14days	LB__min	16.2081826	ID_5
14days	LB__min	18.3561095	ID_6
14days	LB__min	13.0310458	ID_7
14days	LB__min	11.6624493	ID_8
14days	LB__min	18.5569438	ID_9
14days	LB__min	15.700712	ID_10
14days	LB__min	19.3793992	ID_11
21days	LB__min	12.8352746	ID_1
21days	LB__min	19.2433292	ID_2
21days	LB__min	17.5211671	ID_3
21days	LB__min	24.6634923	ID_4
21days	LB__min	13.3747976	ID_5
21days	LB__min	9.86454066	ID_6
21days	LB__min	18.4372497	ID_7
21days	LB__min	11.4983433	ID_8
21days	LB__min	12.5371903	ID_9
21days	LB__min	15.2239172	ID_10
0days	LF__avg_Angle	44.3766867	ID_1
0days	LF__avg_Angle	45.6786656	ID_2
0days	LF__avg_Angle	45.6025225	ID_3
0days	LF__avg_Angle	40.0442004	ID_4
0days	LF__avg_Angle	41.658499	ID_5
0days	LF__avg_Angle	45.8373681	ID_6
0days	LF__avg_Angle	40.9825227	ID_7
0days	LF__avg_Angle	46.3197746	ID_8
0days	LF__avg_Angle	39.7405324	ID_9
0days	LF__avg_Angle	41.446467	ID_10
0days	LF__avg_Angle	47.9112928	ID_11
0days	LF__avg_Angle	42.0872957	ID_12
0days	LF__avg_Angle	40.4297428	ID_13

3days	LF__avg_Angle	40.1017464	ID_1
3days	LF__avg_Angle	38.7049219	ID_2
3days	LF__avg_Angle	38.4627874	ID_3
3days	LF__avg_Angle	45.6049819	ID_4
3days	LF__avg_Angle	44.6651621	ID_5
3days	LF__avg_Angle	41.476749	ID_6
3days	LF__avg_Angle	41.566284	ID_7
3days	LF__avg_Angle	41.9276646	ID_8
7days	LF__avg_Angle	43.3831178	ID_1
7days	LF__avg_Angle	43.9012933	ID_2
7days	LF__avg_Angle	45.2450563	ID_3
7days	LF__avg_Angle	44.252576	ID_4
7days	LF__avg_Angle	47.1048525	ID_5
7days	LF__avg_Angle	38.8874691	ID_6
7days	LF__avg_Angle	43.4536674	ID_7
7days	LF__avg_Angle	41.652114	ID_8
7days	LF__avg_Angle	44.5867547	ID_9
14days	LF__avg_Angle	38.9225394	ID_1
14days	LF__avg_Angle	45.8809814	ID_2
14days	LF__avg_Angle	45.7993107	ID_3
14days	LF__avg_Angle	38.4470849	ID_4
14days	LF__avg_Angle	39.600663	ID_5
14days	LF__avg_Angle	38.4059727	ID_6
14days	LF__avg_Angle	37.2801076	ID_7
14days	LF__avg_Angle	37.5934035	ID_8
14days	LF__avg_Angle	39.6993253	ID_9
14days	LF__avg_Angle	43.2303069	ID_10
14days	LF__avg_Angle	39.7132891	ID_11
21days	LF__avg_Angle	41.9922658	ID_1
21days	LF__avg_Angle	38.1049868	ID_2
21days	LF__avg_Angle	38.8365056	ID_3
21days	LF__avg_Angle	44.9341325	ID_4
21days	LF__avg_Angle	45.5550475	ID_5
21days	LF__avg_Angle	41.8214176	ID_6
21days	LF__avg_Angle	42.9731244	ID_7
21days	LF__avg_Angle	43.8879138	ID_8
21days	LF__avg_Angle	40.3193466	ID_9
21days	LF__avg_Angle	45.9123759	ID_10
0days	LF__max	71.3555474	ID_1
0days	LF__max	73.6205435	ID_2
0days	LF__max	72.7194728	ID_3
0days	LF__max	73.9135913	ID_4
0days	LF__max	72.1734714	ID_5
0days	LF__max	76.8629446	ID_6
0days	LF__max	65.9668713	ID_7
0days	LF__max	71.6323368	ID_8
0days	LF__max	68.1159155	ID_9
0days	LF__max	63.0155705	ID_10
0days	LF__max	71.6518054	ID_11
0days	LF__max	66.2048668	ID_12
0days	LF__max	67.1179287	ID_13
3days	LF__max	62.8944716	ID_1
3days	LF__max	62.0243492	ID_2
3days	LF__max	64.4625047	ID_3
3days	LF__max	65.1996682	ID_4
3days	LF__max	63.0171567	ID_5

3days	LF__max	67.9348204	ID_6
3days	LF__max	69.0442159	ID_7
3days	LF__max	69.2306117	ID_8
7days	LF__max	74.7220877	ID_1
7days	LF__max	60.1052282	ID_2
7days	LF__max	73.5050624	ID_3
7days	LF__max	66.6297859	ID_4
7days	LF__max	70.7693029	ID_5
7days	LF__max	62.5451288	ID_6
7days	LF__max	72.0979674	ID_7
7days	LF__max	67.5696475	ID_8
7days	LF__max	61.6892277	ID_9
14days	LF__max	70.8544514	ID_1
14days	LF__max	69.2382287	ID_2
14days	LF__max	71.0093515	ID_3
14days	LF__max	59.1540156	ID_4
14days	LF__max	58.9617768	ID_5
14days	LF__max	65.8127948	ID_6
14days	LF__max	60.3439419	ID_7
14days	LF__max	58.0746393	ID_8
14days	LF__max	63.8947623	ID_9
14days	LF__max	62.8024434	ID_10
14days	LF__max	70.2428672	ID_11
21days	LF__max	67.897832	ID_1
21days	LF__max	68.5109692	ID_2
21days	LF__max	72.0326392	ID_3
21days	LF__max	70.721441	ID_4
21days	LF__max	67.4516664	ID_5
21days	LF__max	62.7378209	ID_6
21days	LF__max	67.4799931	ID_7
21days	LF__max	63.9467885	ID_8
21days	LF__max	59.830988	ID_9
21days	LF__max	73.2086895	ID_10
0days	LF__min	23.8666621	ID_1
0days	LF__min	22.012441	ID_2
0days	LF__min	23.2117812	ID_3
0days	LF__min	17.4185718	ID_4
0days	LF__min	13.0684365	ID_5
0days	LF__min	13.8849846	ID_6
0days	LF__min	17.3052319	ID_7
0days	LF__min	21.8392712	ID_8
0days	LF__min	17.7712817	ID_9
0days	LF__min	13.9475869	ID_10
0days	LF__min	20.8303449	ID_11
0days	LF__min	23.4446216	ID_12
0days	LF__min	22.143269	ID_13
3days	LF__min	20.0237983	ID_1
3days	LF__min	16.8376944	ID_2
3days	LF__min	19.4127383	ID_3
3days	LF__min	24.6163476	ID_4
3days	LF__min	27.4194864	ID_5
3days	LF__min	24.5957825	ID_6
3days	LF__min	19.5470476	ID_7
3days	LF__min	15.5388239	ID_8
7days	LF__min	20.5571831	ID_1
7days	LF__min	25.0332006	ID_2

7days	LF__min	22.8107086	ID_3
7days	LF__min	28.962962	ID_4
7days	LF__min	26.9066641	ID_5
7days	LF__min	15.8483888	ID_6
7days	LF__min	17.052464	ID_7
7days	LF__min	18.0987147	ID_8
7days	LF__min	13.8332881	ID_9
14days	LF__min	18.1671132	ID_1
14days	LF__min	21.0296377	ID_2
14days	LF__min	26.4345983	ID_3
14days	LF__min	23.4241709	ID_4
14days	LF__min	19.4164151	ID_5
14days	LF__min	17.8566158	ID_6
14days	LF__min	20.1419256	ID_7
14days	LF__min	22.1649327	ID_8
14days	LF__min	15.9152806	ID_9
14days	LF__min	19.8013228	ID_10
14days	LF__min	16.1697534	ID_11
21days	LF__min	22.1063674	ID_1
21days	LF__min	14.0338723	ID_2
21days	LF__min	15.5453273	ID_3
21days	LF__min	21.0632339	ID_4
21days	LF__min	18.8898574	ID_5
21days	LF__min	22.6806905	ID_6
21days	LF__min	20.0356047	ID_7
21days	LF__min	16.0254051	ID_8
21days	LF__min	22.2348411	ID_9
21days	LF__min	17.4894865	ID_10
0days	RB__avg_Angle	42.8575696	ID_1
0days	RB__avg_Angle	47.8907736	ID_2
0days	RB__avg_Angle	45.4616962	ID_3
0days	RB__avg_Angle	46.4126777	ID_4
0days	RB__avg_Angle	51.1492484	ID_5
0days	RB__avg_Angle	48.938546	ID_6
0days	RB__avg_Angle	50.3347242	ID_7
0days	RB__avg_Angle	42.5578142	ID_8
0days	RB__avg_Angle	45.3973929	ID_9
0days	RB__avg_Angle	48.2625869	ID_10
0days	RB__avg_Angle	49.9375309	ID_11
0days	RB__avg_Angle	47.5111561	ID_12
0days	RB__avg_Angle	46.988602	ID_13
3days	RB__avg_Angle	56.6963994	ID_1
3days	RB__avg_Angle	50.0101968	ID_2
3days	RB__avg_Angle	48.2409531	ID_3
3days	RB__avg_Angle	56.5917773	ID_4
3days	RB__avg_Angle	53.4249406	ID_5
3days	RB__avg_Angle	50.6791906	ID_6
3days	RB__avg_Angle	53.1195539	ID_7
3days	RB__avg_Angle	52.5745364	ID_8
7days	RB__avg_Angle	50.1109123	ID_1
7days	RB__avg_Angle	43.3631402	ID_2
7days	RB__avg_Angle	47.2184043	ID_3
7days	RB__avg_Angle	47.295858	ID_4
7days	RB__avg_Angle	47.4237984	ID_5
7days	RB__avg_Angle	49.2825584	ID_6
7days	RB__avg_Angle	45.6127664	ID_7

7days	RB__avg_Angle	45.5638926	ID_8
7days	RB__avg_Angle	57.9434523	ID_9
14days	RB__avg_Angle	52.7787434	ID_1
14days	RB__avg_Angle	44.1025601	ID_2
14days	RB__avg_Angle	44.1475928	ID_3
14days	RB__avg_Angle	46.4501452	ID_4
14days	RB__avg_Angle	51.0710783	ID_5
14days	RB__avg_Angle	54.6343834	ID_6
14days	RB__avg_Angle	52.0154762	ID_7
14days	RB__avg_Angle	52.7532014	ID_8
14days	RB__avg_Angle	54.9479005	ID_9
14days	RB__avg_Angle	47.1932394	ID_10
14days	RB__avg_Angle	43.3683346	ID_11
21days	RB__avg_Angle	46.5341358	ID_1
21days	RB__avg_Angle	49.6472984	ID_2
21days	RB__avg_Angle	53.0770775	ID_3
21days	RB__avg_Angle	47.5182862	ID_4
21days	RB__avg_Angle	51.0303299	ID_5
21days	RB__avg_Angle	51.8459975	ID_6
21days	RB__avg_Angle	47.550468	ID_7
21days	RB__avg_Angle	43.8839373	ID_8
21days	RB__avg_Angle	53.6451286	ID_9
21days	RB__avg_Angle	48.3182814	ID_10
0days	RB__max	79.2524699	ID_1
0days	RB__max	76.5545904	ID_2
0days	RB__max	76.2632394	ID_3
0days	RB__max	74.4156858	ID_4
0days	RB__max	69.7314665	ID_5
0days	RB__max	73.1861504	ID_6
0days	RB__max	78.4634387	ID_7
0days	RB__max	77.4835416	ID_8
0days	RB__max	74.4808528	ID_9
0days	RB__max	77.6515358	ID_10
0days	RB__max	70.9886417	ID_11
0days	RB__max	73.2560674	ID_12
0days	RB__max	74.6354537	ID_13
3days	RB__max	74.4457326	ID_1
3days	RB__max	73.6827252	ID_2
3days	RB__max	80.1357593	ID_3
3days	RB__max	73.6737137	ID_4
3days	RB__max	74.8518291	ID_5
3days	RB__max	76.7463005	ID_6
3days	RB__max	70.605494	ID_7
3days	RB__max	81.7661769	ID_8
7days	RB__max	78.607353	ID_1
7days	RB__max	73.582011	ID_2
7days	RB__max	76.0878981	ID_3
7days	RB__max	75.9765241	ID_4
7days	RB__max	77.4873907	ID_5
7days	RB__max	74.3065407	ID_6
7days	RB__max	67.1127163	ID_7
7days	RB__max	74.5862763	ID_8
7days	RB__max	72.2565461	ID_9
14days	RB__max	80.9040511	ID_1
14days	RB__max	71.0466587	ID_2
14days	RB__max	75.2850996	ID_3

14days	RB__max	79.4131704	ID_4
14days	RB__max	76.2390665	ID_5
14days	RB__max	72.1987127	ID_6
14days	RB__max	75.1817158	ID_7
14days	RB__max	75.8606938	ID_8
14days	RB__max	81.5700622	ID_9
14days	RB__max	71.4506763	ID_10
14days	RB__max	74.1709864	ID_11
21days	RB__max	77.0178975	ID_1
21days	RB__max	74.621803	ID_2
21days	RB__max	74.5669036	ID_3
21days	RB__max	69.2736063	ID_4
21days	RB__max	76.0697052	ID_5
21days	RB__max	76.5524895	ID_6
21days	RB__max	78.7967047	ID_7
21days	RB__max	69.5051639	ID_8
21days	RB__max	79.9013704	ID_9
21days	RB__max	70.4869638	ID_10
0days	RB__min	11.0013328	ID_1
0days	RB__min	16.3580449	ID_2
0days	RB__min	12.0878673	ID_3
0days	RB__min	14.0298275	ID_4
0days	RB__min	12.3896594	ID_5
0days	RB__min	15.1524297	ID_6
0days	RB__min	13.928863	ID_7
0days	RB__min	17.8776828	ID_8
0days	RB__min	8.49166957	ID_9
0days	RB__min	13.9296039	ID_10
0days	RB__min	16.3993358	ID_11
0days	RB__min	9.32792728	ID_12
0days	RB__min	15.0074391	ID_13
3days	RB__min	18.9303141	ID_1
3days	RB__min	8.110522	ID_2
3days	RB__min	14.7929504	ID_3
3days	RB__min	23.3820277	ID_4
3days	RB__min	21.5492284	ID_5
3days	RB__min	15.2845306	ID_6
3days	RB__min	11.8865389	ID_7
3days	RB__min	17.4785325	ID_8
7days	RB__min	10.9708971	ID_1
7days	RB__min	13.8859988	ID_2
7days	RB__min	14.3423761	ID_3
7days	RB__min	15.0188234	ID_4
7days	RB__min	13.8104608	ID_5
7days	RB__min	19.1767414	ID_6
7days	RB__min	10.7747456	ID_7
7days	RB__min	9.09689337	ID_8
7days	RB__min	20.9777243	ID_9
14days	RB__min	13.6894278	ID_1
14days	RB__min	19.3527546	ID_2
14days	RB__min	16.8424035	ID_3
14days	RB__min	13.6571717	ID_4
14days	RB__min	12.8456176	ID_5
14days	RB__min	18.6861882	ID_6
14days	RB__min	12.9638358	ID_7
14days	RB__min	13.1649191	ID_8

14days	RB__min	18.5242693	ID_9
14days	RB__min	16.5177571	ID_10
14days	RB__min	15.4198488	ID_11
21days	RB__min	13.8780983	ID_1
21days	RB__min	18.4699646	ID_2
21days	RB__min	18.3929991	ID_3
21days	RB__min	21.705148	ID_4
21days	RB__min	16.0258867	ID_5
21days	RB__min	19.026834	ID_6
21days	RB__min	15.4930936	ID_7
21days	RB__min	7.86374811	ID_8
21days	RB__min	12.7132641	ID_9
21days	RB__min	19.236803	ID_10
0days	RF__avg_Angle	42.5470983	ID_1
0days	RF__avg_Angle	42.0275756	ID_2
0days	RF__avg_Angle	44.9028249	ID_3
0days	RF__avg_Angle	47.1531512	ID_4
0days	RF__avg_Angle	44.5139313	ID_5
0days	RF__avg_Angle	38.5663288	ID_6
0days	RF__avg_Angle	41.2785736	ID_7
0days	RF__avg_Angle	41.1782994	ID_8
0days	RF__avg_Angle	40.1662729	ID_9
0days	RF__avg_Angle	39.1084113	ID_10
0days	RF__avg_Angle	44.0872109	ID_11
0days	RF__avg_Angle	38.1311052	ID_12
0days	RF__avg_Angle	38.6904266	ID_13
3days	RF__avg_Angle	41.245129	ID_1
3days	RF__avg_Angle	39.4679567	ID_2
3days	RF__avg_Angle	42.4764923	ID_3
3days	RF__avg_Angle	48.3882549	ID_4
3days	RF__avg_Angle	40.0920999	ID_5
3days	RF__avg_Angle	38.9129161	ID_6
3days	RF__avg_Angle	34.324454	ID_7
3days	RF__avg_Angle	37.616126	ID_8
7days	RF__avg_Angle	42.4987069	ID_1
7days	RF__avg_Angle	42.3441563	ID_2
7days	RF__avg_Angle	43.7749879	ID_3
7days	RF__avg_Angle	43.2740301	ID_4
7days	RF__avg_Angle	43.4596765	ID_5
7days	RF__avg_Angle	40.4807501	ID_6
7days	RF__avg_Angle	41.7278437	ID_7
7days	RF__avg_Angle	43.4188312	ID_8
7days	RF__avg_Angle	44.9213287	ID_9
14days	RF__avg_Angle	44.0113542	ID_1
14days	RF__avg_Angle	46.7872042	ID_2
14days	RF__avg_Angle	39.700803	ID_3
14days	RF__avg_Angle	43.9078137	ID_4
14days	RF__avg_Angle	46.5437653	ID_5
14days	RF__avg_Angle	42.4092405	ID_6
14days	RF__avg_Angle	40.5157696	ID_7
14days	RF__avg_Angle	42.6896769	ID_8
14days	RF__avg_Angle	36.7752737	ID_9
14days	RF__avg_Angle	43.9457101	ID_10
14days	RF__avg_Angle	37.4580785	ID_11
21days	RF__avg_Angle	42.4759573	ID_1
21days	RF__avg_Angle	44.5900231	ID_2

21days	RF__avg_Angle	47.919704	ID_3
21days	RF__avg_Angle	41.8289005	ID_4
21days	RF__avg_Angle	40.3849643	ID_5
21days	RF__avg_Angle	42.0178862	ID_6
21days	RF__avg_Angle	44.1430753	ID_7
21days	RF__avg_Angle	39.145023	ID_8
21days	RF__avg_Angle	41.2880727	ID_9
21days	RF__avg_Angle	45.7266488	ID_10
0days	RF__max	65.1650482	ID_1
0days	RF__max	63.5633778	ID_2
0days	RF__max	66.2751629	ID_3
0days	RF__max	63.3982971	ID_4
0days	RF__max	68.1431847	ID_5
0days	RF__max	63.1142133	ID_6
0days	RF__max	68.3333809	ID_7
0days	RF__max	65.4583984	ID_8
0days	RF__max	66.4922866	ID_9
0days	RF__max	63.9437223	ID_10
0days	RF__max	65.649095	ID_11
0days	RF__max	61.7136828	ID_12
0days	RF__max	64.8635144	ID_13
3days	RF__max	58.1757834	ID_1
3days	RF__max	57.18388	ID_2
3days	RF__max	61.873653	ID_3
3days	RF__max	60.3414347	ID_4
3days	RF__max	64.3680365	ID_5
3days	RF__max	62.9111936	ID_6
3days	RF__max	69.0835872	ID_7
3days	RF__max	66.1051816	ID_8
7days	RF__max	64.2548686	ID_1
7days	RF__max	66.1595638	ID_2
7days	RF__max	67.2837534	ID_3
7days	RF__max	66.3447234	ID_4
7days	RF__max	66.70868	ID_5
7days	RF__max	64.8272284	ID_6
7days	RF__max	65.2065834	ID_7
7days	RF__max	65.6265089	ID_8
7days	RF__max	81.2978772	ID_9
14days	RF__max	65.7958842	ID_1
14days	RF__max	65.0531998	ID_2
14days	RF__max	68.1754157	ID_3
14days	RF__max	71.5300217	ID_4
14days	RF__max	68.488436	ID_5
14days	RF__max	61.4620513	ID_6
14days	RF__max	64.3952247	ID_7
14days	RF__max	72.7651226	ID_8
14days	RF__max	65.5283881	ID_9
14days	RF__max	74.306757	ID_10
14days	RF__max	65.7601374	ID_11
21days	RF__max	63.3070223	ID_1
21days	RF__max	65.2948342	ID_2
21days	RF__max	72.7203099	ID_3
21days	RF__max	68.5884133	ID_4
21days	RF__max	61.235668	ID_5
21days	RF__max	63.9349587	ID_6
21days	RF__max	66.5295257	ID_7

21days	RF__max	68.8908511	ID_8
21days	RF__max	64.5858355	ID_9
21days	RF__max	65.0675158	ID_10
0days	RF__min	24.6319339	ID_1
0days	RF__min	14.7688315	ID_2
0days	RF__min	18.0309036	ID_3
0days	RF__min	21.744939	ID_4
0days	RF__min	19.2623516	ID_5
0days	RF__min	16.7684322	ID_6
0days	RF__min	21.5467565	ID_7
0days	RF__min	17.1549487	ID_8
0days	RF__min	18.826316	ID_9
0days	RF__min	23.5426925	ID_10
0days	RF__min	22.3413851	ID_11
0days	RF__min	22.3218924	ID_12
0days	RF__min	21.0932269	ID_13
3days	RF__min	23.8001133	ID_1
3days	RF__min	21.5756968	ID_2
3days	RF__min	22.6118278	ID_3
3days	RF__min	24.4239223	ID_4
3days	RF__min	17.9108196	ID_5
3days	RF__min	22.6727528	ID_6
3days	RF__min	13.8024176	ID_7
3days	RF__min	17.578919	ID_8
7days	RF__min	22.6544294	ID_1
7days	RF__min	18.8966195	ID_2
7days	RF__min	18.9814378	ID_3
7days	RF__min	17.7187437	ID_4
7days	RF__min	20.6005688	ID_5
7days	RF__min	13.0939727	ID_6
7days	RF__min	23.2448505	ID_7
7days	RF__min	19.0907271	ID_8
7days	RF__min	19.7519316	ID_9
14days	RF__min	27.0984077	ID_1
14days	RF__min	23.841235	ID_2
14days	RF__min	12.7051403	ID_3
14days	RF__min	14.6091791	ID_4
14days	RF__min	16.563378	ID_5
14days	RF__min	14.4490435	ID_6
14days	RF__min	17.6629117	ID_7
14days	RF__min	18.5965583	ID_8
14days	RF__min	15.3036826	ID_9
14days	RF__min	12.5284398	ID_10
14days	RF__min	20.3577251	ID_11
21days	RF__min	24.6862726	ID_1
21days	RF__min	19.9209133	ID_2
21days	RF__min	22.1483525	ID_3
21days	RF__min	16.1469054	ID_4
21days	RF__min	21.1234341	ID_5
21days	RF__min	17.4537046	ID_6
21days	RF__min	21.6309296	ID_7
21days	RF__min	13.7761458	ID_8
21days	RF__min	17.7483715	ID_9
21days	RF__min	24.29959	ID_10

Suppl. Table 17: Summary of bottom angle analysis

day	Measure	Mean	SD
0days	LB__avg_Angle	47.75408182	3.3690005
3days	LB__avg_Angle	50.15381894	4.33437741
7days	LB__avg_Angle	50.21187711	2.8908405
14days	LB__avg_Angle	51.00199275	2.75730422
21days	LB__avg_Angle	49.7391305	4.06844479
0days	LB__max	74.63662441	3.88910043
3days	LB__max	75.46730787	3.88677426
7days	LB__max	73.87982019	3.265263
14days	LB__max	74.52318194	3.51678681
21days	LB__max	75.60318105	3.12537027
0days	LB__min	15.40789053	3.83166016
3days	LB__min	18.12057878	5.21855366
7days	LB__min	14.90424509	4.87824285
14days	LB__min	16.16849516	3.10961442
21days	LB__min	15.5199302	4.45887447
0days	LF__avg_Angle	43.23965924	2.78945572
3days	LF__avg_Angle	41.56378715	2.56394347
7days	LF__avg_Angle	43.60743345	2.30625999
14days	LF__avg_Angle	40.41572586	3.10462092
21days	LF__avg_Angle	42.43371163	2.73292041
0days	LF__max	70.334682	3.92349668
3days	LF__max	65.47597481	2.89340866
7days	LF__max	67.73704872	5.4002759
14days	LF__max	64.580843	5.10775037
21days	LF__max	67.38188278	4.19884365
0days	LF__min	19.28803726	3.91814991
3days	LF__min	20.99896488	4.13953225
7days	LF__min	21.01150822	5.24331798
14days	LF__min	20.04743327	3.13943973
21days	LF__min	19.0104686	3.10427479
0days	RB__avg_Angle	47.20771682	2.66120631
3days	RB__avg_Angle	52.66719351	3.00065815
7days	RB__avg_Angle	48.20164253	4.17102957
14days	RB__avg_Angle	49.40569593	4.42802183
21days	RB__avg_Angle	49.30509406	3.10780087
0days	RB__max	75.10485648	2.85876929
3days	RB__max	75.7384664	3.66409559
7days	RB__max	74.44480626	3.37197267
14days	RB__max	75.75644489	3.61049547
21days	RB__max	74.6792608	3.78181284
0days	RB__min	13.53705253	2.78885881
3days	RB__min	16.42683058	5.01051585
7days	RB__min	14.22829565	3.87040931
14days	RB__min	15.60583578	2.49934934
21days	RB__min	16.28058394	4.01370633
0days	RF__avg_Angle	41.71932383	2.822009
3days	RF__avg_Angle	40.31542859	4.08163483
7days	RF__avg_Angle	42.87781238	1.28772978
14days	RF__avg_Angle	42.24951723	3.32277835
21days	RF__avg_Angle	42.95202551	2.63924393
0days	RF__max	65.08564342	1.94780001
3days	RF__max	62.50534375	4.00019755
7days	RF__max	67.52330968	5.25149054
14days	RF__max	67.56914894	3.91888757

21days	RF__max	66.01549344	3.30116977
0days	RF__min	20.15650845	2.92822757
3days	RF__min	20.54705866	3.71679916
7days	RF__min	19.33703122	2.95847428
14days	RF__min	17.6105183	4.61167268
21days	RF__min	19.89346194	3.5592443

Suppl. Table 18: Statistical test of bottom angle analysis

Measure	group1	group2	p.adj	sig	Test*
LB__avg_Ang	0days	3days	0.35602904	ns	Repeat ANOVA + PH test
LB__avg_Ang	0days	7days	0.35602904	ns	Repeat ANOVA + PH test
LB__avg_Ang	0days	14days	0.1169572	ns	Repeat ANOVA + PH test
LB__avg_Ang	0days	21days	0.35602904	ns	Repeat ANOVA + PH test
LB__max2	0days	3days	1	ns	Repeat ANOVA + PH test
LB__max2	0days	7days	1	ns	Repeat ANOVA + PH test
LB__max2	0days	14days	1	ns	Repeat ANOVA + PH test
LB__max2	0days	21days	1	ns	Repeat ANOVA + PH test
LB__min2	0days	3days	0.3863356	ns	Repeat ANOVA + PH test
LB__min2	0days	7days	1	ns	Repeat ANOVA + PH test
LB__min2	0days	14days	1	ns	Repeat ANOVA + PH test
LB__min2	0days	21days	1	ns	Repeat ANOVA + PH test
LF__avg_Ang	0days	3days	0.91277765	ns	Repeat ANOVA + PH test
LF__avg_Ang	0days	7days	1	ns	Repeat ANOVA + PH test
LF__avg_Ang	0days	14days	0.23150504	ns	Repeat ANOVA + PH test
LF__avg_Ang	0days	21days	1	ns	Repeat ANOVA + PH test
LF__max2	0days	3days	0.00899179	**	Repeat ANOVA + PH test
LF__max2	0days	7days	0.10685635	ns	Repeat ANOVA + PH test
LF__max2	0days	14days	0.00048251	***	Repeat ANOVA + PH test
LF__max2	0days	21days	0.10685635	ns	Repeat ANOVA + PH test
LF__min2	0days	3days	1	ns	Repeat ANOVA + PH test
LF__min2	0days	7days	1	ns	Repeat ANOVA + PH test
LF__min2	0days	14days	1	ns	Repeat ANOVA + PH test
LF__min2	0days	21days	1	ns	Repeat ANOVA + PH test
RB__avg_Ang	0days	3days	0.0034547	**	Repeat ANOVA + PH test
RB__avg_Ang	0days	7days	0.52765777	ns	Repeat ANOVA + PH test
RB__avg_Ang	0days	14days	0.41888756	ns	Repeat ANOVA + PH test
RB__avg_Ang	0days	21days	0.41888756	ns	Repeat ANOVA + PH test
RB__max2	0days	3days	1	ns	Repeat ANOVA + PH test
RB__max2	0days	7days	1	ns	Repeat ANOVA + PH test
RB__max2	0days	14days	1	ns	Repeat ANOVA + PH test
RB__max2	0days	21days	1	ns	Repeat ANOVA + PH test
RB__min2	0days	3days	0.29063093	ns	Repeat ANOVA + PH test
RB__min2	0days	7days	0.6604527	ns	Repeat ANOVA + PH test
RB__min2	0days	14days	0.32875256	ns	Repeat ANOVA + PH test
RB__min2	0days	21days	0.29063093	ns	Repeat ANOVA + PH test
RF__avg_Ang	0days	3days	1	ns	Repeat ANOVA + PH test
RF__avg_Ang	0days	7days	1	ns	Repeat ANOVA + PH test
RF__avg_Ang	0days	14days	1	ns	Repeat ANOVA + PH test
RF__avg_Ang	0days	21days	1	ns	Repeat ANOVA + PH test
RF__max2	0days	3days	0.38078015	ns	Repeat ANOVA + PH test
RF__max2	0days	7days	0.38078015	ns	Repeat ANOVA + PH test
RF__max2	0days	14days	0.38078015	ns	Repeat ANOVA + PH test
RF__max2	0days	21days	0.5424264	ns	Repeat ANOVA + PH test
RF__min2	0days	3days	1	ns	Repeat ANOVA + PH test
RF__min2	0days	7days	1	ns	Repeat ANOVA + PH test
RF__min2	0days	14days	0.34870829	ns	Repeat ANOVA + PH test
RF__min2	0days	21days	1	ns	Repeat ANOVA + PH test

Suppl. Table 19: Raw data side perspective height analysis

day	Measure	Value	ID
0days	l-back-ankle__Average_Heig	-0.558372	ID_1
0days	l-back-ankle__Average_Heig	-0.0928311	ID_2
0days	l-back-ankle__Average_Heig	-0.0743401	ID_3
0days	l-back-ankle__Average_Heig	0	ID_4
0days	l-back-ankle__Average_Heig	1.04525235	ID_5
0days	l-back-ankle__Average_Heig	0.46756773	ID_6
0days	l-back-ankle__Average_Heig	-0.306591	ID_7
0days	l-back-ankle__Average_Heig	-0.8661789	ID_8
0days	l-back-ankle__Average_Heig	0.49640185	ID_9
0days	l-back-ankle__Average_Heig	-0.5475319	ID_10
0days	l-back-ankle__Average_Heig	1.5837189	ID_11
0days	l-back-ankle__Average_Heig	0.12513895	ID_12
0days	l-back-ankle__Average_Heig	0.04543175	ID_13
3days	l-back-ankle__Average_Heig	1.12716089	ID_1
3days	l-back-ankle__Average_Heig	0.01360356	ID_2
3days	l-back-ankle__Average_Heig	0.17252705	ID_3
3days	l-back-ankle__Average_Heig	0.0378154	ID_4
3days	l-back-ankle__Average_Heig	-0.5515274	ID_5
3days	l-back-ankle__Average_Heig	-0.614162	ID_6
3days	l-back-ankle__Average_Heig	0.68601229	ID_7
3days	l-back-ankle__Average_Heig	-0.1506365	ID_8
7days	l-back-ankle__Average_Heig	-0.5815902	ID_1
7days	l-back-ankle__Average_Heig	-0.6634506	ID_2
7days	l-back-ankle__Average_Heig	-0.4829293	ID_3
7days	l-back-ankle__Average_Heig	-0.3621267	ID_4
7days	l-back-ankle__Average_Heig	-0.1706575	ID_5
7days	l-back-ankle__Average_Heig	0.13531241	ID_6
7days	l-back-ankle__Average_Heig	0.49527216	ID_7
7days	l-back-ankle__Average_Heig	-0.351378	ID_8
7days	l-back-ankle__Average_Heig	0.06333767	ID_9
14days	l-back-ankle__Average_Heig	-0.076288	ID_1
14days	l-back-ankle__Average_Heig	0.15133667	ID_2
14days	l-back-ankle__Average_Heig	-0.1810717	ID_3
14days	l-back-ankle__Average_Heig	0.15781205	ID_4
14days	l-back-ankle__Average_Heig	0.0320646	ID_5
14days	l-back-ankle__Average_Heig	1.71953848	ID_6
14days	l-back-ankle__Average_Heig	-0.032379	ID_7
14days	l-back-ankle__Average_Heig	0.19315873	ID_8
14days	l-back-ankle__Average_Heig	-0.499656	ID_9
14days	l-back-ankle__Average_Heig	0.50124279	ID_10
14days	l-back-ankle__Average_Heig	-0.5566281	ID_11
21days	l-back-ankle__Average_Heig	-0.0485212	ID_1
21days	l-back-ankle__Average_Heig	1.00486578	ID_2
21days	l-back-ankle__Average_Heig	1.55559221	ID_3
21days	l-back-ankle__Average_Heig	-0.1152101	ID_4
21days	l-back-ankle__Average_Heig	-0.345291	ID_5
21days	l-back-ankle__Average_Heig	-0.527633	ID_6
21days	l-back-ankle__Average_Heig	-0.9426903	ID_7
21days	l-back-ankle__Average_Heig	-0.4602843	ID_8
21days	l-back-ankle__Average_Heig	-0.7781565	ID_9
21days	l-back-ankle__Average_Heig	0.49855778	ID_10
0days	l-back-ankle__Movement	-0.9914844	ID_1
0days	l-back-ankle__Movement	0.02306694	ID_2
0days	l-back-ankle__Movement	-0.2829376	ID_3

0days	l-back-ankle__Movement	0.74780835	ID_4
0days	l-back-ankle__Movement	1.85085199	ID_5
0days	l-back-ankle__Movement	2.32714528	ID_6
0days	l-back-ankle__Movement	-0.681279	ID_7
0days	l-back-ankle__Movement	-2.3847816	ID_8
0days	l-back-ankle__Movement	1.82626031	ID_9
0days	l-back-ankle__Movement	-1.0159964	ID_10
0days	l-back-ankle__Movement	1.79036629	ID_11
0days	l-back-ankle__Movement	0	ID_12
0days	l-back-ankle__Movement	-0.1161506	ID_13
3days	l-back-ankle__Movement	0.21187297	ID_1
3days	l-back-ankle__Movement	0.2124552	ID_2
3days	l-back-ankle__Movement	0.27917367	ID_3
3days	l-back-ankle__Movement	-1.2343567	ID_4
3days	l-back-ankle__Movement	-2.0757384	ID_5
3days	l-back-ankle__Movement	-1.9038635	ID_6
3days	l-back-ankle__Movement	1.32226482	ID_7
3days	l-back-ankle__Movement	-0.1723685	ID_8
7days	l-back-ankle__Movement	-0.9425015	ID_1
7days	l-back-ankle__Movement	-0.9437131	ID_2
7days	l-back-ankle__Movement	-0.8073341	ID_3
7days	l-back-ankle__Movement	-0.6768367	ID_4
7days	l-back-ankle__Movement	-0.6315821	ID_5
7days	l-back-ankle__Movement	0.10237663	ID_6
7days	l-back-ankle__Movement	0.62162624	ID_7
7days	l-back-ankle__Movement	-0.1545461	ID_8
7days	l-back-ankle__Movement	-0.0778493	ID_9
14days	l-back-ankle__Movement	1.28366887	ID_1
14days	l-back-ankle__Movement	0.20422954	ID_2
14days	l-back-ankle__Movement	-0.2875744	ID_3
14days	l-back-ankle__Movement	0.37955496	ID_4
14days	l-back-ankle__Movement	0.60992437	ID_5
14days	l-back-ankle__Movement	2.32838002	ID_6
14days	l-back-ankle__Movement	0.2006356	ID_7
14days	l-back-ankle__Movement	0.27062048	ID_8
14days	l-back-ankle__Movement	-0.9489289	ID_9
14days	l-back-ankle__Movement	0.48324093	ID_10
14days	l-back-ankle__Movement	-0.0334763	ID_11
21days	l-back-ankle__Movement	-1.1024072	ID_1
21days	l-back-ankle__Movement	0.82531936	ID_2
21days	l-back-ankle__Movement	1.64836129	ID_3
21days	l-back-ankle__Movement	-0.6164052	ID_4
21days	l-back-ankle__Movement	-0.8705098	ID_5
21days	l-back-ankle__Movement	-1.5564573	ID_6
21days	l-back-ankle__Movement	-2.2947539	ID_7
21days	l-back-ankle__Movement	-0.1685381	ID_8
21days	l-back-ankle__Movement	-1.5300792	ID_9
21days	l-back-ankle__Movement	0.1785303	ID_10
0days	l-back-toe__Average_Height	-0.2007638	ID_1
0days	l-back-toe__Average_Height	-0.0731845	ID_2
0days	l-back-toe__Average_Height	-0.1845622	ID_3
0days	l-back-toe__Average_Height	0.1274839	ID_4
0days	l-back-toe__Average_Height	-0.3949807	ID_5
0days	l-back-toe__Average_Height	0.14552974	ID_6
0days	l-back-toe__Average_Height	0.25121786	ID_7
0days	l-back-toe__Average_Height	-0.5706094	ID_8

0days	l-back-toe__Average_Height	-0.2047977	ID_9
0days	l-back-toe__Average_Height	0	ID_10
0days	l-back-toe__Average_Height	0.27136664	ID_11
0days	l-back-toe__Average_Height	0.50930984	ID_12
0days	l-back-toe__Average_Height	0.33806656	ID_13
3days	l-back-toe__Average_Height	-0.1667614	ID_1
3days	l-back-toe__Average_Height	-0.2929109	ID_2
3days	l-back-toe__Average_Height	-0.2101754	ID_3
3days	l-back-toe__Average_Height	-0.3827026	ID_4
3days	l-back-toe__Average_Height	-0.3570359	ID_5
3days	l-back-toe__Average_Height	-0.1997423	ID_6
3days	l-back-toe__Average_Height	-0.4044975	ID_7
3days	l-back-toe__Average_Height	-0.1764409	ID_8
7days	l-back-toe__Average_Height	-0.3629967	ID_1
7days	l-back-toe__Average_Height	-0.3695886	ID_2
7days	l-back-toe__Average_Height	-0.149925	ID_3
7days	l-back-toe__Average_Height	-0.2441127	ID_4
7days	l-back-toe__Average_Height	-0.1580983	ID_5
7days	l-back-toe__Average_Height	-0.0772535	ID_6
7days	l-back-toe__Average_Height	-0.2933143	ID_7
7days	l-back-toe__Average_Height	-0.1037771	ID_8
7days	l-back-toe__Average_Height	-0.18197	ID_9
14days	l-back-toe__Average_Height	-0.1052261	ID_1
14days	l-back-toe__Average_Height	-0.0422381	ID_2
14days	l-back-toe__Average_Height	-0.2627759	ID_3
14days	l-back-toe__Average_Height	-0.2959257	ID_4
14days	l-back-toe__Average_Height	-0.4000789	ID_5
14days	l-back-toe__Average_Height	-0.2026806	ID_6
14days	l-back-toe__Average_Height	-0.1521897	ID_7
14days	l-back-toe__Average_Height	-0.0435699	ID_8
14days	l-back-toe__Average_Height	-0.2636383	ID_9
14days	l-back-toe__Average_Height	-0.1224169	ID_10
14days	l-back-toe__Average_Height	0.21770285	ID_11
21days	l-back-toe__Average_Height	-0.0608787	ID_1
21days	l-back-toe__Average_Height	0.27231807	ID_2
21days	l-back-toe__Average_Height	-0.0697866	ID_3
21days	l-back-toe__Average_Height	-0.0390446	ID_4
21days	l-back-toe__Average_Height	-0.0497526	ID_5
21days	l-back-toe__Average_Height	-0.1145241	ID_6
21days	l-back-toe__Average_Height	-0.1393812	ID_7
21days	l-back-toe__Average_Height	-0.1410475	ID_8
21days	l-back-toe__Average_Height	0.13321495	ID_9
21days	l-back-toe__Average_Height	0.31476302	ID_10
0days	l-back-toe__Movement	-0.891783	ID_1
0days	l-back-toe__Movement	-0.912764	ID_2
0days	l-back-toe__Movement	-0.6658001	ID_3
0days	l-back-toe__Movement	1.48389972	ID_4
0days	l-back-toe__Movement	1.2754539	ID_5
0days	l-back-toe__Movement	0	ID_6
0days	l-back-toe__Movement	0.19085782	ID_7
0days	l-back-toe__Movement	-2.7356388	ID_8
0days	l-back-toe__Movement	-0.6101814	ID_9
0days	l-back-toe__Movement	-0.934677	ID_10
0days	l-back-toe__Movement	0.57495879	ID_11
0days	l-back-toe__Movement	0.70774739	ID_12
0days	l-back-toe__Movement	0.51821809	ID_13

3days	l-back-toe__Movement	-1.3070101	ID_1
3days	l-back-toe__Movement	-0.950261	ID_2
3days	l-back-toe__Movement	-1.375857	ID_3
3days	l-back-toe__Movement	-1.0712494	ID_4
3days	l-back-toe__Movement	-1.4527488	ID_5
3days	l-back-toe__Movement	-0.7754522	ID_6
3days	l-back-toe__Movement	-1.0120016	ID_7
3days	l-back-toe__Movement	-1.684607	ID_8
7days	l-back-toe__Movement	-0.9393896	ID_1
7days	l-back-toe__Movement	-1.9767143	ID_2
7days	l-back-toe__Movement	-0.5922708	ID_3
7days	l-back-toe__Movement	-1.1917231	ID_4
7days	l-back-toe__Movement	-1.3039235	ID_5
7days	l-back-toe__Movement	-0.5461987	ID_6
7days	l-back-toe__Movement	-1.1634658	ID_7
7days	l-back-toe__Movement	-1.44731	ID_8
7days	l-back-toe__Movement	-1.3614519	ID_9
14days	l-back-toe__Movement	0.41138077	ID_1
14days	l-back-toe__Movement	0.33586402	ID_2
14days	l-back-toe__Movement	-1.4108377	ID_3
14days	l-back-toe__Movement	-1.6355577	ID_4
14days	l-back-toe__Movement	-1.5974239	ID_5
14days	l-back-toe__Movement	-0.0730451	ID_6
14days	l-back-toe__Movement	-0.7894132	ID_7
14days	l-back-toe__Movement	-1.0419263	ID_8
14days	l-back-toe__Movement	-2.0363561	ID_9
14days	l-back-toe__Movement	-0.8431799	ID_10
14days	l-back-toe__Movement	-0.2961896	ID_11
21days	l-back-toe__Movement	-0.7334589	ID_1
21days	l-back-toe__Movement	0.16867439	ID_2
21days	l-back-toe__Movement	-0.3295037	ID_3
21days	l-back-toe__Movement	-1.3069666	ID_4
21days	l-back-toe__Movement	-0.4545942	ID_5
21days	l-back-toe__Movement	-1.5645656	ID_6
21days	l-back-toe__Movement	-1.7102679	ID_7
21days	l-back-toe__Movement	-1.3163913	ID_8
21days	l-back-toe__Movement	-0.2712721	ID_9
21days	l-back-toe__Movement	-0.007773	ID_10
0days	l-elbow__Average_Height	-0.2393984	ID_1
0days	l-elbow__Average_Height	-0.0606596	ID_2
0days	l-elbow__Average_Height	0.05889582	ID_3
0days	l-elbow__Average_Height	0.05348408	ID_4
0days	l-elbow__Average_Height	-0.2840598	ID_5
0days	l-elbow__Average_Height	0.08663327	ID_6
0days	l-elbow__Average_Height	0.86839951	ID_7
0days	l-elbow__Average_Height	0.13338148	ID_8
0days	l-elbow__Average_Height	0.10046729	ID_9
0days	l-elbow__Average_Height	-0.2225656	ID_10
0days	l-elbow__Average_Height	-0.2256471	ID_11
0days	l-elbow__Average_Height	-0.1043292	ID_12
0days	l-elbow__Average_Height	0	ID_13
3days	l-elbow__Average_Height	-0.0945403	ID_1
3days	l-elbow__Average_Height	-0.2066039	ID_2
3days	l-elbow__Average_Height	-0.3239991	ID_3
3days	l-elbow__Average_Height	-0.2082555	ID_4
3days	l-elbow__Average_Height	-0.0155349	ID_5

3days	l-elbow__Average_Height	-0.2428056	ID_6
3days	l-elbow__Average_Height	-0.1569429	ID_7
3days	l-elbow__Average_Height	-0.0066386	ID_8
7days	l-elbow__Average_Height	0.16589829	ID_1
7days	l-elbow__Average_Height	-0.0325917	ID_2
7days	l-elbow__Average_Height	-0.2381256	ID_3
7days	l-elbow__Average_Height	-0.1196973	ID_4
7days	l-elbow__Average_Height	-0.3797344	ID_5
7days	l-elbow__Average_Height	0.06673845	ID_6
7days	l-elbow__Average_Height	0.16629383	ID_7
7days	l-elbow__Average_Height	-0.1595892	ID_8
7days	l-elbow__Average_Height	-0.2987942	ID_9
14days	l-elbow__Average_Height	-0.1311944	ID_1
14days	l-elbow__Average_Height	-0.2710208	ID_2
14days	l-elbow__Average_Height	0.21045108	ID_3
14days	l-elbow__Average_Height	0.51704939	ID_4
14days	l-elbow__Average_Height	0.33233005	ID_5
14days	l-elbow__Average_Height	-0.1913757	ID_6
14days	l-elbow__Average_Height	0.7371687	ID_7
14days	l-elbow__Average_Height	0.51850075	ID_8
14days	l-elbow__Average_Height	-0.1202261	ID_9
14days	l-elbow__Average_Height	0.17608252	ID_10
14days	l-elbow__Average_Height	0.14184654	ID_11
21days	l-elbow__Average_Height	-0.22229	ID_1
21days	l-elbow__Average_Height	-0.2523522	ID_2
21days	l-elbow__Average_Height	0.00876878	ID_3
21days	l-elbow__Average_Height	0.97198922	ID_4
21days	l-elbow__Average_Height	0.45282924	ID_5
21days	l-elbow__Average_Height	0.12554679	ID_6
21days	l-elbow__Average_Height	0.63911243	ID_7
21days	l-elbow__Average_Height	0.5608876	ID_8
21days	l-elbow__Average_Height	0.18487568	ID_9
0days	l-elbow__Movement	-0.3591778	ID_1
0days	l-elbow__Movement	-0.1485998	ID_2
0days	l-elbow__Movement	-0.250601	ID_3
0days	l-elbow__Movement	0.06269647	ID_4
0days	l-elbow__Movement	-0.5882387	ID_5
0days	l-elbow__Movement	0.36670743	ID_6
0days	l-elbow__Movement	1.46395694	ID_7
0days	l-elbow__Movement	0.72611005	ID_8
0days	l-elbow__Movement	0.07244689	ID_9
0days	l-elbow__Movement	0	ID_10
0days	l-elbow__Movement	-0.6767126	ID_11
0days	l-elbow__Movement	-0.2700936	ID_12
0days	l-elbow__Movement	0.06863677	ID_13
3days	l-elbow__Movement	-0.4701718	ID_1
3days	l-elbow__Movement	-0.869143	ID_2
3days	l-elbow__Movement	-0.874188	ID_3
3days	l-elbow__Movement	-0.5699819	ID_4
3days	l-elbow__Movement	-0.4102257	ID_5
3days	l-elbow__Movement	-0.7628235	ID_6
3days	l-elbow__Movement	-0.8789411	ID_7
3days	l-elbow__Movement	-0.3000482	ID_8
7days	l-elbow__Movement	-0.110808	ID_1
7days	l-elbow__Movement	0.02819215	ID_2
7days	l-elbow__Movement	-0.8159358	ID_3

7days	l-elbow__Movement	-0.2017046	ID_4
7days	l-elbow__Movement	-0.8158182	ID_5
7days	l-elbow__Movement	1.26708913	ID_6
7days	l-elbow__Movement	0.06676947	ID_7
7days	l-elbow__Movement	-0.5380342	ID_8
7days	l-elbow__Movement	-0.5854277	ID_9
14days	l-elbow__Movement	-0.3594977	ID_1
14days	l-elbow__Movement	-0.5746941	ID_2
14days	l-elbow__Movement	0.70404172	ID_3
14days	l-elbow__Movement	1.48744661	ID_4
14days	l-elbow__Movement	0.74460189	ID_5
14days	l-elbow__Movement	-0.4975155	ID_6
14days	l-elbow__Movement	1.19959825	ID_7
14days	l-elbow__Movement	0.91943454	ID_8
14days	l-elbow__Movement	-0.4305603	ID_9
14days	l-elbow__Movement	0.77137131	ID_10
14days	l-elbow__Movement	0.12683158	ID_11
21days	l-elbow__Movement	-0.4734081	ID_1
21days	l-elbow__Movement	-0.2018055	ID_2
21days	l-elbow__Movement	-0.4027433	ID_3
21days	l-elbow__Movement	2.29973789	ID_4
21days	l-elbow__Movement	0.62506938	ID_5
21days	l-elbow__Movement	0.26455313	ID_6
21days	l-elbow__Movement	1.56354487	ID_7
21days	l-elbow__Movement	1.2242387	ID_8
21days	l-elbow__Movement	0.02430408	ID_9
0days	l-front-toe-tip__Average_Hei	-0.0147911	ID_1
0days	l-front-toe-tip__Average_Hei	-0.4672062	ID_2
0days	l-front-toe-tip__Average_Hei	0.13754486	ID_3
0days	l-front-toe-tip__Average_Hei	-0.2127102	ID_4
0days	l-front-toe-tip__Average_Hei	-0.2197684	ID_5
0days	l-front-toe-tip__Average_Hei	0.11758264	ID_6
0days	l-front-toe-tip__Average_Hei	0.09879332	ID_7
0days	l-front-toe-tip__Average_Hei	0.01056145	ID_8
0days	l-front-toe-tip__Average_Hei	-0.0771106	ID_9
0days	l-front-toe-tip__Average_Hei	0	ID_10
0days	l-front-toe-tip__Average_Hei	-0.3761377	ID_11
0days	l-front-toe-tip__Average_Hei	0.15583334	ID_12
0days	l-front-toe-tip__Average_Hei	0.2226021	ID_13
3days	l-front-toe-tip__Average_Hei	-0.5414452	ID_1
3days	l-front-toe-tip__Average_Hei	-0.3469393	ID_2
3days	l-front-toe-tip__Average_Hei	-0.188103	ID_3
3days	l-front-toe-tip__Average_Hei	-0.6663616	ID_4
3days	l-front-toe-tip__Average_Hei	-0.5568749	ID_5
3days	l-front-toe-tip__Average_Hei	-0.3228904	ID_6
3days	l-front-toe-tip__Average_Hei	-0.5251257	ID_7
3days	l-front-toe-tip__Average_Hei	-0.4537633	ID_8
7days	l-front-toe-tip__Average_Hei	-0.3615072	ID_1
7days	l-front-toe-tip__Average_Hei	-0.4075054	ID_2
7days	l-front-toe-tip__Average_Hei	-0.3830309	ID_3
7days	l-front-toe-tip__Average_Hei	-0.3858967	ID_4
7days	l-front-toe-tip__Average_Hei	-0.269788	ID_5
7days	l-front-toe-tip__Average_Hei	-0.4060665	ID_6
7days	l-front-toe-tip__Average_Hei	-0.4506956	ID_7
7days	l-front-toe-tip__Average_Hei	-0.2462014	ID_8
7days	l-front-toe-tip__Average_Hei	-0.3652331	ID_9

14days	l-front-toe-tip__Average_Hei	-0.2869397	ID_1
14days	l-front-toe-tip__Average_Hei	-0.0884651	ID_2
14days	l-front-toe-tip__Average_Hei	-0.4246284	ID_3
14days	l-front-toe-tip__Average_Hei	-0.4654027	ID_4
14days	l-front-toe-tip__Average_Hei	0.13222713	ID_5
14days	l-front-toe-tip__Average_Hei	-0.646435	ID_6
14days	l-front-toe-tip__Average_Hei	-0.2675328	ID_7
14days	l-front-toe-tip__Average_Hei	-0.2986062	ID_8
14days	l-front-toe-tip__Average_Hei	-0.7209941	ID_9
14days	l-front-toe-tip__Average_Hei	-0.4827357	ID_10
14days	l-front-toe-tip__Average_Hei	0.03143259	ID_11
21days	l-front-toe-tip__Average_Hei	-0.3688372	ID_1
21days	l-front-toe-tip__Average_Hei	0.21574139	ID_2
21days	l-front-toe-tip__Average_Hei	-0.0419055	ID_3
21days	l-front-toe-tip__Average_Hei	0.1045365	ID_4
21days	l-front-toe-tip__Average_Hei	-0.0544842	ID_5
21days	l-front-toe-tip__Average_Hei	-0.5595758	ID_6
21days	l-front-toe-tip__Average_Hei	0.09082528	ID_7
21days	l-front-toe-tip__Average_Hei	-0.4457039	ID_8
21days	l-front-toe-tip__Average_Hei	-0.3679859	ID_9
21days	l-front-toe-tip__Average_Hei	-0.1242323	ID_10
0days	l-front-toe-tip__Movement	-0.1104458	ID_1
0days	l-front-toe-tip__Movement	-0.4465396	ID_2
0days	l-front-toe-tip__Movement	0.23189562	ID_3
0days	l-front-toe-tip__Movement	0.5418076	ID_4
0days	l-front-toe-tip__Movement	-0.2357491	ID_5
0days	l-front-toe-tip__Movement	0.34702465	ID_6
0days	l-front-toe-tip__Movement	0.57335941	ID_7
0days	l-front-toe-tip__Movement	2.08940108	ID_8
0days	l-front-toe-tip__Movement	0	ID_9
0days	l-front-toe-tip__Movement	-0.5582958	ID_10
0days	l-front-toe-tip__Movement	-0.3919764	ID_11
0days	l-front-toe-tip__Movement	-0.1937157	ID_12
0days	l-front-toe-tip__Movement	0.3474683	ID_13
3days	l-front-toe-tip__Movement	-1.0127956	ID_1
3days	l-front-toe-tip__Movement	-0.5421167	ID_2
3days	l-front-toe-tip__Movement	-0.177845	ID_3
3days	l-front-toe-tip__Movement	-0.7988863	ID_4
3days	l-front-toe-tip__Movement	-0.7890427	ID_5
3days	l-front-toe-tip__Movement	-0.3779705	ID_6
3days	l-front-toe-tip__Movement	-0.7549858	ID_7
3days	l-front-toe-tip__Movement	-1.1554824	ID_8
7days	l-front-toe-tip__Movement	-0.6854878	ID_1
7days	l-front-toe-tip__Movement	-0.6253576	ID_2
7days	l-front-toe-tip__Movement	-0.5864964	ID_3
7days	l-front-toe-tip__Movement	-0.7229588	ID_4
7days	l-front-toe-tip__Movement	-0.8602315	ID_5
7days	l-front-toe-tip__Movement	-0.6352478	ID_6
7days	l-front-toe-tip__Movement	-0.9297532	ID_7
7days	l-front-toe-tip__Movement	-0.7220486	ID_8
7days	l-front-toe-tip__Movement	-1.0779973	ID_9
14days	l-front-toe-tip__Movement	-0.4652339	ID_1
14days	l-front-toe-tip__Movement	-0.419892	ID_2
14days	l-front-toe-tip__Movement	-0.4886277	ID_3
14days	l-front-toe-tip__Movement	-0.4357413	ID_4
14days	l-front-toe-tip__Movement	0.58281312	ID_5

14days	l-front-toe-tip__Movement	-0.927662	ID_6
14days	l-front-toe-tip__Movement	0.16564881	ID_7
14days	l-front-toe-tip__Movement	-0.1091076	ID_8
14days	l-front-toe-tip__Movement	-1.4602212	ID_9
14days	l-front-toe-tip__Movement	-0.0918883	ID_10
14days	l-front-toe-tip__Movement	0.38813104	ID_11
21days	l-front-toe-tip__Movement	-0.1569518	ID_1
21days	l-front-toe-tip__Movement	-0.1643208	ID_2
21days	l-front-toe-tip__Movement	0.27387035	ID_3
21days	l-front-toe-tip__Movement	0.71802332	ID_4
21days	l-front-toe-tip__Movement	-0.2800393	ID_5
21days	l-front-toe-tip__Movement	-1.1417461	ID_6
21days	l-front-toe-tip__Movement	0.08672806	ID_7
21days	l-front-toe-tip__Movement	0.04293131	ID_8
21days	l-front-toe-tip__Movement	-1.1301173	ID_9
21days	l-front-toe-tip__Movement	-0.2582717	ID_10
0days	l-head__Average_Height	-0.0399667	ID_1
0days	l-head__Average_Height	0.44070507	ID_2
0days	l-head__Average_Height	1.04710834	ID_3
0days	l-head__Average_Height	1.78224115	ID_4
0days	l-head__Average_Height	0.87841401	ID_5
0days	l-head__Average_Height	-0.2647838	ID_6
0days	l-head__Average_Height	0.03996673	ID_7
0days	l-head__Average_Height	0.52762914	ID_8
0days	l-head__Average_Height	-0.6174724	ID_9
0days	l-head__Average_Height	-0.1490404	ID_10
0days	l-head__Average_Height	-0.1169046	ID_11
0days	l-head__Average_Height	-0.3830891	ID_12
3days	l-head__Average_Height	-0.6172452	ID_1
3days	l-head__Average_Height	-0.7234231	ID_2
3days	l-head__Average_Height	-0.6205401	ID_3
3days	l-head__Average_Height	-0.4518147	ID_4
3days	l-head__Average_Height	-0.7060306	ID_5
3days	l-head__Average_Height	-0.3735361	ID_6
3days	l-head__Average_Height	-0.9765378	ID_7
3days	l-head__Average_Height	-0.1629469	ID_8
7days	l-head__Average_Height	0.81704756	ID_1
7days	l-head__Average_Height	-0.630912	ID_2
7days	l-head__Average_Height	-0.8995152	ID_3
7days	l-head__Average_Height	-1.103179	ID_4
7days	l-head__Average_Height	-1.1233793	ID_5
7days	l-head__Average_Height	-0.7840083	ID_6
7days	l-head__Average_Height	-0.7860921	ID_7
7days	l-head__Average_Height	-0.4804879	ID_8
7days	l-head__Average_Height	-0.6033511	ID_9
14days	l-head__Average_Height	0.1112093	ID_1
14days	l-head__Average_Height	-0.3559697	ID_2
14days	l-head__Average_Height	-0.5079412	ID_3
14days	l-head__Average_Height	1.54130125	ID_4
14days	l-head__Average_Height	0.42462363	ID_5
14days	l-head__Average_Height	-0.2031307	ID_6
14days	l-head__Average_Height	-0.6163382	ID_7
14days	l-head__Average_Height	-0.6897253	ID_8
14days	l-head__Average_Height	-0.0913937	ID_9
14days	l-head__Average_Height	0.23132581	ID_10
14days	l-head__Average_Height	-0.1329957	ID_11

21days	l-head__Average_Height	1.36458986	ID_1
21days	l-head__Average_Height	0.04135524	ID_2
21days	l-head__Average_Height	-0.6929751	ID_3
21days	l-head__Average_Height	-0.2387244	ID_4
21days	l-head__Average_Height	-0.325672	ID_5
21days	l-head__Average_Height	-0.579733	ID_6
21days	l-head__Average_Height	-0.441725	ID_7
21days	l-head__Average_Height	-0.0284937	ID_8
21days	l-head__Average_Height	-1.0952739	ID_9
21days	l-head__Average_Height	-0.6293497	ID_10
0days	l-head__Movement	-0.2423463	ID_1
0days	l-head__Movement	2.99432841	ID_2
0days	l-head__Movement	0.27923577	ID_3
0days	l-head__Movement	4.02776935	ID_4
0days	l-head__Movement	1.75034694	ID_5
0days	l-head__Movement	-0.6740571	ID_6
0days	l-head__Movement	0.05166077	ID_7
0days	l-head__Movement	0.94065384	ID_8
0days	l-head__Movement	-1.9499972	ID_9
0days	l-head__Movement	-0.0516608	ID_10
0days	l-head__Movement	-1.0674218	ID_11
0days	l-head__Movement	-1.4630043	ID_12
3days	l-head__Movement	-2.8558414	ID_1
3days	l-head__Movement	-2.5586508	ID_2
3days	l-head__Movement	-2.3237033	ID_3
3days	l-head__Movement	-2.0557824	ID_4
3days	l-head__Movement	-2.5158933	ID_5
3days	l-head__Movement	-1.0614751	ID_6
3days	l-head__Movement	-2.6521061	ID_7
3days	l-head__Movement	-0.46177	ID_8
7days	l-head__Movement	1.62925398	ID_1
7days	l-head__Movement	-1.5110295	ID_2
7days	l-head__Movement	-2.673798	ID_3
7days	l-head__Movement	-2.8680211	ID_4
7days	l-head__Movement	-3.2296941	ID_5
7days	l-head__Movement	-1.8488808	ID_6
7days	l-head__Movement	-2.3014372	ID_7
7days	l-head__Movement	-2.0484046	ID_8
7days	l-head__Movement	-1.9557916	ID_9
14days	l-head__Movement	-0.1129699	ID_1
14days	l-head__Movement	-0.9414617	ID_2
14days	l-head__Movement	-2.2444634	ID_3
14days	l-head__Movement	2.33652881	ID_4
14days	l-head__Movement	2.5672687	ID_5
14days	l-head__Movement	-1.7412132	ID_6
14days	l-head__Movement	-2.1233444	ID_7
14days	l-head__Movement	-2.1287506	ID_8
14days	l-head__Movement	-0.4323665	ID_9
14days	l-head__Movement	-1.3024088	ID_10
14days	l-head__Movement	-0.9574556	ID_11
21days	l-head__Movement	2.49882834	ID_1
21days	l-head__Movement	-0.1015888	ID_2
21days	l-head__Movement	-2.488392	ID_3
21days	l-head__Movement	-2.0341705	ID_4
21days	l-head__Movement	-1.1551673	ID_5
21days	l-head__Movement	-2.6573545	ID_6

21days	l-head__Movement	-1.6932782	ID_7
21days	l-head__Movement	-0.9107645	ID_8
21days	l-head__Movement	-2.940025	ID_9
21days	l-head__Movement	-2.0159836	ID_10
0days	l-hip__Average_Height	-0.44478	ID_1
0days	l-hip__Average_Height	-0.0779021	ID_2
0days	l-hip__Average_Height	0.22000872	ID_3
0days	l-hip__Average_Height	0.37772816	ID_4
0days	l-hip__Average_Height	0.06173439	ID_5
0days	l-hip__Average_Height	0.28655259	ID_6
0days	l-hip__Average_Height	0	ID_7
0days	l-hip__Average_Height	0.28380395	ID_8
0days	l-hip__Average_Height	0.09884662	ID_9
0days	l-hip__Average_Height	-0.3924345	ID_10
0days	l-hip__Average_Height	-0.2661656	ID_11
0days	l-hip__Average_Height	-0.1642707	ID_12
0days	l-hip__Average_Height	-0.3138523	ID_13
3days	l-hip__Average_Height	-0.4424023	ID_1
3days	l-hip__Average_Height	-0.3450636	ID_2
3days	l-hip__Average_Height	-0.318778	ID_3
3days	l-hip__Average_Height	-0.3109952	ID_4
3days	l-hip__Average_Height	-0.3595845	ID_5
3days	l-hip__Average_Height	-0.4389856	ID_6
3days	l-hip__Average_Height	0.06458484	ID_7
3days	l-hip__Average_Height	0.28541868	ID_8
7days	l-hip__Average_Height	-0.5057745	ID_1
7days	l-hip__Average_Height	0.01702515	ID_2
7days	l-hip__Average_Height	-0.5614886	ID_3
7days	l-hip__Average_Height	-0.2643041	ID_4
7days	l-hip__Average_Height	0.17797797	ID_5
7days	l-hip__Average_Height	-0.4972085	ID_6
7days	l-hip__Average_Height	-0.1133062	ID_7
7days	l-hip__Average_Height	-0.4577949	ID_8
7days	l-hip__Average_Height	0.11693554	ID_9
14days	l-hip__Average_Height	0.04016729	ID_1
14days	l-hip__Average_Height	-0.1670369	ID_2
14days	l-hip__Average_Height	0.33361269	ID_3
14days	l-hip__Average_Height	-0.5270553	ID_4
14days	l-hip__Average_Height	-0.4989137	ID_5
14days	l-hip__Average_Height	-0.5581158	ID_6
14days	l-hip__Average_Height	-0.5762918	ID_7
14days	l-hip__Average_Height	-0.739587	ID_8
14days	l-hip__Average_Height	0.21458182	ID_9
14days	l-hip__Average_Height	-0.4010188	ID_10
14days	l-hip__Average_Height	-0.3005449	ID_11
21days	l-hip__Average_Height	-0.1404379	ID_1
21days	l-hip__Average_Height	-0.1076918	ID_2
21days	l-hip__Average_Height	0.17621642	ID_3
21days	l-hip__Average_Height	-0.3595326	ID_4
21days	l-hip__Average_Height	-0.2215566	ID_5
21days	l-hip__Average_Height	-0.3710596	ID_6
21days	l-hip__Average_Height	0.26581864	ID_7
21days	l-hip__Average_Height	-0.5029991	ID_8
21days	l-hip__Average_Height	-0.3125981	ID_9
21days	l-hip__Average_Height	0.17901183	ID_10
0days	l-hip__Movement	-0.449144	ID_1

0days	l-hip__Movement	0.3985917	ID_2
0days	l-hip__Movement	0	ID_3
0days	l-hip__Movement	1.12620366	ID_4
0days	l-hip__Movement	-0.1733888	ID_5
0days	l-hip__Movement	1.14533099	ID_6
0days	l-hip__Movement	1.09662952	ID_7
0days	l-hip__Movement	0.53047927	ID_8
0days	l-hip__Movement	0.75835218	ID_9
0days	l-hip__Movement	-0.1286163	ID_10
0days	l-hip__Movement	-0.1952401	ID_11
0days	l-hip__Movement	-0.2297682	ID_12
0days	l-hip__Movement	-0.163768	ID_13
3days	l-hip__Movement	-1.1155466	ID_1
3days	l-hip__Movement	-0.6857796	ID_2
3days	l-hip__Movement	-0.4757547	ID_3
3days	l-hip__Movement	-0.3524353	ID_4
3days	l-hip__Movement	-0.8004561	ID_5
3days	l-hip__Movement	-0.4620348	ID_6
3days	l-hip__Movement	0.95327937	ID_7
3days	l-hip__Movement	0.5827101	ID_8
7days	l-hip__Movement	-0.5792133	ID_1
7days	l-hip__Movement	-0.0686231	ID_2
7days	l-hip__Movement	-1.2099425	ID_3
7days	l-hip__Movement	-0.577758	ID_4
7days	l-hip__Movement	0.17055567	ID_5
7days	l-hip__Movement	-0.4865024	ID_6
7days	l-hip__Movement	0.15170056	ID_7
7days	l-hip__Movement	-0.3605221	ID_8
7days	l-hip__Movement	0.36856975	ID_9
14days	l-hip__Movement	0.25712958	ID_1
14days	l-hip__Movement	0.42699317	ID_2
14days	l-hip__Movement	-0.0199676	ID_3
14days	l-hip__Movement	-0.964921	ID_4
14days	l-hip__Movement	-0.444228	ID_5
14days	l-hip__Movement	-1.1068478	ID_6
14days	l-hip__Movement	-0.8293296	ID_7
14days	l-hip__Movement	-0.6747009	ID_8
14days	l-hip__Movement	1.24007211	ID_9
14days	l-hip__Movement	-0.0778198	ID_10
14days	l-hip__Movement	0.20605933	ID_11
21days	l-hip__Movement	0.00652735	ID_1
21days	l-hip__Movement	0.12645404	ID_2
21days	l-hip__Movement	-0.126433	ID_3
21days	l-hip__Movement	-0.6851101	ID_4
21days	l-hip__Movement	-0.3674863	ID_5
21days	l-hip__Movement	-0.2466754	ID_6
21days	l-hip__Movement	0.46760155	ID_7
21days	l-hip__Movement	-0.6284351	ID_8
21days	l-hip__Movement	-0.9116065	ID_9
21days	l-hip__Movement	1.08235313	ID_10
0days	l-iliac-crest__Average_Height	-0.3628712	ID_1
0days	l-iliac-crest__Average_Height	0.13439664	ID_2
0days	l-iliac-crest__Average_Height	0.38431837	ID_3
0days	l-iliac-crest__Average_Height	0.57175904	ID_4
0days	l-iliac-crest__Average_Height	-0.3900182	ID_5
0days	l-iliac-crest__Average_Height	0	ID_6

0days	l-iliac-crest__Average_Height	0.19718105	ID_7
0days	l-iliac-crest__Average_Height	-0.136695	ID_8
0days	l-iliac-crest__Average_Height	0.23736064	ID_9
0days	l-iliac-crest__Average_Height	-0.031464	ID_10
0days	l-iliac-crest__Average_Height	-0.3975253	ID_11
0days	l-iliac-crest__Average_Height	-0.0896213	ID_12
0days	l-iliac-crest__Average_Height	0.08435949	ID_13
3days	l-iliac-crest__Average_Height	-0.5494283	ID_1
3days	l-iliac-crest__Average_Height	-0.4668579	ID_2
3days	l-iliac-crest__Average_Height	-0.2158797	ID_3
3days	l-iliac-crest__Average_Height	-0.1701912	ID_4
3days	l-iliac-crest__Average_Height	-0.1780643	ID_5
3days	l-iliac-crest__Average_Height	-0.1401275	ID_6
3days	l-iliac-crest__Average_Height	0.21172895	ID_7
3days	l-iliac-crest__Average_Height	0.28233014	ID_8
7days	l-iliac-crest__Average_Height	-0.3104302	ID_1
7days	l-iliac-crest__Average_Height	-0.2572337	ID_2
7days	l-iliac-crest__Average_Height	-0.5490395	ID_3
7days	l-iliac-crest__Average_Height	-0.4375251	ID_4
7days	l-iliac-crest__Average_Height	-0.0306272	ID_5
7days	l-iliac-crest__Average_Height	-0.285001	ID_6
7days	l-iliac-crest__Average_Height	0.36952528	ID_7
7days	l-iliac-crest__Average_Height	0.00995395	ID_8
14days	l-iliac-crest__Average_Height	-0.2170776	ID_9
14days	l-iliac-crest__Average_Height	0.00879214	ID_1
14days	l-iliac-crest__Average_Height	-0.4679329	ID_2
14days	l-iliac-crest__Average_Height	-0.5622044	ID_3
14days	l-iliac-crest__Average_Height	-0.442494	ID_4
14days	l-iliac-crest__Average_Height	-0.3832791	ID_5
14days	l-iliac-crest__Average_Height	-0.4039169	ID_6
14days	l-iliac-crest__Average_Height	-0.5298417	ID_7
14days	l-iliac-crest__Average_Height	0.38699294	ID_8
14days	l-iliac-crest__Average_Height	-0.5183808	ID_9
14days	l-iliac-crest__Average_Height	-0.1409395	ID_10
21days	l-iliac-crest__Average_Height	0.15711757	ID_11
21days	l-iliac-crest__Average_Height	-0.0382242	ID_1
21days	l-iliac-crest__Average_Height	-0.2682869	ID_2
21days	l-iliac-crest__Average_Height	-0.1676372	ID_3
21days	l-iliac-crest__Average_Height	0.05191179	ID_4
21days	l-iliac-crest__Average_Height	-0.1616574	ID_5
21days	l-iliac-crest__Average_Height	0.30549848	ID_6
21days	l-iliac-crest__Average_Height	-0.1079949	ID_7
21days	l-iliac-crest__Average_Height	-0.5673959	ID_8
21days	l-iliac-crest__Average_Height	0.65186404	ID_9
0days	l-iliac-crest__Movement	-0.9264023	ID_10
0days	l-iliac-crest__Movement	-0.2966488	ID_1
0days	l-iliac-crest__Movement	0	ID_2
0days	l-iliac-crest__Movement	0.96675868	ID_3
0days	l-iliac-crest__Movement	-0.9852611	ID_4
0days	l-iliac-crest__Movement	0.91055	ID_5
0days	l-iliac-crest__Movement	0.63231218	ID_6
0days	l-iliac-crest__Movement	-1.0445128	ID_7
0days	l-iliac-crest__Movement	0.80678632	ID_8
0days	l-iliac-crest__Movement	0.47001061	ID_9
0days	l-iliac-crest__Movement	-1.0238567	ID_10
0days	l-iliac-crest__Movement	-0.3866982	ID_11

0days	l-iliac-crest__Movement	0.08397723	ID_12
3days	l-iliac-crest__Movement	-1.31425	ID_13
3days	l-iliac-crest__Movement	-1.1431332	ID_1
3days	l-iliac-crest__Movement	-0.648544	ID_2
3days	l-iliac-crest__Movement	-0.4993067	ID_3
3days	l-iliac-crest__Movement	-0.8674843	ID_4
3days	l-iliac-crest__Movement	-0.7261842	ID_5
3days	l-iliac-crest__Movement	0.81199157	ID_6
3days	l-iliac-crest__Movement	0.47738176	ID_7
7days	l-iliac-crest__Movement	-0.3812341	ID_8
7days	l-iliac-crest__Movement	-1.2193037	ID_1
7days	l-iliac-crest__Movement	-1.261826	ID_2
7days	l-iliac-crest__Movement	-0.7895844	ID_3
7days	l-iliac-crest__Movement	-0.3242382	ID_4
7days	l-iliac-crest__Movement	-0.8407973	ID_5
7days	l-iliac-crest__Movement	0.71135336	ID_6
7days	l-iliac-crest__Movement	-0.0333127	ID_7
14days	l-iliac-crest__Movement	0.06016784	ID_8
14days	l-iliac-crest__Movement	0.36320639	ID_9
14days	l-iliac-crest__Movement	-1.5307449	ID_1
14days	l-iliac-crest__Movement	-1.408449	ID_2
14days	l-iliac-crest__Movement	-1.4738531	ID_3
14days	l-iliac-crest__Movement	-1.4955974	ID_4
14days	l-iliac-crest__Movement	-0.5683011	ID_5
14days	l-iliac-crest__Movement	-0.6846737	ID_6
14days	l-iliac-crest__Movement	0.74989581	ID_7
14days	l-iliac-crest__Movement	-0.8236276	ID_8
14days	l-iliac-crest__Movement	-0.5397105	ID_9
21days	l-iliac-crest__Movement	-0.1947859	ID_10
21days	l-iliac-crest__Movement	-0.2728709	ID_11
21days	l-iliac-crest__Movement	-0.6709748	ID_1
21days	l-iliac-crest__Movement	-0.406304	ID_2
21days	l-iliac-crest__Movement	-0.450563	ID_3
21days	l-iliac-crest__Movement	-0.9414072	ID_4
21days	l-iliac-crest__Movement	0.18955474	ID_5
21days	l-iliac-crest__Movement	-0.0912889	ID_6
21days	l-iliac-crest__Movement	-1.4079614	ID_7
21days	l-iliac-crest__Movement	0.56774494	ID_8
0days	l-shoulder__Average_Height	-0.2639678	ID_1
0days	l-shoulder__Average_Height	-0.2569669	ID_2
0days	l-shoulder__Average_Height	-0.2782261	ID_3
0days	l-shoulder__Average_Height	0.06219628	ID_4
0days	l-shoulder__Average_Height	0.33123189	ID_5
0days	l-shoulder__Average_Height	0.03136468	ID_6
0days	l-shoulder__Average_Height	0.14397045	ID_7
0days	l-shoulder__Average_Height	0.92531116	ID_8
0days	l-shoulder__Average_Height	0.24068171	ID_9
0days	l-shoulder__Average_Height	-0.0313647	ID_10
0days	l-shoulder__Average_Height	-0.227171	ID_11
0days	l-shoulder__Average_Height	-0.1833853	ID_12
3days	l-shoulder__Average_Height	-0.3847011	ID_1
3days	l-shoulder__Average_Height	-0.2549553	ID_2
3days	l-shoulder__Average_Height	-0.3930354	ID_3
3days	l-shoulder__Average_Height	-0.1082167	ID_4
3days	l-shoulder__Average_Height	0.06229494	ID_5
7days	l-shoulder__Average_Height	-0.1833509	ID_1

7days	l-shoulder__Average_Height	-0.2264867	ID_2
7days	l-shoulder__Average_Height	-0.1820852	ID_3
7days	l-shoulder__Average_Height	-0.3811283	ID_4
7days	l-shoulder__Average_Height	-0.3155338	ID_5
7days	l-shoulder__Average_Height	-0.1605345	ID_6
7days	l-shoulder__Average_Height	0.14939833	ID_7
7days	l-shoulder__Average_Height	-0.2664628	ID_8
14days	l-shoulder__Average_Height	-0.0337489	ID_1
14days	l-shoulder__Average_Height	-0.3586667	ID_2
14days	l-shoulder__Average_Height	0.02657494	ID_3
14days	l-shoulder__Average_Height	0.22786212	ID_4
14days	l-shoulder__Average_Height	0.00729058	ID_5
14days	l-shoulder__Average_Height	-0.0236413	ID_6
14days	l-shoulder__Average_Height	0.11387469	ID_7
14days	l-shoulder__Average_Height	0.18105948	ID_8
14days	l-shoulder__Average_Height	0.2490173	ID_9
21days	l-shoulder__Average_Height	-0.2036713	ID_1
21days	l-shoulder__Average_Height	-0.2287081	ID_2
21days	l-shoulder__Average_Height	-0.0517059	ID_3
21days	l-shoulder__Average_Height	0.17344833	ID_4
21days	l-shoulder__Average_Height	-0.2653139	ID_5
21days	l-shoulder__Average_Height	-0.177478	ID_6
21days	l-shoulder__Average_Height	-0.0956549	ID_7
21days	l-shoulder__Average_Height	0.31921705	ID_8
21days	l-shoulder__Average_Height	0.01745091	ID_9
21days	l-shoulder__Average_Height	0.02632362	ID_10
0days	l-shoulder__Movement	-0.4993784	ID_1
0days	l-shoulder__Movement	-0.3641267	ID_2
0days	l-shoulder__Movement	-0.3023594	ID_3
0days	l-shoulder__Movement	0.16173306	ID_4
0days	l-shoulder__Movement	-0.0467876	ID_5
0days	l-shoulder__Movement	0.1527792	ID_6
0days	l-shoulder__Movement	0.54277713	ID_7
0days	l-shoulder__Movement	2.37741519	ID_8
0days	l-shoulder__Movement	0.0526326	ID_9
0days	l-shoulder__Movement	0.04678756	ID_10
0days	l-shoulder__Movement	-0.5659054	ID_11
0days	l-shoulder__Movement	-0.3075269	ID_12
3days	l-shoulder__Movement	-0.9211351	ID_1
3days	l-shoulder__Movement	-0.8850393	ID_2
3days	l-shoulder__Movement	-0.7711989	ID_3
3days	l-shoulder__Movement	-0.574643	ID_4
3days	l-shoulder__Movement	-0.358047	ID_5
7days	l-shoulder__Movement	-0.8068592	ID_1
7days	l-shoulder__Movement	-0.8889002	ID_2
7days	l-shoulder__Movement	-0.690238	ID_3
7days	l-shoulder__Movement	-0.9319632	ID_4
7days	l-shoulder__Movement	-0.8581543	ID_5
7days	l-shoulder__Movement	-0.6503535	ID_6
7days	l-shoulder__Movement	-0.4490889	ID_7
7days	l-shoulder__Movement	-0.4100919	ID_8
14days	l-shoulder__Movement	-0.4189732	ID_1
14days	l-shoulder__Movement	-0.908218	ID_2
14days	l-shoulder__Movement	-0.1772168	ID_3
14days	l-shoulder__Movement	0.10042066	ID_4
14days	l-shoulder__Movement	-0.5304521	ID_5

14days	l-shoulder__Movement	-0.3574914	ID_6
14days	l-shoulder__Movement	-0.1376612	ID_7
14days	l-shoulder__Movement	0.0846998	ID_8
14days	l-shoulder__Movement	0.50607172	ID_9
21days	l-shoulder__Movement	-0.2942903	ID_1
21days	l-shoulder__Movement	-0.4887408	ID_2
21days	l-shoulder__Movement	-0.6515596	ID_3
21days	l-shoulder__Movement	-0.2397791	ID_4
21days	l-shoulder__Movement	-0.7298592	ID_5
21days	l-shoulder__Movement	-0.7385215	ID_6
21days	l-shoulder__Movement	-0.3908298	ID_7
21days	l-shoulder__Movement	0.04704945	ID_8
21days	l-shoulder__Movement	-0.5734602	ID_9
21days	l-shoulder__Movement	-0.251682	ID_10
0days	l-tail-base__Average_Height	-0.3192629	ID_1
0days	l-tail-base__Average_Height	0.06050594	ID_2
0days	l-tail-base__Average_Height	-0.2867967	ID_3
0days	l-tail-base__Average_Height	0.70787679	ID_4
0days	l-tail-base__Average_Height	-0.3067845	ID_5
0days	l-tail-base__Average_Height	0	ID_6
0days	l-tail-base__Average_Height	0.02259488	ID_7
0days	l-tail-base__Average_Height	0.32524338	ID_8
0days	l-tail-base__Average_Height	0.02259161	ID_9
0days	l-tail-base__Average_Height	-0.3950668	ID_10
0days	l-tail-base__Average_Height	-0.3294701	ID_11
0days	l-tail-base__Average_Height	-0.6811874	ID_12
0days	l-tail-base__Average_Height	0.21650675	ID_13
3days	l-tail-base__Average_Height	-0.2851315	ID_1
3days	l-tail-base__Average_Height	-0.3042199	ID_2
3days	l-tail-base__Average_Height	-0.324553	ID_3
3days	l-tail-base__Average_Height	-0.0835919	ID_4
3days	l-tail-base__Average_Height	0.11254996	ID_5
3days	l-tail-base__Average_Height	-0.3287968	ID_6
3days	l-tail-base__Average_Height	0.64616202	ID_7
3days	l-tail-base__Average_Height	0.98984473	ID_8
7days	l-tail-base__Average_Height	-0.19543	ID_1
7days	l-tail-base__Average_Height	-0.2014654	ID_2
7days	l-tail-base__Average_Height	-0.6513686	ID_3
7days	l-tail-base__Average_Height	0.08705057	ID_4
7days	l-tail-base__Average_Height	-0.5485664	ID_5
7days	l-tail-base__Average_Height	-0.1128758	ID_6
7days	l-tail-base__Average_Height	0.05543214	ID_7
7days	l-tail-base__Average_Height	-0.4786034	ID_8
7days	l-tail-base__Average_Height	0.25057599	ID_9
14days	l-tail-base__Average_Height	0.60247966	ID_1
14days	l-tail-base__Average_Height	-0.5164752	ID_2
14days	l-tail-base__Average_Height	1.14574511	ID_3
14days	l-tail-base__Average_Height	-0.339392	ID_4
14days	l-tail-base__Average_Height	-0.1848235	ID_5
14days	l-tail-base__Average_Height	-0.4406353	ID_6
14days	l-tail-base__Average_Height	-0.8135411	ID_7
14days	l-tail-base__Average_Height	-0.535126	ID_8
14days	l-tail-base__Average_Height	0.96679538	ID_9
14days	l-tail-base__Average_Height	-0.0303554	ID_10
14days	l-tail-base__Average_Height	-0.3294469	ID_11
21days	l-tail-base__Average_Height	0.04145865	ID_1

21days	l-tail-base__Average_Height	-0.5190112	ID_2
21days	l-tail-base__Average_Height	-0.352422	ID_3
21days	l-tail-base__Average_Height	0.19250263	ID_4
21days	l-tail-base__Average_Height	-0.3555155	ID_5
21days	l-tail-base__Average_Height	-0.1853749	ID_6
21days	l-tail-base__Average_Height	-0.2815834	ID_7
21days	l-tail-base__Average_Height	-0.192806	ID_8
21days	l-tail-base__Average_Height	-0.6894058	ID_9
21days	l-tail-base__Average_Height	-0.1881053	ID_10
0days	l-tail-base__Movement	-1.4445186	ID_1
0days	l-tail-base__Movement	0.82770614	ID_2
0days	l-tail-base__Movement	-1.3344519	ID_3
0days	l-tail-base__Movement	1.09139114	ID_4
0days	l-tail-base__Movement	-1.266019	ID_5
0days	l-tail-base__Movement	0.06382176	ID_6
0days	l-tail-base__Movement	0.12528136	ID_7
0days	l-tail-base__Movement	0	ID_8
0days	l-tail-base__Movement	0.07114391	ID_9
0days	l-tail-base__Movement	-1.0333938	ID_10
0days	l-tail-base__Movement	-1.6426021	ID_11
0days	l-tail-base__Movement	-2.000292	ID_12
0days	l-tail-base__Movement	0.45592032	ID_13
3days	l-tail-base__Movement	-1.3518024	ID_1
3days	l-tail-base__Movement	-1.026158	ID_2
3days	l-tail-base__Movement	-1.0562546	ID_3
3days	l-tail-base__Movement	-0.7828295	ID_4
3days	l-tail-base__Movement	-0.7770649	ID_5
3days	l-tail-base__Movement	-0.9997812	ID_6
3days	l-tail-base__Movement	0.71321414	ID_7
3days	l-tail-base__Movement	1.08829781	ID_8
7days	l-tail-base__Movement	-1.437235	ID_1
7days	l-tail-base__Movement	0.20246082	ID_2
7days	l-tail-base__Movement	-1.6303672	ID_3
7days	l-tail-base__Movement	-0.5314102	ID_4
7days	l-tail-base__Movement	-0.9261191	ID_5
7days	l-tail-base__Movement	0.2067443	ID_6
7days	l-tail-base__Movement	-0.4474122	ID_7
7days	l-tail-base__Movement	-1.3791844	ID_8
7days	l-tail-base__Movement	0.51120546	ID_9
14days	l-tail-base__Movement	0.77962477	ID_1
14days	l-tail-base__Movement	-0.9008344	ID_2
14days	l-tail-base__Movement	0.57259416	ID_3
14days	l-tail-base__Movement	-1.7896185	ID_4
14days	l-tail-base__Movement	-1.5304171	ID_5
14days	l-tail-base__Movement	-1.5906238	ID_6
14days	l-tail-base__Movement	-2.0533953	ID_7
14days	l-tail-base__Movement	-1.7541873	ID_8
14days	l-tail-base__Movement	1.02715145	ID_9
14days	l-tail-base__Movement	-0.063508	ID_10
14days	l-tail-base__Movement	-0.7408715	ID_11
21days	l-tail-base__Movement	-0.5553334	ID_1
21days	l-tail-base__Movement	-1.6746836	ID_2
21days	l-tail-base__Movement	-1.4230839	ID_3
21days	l-tail-base__Movement	-0.7162338	ID_4
21days	l-tail-base__Movement	-1.0745174	ID_5
21days	l-tail-base__Movement	0.12446746	ID_6

21days	l-tail-base__Movement	-0.8875311	ID_7
21days	l-tail-base__Movement	-1.0737186	ID_8
21days	l-tail-base__Movement	-1.550028	ID_9
21days	l-tail-base__Movement	-0.6307086	ID_10
0days	l-wrist__Average_Height	0	ID_1
0days	l-wrist__Average_Height	-0.9168882	ID_2
0days	l-wrist__Average_Height	0.13156143	ID_3
0days	l-wrist__Average_Height	-0.0089616	ID_4
0days	l-wrist__Average_Height	-0.8736788	ID_5
0days	l-wrist__Average_Height	0.42761381	ID_6
0days	l-wrist__Average_Height	0.16573231	ID_7
0days	l-wrist__Average_Height	-0.5073333	ID_8
0days	l-wrist__Average_Height	0.26751428	ID_9
0days	l-wrist__Average_Height	-0.2163956	ID_10
0days	l-wrist__Average_Height	-0.7771338	ID_11
0days	l-wrist__Average_Height	0.05496432	ID_12
0days	l-wrist__Average_Height	0.46029332	ID_13
3days	l-wrist__Average_Height	-1.2033035	ID_1
3days	l-wrist__Average_Height	-0.5027445	ID_2
3days	l-wrist__Average_Height	-0.6602885	ID_3
3days	l-wrist__Average_Height	-1.2235549	ID_4
3days	l-wrist__Average_Height	-1.2686132	ID_5
3days	l-wrist__Average_Height	-0.6346261	ID_6
3days	l-wrist__Average_Height	-0.6605776	ID_7
3days	l-wrist__Average_Height	-0.5429727	ID_8
7days	l-wrist__Average_Height	-0.3656664	ID_1
7days	l-wrist__Average_Height	-0.7288855	ID_2
7days	l-wrist__Average_Height	-0.4997624	ID_3
7days	l-wrist__Average_Height	-0.9261974	ID_4
7days	l-wrist__Average_Height	-0.4650491	ID_5
7days	l-wrist__Average_Height	-0.8274723	ID_6
7days	l-wrist__Average_Height	-1.107149	ID_7
7days	l-wrist__Average_Height	-0.7732756	ID_8
7days	l-wrist__Average_Height	-0.9342893	ID_9
14days	l-wrist__Average_Height	-0.3065015	ID_1
14days	l-wrist__Average_Height	-0.2901254	ID_2
14days	l-wrist__Average_Height	-0.7515281	ID_3
14days	l-wrist__Average_Height	-0.8692448	ID_4
14days	l-wrist__Average_Height	-0.6732098	ID_5
14days	l-wrist__Average_Height	-1.476333	ID_6
14days	l-wrist__Average_Height	-0.7906645	ID_7
14days	l-wrist__Average_Height	-0.8622238	ID_8
14days	l-wrist__Average_Height	-1.4346124	ID_9
14days	l-wrist__Average_Height	-1.0778146	ID_10
14days	l-wrist__Average_Height	-0.333383	ID_11
21days	l-wrist__Average_Height	-0.2890956	ID_1
21days	l-wrist__Average_Height	0.25069776	ID_2
21days	l-wrist__Average_Height	-0.0317026	ID_3
21days	l-wrist__Average_Height	-0.3461518	ID_4
21days	l-wrist__Average_Height	-0.3719616	ID_5
21days	l-wrist__Average_Height	-1.134017	ID_6
21days	l-wrist__Average_Height	-0.068622	ID_7
21days	l-wrist__Average_Height	-0.6158714	ID_8
21days	l-wrist__Average_Height	-0.6861222	ID_9
21days	l-wrist__Average_Height	-0.2163796	ID_10
0days	l-wrist__Movement	-0.0668485	ID_1

0days	l-wrist__Movement	-0.8233552	ID_2
0days	l-wrist__Movement	0.43410383	ID_3
0days	l-wrist__Movement	0.65900356	ID_4
0days	l-wrist__Movement	-0.7015359	ID_5
0days	l-wrist__Movement	1.0313634	ID_6
0days	l-wrist__Movement	0	ID_7
0days	l-wrist__Movement	-0.7123789	ID_8
0days	l-wrist__Movement	0.36823713	ID_9
0days	l-wrist__Movement	-0.3149964	ID_10
0days	l-wrist__Movement	-0.2060994	ID_11
0days	l-wrist__Movement	0.29965421	ID_12
0days	l-wrist__Movement	0.32131534	ID_13
3days	l-wrist__Movement	-1.5042083	ID_1
3days	l-wrist__Movement	-0.5815651	ID_2
3days	l-wrist__Movement	-0.5748475	ID_3
3days	l-wrist__Movement	-1.5834412	ID_4
3days	l-wrist__Movement	-2.1141262	ID_5
3days	l-wrist__Movement	-0.5199515	ID_6
3days	l-wrist__Movement	-0.7188211	ID_7
3days	l-wrist__Movement	-1.0053682	ID_8
7days	l-wrist__Movement	-0.040705	ID_1
7days	l-wrist__Movement	-1.3582428	ID_2
7days	l-wrist__Movement	-0.9271556	ID_3
7days	l-wrist__Movement	-1.6903592	ID_4
7days	l-wrist__Movement	-0.9693707	ID_5
7days	l-wrist__Movement	-0.876172	ID_6
7days	l-wrist__Movement	-1.0666631	ID_7
7days	l-wrist__Movement	-1.122757	ID_8
7days	l-wrist__Movement	-1.4632493	ID_9
14days	l-wrist__Movement	-0.026349	ID_1
14days	l-wrist__Movement	-0.5308143	ID_2
14days	l-wrist__Movement	-1.1437958	ID_3
14days	l-wrist__Movement	-1.1734528	ID_4
14days	l-wrist__Movement	-0.6209772	ID_5
14days	l-wrist__Movement	-1.5635549	ID_6
14days	l-wrist__Movement	-0.7988941	ID_7
14days	l-wrist__Movement	-0.8620086	ID_8
14days	l-wrist__Movement	-0.6140472	ID_9
14days	l-wrist__Movement	-0.8267626	ID_10
14days	l-wrist__Movement	0.43595011	ID_11
21days	l-wrist__Movement	-0.4633034	ID_1
21days	l-wrist__Movement	1.32532468	ID_2
21days	l-wrist__Movement	0.48366636	ID_3
21days	l-wrist__Movement	0.19644942	ID_4
21days	l-wrist__Movement	-0.7893995	ID_5
21days	l-wrist__Movement	-1.0992442	ID_6
21days	l-wrist__Movement	-0.0645873	ID_7
21days	l-wrist__Movement	0.23430611	ID_8
21days	l-wrist__Movement	-0.832874	ID_9
21days	l-wrist__Movement	0.31235869	ID_10
0days	r-back-ankle__Average_Heig	-0.1468829	ID_1
0days	r-back-ankle__Average_Heig	-0.0401207	ID_2
0days	r-back-ankle__Average_Heig	0.39735087	ID_3
0days	r-back-ankle__Average_Heig	0.37653903	ID_4
0days	r-back-ankle__Average_Heig	0.96955425	ID_5
0days	r-back-ankle__Average_Heig	0	ID_6

0days	r-back-ankle__Average_Heig	-0.0345592	ID_7
0days	r-back-ankle__Average_Heig	-0.5484057	ID_8
0days	r-back-ankle__Average_Heig	-0.1130645	ID_9
0days	r-back-ankle__Average_Heig	-0.1082404	ID_10
0days	r-back-ankle__Average_Heig	0.60831657	ID_11
0days	r-back-ankle__Average_Heig	0.58211529	ID_12
0days	r-back-ankle__Average_Heig	0.56271777	ID_13
3days	r-back-ankle__Average_Heig	0.47113181	ID_1
3days	r-back-ankle__Average_Heig	-0.2493458	ID_2
3days	r-back-ankle__Average_Heig	-0.1913517	ID_3
3days	r-back-ankle__Average_Heig	0.67381952	ID_4
3days	r-back-ankle__Average_Heig	1.23222732	ID_5
3days	r-back-ankle__Average_Heig	0.0424854	ID_6
3days	r-back-ankle__Average_Heig	0.98055707	ID_7
3days	r-back-ankle__Average_Heig	0.11957431	ID_8
7days	r-back-ankle__Average_Heig	0.07067285	ID_1
7days	r-back-ankle__Average_Heig	-0.1828693	ID_2
7days	r-back-ankle__Average_Heig	0.36533421	ID_3
7days	r-back-ankle__Average_Heig	0.46978811	ID_4
7days	r-back-ankle__Average_Heig	0.22741484	ID_5
7days	r-back-ankle__Average_Heig	0.96930615	ID_6
7days	r-back-ankle__Average_Heig	0.87412391	ID_7
7days	r-back-ankle__Average_Heig	0.09158385	ID_8
7days	r-back-ankle__Average_Heig	0.60109886	ID_9
14days	r-back-ankle__Average_Heig	-0.1909383	ID_1
14days	r-back-ankle__Average_Heig	0.47264935	ID_2
14days	r-back-ankle__Average_Heig	0.62618015	ID_3
14days	r-back-ankle__Average_Heig	0.70032853	ID_4
14days	r-back-ankle__Average_Heig	0.88070319	ID_5
14days	r-back-ankle__Average_Heig	0.82596298	ID_6
14days	r-back-ankle__Average_Heig	0.96959819	ID_7
14days	r-back-ankle__Average_Heig	0.86704915	ID_8
14days	r-back-ankle__Average_Heig	-0.2693921	ID_9
14days	r-back-ankle__Average_Heig	1.01156415	ID_10
14days	r-back-ankle__Average_Heig	-0.1367473	ID_11
21days	r-back-ankle__Average_Heig	-0.3918919	ID_1
21days	r-back-ankle__Average_Heig	0.53226777	ID_2
21days	r-back-ankle__Average_Heig	0.58103652	ID_3
21days	r-back-ankle__Average_Heig	1.05763863	ID_4
21days	r-back-ankle__Average_Heig	0.31793073	ID_5
21days	r-back-ankle__Average_Heig	0.85203847	ID_6
21days	r-back-ankle__Average_Heig	-0.1132625	ID_7
21days	r-back-ankle__Average_Heig	0.22886673	ID_8
21days	r-back-ankle__Average_Heig	0.59331043	ID_9
21days	r-back-ankle__Average_Heig	0.23823543	ID_10
0days	r-back-ankle__Movement	-0.53666	ID_1
0days	r-back-ankle__Movement	-0.2437109	ID_2
0days	r-back-ankle__Movement	0.12589186	ID_3
0days	r-back-ankle__Movement	-0.2556339	ID_4
0days	r-back-ankle__Movement	0.9582799	ID_5
0days	r-back-ankle__Movement	0.72373607	ID_6
0days	r-back-ankle__Movement	0.1745796	ID_7
0days	r-back-ankle__Movement	-1.7483562	ID_8
0days	r-back-ankle__Movement	-1.2817317	ID_9
0days	r-back-ankle__Movement	-0.5547742	ID_10
0days	r-back-ankle__Movement	0	ID_11

0days	r-back-ankle__Movement	0.78925078	ID_12
0days	r-back-ankle__Movement	0.77578419	ID_13
3days	r-back-ankle__Movement	-0.4562464	ID_1
3days	r-back-ankle__Movement	-1.2200183	ID_2
3days	r-back-ankle__Movement	-1.312673	ID_3
3days	r-back-ankle__Movement	-0.2749098	ID_4
3days	r-back-ankle__Movement	1.11911827	ID_5
3days	r-back-ankle__Movement	-0.2213618	ID_6
3days	r-back-ankle__Movement	1.81552971	ID_7
3days	r-back-ankle__Movement	0.43921921	ID_8
7days	r-back-ankle__Movement	-1.0519767	ID_1
7days	r-back-ankle__Movement	-0.5108035	ID_2
7days	r-back-ankle__Movement	-0.9018289	ID_3
7days	r-back-ankle__Movement	0.0665612	ID_4
7days	r-back-ankle__Movement	-0.6195518	ID_5
7days	r-back-ankle__Movement	2.01364032	ID_6
7days	r-back-ankle__Movement	0.83046006	ID_7
7days	r-back-ankle__Movement	0.05341691	ID_8
7days	r-back-ankle__Movement	0.67465299	ID_9
14days	r-back-ankle__Movement	-1.1212053	ID_1
14days	r-back-ankle__Movement	0.29950301	ID_2
14days	r-back-ankle__Movement	0.33130637	ID_3
14days	r-back-ankle__Movement	1.03161695	ID_4
14days	r-back-ankle__Movement	1.0908857	ID_5
14days	r-back-ankle__Movement	0.33265384	ID_6
14days	r-back-ankle__Movement	1.24265452	ID_7
14days	r-back-ankle__Movement	1.28354218	ID_8
14days	r-back-ankle__Movement	0.57465742	ID_9
14days	r-back-ankle__Movement	3.04106747	ID_10
14days	r-back-ankle__Movement	-0.2951909	ID_11
21days	r-back-ankle__Movement	-1.5107487	ID_1
21days	r-back-ankle__Movement	0.78949516	ID_2
21days	r-back-ankle__Movement	0.81447419	ID_3
21days	r-back-ankle__Movement	1.56851356	ID_4
21days	r-back-ankle__Movement	0.25379168	ID_5
21days	r-back-ankle__Movement	1.47900434	ID_6
21days	r-back-ankle__Movement	-1.0127802	ID_7
21days	r-back-ankle__Movement	0.68665494	ID_8
21days	r-back-ankle__Movement	0.91334627	ID_9
21days	r-back-ankle__Movement	0.51075904	ID_10
0days	r-back-toe__Average_Height	0.16428184	ID_1
0days	r-back-toe__Average_Height	0.23238301	ID_2
0days	r-back-toe__Average_Height	-0.205038	ID_3
0days	r-back-toe__Average_Height	-0.0667881	ID_4
0days	r-back-toe__Average_Height	-0.2077944	ID_5
0days	r-back-toe__Average_Height	0	ID_6
0days	r-back-toe__Average_Height	0.09773425	ID_7
0days	r-back-toe__Average_Height	0.10768269	ID_8
0days	r-back-toe__Average_Height	0.02231382	ID_9
0days	r-back-toe__Average_Height	0.07480318	ID_10
0days	r-back-toe__Average_Height	-0.2910118	ID_11
0days	r-back-toe__Average_Height	-0.0926191	ID_12
0days	r-back-toe__Average_Height	-0.0277035	ID_13
3days	r-back-toe__Average_Height	-0.1705249	ID_1
3days	r-back-toe__Average_Height	-0.2552123	ID_2
3days	r-back-toe__Average_Height	-0.0630304	ID_3

3days	r-back-toe__Average_Height	-0.2271536	ID_4
3days	r-back-toe__Average_Height	0.06989828	ID_5
3days	r-back-toe__Average_Height	-0.1739801	ID_6
3days	r-back-toe__Average_Height	0.1501916	ID_7
3days	r-back-toe__Average_Height	-0.0341578	ID_8
7days	r-back-toe__Average_Height	-0.0422763	ID_1
7days	r-back-toe__Average_Height	-0.0695279	ID_2
7days	r-back-toe__Average_Height	0.34405031	ID_3
7days	r-back-toe__Average_Height	0.74579805	ID_4
7days	r-back-toe__Average_Height	-0.037491	ID_5
7days	r-back-toe__Average_Height	0.41882362	ID_6
7days	r-back-toe__Average_Height	-0.0918367	ID_7
7days	r-back-toe__Average_Height	-0.0923621	ID_8
7days	r-back-toe__Average_Height	-0.2709577	ID_9
14days	r-back-toe__Average_Height	-0.2069974	ID_1
14days	r-back-toe__Average_Height	-0.132112	ID_2
14days	r-back-toe__Average_Height	0.14321988	ID_3
14days	r-back-toe__Average_Height	0.21929715	ID_4
14days	r-back-toe__Average_Height	0.31739203	ID_5
14days	r-back-toe__Average_Height	-0.0223594	ID_6
14days	r-back-toe__Average_Height	0.60258774	ID_7
14days	r-back-toe__Average_Height	0.77690214	ID_8
14days	r-back-toe__Average_Height	-0.0537482	ID_9
14days	r-back-toe__Average_Height	0.29723644	ID_10
14days	r-back-toe__Average_Height	-0.0556741	ID_11
21days	r-back-toe__Average_Height	-0.204243	ID_1
21days	r-back-toe__Average_Height	0.16089253	ID_2
21days	r-back-toe__Average_Height	0.07056671	ID_3
21days	r-back-toe__Average_Height	1.0563191	ID_4
21days	r-back-toe__Average_Height	-0.0126671	ID_5
21days	r-back-toe__Average_Height	0.13612435	ID_6
21days	r-back-toe__Average_Height	0.07347411	ID_7
21days	r-back-toe__Average_Height	0.60808882	ID_8
21days	r-back-toe__Average_Height	0.37612957	ID_9
21days	r-back-toe__Average_Height	0.43363305	ID_10
0days	r-back-toe__Movement	0.15088302	ID_1
0days	r-back-toe__Movement	0.84273158	ID_2
0days	r-back-toe__Movement	-0.5728224	ID_3
0days	r-back-toe__Movement	-0.8089688	ID_4
0days	r-back-toe__Movement	-0.2289414	ID_5
0days	r-back-toe__Movement	0.20414094	ID_6
0days	r-back-toe__Movement	0.01306226	ID_7
0days	r-back-toe__Movement	1.49536378	ID_8
0days	r-back-toe__Movement	-0.5327173	ID_9
0days	r-back-toe__Movement	-0.0958501	ID_10
0days	r-back-toe__Movement	-0.0451249	ID_11
0days	r-back-toe__Movement	0.47176219	ID_12
0days	r-back-toe__Movement	0	ID_13
3days	r-back-toe__Movement	-0.413253	ID_1
3days	r-back-toe__Movement	-0.4418956	ID_2
3days	r-back-toe__Movement	-0.4449753	ID_3
3days	r-back-toe__Movement	-0.2852193	ID_4
3days	r-back-toe__Movement	0.94427768	ID_5
3days	r-back-toe__Movement	-0.1405329	ID_6
3days	r-back-toe__Movement	1.1655564	ID_7
3days	r-back-toe__Movement	0.4619632	ID_8

7days	r-back-toe__Movement	0.64306093	ID_1
7days	r-back-toe__Movement	0.88709069	ID_2
7days	r-back-toe__Movement	0.57444742	ID_3
7days	r-back-toe__Movement	0.76843434	ID_4
7days	r-back-toe__Movement	-0.1996166	ID_5
7days	r-back-toe__Movement	1.23218581	ID_6
7days	r-back-toe__Movement	0.31819638	ID_7
7days	r-back-toe__Movement	-0.2934807	ID_8
7days	r-back-toe__Movement	-0.1789538	ID_9
14days	r-back-toe__Movement	-0.8820356	ID_1
14days	r-back-toe__Movement	-0.3334188	ID_2
14days	r-back-toe__Movement	1.73425966	ID_3
14days	r-back-toe__Movement	0.51693385	ID_4
14days	r-back-toe__Movement	0.87075354	ID_5
14days	r-back-toe__Movement	-0.1281288	ID_6
14days	r-back-toe__Movement	1.32247091	ID_7
14days	r-back-toe__Movement	1.41374384	ID_8
14days	r-back-toe__Movement	0.04532501	ID_9
14days	r-back-toe__Movement	1.00426211	ID_10
14days	r-back-toe__Movement	-0.5592575	ID_11
21days	r-back-toe__Movement	-0.1767319	ID_1
21days	r-back-toe__Movement	0.55448603	ID_2
21days	r-back-toe__Movement	0.33671286	ID_3
21days	r-back-toe__Movement	3.09688759	ID_4
21days	r-back-toe__Movement	0.07650652	ID_5
21days	r-back-toe__Movement	0.44406029	ID_6
21days	r-back-toe__Movement	-0.0751893	ID_7
21days	r-back-toe__Movement	0.99516644	ID_8
21days	r-back-toe__Movement	0.6239984	ID_9
21days	r-back-toe__Movement	1.74329356	ID_10
0days	r-elbow__Average_Height	0.00362726	ID_1
0days	r-elbow__Average_Height	-0.4731648	ID_2
0days	r-elbow__Average_Height	0	ID_3
0days	r-elbow__Average_Height	0.59821548	ID_4
0days	r-elbow__Average_Height	-0.1795274	ID_5
0days	r-elbow__Average_Height	-0.2091248	ID_6
0days	r-elbow__Average_Height	0.06555415	ID_7
0days	r-elbow__Average_Height	0.0028623	ID_8
0days	r-elbow__Average_Height	0.00705776	ID_9
0days	r-elbow__Average_Height	-0.3061086	ID_10
0days	r-elbow__Average_Height	-0.3496225	ID_11
0days	r-elbow__Average_Height	-0.2138599	ID_12
0days	r-elbow__Average_Height	0.15087725	ID_13
3days	r-elbow__Average_Height	-0.4602182	ID_1
3days	r-elbow__Average_Height	-0.44453	ID_2
3days	r-elbow__Average_Height	-0.4661707	ID_3
3days	r-elbow__Average_Height	-0.5317932	ID_4
3days	r-elbow__Average_Height	-0.6897216	ID_5
3days	r-elbow__Average_Height	-0.4917161	ID_6
3days	r-elbow__Average_Height	0.08653793	ID_7
3days	r-elbow__Average_Height	-0.0810403	ID_8
7days	r-elbow__Average_Height	-0.5885457	ID_1
7days	r-elbow__Average_Height	-0.3345832	ID_2
7days	r-elbow__Average_Height	-0.38448	ID_3
7days	r-elbow__Average_Height	-0.0576795	ID_4
7days	r-elbow__Average_Height	-0.0200633	ID_5

7days	r-elbow__Average_Height	-0.3382561	ID_6
7days	r-elbow__Average_Height	-0.1930686	ID_7
7days	r-elbow__Average_Height	-0.1289549	ID_8
7days	r-elbow__Average_Height	-0.269897	ID_9
14days	r-elbow__Average_Height	-0.2779463	ID_1
14days	r-elbow__Average_Height	0.04722786	ID_2
14days	r-elbow__Average_Height	-0.4490856	ID_3
14days	r-elbow__Average_Height	-0.1542237	ID_4
14days	r-elbow__Average_Height	-0.3414627	ID_5
14days	r-elbow__Average_Height	-0.3675006	ID_6
14days	r-elbow__Average_Height	-0.7401342	ID_7
14days	r-elbow__Average_Height	-0.168529	ID_8
14days	r-elbow__Average_Height	-0.272153	ID_9
14days	r-elbow__Average_Height	-0.2597085	ID_10
21days	r-elbow__Average_Height	-0.3408939	ID_11
21days	r-elbow__Average_Height	0.31149782	ID_1
21days	r-elbow__Average_Height	-0.2743563	ID_2
21days	r-elbow__Average_Height	-0.252446	ID_3
21days	r-elbow__Average_Height	0.06826335	ID_4
21days	r-elbow__Average_Height	-0.5913508	ID_5
21days	r-elbow__Average_Height	-0.0757413	ID_6
21days	r-elbow__Average_Height	0.08629657	ID_7
21days	r-elbow__Average_Height	-0.0771913	ID_8
21days	r-elbow__Average_Height	0.63612734	ID_9
0days	r-elbow__Movement	-0.1548557	ID_10
0days	r-elbow__Movement	-0.9641164	ID_1
0days	r-elbow__Movement	0.02450275	ID_2
0days	r-elbow__Movement	0.57259644	ID_3
0days	r-elbow__Movement	-0.1796794	ID_4
0days	r-elbow__Movement	-0.7718495	ID_5
0days	r-elbow__Movement	0.36785776	ID_6
0days	r-elbow__Movement	0	ID_7
0days	r-elbow__Movement	0.37572922	ID_8
0days	r-elbow__Movement	-1.1822798	ID_9
0days	r-elbow__Movement	-0.8096325	ID_10
0days	r-elbow__Movement	0.11151942	ID_11
0days	r-elbow__Movement	0.46812633	ID_12
3days	r-elbow__Movement	-0.9704	ID_13
3days	r-elbow__Movement	-1.2574547	ID_1
3days	r-elbow__Movement	-0.8054357	ID_2
3days	r-elbow__Movement	-1.0453943	ID_3
3days	r-elbow__Movement	-1.4619121	ID_4
3days	r-elbow__Movement	-0.9935279	ID_5
3days	r-elbow__Movement	-0.1506226	ID_6
3days	r-elbow__Movement	0.06173137	ID_7
7days	r-elbow__Movement	-1.4591558	ID_8
7days	r-elbow__Movement	-0.7326618	ID_1
7days	r-elbow__Movement	-1.0562758	ID_2
7days	r-elbow__Movement	-0.9081111	ID_3
7days	r-elbow__Movement	-0.7497038	ID_4
7days	r-elbow__Movement	-0.9367159	ID_5
7days	r-elbow__Movement	-0.8367696	ID_6
7days	r-elbow__Movement	-0.9526068	ID_7
7days	r-elbow__Movement	-0.9394047	ID_8
14days	r-elbow__Movement	-0.6687055	ID_9
14days	r-elbow__Movement	-0.454514	ID_1

14days	r-elbow__Movement	-1.1927558	ID_2
14days	r-elbow__Movement	-0.4436888	ID_3
14days	r-elbow__Movement	-0.957033	ID_4
14days	r-elbow__Movement	-0.7357572	ID_5
14days	r-elbow__Movement	-1.4834283	ID_6
14days	r-elbow__Movement	0.23674366	ID_7
14days	r-elbow__Movement	-0.7070423	ID_8
14days	r-elbow__Movement	-0.4223416	ID_9
21days	r-elbow__Movement	-0.8425795	ID_10
21days	r-elbow__Movement	0.29334309	ID_11
21days	r-elbow__Movement	-0.3426718	ID_1
21days	r-elbow__Movement	-0.4123859	ID_2
21days	r-elbow__Movement	-0.5608952	ID_3
21days	r-elbow__Movement	-1.4820006	ID_4
21days	r-elbow__Movement	-0.465817	ID_5
21days	r-elbow__Movement	-0.3569001	ID_6
21days	r-elbow__Movement	-0.7083302	ID_7
21days	r-elbow__Movement	1.64210311	ID_8
0days	r-front-toe-tip__Average_Hei	0.03135791	ID_1
0days	r-front-toe-tip__Average_Hei	0.18709357	ID_2
0days	r-front-toe-tip__Average_Hei	0.1476945	ID_3
0days	r-front-toe-tip__Average_Hei	-0.001675	ID_4
0days	r-front-toe-tip__Average_Hei	-0.1703256	ID_5
0days	r-front-toe-tip__Average_Hei	0.00514434	ID_6
0days	r-front-toe-tip__Average_Hei	0	ID_7
0days	r-front-toe-tip__Average_Hei	0.57169011	ID_8
0days	r-front-toe-tip__Average_Hei	-0.0052955	ID_9
0days	r-front-toe-tip__Average_Hei	-0.2480867	ID_10
0days	r-front-toe-tip__Average_Hei	-0.1338386	ID_11
0days	r-front-toe-tip__Average_Hei	-0.1716836	ID_12
0days	r-front-toe-tip__Average_Hei	0.20355628	ID_13
3days	r-front-toe-tip__Average_Hei	-0.2158472	ID_1
3days	r-front-toe-tip__Average_Hei	-0.3191591	ID_2
3days	r-front-toe-tip__Average_Hei	-0.0324925	ID_3
3days	r-front-toe-tip__Average_Hei	-0.2357843	ID_4
3days	r-front-toe-tip__Average_Hei	-0.1801322	ID_5
3days	r-front-toe-tip__Average_Hei	-0.3621174	ID_6
3days	r-front-toe-tip__Average_Hei	-0.3246147	ID_7
3days	r-front-toe-tip__Average_Hei	0.12807963	ID_8
7days	r-front-toe-tip__Average_Hei	-0.0384315	ID_1
7days	r-front-toe-tip__Average_Hei	0.40600335	ID_2
7days	r-front-toe-tip__Average_Hei	-0.3368922	ID_3
7days	r-front-toe-tip__Average_Hei	0.25196417	ID_4
7days	r-front-toe-tip__Average_Hei	-0.3000916	ID_5
7days	r-front-toe-tip__Average_Hei	0.27875009	ID_6
7days	r-front-toe-tip__Average_Hei	-0.2231241	ID_7
7days	r-front-toe-tip__Average_Hei	-0.3058005	ID_8
7days	r-front-toe-tip__Average_Hei	-0.1284495	ID_9
14days	r-front-toe-tip__Average_Hei	0.17720183	ID_1
14days	r-front-toe-tip__Average_Hei	-0.4238257	ID_2
14days	r-front-toe-tip__Average_Hei	0.03896691	ID_3
14days	r-front-toe-tip__Average_Hei	0.18950152	ID_4
14days	r-front-toe-tip__Average_Hei	0.17184373	ID_5
14days	r-front-toe-tip__Average_Hei	-0.4353274	ID_6
14days	r-front-toe-tip__Average_Hei	0.1754423	ID_7
14days	r-front-toe-tip__Average_Hei	0.2353217	ID_8

14days	r-front-toe-tip__Average_Hei	-0.3142367	ID_9
14days	r-front-toe-tip__Average_Hei	-0.0381014	ID_10
14days	r-front-toe-tip__Average_Hei	-0.1564677	ID_11
21days	r-front-toe-tip__Average_Hei	-0.2037808	ID_1
21days	r-front-toe-tip__Average_Hei	-0.3479303	ID_2
21days	r-front-toe-tip__Average_Hei	-0.3511617	ID_3
21days	r-front-toe-tip__Average_Hei	0.43123269	ID_4
21days	r-front-toe-tip__Average_Hei	-0.5066782	ID_5
21days	r-front-toe-tip__Average_Hei	0.24663604	ID_6
21days	r-front-toe-tip__Average_Hei	-0.1644824	ID_7
21days	r-front-toe-tip__Average_Hei	0.27898279	ID_8
21days	r-front-toe-tip__Average_Hei	0.19914095	ID_9
21days	r-front-toe-tip__Average_Hei	0.12763178	ID_10
0days	r-front-toe-tip__Movement	0.38925413	ID_1
0days	r-front-toe-tip__Movement	0	ID_2
0days	r-front-toe-tip__Movement	-0.012741	ID_3
0days	r-front-toe-tip__Movement	-0.0011338	ID_4
0days	r-front-toe-tip__Movement	0.09993967	ID_5
0days	r-front-toe-tip__Movement	0.29299115	ID_6
0days	r-front-toe-tip__Movement	-0.0045303	ID_7
0days	r-front-toe-tip__Movement	1.39466025	ID_8
0days	r-front-toe-tip__Movement	-0.5692494	ID_9
0days	r-front-toe-tip__Movement	-0.5764126	ID_10
0days	r-front-toe-tip__Movement	0.27938092	ID_11
0days	r-front-toe-tip__Movement	-0.4648929	ID_12
0days	r-front-toe-tip__Movement	0.25553153	ID_13
3days	r-front-toe-tip__Movement	-0.4652572	ID_1
3days	r-front-toe-tip__Movement	0.01358452	ID_2
3days	r-front-toe-tip__Movement	-0.3115956	ID_3
3days	r-front-toe-tip__Movement	-0.1114153	ID_4
3days	r-front-toe-tip__Movement	-0.0541393	ID_5
3days	r-front-toe-tip__Movement	-0.1504963	ID_6
3days	r-front-toe-tip__Movement	-0.5226726	ID_7
3days	r-front-toe-tip__Movement	0.10113918	ID_8
7days	r-front-toe-tip__Movement	0.29063786	ID_1
7days	r-front-toe-tip__Movement	0.83210408	ID_2
7days	r-front-toe-tip__Movement	-0.5529492	ID_3
7days	r-front-toe-tip__Movement	0.46573136	ID_4
7days	r-front-toe-tip__Movement	-0.4383184	ID_5
7days	r-front-toe-tip__Movement	0.58447269	ID_6
7days	r-front-toe-tip__Movement	-0.727144	ID_7
7days	r-front-toe-tip__Movement	-0.1118996	ID_8
7days	r-front-toe-tip__Movement	-0.2620417	ID_9
14days	r-front-toe-tip__Movement	0.4401529	ID_1
14days	r-front-toe-tip__Movement	-0.146346	ID_2
14days	r-front-toe-tip__Movement	-0.0910604	ID_3
14days	r-front-toe-tip__Movement	0.62465954	ID_4
14days	r-front-toe-tip__Movement	0.27596688	ID_5
14days	r-front-toe-tip__Movement	-1.1249305	ID_6
14days	r-front-toe-tip__Movement	0.6489982	ID_7
14days	r-front-toe-tip__Movement	0.56490184	ID_8
14days	r-front-toe-tip__Movement	-0.6545676	ID_9
14days	r-front-toe-tip__Movement	0.19486483	ID_10
14days	r-front-toe-tip__Movement	-0.629778	ID_11
21days	r-front-toe-tip__Movement	-0.065203	ID_1
21days	r-front-toe-tip__Movement	-0.3671034	ID_2

21days	r-front-toe-tip__Movement	-0.3778014	ID_3
21days	r-front-toe-tip__Movement	0.93774655	ID_4
21days	r-front-toe-tip__Movement	-1.1437734	ID_5
21days	r-front-toe-tip__Movement	0.38580527	ID_6
21days	r-front-toe-tip__Movement	-0.2891268	ID_7
21days	r-front-toe-tip__Movement	0.15692873	ID_8
21days	r-front-toe-tip__Movement	0.07725664	ID_9
21days	r-front-toe-tip__Movement	0.29202303	ID_10
0days	r-hip__Average_Height	-0.4497609	ID_1
0days	r-hip__Average_Height	-0.059288	ID_2
0days	r-hip__Average_Height	0	ID_3
0days	r-hip__Average_Height	0.46250577	ID_4
0days	r-hip__Average_Height	-0.2079616	ID_5
0days	r-hip__Average_Height	0.01601584	ID_6
0days	r-hip__Average_Height	0.0162802	ID_7
0days	r-hip__Average_Height	0.19679759	ID_8
0days	r-hip__Average_Height	-0.3757805	ID_9
0days	r-hip__Average_Height	0.02052768	ID_10
0days	r-hip__Average_Height	-0.1778465	ID_11
0days	r-hip__Average_Height	0.40169812	ID_12
0days	r-hip__Average_Height	-0.3324927	ID_13
3days	r-hip__Average_Height	-0.4157003	ID_1
3days	r-hip__Average_Height	-0.4197327	ID_2
3days	r-hip__Average_Height	-0.2307823	ID_3
3days	r-hip__Average_Height	-0.4071027	ID_4
3days	r-hip__Average_Height	-0.2016535	ID_5
3days	r-hip__Average_Height	-0.4136191	ID_6
3days	r-hip__Average_Height	-0.0399806	ID_7
3days	r-hip__Average_Height	0.3522125	ID_8
7days	r-hip__Average_Height	0.01960609	ID_1
7days	r-hip__Average_Height	-0.2428318	ID_2
7days	r-hip__Average_Height	-0.0476098	ID_3
7days	r-hip__Average_Height	0.14324227	ID_4
7days	r-hip__Average_Height	-0.0017086	ID_5
7days	r-hip__Average_Height	-0.1109276	ID_6
7days	r-hip__Average_Height	-0.411578	ID_7
7days	r-hip__Average_Height	-0.190413	ID_8
7days	r-hip__Average_Height	0.22695951	ID_9
14days	r-hip__Average_Height	0.2049254	ID_1
14days	r-hip__Average_Height	-0.0463459	ID_2
14days	r-hip__Average_Height	-0.1501316	ID_3
14days	r-hip__Average_Height	-0.1642908	ID_4
14days	r-hip__Average_Height	0.13371959	ID_5
14days	r-hip__Average_Height	0.217187	ID_6
14days	r-hip__Average_Height	-0.5425727	ID_7
14days	r-hip__Average_Height	-0.522632	ID_8
14days	r-hip__Average_Height	0.22694619	ID_9
14days	r-hip__Average_Height	0.00571137	ID_10
14days	r-hip__Average_Height	-0.0915817	ID_11
21days	r-hip__Average_Height	-0.0990266	ID_1
21days	r-hip__Average_Height	-0.5339945	ID_2
21days	r-hip__Average_Height	-0.4061764	ID_3
21days	r-hip__Average_Height	0.40818297	ID_4
21days	r-hip__Average_Height	-0.1786207	ID_5
21days	r-hip__Average_Height	-0.1757608	ID_6
21days	r-hip__Average_Height	0.24357971	ID_7

21days	r-hip__Average_Height	0.59612176	ID_8
21days	r-hip__Average_Height	-0.0116845	ID_9
21days	r-hip__Average_Height	-0.112731	ID_10
0days	r-hip__Movement	-0.5543281	ID_1
0days	r-hip__Movement	0.97697186	ID_2
0days	r-hip__Movement	0.46183821	ID_3
0days	r-hip__Movement	1.71775673	ID_4
0days	r-hip__Movement	-0.4879813	ID_5
0days	r-hip__Movement	0	ID_6
0days	r-hip__Movement	-0.3338742	ID_7
0days	r-hip__Movement	1.13570277	ID_8
0days	r-hip__Movement	-0.8249045	ID_9
0days	r-hip__Movement	-0.1916942	ID_10
0days	r-hip__Movement	-0.0039839	ID_11
0days	r-hip__Movement	0.26954998	ID_12
0days	r-hip__Movement	0.00253249	ID_13
3days	r-hip__Movement	-0.8315936	ID_1
3days	r-hip__Movement	-0.599086	ID_2
3days	r-hip__Movement	-0.317279	ID_3
3days	r-hip__Movement	-0.7174234	ID_4
3days	r-hip__Movement	-0.5445019	ID_5
3days	r-hip__Movement	-0.630397	ID_6
3days	r-hip__Movement	-0.0882008	ID_7
3days	r-hip__Movement	1.62112633	ID_8
7days	r-hip__Movement	0.13201038	ID_1
7days	r-hip__Movement	-0.076238	ID_2
7days	r-hip__Movement	-0.5621799	ID_3
7days	r-hip__Movement	0.44731972	ID_4
7days	r-hip__Movement	-0.2004937	ID_5
7days	r-hip__Movement	0.38042612	ID_6
7days	r-hip__Movement	-0.6785643	ID_7
7days	r-hip__Movement	-0.0853527	ID_8
7days	r-hip__Movement	0.52051755	ID_9
14days	r-hip__Movement	0.11939104	ID_1
14days	r-hip__Movement	-0.0290794	ID_2
14days	r-hip__Movement	0.71295079	ID_3
14days	r-hip__Movement	-0.3349939	ID_4
14days	r-hip__Movement	0.37541754	ID_5
14days	r-hip__Movement	0.08961341	ID_6
14days	r-hip__Movement	-0.9189982	ID_7
14days	r-hip__Movement	-0.7602581	ID_8
14days	r-hip__Movement	0.17726289	ID_9
14days	r-hip__Movement	0.82457658	ID_10
14days	r-hip__Movement	-0.5056043	ID_11
21days	r-hip__Movement	-0.0628528	ID_1
21days	r-hip__Movement	-1.0330445	ID_2
21days	r-hip__Movement	-0.2517236	ID_3
21days	r-hip__Movement	1.2426687	ID_4
21days	r-hip__Movement	-0.6463288	ID_5
21days	r-hip__Movement	0.46505367	ID_6
21days	r-hip__Movement	0.26521156	ID_7
21days	r-hip__Movement	1.03866696	ID_8
21days	r-hip__Movement	-0.1559217	ID_9
21days	r-hip__Movement	-0.2562772	ID_10
0days	r-iliac-crest__Average_Heigh	-0.2505019	ID_1
0days	r-iliac-crest__Average_Heigh	0.31183046	ID_2

0days	r-iliac-crest__Average_Heigh	0.16535694	ID_3
0days	r-iliac-crest__Average_Heigh	0.504612	ID_4
0days	r-iliac-crest__Average_Heigh	0	ID_5
0days	r-iliac-crest__Average_Heigh	0.22236175	ID_6
0days	r-iliac-crest__Average_Heigh	-0.0271328	ID_7
0days	r-iliac-crest__Average_Heigh	0.44566443	ID_8
0days	r-iliac-crest__Average_Heigh	-0.1167121	ID_9
0days	r-iliac-crest__Average_Heigh	0.12722814	ID_10
0days	r-iliac-crest__Average_Heigh	-0.1694924	ID_11
0days	r-iliac-crest__Average_Heigh	-0.0818991	ID_12
0days	r-iliac-crest__Average_Heigh	-0.1263372	ID_13
3days	r-iliac-crest__Average_Heigh	-0.2488331	ID_1
3days	r-iliac-crest__Average_Heigh	-0.0575799	ID_2
3days	r-iliac-crest__Average_Heigh	-0.1217178	ID_3
3days	r-iliac-crest__Average_Heigh	-0.1231048	ID_4
3days	r-iliac-crest__Average_Heigh	-0.028291	ID_5
3days	r-iliac-crest__Average_Heigh	-0.1996952	ID_6
3days	r-iliac-crest__Average_Heigh	0.20389234	ID_7
3days	r-iliac-crest__Average_Heigh	0.29356764	ID_8
7days	r-iliac-crest__Average_Heigh	-0.0558289	ID_1
7days	r-iliac-crest__Average_Heigh	0.11319651	ID_2
7days	r-iliac-crest__Average_Heigh	0.03684093	ID_3
7days	r-iliac-crest__Average_Heigh	0.30489547	ID_4
7days	r-iliac-crest__Average_Heigh	0.03775729	ID_5
7days	r-iliac-crest__Average_Heigh	0.52473411	ID_6
7days	r-iliac-crest__Average_Heigh	0.69938113	ID_7
7days	r-iliac-crest__Average_Heigh	-0.2851213	ID_8
7days	r-iliac-crest__Average_Heigh	-0.3502932	ID_9
14days	r-iliac-crest__Average_Heigh	0.23703158	ID_1
14days	r-iliac-crest__Average_Heigh	0.13260314	ID_2
14days	r-iliac-crest__Average_Heigh	0.40341246	ID_3
14days	r-iliac-crest__Average_Heigh	0.12627804	ID_4
14days	r-iliac-crest__Average_Heigh	0.6876098	ID_5
14days	r-iliac-crest__Average_Heigh	-0.011419	ID_6
14days	r-iliac-crest__Average_Heigh	-0.4675634	ID_7
14days	r-iliac-crest__Average_Heigh	-0.4001553	ID_8
14days	r-iliac-crest__Average_Heigh	0.39091757	ID_9
14days	r-iliac-crest__Average_Heigh	0.18609424	ID_10
14days	r-iliac-crest__Average_Heigh	0.04278494	ID_11
21days	r-iliac-crest__Average_Heigh	-0.2106064	ID_1
21days	r-iliac-crest__Average_Heigh	-0.2841178	ID_2
21days	r-iliac-crest__Average_Heigh	-0.3105821	ID_3
21days	r-iliac-crest__Average_Heigh	0.76170517	ID_4
21days	r-iliac-crest__Average_Heigh	-0.2154079	ID_5
21days	r-iliac-crest__Average_Heigh	0.39869757	ID_6
21days	r-iliac-crest__Average_Heigh	-0.1317501	ID_7
21days	r-iliac-crest__Average_Heigh	0.4514136	ID_8
21days	r-iliac-crest__Average_Heigh	0.43861571	ID_9
21days	r-iliac-crest__Average_Heigh	0.15495561	ID_10
0days	r-iliac-crest__Movement	-0.5478296	ID_1
0days	r-iliac-crest__Movement	1.2688499	ID_2
0days	r-iliac-crest__Movement	0.37669238	ID_3
0days	r-iliac-crest__Movement	1.17802958	ID_4
0days	r-iliac-crest__Movement	-0.0773928	ID_5
0days	r-iliac-crest__Movement	0.00950445	ID_6
0days	r-iliac-crest__Movement	-0.0847591	ID_7

0days	r-iliac-crest__Movement	1.91939243	ID_8
0days	r-iliac-crest__Movement	-0.2848737	ID_9
0days	r-iliac-crest__Movement	0	ID_10
0days	r-iliac-crest__Movement	0.69444858	ID_11
0days	r-iliac-crest__Movement	-0.2142627	ID_12
0days	r-iliac-crest__Movement	-0.6708985	ID_13
3days	r-iliac-crest__Movement	-0.67236	ID_1
3days	r-iliac-crest__Movement	-0.3112036	ID_2
3days	r-iliac-crest__Movement	-0.5177308	ID_3
3days	r-iliac-crest__Movement	-0.3426203	ID_4
3days	r-iliac-crest__Movement	-0.4293133	ID_5
3days	r-iliac-crest__Movement	-0.3657583	ID_6
3days	r-iliac-crest__Movement	0.46783034	ID_7
3days	r-iliac-crest__Movement	0.92146101	ID_8
7days	r-iliac-crest__Movement	-0.6382622	ID_1
7days	r-iliac-crest__Movement	0.51034028	ID_2
7days	r-iliac-crest__Movement	-0.1402113	ID_3
7days	r-iliac-crest__Movement	0.95080326	ID_4
7days	r-iliac-crest__Movement	-0.1795549	ID_5
7days	r-iliac-crest__Movement	1.4135252	ID_6
7days	r-iliac-crest__Movement	1.12129972	ID_7
7days	r-iliac-crest__Movement	-0.4370683	ID_8
7days	r-iliac-crest__Movement	-0.4894146	ID_9
14days	r-iliac-crest__Movement	0.36710025	ID_1
14days	r-iliac-crest__Movement	0.21510089	ID_2
14days	r-iliac-crest__Movement	0.79584784	ID_3
14days	r-iliac-crest__Movement	0.39869722	ID_4
14days	r-iliac-crest__Movement	1.67559451	ID_5
14days	r-iliac-crest__Movement	0.06223912	ID_6
14days	r-iliac-crest__Movement	-0.6342515	ID_7
14days	r-iliac-crest__Movement	-0.4390243	ID_8
14days	r-iliac-crest__Movement	0.50996829	ID_9
14days	r-iliac-crest__Movement	1.15494125	ID_10
14days	r-iliac-crest__Movement	0.56894137	ID_11
21days	r-iliac-crest__Movement	-0.6107009	ID_1
21days	r-iliac-crest__Movement	-0.1710312	ID_2
21days	r-iliac-crest__Movement	-0.4965877	ID_3
21days	r-iliac-crest__Movement	1.31765594	ID_4
21days	r-iliac-crest__Movement	-0.4556228	ID_5
21days	r-iliac-crest__Movement	1.33111391	ID_6
21days	r-iliac-crest__Movement	-0.4287663	ID_7
21days	r-iliac-crest__Movement	0.95618634	ID_8
21days	r-iliac-crest__Movement	0.77571354	ID_9
21days	r-iliac-crest__Movement	-0.0321817	ID_10
0days	r-shoulder__Average_Height	-0.0410086	ID_1
0days	r-shoulder__Average_Height	-0.1994091	ID_2
0days	r-shoulder__Average_Height	0.00507822	ID_3
0days	r-shoulder__Average_Height	0.65070218	ID_4
0days	r-shoulder__Average_Height	0	ID_5
0days	r-shoulder__Average_Height	0.03556996	ID_6
0days	r-shoulder__Average_Height	-0.161415	ID_7
0days	r-shoulder__Average_Height	0.78605563	ID_8
0days	r-shoulder__Average_Height	0.145472	ID_9
0days	r-shoulder__Average_Height	-0.1727972	ID_10
0days	r-shoulder__Average_Height	-0.001637	ID_11
0days	r-shoulder__Average_Height	-0.0847022	ID_12

0days	r-shoulder__Average_Height	0.02663445	ID_13
3days	r-shoulder__Average_Height	-0.3224065	ID_1
3days	r-shoulder__Average_Height	-0.039671	ID_2
3days	r-shoulder__Average_Height	0.33247339	ID_3
7days	r-shoulder__Average_Height	-0.0463295	ID_1
7days	r-shoulder__Average_Height	0.11004258	ID_2
7days	r-shoulder__Average_Height	-0.0734568	ID_3
7days	r-shoulder__Average_Height	-0.101915	ID_4
7days	r-shoulder__Average_Height	-0.1659842	ID_5
7days	r-shoulder__Average_Height	-0.1339037	ID_6
7days	r-shoulder__Average_Height	-0.031272	ID_7
7days	r-shoulder__Average_Height	-0.2939886	ID_8
14days	r-shoulder__Average_Height	-0.1375322	ID_1
14days	r-shoulder__Average_Height	-0.1722596	ID_2
14days	r-shoulder__Average_Height	0.00811027	ID_3
14days	r-shoulder__Average_Height	-0.033674	ID_4
14days	r-shoulder__Average_Height	-0.4199987	ID_5
14days	r-shoulder__Average_Height	-0.016972	ID_6
14days	r-shoulder__Average_Height	0.03909872	ID_7
14days	r-shoulder__Average_Height	0.09131395	ID_8
14days	r-shoulder__Average_Height	0.18869352	ID_9
21days	r-shoulder__Average_Height	-0.1975349	ID_1
21days	r-shoulder__Average_Height	-0.2335856	ID_2
21days	r-shoulder__Average_Height	-0.2111115	ID_3
21days	r-shoulder__Average_Height	-0.0205237	ID_4
21days	r-shoulder__Average_Height	-0.2132415	ID_5
21days	r-shoulder__Average_Height	0.42863289	ID_6
21days	r-shoulder__Average_Height	-0.2062587	ID_7
21days	r-shoulder__Average_Height	-0.1528395	ID_8
0days	r-shoulder__Movement	0.28274637	ID_1
0days	r-shoulder__Movement	-0.5001339	ID_2
0days	r-shoulder__Movement	0.47819926	ID_3
0days	r-shoulder__Movement	1.48064408	ID_4
0days	r-shoulder__Movement	0.36421513	ID_5
0days	r-shoulder__Movement	0.37497867	ID_6
0days	r-shoulder__Movement	-0.0597733	ID_7
0days	r-shoulder__Movement	1.38267606	ID_8
0days	r-shoulder__Movement	0	ID_9
0days	r-shoulder__Movement	-0.2915515	ID_10
0days	r-shoulder__Movement	-0.2088125	ID_11
0days	r-shoulder__Movement	-0.3701008	ID_12
0days	r-shoulder__Movement	-0.1323511	ID_13
3days	r-shoulder__Movement	-0.7424135	ID_1
3days	r-shoulder__Movement	-0.1246607	ID_2
3days	r-shoulder__Movement	0.36773151	ID_3
7days	r-shoulder__Movement	-0.5371689	ID_4
7days	r-shoulder__Movement	0.04017472	ID_5
7days	r-shoulder__Movement	-0.5611534	ID_6
7days	r-shoulder__Movement	-0.4302203	ID_7
7days	r-shoulder__Movement	-0.4650271	ID_8
7days	r-shoulder__Movement	-0.3308973	ID_1
7days	r-shoulder__Movement	-0.3384382	ID_2
7days	r-shoulder__Movement	-0.2716098	ID_3
14days	r-shoulder__Movement	-0.3166392	ID_4
14days	r-shoulder__Movement	-0.4847344	ID_5
14days	r-shoulder__Movement	0.03914954	ID_6

14days	r-shoulder__Movement	0.53511622	ID_7
14days	r-shoulder__Movement	-0.7019067	ID_8
14days	r-shoulder__Movement	-0.4604283	ID_9
14days	r-shoulder__Movement	-0.032709	ID_1
14days	r-shoulder__Movement	0.27185114	ID_2
14days	r-shoulder__Movement	-0.1576145	ID_3
21days	r-shoulder__Movement	-0.6614872	ID_4
21days	r-shoulder__Movement	-0.4029086	ID_5
21days	r-shoulder__Movement	-0.6768168	ID_6
21days	r-shoulder__Movement	0.09110983	ID_7
21days	r-shoulder__Movement	-0.3405079	ID_8
21days	r-shoulder__Movement	0.2599305	ID_9
21days	r-shoulder__Movement	-0.3577997	ID_10
21days	r-shoulder__Movement	-0.4206061	ID_11
0days	r-tail-base__Average_Height	-0.3279288	ID_1
0days	r-tail-base__Average_Height	0.18965672	ID_2
0days	r-tail-base__Average_Height	-0.1291052	ID_3
0days	r-tail-base__Average_Height	-0.1928172	ID_4
0days	r-tail-base__Average_Height	0.51735276	ID_5
0days	r-tail-base__Average_Height	1.24942018	ID_6
0days	r-tail-base__Average_Height	-0.2173085	ID_7
0days	r-tail-base__Average_Height	-0.2346401	ID_8
0days	r-tail-base__Average_Height	-0.7510641	ID_9
0days	r-tail-base__Average_Height	0.12910519	ID_10
0days	r-tail-base__Average_Height	0.91701353	ID_11
0days	r-tail-base__Average_Height	0.62503555	ID_12
3days	r-tail-base__Average_Height	-0.0605289	ID_13
3days	r-tail-base__Average_Height	-0.0085418	ID_1
3days	r-tail-base__Average_Height	-0.1436656	ID_2
3days	r-tail-base__Average_Height	-0.0564892	ID_3
3days	r-tail-base__Average_Height	-0.5335647	ID_4
3days	r-tail-base__Average_Height	-0.3954393	ID_5
3days	r-tail-base__Average_Height	0.6222008	ID_6
3days	r-tail-base__Average_Height	0.44764986	ID_7
7days	r-tail-base__Average_Height	0.48413936	ID_8
7days	r-tail-base__Average_Height	-0.7542402	ID_1
7days	r-tail-base__Average_Height	-0.6463946	ID_2
7days	r-tail-base__Average_Height	-0.4015599	ID_3
7days	r-tail-base__Average_Height	-0.0265957	ID_4
7days	r-tail-base__Average_Height	-0.2390397	ID_5
7days	r-tail-base__Average_Height	0.23780745	ID_6
7days	r-tail-base__Average_Height	-0.3404965	ID_7
7days	r-tail-base__Average_Height	-0.2709525	ID_8
14days	r-tail-base__Average_Height	0.16090946	ID_9
14days	r-tail-base__Average_Height	-0.2433202	ID_1
14days	r-tail-base__Average_Height	-0.1217874	ID_2
14days	r-tail-base__Average_Height	-0.404408	ID_3
14days	r-tail-base__Average_Height	-0.1276667	ID_4
14days	r-tail-base__Average_Height	0.48894002	ID_5
14days	r-tail-base__Average_Height	-0.5521597	ID_6
14days	r-tail-base__Average_Height	-0.6625228	ID_7
14days	r-tail-base__Average_Height	0.42708776	ID_8
14days	r-tail-base__Average_Height	0.52358889	ID_9
14days	r-tail-base__Average_Height	-0.5269455	ID_10
21days	r-tail-base__Average_Height	-0.1884373	ID_11
21days	r-tail-base__Average_Height	-0.3999281	ID_1

21days	r-tail-base__Average_Height	-0.1141487	ID_2
21days	r-tail-base__Average_Height	-0.3506993	ID_3
21days	r-tail-base__Average_Height	-0.4600231	ID_4
21days	r-tail-base__Average_Height	-0.0594046	ID_5
21days	r-tail-base__Average_Height	-0.6471636	ID_6
21days	r-tail-base__Average_Height	-0.1401804	ID_7
21days	r-tail-base__Average_Height	-0.074647	ID_8
21days	r-tail-base__Average_Height	0.25237762	ID_9
0days	r-tail-base__Movement	-1.4884328	ID_10
0days	r-tail-base__Movement	0.6628308	ID_1
0days	r-tail-base__Movement	-0.0280488	ID_2
0days	r-tail-base__Movement	-0.7519261	ID_3
0days	r-tail-base__Movement	0.6841327	ID_4
0days	r-tail-base__Movement	1.21929734	ID_5
0days	r-tail-base__Movement	-0.1554949	ID_6
0days	r-tail-base__Movement	0.02804877	ID_7
0days	r-tail-base__Movement	-1.3594903	ID_8
0days	r-tail-base__Movement	-0.4168364	ID_9
0days	r-tail-base__Movement	0.3103583	ID_10
0days	r-tail-base__Movement	0.44898195	ID_11
3days	r-tail-base__Movement	-0.5096249	ID_12
3days	r-tail-base__Movement	-0.2337252	ID_13
3days	r-tail-base__Movement	0.24137003	ID_1
3days	r-tail-base__Movement	-0.2597581	ID_2
3days	r-tail-base__Movement	-1.2060889	ID_3
3days	r-tail-base__Movement	-0.4682711	ID_4
3days	r-tail-base__Movement	0.66638495	ID_5
3days	r-tail-base__Movement	0.66566255	ID_6
7days	r-tail-base__Movement	0.39704532	ID_7
7days	r-tail-base__Movement	-1.146498	ID_8
7days	r-tail-base__Movement	-1.4238269	ID_1
7days	r-tail-base__Movement	-0.3049662	ID_2
7days	r-tail-base__Movement	-0.6296294	ID_3
7days	r-tail-base__Movement	-0.976006	ID_4
7days	r-tail-base__Movement	-0.3438036	ID_5
7days	r-tail-base__Movement	-1.4581196	ID_6
7days	r-tail-base__Movement	-0.133536	ID_7
14days	r-tail-base__Movement	2.08222264	ID_8
14days	r-tail-base__Movement	-0.4948627	ID_9
14days	r-tail-base__Movement	-0.2319818	ID_1
14days	r-tail-base__Movement	-1.3286843	ID_2
14days	r-tail-base__Movement	-0.8492673	ID_3
14days	r-tail-base__Movement	0.62519524	ID_4
14days	r-tail-base__Movement	-1.6729081	ID_5
14days	r-tail-base__Movement	-1.5562913	ID_6
14days	r-tail-base__Movement	0.54171191	ID_7
14days	r-tail-base__Movement	0.09801549	ID_8
14days	r-tail-base__Movement	-0.3923154	ID_9
21days	r-tail-base__Movement	-0.5128268	ID_10
21days	r-tail-base__Movement	-1.0002567	ID_11
21days	r-tail-base__Movement	-0.7168314	ID_1
21days	r-tail-base__Movement	-1.4626938	ID_2
21days	r-tail-base__Movement	-1.5081911	ID_3
21days	r-tail-base__Movement	-0.3173735	ID_4
21days	r-tail-base__Movement	-0.6899591	ID_5
21days	r-tail-base__Movement	-1.1208162	ID_6

21days	r-tail-base__Movement	-1.1293678	ID_7
21days	r-tail-base__Movement	-0.0105076	ID_8
0days	r-wrist__Average_Height	0.26295344	ID_1
0days	r-wrist__Average_Height	0.51838424	ID_2
0days	r-wrist__Average_Height	0.03283572	ID_3
0days	r-wrist__Average_Height	0	ID_4
0days	r-wrist__Average_Height	-0.3560535	ID_5
0days	r-wrist__Average_Height	-0.1617122	ID_6
0days	r-wrist__Average_Height	-0.0015835	ID_7
0days	r-wrist__Average_Height	0.38365351	ID_8
0days	r-wrist__Average_Height	-0.2755916	ID_9
0days	r-wrist__Average_Height	-0.216229	ID_10
0days	r-wrist__Average_Height	-0.5535379	ID_11
0days	r-wrist__Average_Height	0.06851116	ID_12
0days	r-wrist__Average_Height	0.23325182	ID_13
3days	r-wrist__Average_Height	-0.87	ID_1
3days	r-wrist__Average_Height	-0.6058212	ID_2
3days	r-wrist__Average_Height	-0.4749152	ID_3
3days	r-wrist__Average_Height	-0.7838648	ID_4
3days	r-wrist__Average_Height	-0.4865431	ID_5
3days	r-wrist__Average_Height	-0.2179645	ID_6
3days	r-wrist__Average_Height	-0.5395759	ID_7
3days	r-wrist__Average_Height	-0.3081241	ID_8
7days	r-wrist__Average_Height	-0.2724075	ID_1
7days	r-wrist__Average_Height	0.52607768	ID_2
7days	r-wrist__Average_Height	-0.3460832	ID_3
7days	r-wrist__Average_Height	0.28358721	ID_4
7days	r-wrist__Average_Height	-0.228636	ID_5
7days	r-wrist__Average_Height	0.71822456	ID_6
7days	r-wrist__Average_Height	-0.7131964	ID_7
7days	r-wrist__Average_Height	-0.6053384	ID_8
7days	r-wrist__Average_Height	-0.6369777	ID_9
14days	r-wrist__Average_Height	-0.428482	ID_1
14days	r-wrist__Average_Height	-0.2108008	ID_2
14days	r-wrist__Average_Height	0.49135368	ID_3
14days	r-wrist__Average_Height	0.30058789	ID_4
14days	r-wrist__Average_Height	0.38267076	ID_5
14days	r-wrist__Average_Height	-0.9187281	ID_6
14days	r-wrist__Average_Height	0.2700295	ID_7
14days	r-wrist__Average_Height	0.45310704	ID_8
14days	r-wrist__Average_Height	-0.5165896	ID_9
14days	r-wrist__Average_Height	0.139375	ID_10
14days	r-wrist__Average_Height	-0.6044063	ID_11
21days	r-wrist__Average_Height	-0.0892213	ID_1
21days	r-wrist__Average_Height	0.21994043	ID_2
21days	r-wrist__Average_Height	-0.2067468	ID_3
21days	r-wrist__Average_Height	0.54698761	ID_4
21days	r-wrist__Average_Height	-0.5284613	ID_5
21days	r-wrist__Average_Height	0.43272774	ID_6
21days	r-wrist__Average_Height	-0.5033973	ID_7
21days	r-wrist__Average_Height	0.10798609	ID_8
21days	r-wrist__Average_Height	0.56595993	ID_9
21days	r-wrist__Average_Height	0.32389897	ID_10
0days	r-wrist__Movement	0.1908363	ID_1
0days	r-wrist__Movement	0.81883435	ID_2
0days	r-wrist__Movement	-0.1474982	ID_3

0days	r-wrist__Movement	0.84177678	ID_4
0days	r-wrist__Movement	0.18972796	ID_5
0days	r-wrist__Movement	-0.2184829	ID_6
0days	r-wrist__Movement	-0.058932	ID_7
0days	r-wrist__Movement	1.2874895	ID_8
0days	r-wrist__Movement	-0.0486132	ID_9
0days	r-wrist__Movement	-0.2187028	ID_10
0days	r-wrist__Movement	-0.47457	ID_11
0days	r-wrist__Movement	0	ID_12
0days	r-wrist__Movement	0.38107663	ID_13
3days	r-wrist__Movement	-1.1042429	ID_1
3days	r-wrist__Movement	-1.0237118	ID_2
3days	r-wrist__Movement	-0.9050514	ID_3
3days	r-wrist__Movement	-1.1928219	ID_4
3days	r-wrist__Movement	0.2480288	ID_5
3days	r-wrist__Movement	-0.5360732	ID_6
3days	r-wrist__Movement	-0.2693848	ID_7
3days	r-wrist__Movement	-0.4260094	ID_8
7days	r-wrist__Movement	-0.0994617	ID_1
7days	r-wrist__Movement	0.65778584	ID_2
7days	r-wrist__Movement	-0.5010031	ID_3
7days	r-wrist__Movement	0.5203005	ID_4
7days	r-wrist__Movement	-0.3331101	ID_5
7days	r-wrist__Movement	1.50076289	ID_6
7days	r-wrist__Movement	-0.859766	ID_7
7days	r-wrist__Movement	-0.7879348	ID_8
7days	r-wrist__Movement	-0.7187565	ID_9
14days	r-wrist__Movement	-0.8010218	ID_1
14days	r-wrist__Movement	0.11010138	ID_2
14days	r-wrist__Movement	0.50357344	ID_3
14days	r-wrist__Movement	0.2559005	ID_4
14days	r-wrist__Movement	0.81963793	ID_5
14days	r-wrist__Movement	-1.1598687	ID_6
14days	r-wrist__Movement	0.64383236	ID_7
14days	r-wrist__Movement	0.53176811	ID_8
14days	r-wrist__Movement	-0.2621248	ID_9
14days	r-wrist__Movement	0.34417921	ID_10
14days	r-wrist__Movement	-0.5241281	ID_11
21days	r-wrist__Movement	-0.1508607	ID_1
21days	r-wrist__Movement	0.52496405	ID_2
21days	r-wrist__Movement	-0.332752	ID_3
21days	r-wrist__Movement	0.88027179	ID_4
21days	r-wrist__Movement	-0.5483315	ID_5
21days	r-wrist__Movement	0.62183994	ID_6
21days	r-wrist__Movement	-0.5415581	ID_7
21days	r-wrist__Movement	0.04978707	ID_8
21days	r-wrist__Movement	1.03831121	ID_9
21days	r-wrist__Movement	1.07516934	ID_10

Suppl. Table 20: Summary of side perspective height analysis

day	Measure	Value_Mean	Value_SD
0days	l-back-ankle__Average_Heig	0	0.67205514
0days	l-back-ankle__Movement	0	1.39839106
0days	l-back-toe__Average_Height	0	0.30972082
0days	l-back-toe__Movement	0	1.13500976
0days	l-elbow__Average_Height	0	0.2953487
0days	l-elbow__Movement	0	0.56956062
0days	l-front-toe-tip__Average_Hei	0	0.21328618
0days	l-front-toe-tip__Movement	0	0.68814422
0days	l-head__Average_Height	0	0.69609999
0days	l-head__Movement	0	1.78519764
0days	l-hip__Average_Height	0	0.27585357
0days	l-hip__Movement	0	0.58467604
0days	l-iliac-crest__Average_Height	0	0.29740806
0days	l-iliac-crest__Movement	0	0.77458719
0days	l-shoulder__Average_Height	0	0.34751774
0days	l-shoulder__Movement	0	0.78403194
0days	l-tail-base__Average_Height	0	0.36446301
0days	l-tail-base__Movement	0	1.02050403
0days	l-wrist__Average_Height	0	0.4812395
0days	l-wrist__Movement	0	0.56512497
0days	r-back-ankle__Average_Heig	0	0.42151241
0days	r-back-ankle__Movement	0	0.81877275
0days	r-back-toe__Average_Height	0	0.15521927
0days	r-back-toe__Movement	0	0.61251167
0days	r-elbow__Average_Height	0	0.27137972
0days	r-elbow__Movement	0	0.58583293
0days	r-front-toe-tip__Average_Hei	0	0.21423905
0days	r-front-toe-tip__Movement	0	0.50975445
0days	r-head__Average_Height	0	0.29488311
0days	r-head__Movement	0	0.89707013
0days	r-hip__Average_Height	0	0.27823373
0days	r-hip__Movement	0	0.73561941
0days	r-iliac-crest__Average_Heigh	0	0.24053368
0days	r-iliac-crest__Movement	0	0.77555514
0days	r-shoulder__Average_Height	0	0.30191406
0days	r-shoulder__Movement	0	0.62044276
0days	r-tail-base__Average_Height	0	0.57825888
0days	r-tail-base__Movement	0	0.82356179
0days	r-wrist__Average_Height	0	0.30645097
0days	r-wrist__Movement	0	0.50869838
3days	l-back-ankle__Average_Heig	0.02570948	0.58695474
3days	l-back-ankle__Movement	0.01975225	1.19471529
3days	l-back-toe__Average_Height	-0.2515432	0.09761713
3days	l-back-toe__Movement	-1.1891297	0.30131302
3days	l-elbow__Average_Height	-0.1817734	0.11139445
3days	l-elbow__Movement	-0.6664027	0.23348492
3days	l-front-toe-tip__Average_Hei	-0.4894445	0.15474541
3days	l-front-toe-tip__Movement	-0.7720142	0.32233553
3days	l-head__Average_Height	-0.6188926	0.2477646
3days	l-head__Movement	-2.4197983	0.85032677
3days	l-hip__Average_Height	-0.3319208	0.26338908
3days	l-hip__Movement	-0.4688948	0.70446915
3days	l-iliac-crest__Average_Height	-0.1741278	0.2886531
3days	l-iliac-crest__Movement	-0.6873641	0.75218416

3days	l-shoulder__Average_Height	-0.2549553	0.1938969
3days	l-shoulder__Movement	-0.7711989	0.23496572
3days	l-tail-base__Average_Height	-0.1843617	0.50454738
3days	l-tail-base__Movement	-0.8913054	0.90303613
3days	l-wrist__Average_Height	-0.6604331	0.33195927
3days	l-wrist__Movement	-0.8620946	0.59232428
3days	r-back-ankle__Average_Heig	0.29535306	0.54584935
3days	r-back-ankle__Movement	-0.2481358	1.08787015
3days	r-back-toe__Average_Height	-0.1167776	0.14477382
3days	r-back-toe__Movement	-0.2128761	0.65892732
3days	r-elbow__Average_Height	-0.4631945	0.25532092
3days	r-elbow__Movement	-0.9819639	0.52538211
3days	r-front-toe-tip__Average_Hei	-0.2258158	0.16613749
3days	r-front-toe-tip__Movement	-0.1309558	0.22474753
3days	r-head__Average_Height	-0.5023801	0.6731405
3days	r-head__Movement	-1.3000743	1.40052281
3days	r-hip__Average_Height	-0.3189425	0.27016954
3days	r-hip__Movement	-0.5717939	0.7964572
3days	r-iliac-crest__Average_Heigh	-0.0896488	0.19038016
3days	r-iliac-crest__Movement	-0.3541893	0.5509961
3days	r-shoulder__Average_Height	-0.039671	0.32845559
3days	r-shoulder__Movement	-0.1246607	0.55625091
3days	r-tail-base__Average_Height	-0.058509	0.38805286
3days	r-tail-base__Movement	-0.2467417	0.63794498
3days	r-wrist__Average_Height	-0.5130595	0.21945775
3days	r-wrist__Movement	-0.7205623	0.49571998
7days	l-back-ankle__Average_Heig	-0.351378	0.37982559
7days	l-back-ankle__Movement	-0.6315821	0.54145176
7days	l-back-toe__Average_Height	-0.18197	0.10763682
7days	l-back-toe__Movement	-1.1917231	0.4414276
7days	l-elbow__Average_Height	-0.1196973	0.19817834
7days	l-elbow__Movement	-0.2017046	0.64264963
7days	l-front-toe-tip__Average_Hei	-0.3830309	0.06593579
7days	l-front-toe-tip__Movement	-0.7220486	0.16284231
7days	l-head__Average_Height	-0.7840083	0.58142185
7days	l-head__Movement	-2.0484046	1.41819698
7days	l-hip__Average_Height	-0.2643041	0.28978815
7days	l-hip__Movement	-0.3605221	0.49323238
7days	l-iliac-crest__Average_Heigh	-0.2711173	0.29193128
7days	l-iliac-crest__Movement	-0.5854092	0.65743408
7days	l-shoulder__Average_Height	-0.2049188	0.15827927
7days	l-shoulder__Movement	-0.7485486	0.19805054
7days	l-tail-base__Average_Height	-0.19543	0.30844884
7days	l-tail-base__Movement	-0.5314102	0.79321631
7days	l-wrist__Average_Height	-0.7732756	0.24734098
7days	l-wrist__Movement	-1.0666631	0.46789948
7days	r-back-ankle__Average_Heig	0.36533421	0.38194934
7days	r-back-ankle__Movement	0.05341691	0.98345023
7days	r-back-toe__Average_Height	-0.0422763	0.32740692
7days	r-back-toe__Movement	0.57444742	0.54023539
7days	r-elbow__Average_Height	-0.269897	0.17864471
7days	r-elbow__Movement	-0.9367159	0.215844
7days	r-front-toe-tip__Average_Hei	-0.1284495	0.28579316
7days	r-front-toe-tip__Movement	-0.1118996	0.55254655
7days	r-head__Average_Height	-0.2459201	0.57873624
7days	r-head__Movement	-1.0167984	1.4092157

7days	r-hip__Average_Height	-0.0476098	0.19670667
7days	r-hip__Movement	-0.076238	0.42739124
7days	r-iliac-crest__Average_Heigh	0.03775729	0.34700319
7days	r-iliac-crest__Movement	-0.1402113	0.77575065
7days	r-shoulder__Average_Height	-0.0876859	0.11653689
7days	r-shoulder__Movement	-0.3843292	0.19162907
7days	r-tail-base__Average_Height	-0.2709525	0.39692806
7days	r-tail-base__Movement	-0.6296294	0.63025073
7days	r-wrist__Average_Height	-0.2724075	0.52672929
7days	r-wrist__Movement	-0.3331101	0.80322153
14days	l-back-ankle__Average_Heig	0.0320646	0.6093304
14days	l-back-ankle__Movement	0.27062048	0.84411346
14days	l-back-toe__Average_Height	-0.1521897	0.16525996
14days	l-back-toe__Movement	-0.8431799	0.82663937
14days	l-elbow__Average_Height	0.17608252	0.3304861
14days	l-elbow__Movement	0.70404172	0.74357192
14days	l-front-toe-tip__Average_Hei	-0.2986062	0.26680589
14days	l-front-toe-tip__Movement	-0.419892	0.58181483
14days	l-head__Average_Height	-0.1329957	0.62624392
14days	l-head__Movement	-0.9574556	1.68229377
14days	l-hip__Average_Height	-0.4010188	0.35154962
14days	l-hip__Movement	-0.0778198	0.70516224
14days	l-iliac-crest__Average_Height	-0.4039169	0.28894924
14days	l-iliac-crest__Movement	-0.6846737	0.79258113
14days	l-shoulder__Average_Height	0.02657494	0.18503801
14days	l-shoulder__Movement	-0.1772168	0.41194747
14days	l-tail-base__Average_Height	-0.3294469	0.65252075
14days	l-tail-base__Movement	-0.9008344	1.13319296
14days	l-wrist__Average_Height	-0.7906645	0.4105328
14days	l-wrist__Movement	-0.7988941	0.54820836
14days	r-back-ankle__Average_Heig	0.70032853	0.4892584
14days	r-back-ankle__Movement	0.57465742	1.0550547
14days	r-back-toe__Average_Height	0.14321988	0.31110989
14days	r-back-toe__Movement	0.51693385	0.87934271
14days	r-elbow__Average_Height	-0.2750497	0.20660772
14days	r-elbow__Movement	-0.6878739	0.47170411
14days	r-front-toe-tip__Average_Hei	0.03896691	0.2579201
14days	r-front-toe-tip__Movement	0.19486483	0.59646224
14days	r-head__Average_Height	0.00655582	0.68196822
14days	r-head__Movement	-0.4262264	1.52949285
14days	r-hip__Average_Height	-0.0463459	0.27163519
14days	r-hip__Movement	0.08961341	0.5628902
14days	r-iliac-crest__Average_Heigh	0.13260314	0.33662786
14days	r-iliac-crest__Movement	0.39869722	0.65654526
14days	r-shoulder__Average_Height	-0.016972	0.17646677
14days	r-shoulder__Movement	-0.1576145	0.39330664
14days	r-tail-base__Average_Height	-0.1276667	0.43576028
14days	r-tail-base__Movement	-0.3923154	1.10773143
14days	r-wrist__Average_Height	0.139375	0.49423445
14days	r-wrist__Movement	0.2559005	0.64262869
21days	l-back-ankle__Average_Heig	-0.2302505	0.8024245
21days	l-back-ankle__Movement	-0.7434575	1.19399032
21days	l-back-toe__Average_Height	-0.0553157	0.16828268
21days	l-back-toe__Movement	-0.5940266	0.6753578
21days	l-elbow__Average_Height	0.18487568	0.41207686
21days	l-elbow__Movement	0.26455313	0.96353144

21days	l-front-toe-tip__Average_Hei	-0.0893582	0.26423018
21days	l-front-toe-tip__Movement	-0.1606363	0.57479182
21days	l-head__Average_Height	-0.3836985	0.66208614
21days	l-head__Movement	-1.8546309	1.604357
21days	l-hip__Average_Height	-0.1809973	0.26610034
21days	l-hip__Movement	-0.1865542	0.59149771
21days	l-iliac-crest__Average_Heigh	-0.0731095	0.33413492
21days	l-iliac-crest__Movement	-0.3395875	0.55901705
21days	l-shoulder__Average_Height	-0.0736804	0.18763304
21days	l-shoulder__Movement	-0.4397853	0.25248021
21days	l-tail-base__Average_Height	-0.2371947	0.25386117
21days	l-tail-base__Movement	-0.9806249	0.53855857
21days	l-wrist__Average_Height	-0.3176237	0.38897663
21days	l-wrist__Movement	0.06593108	0.73611936
21days	r-back-ankle__Average_Heig	0.42509925	0.43154865
21days	r-back-ankle__Movement	0.73807505	0.99192076
21days	r-back-toe__Average_Height	0.14850844	0.36265549
21days	r-back-toe__Movement	0.49927316	0.99247557
21days	r-elbow__Average_Height	-0.0764663	0.35030255
21days	r-elbow__Movement	-0.4391015	0.8225804
21days	r-front-toe-tip__Average_Hei	-0.0184253	0.32348955
21days	r-front-toe-tip__Movement	0.00602683	0.55799092
21days	r-head__Average_Height	-0.1934474	0.36130061
21days	r-head__Movement	-0.6266862	0.79998934
21days	r-hip__Average_Height	-0.1058788	0.35112317
21days	r-hip__Movement	-0.1093872	0.70830282
21days	r-iliac-crest__Average_Heigh	0.01160277	0.38496129
21days	r-iliac-crest__Movement	-0.1016065	0.78793804
21days	r-shoulder__Average_Height	-0.2018968	0.22436402
21days	r-shoulder__Movement	-0.3803541	0.33118472
21days	r-tail-base__Average_Height	-0.1643088	0.25345077
21days	r-tail-base__Movement	-0.858544	0.48624961
21days	r-wrist__Average_Height	0.16396326	0.405443
21days	r-wrist__Movement	0.28737556	0.64259529

Suppl. Table 21: Statistical test of bside perspective height analysis

Measure	group1	group2	p.adj	sig	Test
l-wrist__Movement	0days	7days	0.00033674	***	Repeat ANOVA + PH test
l-wrist__Movement	0days	3days	0.00034229	***	Repeat ANOVA + PH test
l-back-toe__Movement	0days	7days	0.00086311	***	Repeat ANOVA + PH test
l-back-toe__Movement	0days	3days	0.00086311	***	Repeat ANOVA + PH test
l-front-toe-tip__Movement	0days	7days	0.00281907	**	Repeat ANOVA + PH test
l-front-toe-tip__Movement	0days	3days	0.00662191	**	Repeat ANOVA + PH test
r-head__Movement	0days	3days	0.00870331	**	Repeat ANOVA + PH test
r-back-ankle__Movement	0days	14days	0.00887464	**	Repeat ANOVA + PH test
l-wrist__Movement	0days	14days	0.0103341	*	Repeat ANOVA + PH test
r-wrist__Movement	0days	3days	0.01154712	*	Repeat ANOVA + PH test
l-back-ankle__Movement	0days	21days	0.01242591	*	Repeat ANOVA + PH test
r-elbow__Movement	0days	7days	0.01630839	*	Repeat ANOVA + PH test
r-tail-base__Movement	0days	21days	0.01656914	*	Repeat ANOVA + PH test
l-shoulder__Movement	0days	7days	0.01905166	*	Repeat ANOVA + PH test
l-back-toe__Movement	0days	14days	0.02112799	*	Repeat ANOVA + PH test
l-back-toe__Movement	0days	21days	0.02431522	*	Repeat ANOVA + PH test
r-back-toe__Movement	0days	21days	0.03658615	*	Repeat ANOVA + PH test
l-wrist__Average_Height	0days	14days	0.03960501	*	Repeat ANOVA + PH test
l-wrist__Average_Height	0days	3days	0.04154142	*	Repeat ANOVA + PH test
l-shoulder__Movement	0days	3days	0.04981773	*	Repeat ANOVA + PH test
l-wrist__Average_Height	0days	7days	0.05797498	*	Repeat ANOVA + PH test
r-elbow__Movement	0days	3days	0.05872624	*	Repeat ANOVA + PH test
l-back-ankle__Movement	0days	3days	0.06160991	*	Repeat ANOVA + PH test
l-back-ankle__Movement	0days	7days	0.06160991	*	Repeat ANOVA + PH test
l-elbow__Movement	0days	3days	0.0680736	*	Repeat ANOVA + PH test
r-head__Movement	0days	7days	0.07106673	*	Repeat ANOVA + PH test
l-iliac-crest__Movement	0days	14days	0.07607329	*	Repeat ANOVA + PH test
r-head__Movement	0days	21days	0.08018946	*	Repeat ANOVA + PH test
r-tail-base__Movement	0days	7days	0.09549484	*	Repeat ANOVA + PH test
l-shoulder__Movement	0days	21days	0.09593472	*	Repeat ANOVA + PH test
r-elbow__Movement	0days	14days	0.10261614	ns	Repeat ANOVA + PH test
r-back-ankle__Movement	0days	21days	0.1363321	ns	Repeat ANOVA + PH test
l-front-toe-tip__Movement	0days	14days	0.14456596	ns	Repeat ANOVA + PH test
l-hip__Movement	0days	7days	0.14519584	ns	Repeat ANOVA + PH test
l-hip__Movement	0days	3days	0.14519584	ns	Repeat ANOVA + PH test
l-hip__Movement	0days	14days	0.14519584	ns	Repeat ANOVA + PH test
l-hip__Movement	0days	21days	0.14519584	ns	Repeat ANOVA + PH test
l-front-toe-tip__Movement	0days	21days	0.16410928	ns	Repeat ANOVA + PH test
r-shoulder__Movement	0days	7days	0.16842294	ns	Repeat ANOVA + PH test
l-elbow__Movement	0days	21days	0.18648537	ns	Repeat ANOVA + PH test
r-shoulder__Movement	0days	21days	0.18735186	ns	Repeat ANOVA + PH test
r-wrist__Average_Height	0days	3days	0.24632881	ns	Repeat ANOVA + PH test
l-shoulder__Movement	0days	14days	0.26841028	ns	Repeat ANOVA + PH test
l-tail-base__Movement	0days	21days	0.28857549	ns	Repeat ANOVA + PH test
r-head__Movement	0days	14days	0.29530385	ns	Repeat ANOVA + PH test
l-iliac-crest__Movement	0days	7days	0.3239988	ns	Repeat ANOVA + PH test
l-iliac-crest__Movement	0days	3days	0.3239988	ns	Repeat ANOVA + PH test
l-iliac-crest__Movement	0days	21days	0.3239988	ns	Repeat ANOVA + PH test
r-shoulder__Movement	0days	14days	0.37581512	ns	Repeat ANOVA + PH test
r-shoulder__Movement	0days	3days	0.37581512	ns	Repeat ANOVA + PH test
l-elbow__Movement	0days	14days	0.38853879	ns	Repeat ANOVA + PH test
r-back-toe__Movement	0days	14days	0.40695018	ns	Repeat ANOVA + PH test
r-back-toe__Movement	0days	7days	0.40778698	ns	Repeat ANOVA + PH test
l-elbow__Movement	0days	7days	0.4104934	ns	Repeat ANOVA + PH test

l-wrist__Average_Height	0days	21days	0.4227289	ns	Repeat ANOVA + PH test
l-back-ankle__Movement	0days	14days	0.51051272	ns	Repeat ANOVA + PH test
r-iliac-crest__Movement	0days	3days	0.51755507	ns	Repeat ANOVA + PH test
r-hip__Movement	0days	3days	0.51909376	ns	Repeat ANOVA + PH test
r-elbow__Movement	0days	21days	0.54997212	ns	Repeat ANOVA + PH test
l-front-toe-tip__Average_He	0days	3days	0.62671622	ns	Repeat ANOVA + PH test
r-tail-base__Average_Height	0days	21days	0.70378738	ns	Repeat ANOVA + PH test
r-tail-base__Average_Height	0days	7days	0.70378738	ns	Repeat ANOVA + PH test
r-tail-base__Average_Height	0days	14days	0.71642155	ns	Repeat ANOVA + PH test
r-tail-base__Average_Height	0days	3days	0.71642155	ns	Repeat ANOVA + PH test
l-wrist__Movement	0days	21days	0.72937234	ns	Repeat ANOVA + PH test
l-front-toe-tip__Average_He	0days	7days	0.74647315	ns	Repeat ANOVA + PH test
l-front-toe-tip__Average_He	0days	14days	0.74647315	ns	Repeat ANOVA + PH test
l-front-toe-tip__Average_He	0days	21days	0.74647315	ns	Repeat ANOVA + PH test
r-back-ankle__Average_Hei	0days	14days	0.80579192	ns	Repeat ANOVA + PH test
r-tail-base__Movement	0days	14days	0.81479353	ns	Repeat ANOVA + PH test
r-tail-base__Movement	0days	3days	0.8150049	ns	Repeat ANOVA + PH test
r-back-toe__Movement	0days	3days	0.89626413	ns	Repeat ANOVA + PH test
l-iliac-crest__Average_Heigl	0days	14days	0.9075294	ns	Repeat ANOVA + PH test
l-tail-base__Movement	0days	14days	0.92793154	ns	Repeat ANOVA + PH test
l-back-ankle__Average_Hei	0days	7days	1	ns	Repeat ANOVA + PH test
l-back-ankle__Average_Hei	0days	21days	1	ns	Repeat ANOVA + PH test
l-back-ankle__Average_Hei	0days	14days	1	ns	Repeat ANOVA + PH test
l-back-ankle__Average_Hei	0days	3days	1	ns	Repeat ANOVA + PH test
l-back-toe__Average_Heigh	0days	3days	1	ns	Repeat ANOVA + PH test
l-back-toe__Average_Heigh	0days	7days	1	ns	Repeat ANOVA + PH test
l-back-toe__Average_Heigh	0days	14days	1	ns	Repeat ANOVA + PH test
l-back-toe__Average_Heigh	0days	21days	1	ns	Repeat ANOVA + PH test
l-elbow__Average_Height	0days	21days	1	ns	Repeat ANOVA + PH test
l-elbow__Average_Height	0days	14days	1	ns	Repeat ANOVA + PH test
l-elbow__Average_Height	0days	3days	1	ns	Repeat ANOVA + PH test
l-elbow__Average_Height	0days	7days	1	ns	Repeat ANOVA + PH test
l-hip__Average_Height	0days	14days	1	ns	Repeat ANOVA + PH test
l-hip__Average_Height	0days	7days	1	ns	Repeat ANOVA + PH test
l-hip__Average_Height	0days	3days	1	ns	Repeat ANOVA + PH test
l-hip__Average_Height	0days	21days	1	ns	Repeat ANOVA + PH test
l-iliac-crest__Average_Heigl	0days	7days	1	ns	Repeat ANOVA + PH test
l-iliac-crest__Average_Heigl	0days	3days	1	ns	Repeat ANOVA + PH test
l-iliac-crest__Average_Heigl	0days	21days	1	ns	Repeat ANOVA + PH test
l-shoulder__Average_Heigh	0days	7days	1	ns	Repeat ANOVA + PH test
l-shoulder__Average_Heigh	0days	3days	1	ns	Repeat ANOVA + PH test
l-shoulder__Average_Heigh	0days	21days	1	ns	Repeat ANOVA + PH test
l-shoulder__Average_Heigh	0days	14days	1	ns	Repeat ANOVA + PH test
l-tail-base__Average_Height	0days	21days	1	ns	Repeat ANOVA + PH test
l-tail-base__Average_Height	0days	7days	1	ns	Repeat ANOVA + PH test
l-tail-base__Average_Height	0days	3days	1	ns	Repeat ANOVA + PH test
l-tail-base__Average_Height	0days	14days	1	ns	Repeat ANOVA + PH test
l-tail-base__Movement	0days	7days	1	ns	Repeat ANOVA + PH test
l-tail-base__Movement	0days	3days	1	ns	Repeat ANOVA + PH test
r-back-ankle__Average_Hei	0days	21days	1	ns	Repeat ANOVA + PH test
r-back-ankle__Average_Hei	0days	7days	1	ns	Repeat ANOVA + PH test
r-back-ankle__Average_Hei	0days	3days	1	ns	Repeat ANOVA + PH test
r-back-ankle__Movement	0days	7days	1	ns	Repeat ANOVA + PH test
r-back-ankle__Movement	0days	3days	1	ns	Repeat ANOVA + PH test
r-back-toe__Average_Heigh	0days	21days	1	ns	Repeat ANOVA + PH test
r-back-toe__Average_Heigh	0days	14days	1	ns	Repeat ANOVA + PH test

r-back-toe__Average_Heigh	0days	7days	1	ns	Repeat ANOVA + PH test
r-back-toe__Average_Heigh	0days	3days	1	ns	Repeat ANOVA + PH test
r-elbow__Average_Height	0days	3days	1	ns	Repeat ANOVA + PH test
r-elbow__Average_Height	0days	14days	1	ns	Repeat ANOVA + PH test
r-elbow__Average_Height	0days	7days	1	ns	Repeat ANOVA + PH test
r-elbow__Average_Height	0days	21days	1	ns	Repeat ANOVA + PH test
r-front-toe-tip__Average_He	0days	3days	1	ns	Repeat ANOVA + PH test
r-front-toe-tip__Average_He	0days	7days	1	ns	Repeat ANOVA + PH test
r-front-toe-tip__Average_He	0days	14days	1	ns	Repeat ANOVA + PH test
r-front-toe-tip__Average_He	0days	21days	1	ns	Repeat ANOVA + PH test
r-front-toe-tip__Movement	0days	3days	1	ns	Repeat ANOVA + PH test
r-front-toe-tip__Movement	0days	21days	1	ns	Repeat ANOVA + PH test
r-front-toe-tip__Movement	0days	14days	1	ns	Repeat ANOVA + PH test
r-front-toe-tip__Movement	0days	7days	1	ns	Repeat ANOVA + PH test
r-head__Average_Height	0days	3days	1	ns	Repeat ANOVA + PH test
r-head__Average_Height	0days	7days	1	ns	Repeat ANOVA + PH test
r-head__Average_Height	0days	14days	1	ns	Repeat ANOVA + PH test
r-head__Average_Height	0days	21days	1	ns	Repeat ANOVA + PH test
r-hip__Average_Height	0days	3days	1	ns	Repeat ANOVA + PH test
r-hip__Average_Height	0days	7days	1	ns	Repeat ANOVA + PH test
r-hip__Average_Height	0days	14days	1	ns	Repeat ANOVA + PH test
r-hip__Average_Height	0days	21days	1	ns	Repeat ANOVA + PH test
r-hip__Movement	0days	14days	1	ns	Repeat ANOVA + PH test
r-hip__Movement	0days	7days	1	ns	Repeat ANOVA + PH test
r-hip__Movement	0days	21days	1	ns	Repeat ANOVA + PH test
r-iliac-crest__Average_Heigl	0days	3days	1	ns	Repeat ANOVA + PH test
r-iliac-crest__Average_Heigl	0days	14days	1	ns	Repeat ANOVA + PH test
r-iliac-crest__Average_Heigl	0days	7days	1	ns	Repeat ANOVA + PH test
r-iliac-crest__Average_Heigl	0days	21days	1	ns	Repeat ANOVA + PH test
r-iliac-crest__Movement	0days	14days	1	ns	Repeat ANOVA + PH test
r-iliac-crest__Movement	0days	21days	1	ns	Repeat ANOVA + PH test
r-iliac-crest__Movement	0days	7days	1	ns	Repeat ANOVA + PH test
r-shoulder__Average_Heigh	0days	21days	1	ns	Repeat ANOVA + PH test
r-shoulder__Average_Heigh	0days	7days	1	ns	Repeat ANOVA + PH test
r-shoulder__Average_Heigh	0days	14days	1	ns	Repeat ANOVA + PH test
r-shoulder__Average_Heigh	0days	3days	1	ns	Repeat ANOVA + PH test
r-wrist__Average_Height	0days	7days	1	ns	Repeat ANOVA + PH test
r-wrist__Average_Height	0days	21days	1	ns	Repeat ANOVA + PH test
r-wrist__Average_Height	0days	14days	1	ns	Repeat ANOVA + PH test
r-wrist__Movement	0days	7days	1	ns	Repeat ANOVA + PH test
r-wrist__Movement	0days	14days	1	ns	Repeat ANOVA + PH test
r-wrist__Movement	0days	21days	1	ns	Repeat ANOVA + PH test

Suppl. Table 22: Raw data side perspective horizontal analysis

day	ID	Measure	Length, mm
0days	ID_1	left__back__average	-0.665732881
0days	ID_2	left__back__average	-2.43937031
0days	ID_3	left__back__average	-3.165219533
0days	ID_4	left__back__average	2.780764825
0days	ID_5	left__back__average	-1.016136842
0days	ID_6	left__back__average	-0.11607343
0days	ID_7	left__back__average	0.331419497
0days	ID_8	left__back__average	0.998726988
0days	ID_9	left__back__average	1.029317169
0days	ID_10	left__back__average	1.439472752
0days	ID_11	left__back__average	-6.288500711
0days	ID_12	left__back__average	0.790370464
0days	ID_13	left__back__average	0
3days	ID_1	left__back__average	-3.252123236
3days	ID_2	left__back__average	-0.693573233
3days	ID_3	left__back__average	-1.321642182
3days	ID_4	left__back__average	-4.446968622
3days	ID_5	left__back__average	-5.077335534
3days	ID_6	left__back__average	-2.322387965
3days	ID_7	left__back__average	0.183361776
3days	ID_8	left__back__average	-0.502326708
7days	ID_1	left__back__average	-2.16032667
7days	ID_2	left__back__average	-2.015743425
7days	ID_3	left__back__average	-2.928351493
7days	ID_4	left__back__average	-2.23863132
7days	ID_5	left__back__average	-2.862697583
7days	ID_6	left__back__average	-2.712748448
7days	ID_7	left__back__average	-3.986746149
7days	ID_8	left__back__average	-1.923224411
7days	ID_9	left__back__average	-2.317728529
14days	ID_1	left__back__average	0.323925364
14days	ID_2	left__back__average	-3.950857748
14days	ID_3	left__back__average	-2.796805228
14days	ID_4	left__back__average	-0.687508374
14days	ID_5	left__back__average	-0.947685938
14days	ID_6	left__back__average	-7.996255861
14days	ID_7	left__back__average	-1.453731628
14days	ID_8	left__back__average	-1.463413908
14days	ID_9	left__back__average	-0.913276722
14days	ID_10	left__back__average	-4.226148943
14days	ID_11	left__back__average	1.900302369
21days	ID_1	left__back__average	-0.672312122
21days	ID_2	left__back__average	-0.248371865
21days	ID_3	left__back__average	-3.100045223
21days	ID_4	left__back__average	-0.890662024
21days	ID_5	left__back__average	1.142253382

21days	ID_6	left__back__average	0.988501652
21days	ID_7	left__back__average	0.482267541
21days	ID_8	left__back__average	1.844449648
21days	ID_9	left__back__average	1.094912192
21days	ID_10	left__back__average	-0.679299503
0days	ID_1	left__back__median	-1.674089125
0days	ID_2	left__back__median	-4.439980208
0days	ID_3	left__back__median	-5.078559266
0days	ID_4	left__back__median	2.833925522
0days	ID_5	left__back__median	-2.598521964
0days	ID_6	left__back__median	-0.140126659
0days	ID_7	left__back__median	0
0days	ID_8	left__back__median	0.999616945
0days	ID_9	left__back__median	1.562406868
0days	ID_10	left__back__median	2.202879524
0days	ID_11	left__back__median	-8.200006562
0days	ID_12	left__back__median	0.828872722
0days	ID_13	left__back__median	0.040724184
3days	ID_1	left__back__median	-6.2651764
3days	ID_2	left__back__median	-2.219129741
3days	ID_3	left__back__median	-3.693438635
3days	ID_4	left__back__median	-7.473039746
3days	ID_5	left__back__median	-6.403805071
3days	ID_6	left__back__median	-4.467193939
3days	ID_7	left__back__median	1.544503241
3days	ID_8	left__back__median	-0.534412942
7days	ID_1	left__back__median	-3.80758827
7days	ID_2	left__back__median	-3.348074382
7days	ID_3	left__back__median	-3.855174598
7days	ID_4	left__back__median	-1.834017477
7days	ID_5	left__back__median	-3.759343996
7days	ID_6	left__back__median	-3.443448227
7days	ID_7	left__back__median	-4.111624234
7days	ID_8	left__back__median	-1.317366042
7days	ID_9	left__back__median	-1.844135333
14days	ID_1	left__back__median	-0.695586301
14days	ID_2	left__back__median	-6.229323465
14days	ID_3	left__back__median	-4.933527567
14days	ID_4	left__back__median	-0.795866341
14days	ID_5	left__back__median	-1.176232972
14days	ID_6	left__back__median	-12.74506704
14days	ID_7	left__back__median	-1.659057825
14days	ID_8	left__back__median	-1.623890045
14days	ID_9	left__back__median	-3.03993928
14days	ID_10	left__back__median	-4.904743153
14days	ID_11	left__back__median	2.441005261
21days	ID_1	left__back__median	-2.050191019
21days	ID_2	left__back__median	0.448892446

21days	ID_3	left__back__median	-4.139947609
21days	ID_4	left__back__median	-1.527488306
21days	ID_5	left__back__median	1.802578924
21days	ID_6	left__back__median	0.815885025
21days	ID_7	left__back__median	1.306126126
21days	ID_8	left__back__median	1.568666375
21days	ID_9	left__back__median	0.326482619
21days	ID_10	left__back__median	-1.795462783
0days	ID_1	left__back__protraction	-1.007182427
0days	ID_2	left__back__protraction	-3.653931951
0days	ID_3	left__back__protraction	-2.141409021
0days	ID_4	left__back__protraction	0.576821813
0days	ID_5	left__back__protraction	0.309006776
0days	ID_6	left__back__protraction	1.158577959
0days	ID_7	left__back__protraction	0.596753465
0days	ID_8	left__back__protraction	-1.254364972
0days	ID_9	left__back__protraction	0.226353945
0days	ID_10	left__back__protraction	0
0days	ID_11	left__back__protraction	-4.147008126
0days	ID_12	left__back__protraction	0.754228211
0days	ID_13	left__back__protraction	-0.60652709
3days	ID_1	left__back__protraction	-1.274293944
3days	ID_2	left__back__protraction	0.096048212
3days	ID_3	left__back__protraction	-1.865729097
3days	ID_4	left__back__protraction	-1.979965267
3days	ID_5	left__back__protraction	-5.552383843
3days	ID_6	left__back__protraction	-1.237684327
3days	ID_7	left__back__protraction	-1.105148234
3days	ID_8	left__back__protraction	-0.442272116
7days	ID_1	left__back__protraction	-2.661670395
7days	ID_2	left__back__protraction	-3.245417491
7days	ID_3	left__back__protraction	-1.545360848
7days	ID_4	left__back__protraction	-2.447104324
7days	ID_5	left__back__protraction	-2.507040057
7days	ID_6	left__back__protraction	-2.07934681
7days	ID_7	left__back__protraction	-0.816228153
7days	ID_8	left__back__protraction	-1.485682354
7days	ID_9	left__back__protraction	-1.402951927
14days	ID_1	left__back__protraction	-0.324161835
14days	ID_2	left__back__protraction	-1.57424086
14days	ID_3	left__back__protraction	-2.527734181
14days	ID_4	left__back__protraction	-1.709245047
14days	ID_5	left__back__protraction	-1.607006233
14days	ID_6	left__back__protraction	-3.121501111
14days	ID_7	left__back__protraction	-0.924197633
14days	ID_8	left__back__protraction	-1.643220046
14days	ID_9	left__back__protraction	-0.153902842
14days	ID_10	left__back__protraction	-3.418664461

14days	ID_11	left__back__protraction	2.046542141
21days	ID_1	left__back__protraction	-0.24156792
21days	ID_2	left__back__protraction	1.626731032
21days	ID_3	left__back__protraction	0.851509272
21days	ID_4	left__back__protraction	0.064025612
21days	ID_5	left__back__protraction	1.261124624
21days	ID_6	left__back__protraction	0.445258176
21days	ID_7	left__back__protraction	0.348099895
21days	ID_8	left__back__protraction	0.664082467
21days	ID_9	left__back__protraction	0.599460844
21days	ID_10	left__back__protraction	0.506442497
0days	ID_8	left__back__retraction	1.210140798
0days	ID_9	left__back__retraction	0.02219099
0days	ID_10	left__back__retraction	-1.162018988
0days	ID_8	left__back__retraction	3.017222757
0days	ID_9	left__back__retraction	-1.27637061
0days	ID_10	left__back__retraction	-1.538501377
0days	ID_1	left__back__retraction	0.12559081
0days	ID_2	left__back__retraction	3.889002621
0days	ID_3	left__back__retraction	1.010955045
0days	ID_4	left__back__retraction	-0.341514206
0days	ID_5	left__back__retraction	-8.110801699
0days	ID_6	left__back__retraction	0
0days	ID_7	left__back__retraction	-0.57797182
3days	ID_8	left__back__retraction	-2.819717048
3days	ID_9	left__back__retraction	-0.135272303
3days	ID_10	left__back__retraction	0.460051372
3days	ID_11	left__back__retraction	-2.545966881
3days	ID_12	left__back__retraction	-2.757772999
3days	ID_13	left__back__retraction	-1.293005832
3days	ID_1	left__back__retraction	-1.836321935
3days	ID_2	left__back__retraction	1.001731981
7days	ID_3	left__back__retraction	0.13659226
7days	ID_4	left__back__retraction	0.549676978
7days	ID_5	left__back__retraction	-3.194304685
7days	ID_6	left__back__retraction	-2.038100464
7days	ID_7	left__back__retraction	-2.368477429
7days	ID_8	left__back__retraction	-2.694002504
7days	ID_1	left__back__retraction	-6.256429814
7days	ID_2	left__back__retraction	-4.775949884
7days	ID_3	left__back__retraction	-1.625930648
14days	ID_4	left__back__retraction	1.87976914
14days	ID_5	left__back__retraction	-2.587458199
14days	ID_6	left__back__retraction	-0.883775699
14days	ID_7	left__back__retraction	-1.009640645
14days	ID_8	left__back__retraction	-1.44399568
14days	ID_9	left__back__retraction	-8.1193916
14days	ID_1	left__back__retraction	-2.842384321

14days	ID_2	left__back__retraction	-2.638135661
14days	ID_3	left__back__retraction	0.80479594
14days	ID_4	left__back__retraction	-5.799396098
14days	ID_5	left__back__retraction	-0.789313388
21days	ID_6	left__back__retraction	0.243836979
21days	ID_7	left__back__retraction	-2.424044339
21days	ID_8	left__back__retraction	-6.781224612
21days	ID_9	left__back__retraction	-0.216298896
21days	ID_10	left__back__retraction	-0.419762593
21days	ID_11	left__back__retraction	-0.177530285
21days	ID_1	left__back__retraction	-0.789324573
21days	ID_2	left__back__retraction	1.585602295
21days	ID_3	left__back__retraction	2.569213531
21days	ID_4	left__back__retraction	-0.980244494
0days	ID_1	left__front__average	-0.879607339
0days	ID_2	left__front__average	-0.709980082
0days	ID_3	left__front__average	0
0days	ID_4	left__front__average	0.504144505
0days	ID_5	left__front__average	-1.40961481
0days	ID_6	left__front__average	-0.527251359
0days	ID_7	left__front__average	1.072535671
0days	ID_8	left__front__average	0.448437723
0days	ID_9	left__front__average	0.181167374
0days	ID_10	left__front__average	-0.005719772
0days	ID_11	left__front__average	-3.404842295
0days	ID_12	left__front__average	0.316464062
0days	ID_13	left__front__average	1.607013021
3days	ID_1	left__front__average	-0.907181554
3days	ID_2	left__front__average	-0.401869236
3days	ID_3	left__front__average	0.335036403
3days	ID_4	left__front__average	-2.658869037
3days	ID_5	left__front__average	-2.227689565
3days	ID_6	left__front__average	-0.721798769
3days	ID_7	left__front__average	0.585521939
3days	ID_8	left__front__average	-0.258398494
7days	ID_1	left__front__average	0.025801317
7days	ID_2	left__front__average	-1.214567938
7days	ID_3	left__front__average	-1.002909718
7days	ID_4	left__front__average	-1.739412223
7days	ID_5	left__front__average	0.549012538
7days	ID_6	left__front__average	-3.108332693
7days	ID_7	left__front__average	-1.714345392
7days	ID_8	left__front__average	0.232114186
7days	ID_9	left__front__average	-0.193090236
14days	ID_1	left__front__average	1.910567496
14days	ID_2	left__front__average	0.082685496
14days	ID_3	left__front__average	-1.241697683
14days	ID_4	left__front__average	-1.110237164

14days	ID_5	left__front__average	-0.247411429
14days	ID_6	left__front__average	-1.310406232
14days	ID_7	left__front__average	-1.554612921
14days	ID_8	left__front__average	-1.724099018
14days	ID_9	left__front__average	0.254252606
14days	ID_10	left__front__average	0.237044329
14days	ID_11	left__front__average	0.660205908
21days	ID_1	left__front__average	-0.540968367
21days	ID_2	left__front__average	-0.860216841
21days	ID_3	left__front__average	-1.483103708
21days	ID_4	left__front__average	0.362900494
21days	ID_5	left__front__average	-0.284639455
21days	ID_6	left__front__average	-3.403529073
21days	ID_7	left__front__average	0.910377542
21days	ID_8	left__front__average	-0.930189315
21days	ID_9	left__front__average	-1.665121787
21days	ID_10	left__front__average	0.083718823
0days	ID_1	left__front__median	-0.850321644
0days	ID_2	left__front__median	0
0days	ID_3	left__front__median	0.281198903
0days	ID_4	left__front__median	0.15545647
0days	ID_5	left__front__median	-1.272624197
0days	ID_6	left__front__median	-0.915424142
0days	ID_7	left__front__median	0.927347784
0days	ID_8	left__front__median	1.163047102
0days	ID_9	left__front__median	-1.034528229
0days	ID_10	left__front__median	-0.029419574
0days	ID_11	left__front__median	-4.107992567
0days	ID_12	left__front__median	0.239413114
0days	ID_13	left__front__median	2.592267089
3days	ID_1	left__front__median	-0.958318096
3days	ID_2	left__front__median	-1.267064368
3days	ID_3	left__front__median	-1.021439728
3days	ID_4	left__front__median	-2.699232424
3days	ID_5	left__front__median	-2.323193601
3days	ID_6	left__front__median	-1.015288122
3days	ID_7	left__front__median	0.995621417
3days	ID_8	left__front__median	-0.428999883
7days	ID_1	left__front__median	-0.332171623
7days	ID_2	left__front__median	-0.318934555
7days	ID_3	left__front__median	-0.968282335
7days	ID_4	left__front__median	-0.770697385
7days	ID_5	left__front__median	0.724418545
7days	ID_6	left__front__median	-3.629760179
7days	ID_7	left__front__median	-1.833860047
7days	ID_8	left__front__median	0.629543459
7days	ID_9	left__front__median	1.712253759
14days	ID_1	left__front__median	2.191838668

14days	ID_2	left__front__median	-0.120205915
14days	ID_3	left__front__median	-0.697275341
14days	ID_4	left__front__median	-1.74039099
14days	ID_5	left__front__median	0.386450839
14days	ID_6	left__front__median	-1.765467357
14days	ID_7	left__front__median	-1.946032421
14days	ID_8	left__front__median	-1.83085407
14days	ID_9	left__front__median	0.234359659
14days	ID_10	left__front__median	0.771166045
14days	ID_11	left__front__median	0.782629212
21days	ID_1	left__front__median	-0.75497561
21days	ID_2	left__front__median	-1.1884572
21days	ID_3	left__front__median	-1.419486097
21days	ID_4	left__front__median	-0.349997282
21days	ID_5	left__front__median	-0.828709842
21days	ID_6	left__front__median	-4.917535076
21days	ID_7	left__front__median	2.072451757
21days	ID_8	left__front__median	-0.071091841
21days	ID_9	left__front__median	-2.686558747
21days	ID_10	left__front__median	0.053536251
0days	ID_1	left__front__protraction	-0.431356953
0days	ID_2	left__front__protraction	-2.219015636
0days	ID_3	left__front__protraction	-0.255317951
0days	ID_4	left__front__protraction	0
0days	ID_5	left__front__protraction	0.094228582
0days	ID_6	left__front__protraction	1.451605376
0days	ID_7	left__front__protraction	1.151527795
0days	ID_8	left__front__protraction	-1.429568036
0days	ID_9	left__front__protraction	1.495405382
0days	ID_10	left__front__protraction	-0.122580362
0days	ID_11	left__front__protraction	-1.54287188
0days	ID_12	left__front__protraction	0.769433412
0days	ID_13	left__front__protraction	1.832993513
3days	ID_1	left__front__protraction	0.909095638
3days	ID_2	left__front__protraction	1.372744342
3days	ID_3	left__front__protraction	1.438993147
3days	ID_4	left__front__protraction	-2.392774837
3days	ID_5	left__front__protraction	-1.703263852
3days	ID_6	left__front__protraction	-0.216873025
3days	ID_7	left__front__protraction	0.550482385
3days	ID_8	left__front__protraction	0.289721795
7days	ID_1	left__front__protraction	0.172296956
7days	ID_2	left__front__protraction	-1.681786091
7days	ID_3	left__front__protraction	0.198160508
7days	ID_4	left__front__protraction	-0.937126705
7days	ID_5	left__front__protraction	-0.315310416
7days	ID_6	left__front__protraction	-1.431788271
7days	ID_7	left__front__protraction	1.069687936

7days	ID_8	left__front__protraction	0.72263386
7days	ID_9	left__front__protraction	-0.315511619
14days	ID_1	left__front__protraction	0.154797216
14days	ID_2	left__front__protraction	1.708096434
14days	ID_3	left__front__protraction	-2.185781336
14days	ID_4	left__front__protraction	0.065733988
14days	ID_5	left__front__protraction	0.617927104
14days	ID_6	left__front__protraction	0.325474586
14days	ID_7	left__front__protraction	0.869700545
14days	ID_8	left__front__protraction	1.07357953
14days	ID_9	left__front__protraction	0.432166648
14days	ID_10	left__front__protraction	1.361641015
14days	ID_11	left__front__protraction	1.624899738
21days	ID_1	left__front__protraction	-0.984004383
21days	ID_2	left__front__protraction	1.04709775
21days	ID_3	left__front__protraction	1.55130306
21days	ID_4	left__front__protraction	1.78538358
21days	ID_5	left__front__protraction	1.463509028
21days	ID_6	left__front__protraction	-1.253657421
21days	ID_7	left__front__protraction	1.223119841
21days	ID_8	left__front__protraction	0.735382676
21days	ID_9	left__front__protraction	0.537097742
21days	ID_10	left__front__protraction	1.588416205
0days	ID_1	left__front__retraction	-0.449184075
0days	ID_2	left__front__retraction	0.032254181
0days	ID_3	left__front__retraction	0.243039985
0days	ID_4	left__front__retraction	0.394707929
0days	ID_5	left__front__retraction	-3.266687878
0days	ID_6	left__front__retraction	-2.29195909
0days	ID_7	left__front__retraction	0
0days	ID_8	left__front__retraction	2.031415473
0days	ID_9	left__front__retraction	-0.332777796
0days	ID_10	left__front__retraction	0.089530468
0days	ID_11	left__front__retraction	-4.599697686
0days	ID_12	left__front__retraction	-1.017194709
0days	ID_13	left__front__retraction	0.094253216
3days	ID_1	left__front__retraction	-3.503634286
3days	ID_2	left__front__retraction	-1.644096679
3days	ID_3	left__front__retraction	0.518083081
3days	ID_4	left__front__retraction	-3.413815571
3days	ID_5	left__front__retraction	-2.765435548
3days	ID_6	left__front__retraction	-1.516642635
3days	ID_7	left__front__retraction	-0.328488286
3days	ID_8	left__front__retraction	-1.219683644
7days	ID_1	left__front__retraction	-0.311802376
7days	ID_2	left__front__retraction	-0.796664338
7days	ID_3	left__front__retraction	-2.146841945
7days	ID_4	left__front__retraction	-2.380134239

7days	ID_5	left__front__retraction	-0.074984075
7days	ID_6	left__front__retraction	-4.494462827
7days	ID_7	left__front__retraction	-5.45622217
7days	ID_8	left__front__retraction	-0.965980117
7days	ID_9	left__front__retraction	-2.086637506
14days	ID_1	left__front__retraction	2.140659653
14days	ID_2	left__front__retraction	-1.73669188
14days	ID_3	left__front__retraction	-0.430918492
14days	ID_4	left__front__retraction	-1.647172503
14days	ID_5	left__front__retraction	-2.652616405
14days	ID_6	left__front__retraction	-2.955103925
14days	ID_7	left__front__retraction	-2.613589569
14days	ID_8	left__front__retraction	-4.162630955
14days	ID_9	left__front__retraction	-0.28831661
14days	ID_10	left__front__retraction	0.024024928
14days	ID_11	left__front__retraction	-1.671537507
21days	ID_1	left__front__retraction	-0.2012184
21days	ID_2	left__front__retraction	-2.696259895
21days	ID_3	left__front__retraction	-5.184760652
21days	ID_4	left__front__retraction	-0.499028704
21days	ID_5	left__front__retraction	-0.968420668
21days	ID_6	left__front__retraction	-3.540820983
21days	ID_7	left__front__retraction	-2.347366179
21days	ID_8	left__front__retraction	-2.202341262
21days	ID_9	left__front__retraction	-1.636486244
21days	ID_10	left__front__retraction	-1.354557853
0days	ID_1	right__back__average	-1.220707321
0days	ID_2	right__back__average	0
0days	ID_3	right__back__average	-2.597076385
0days	ID_4	right__back__average	-0.108458564
0days	ID_5	right__back__average	-1.584014298
0days	ID_6	right__back__average	-0.16124635
0days	ID_7	right__back__average	2.241302811
0days	ID_8	right__back__average	5.920478421
0days	ID_9	right__back__average	0.89439213
0days	ID_10	right__back__average	2.723608993
0days	ID_11	right__back__average	-2.645229343
0days	ID_12	right__back__average	1.305643589
0days	ID_13	right__back__average	0.691280553
3days	ID_1	right__back__average	-4.532717748
3days	ID_2	right__back__average	-3.159389207
3days	ID_3	right__back__average	-3.361433109
3days	ID_4	right__back__average	-2.602608926
3days	ID_5	right__back__average	-1.691631756
3days	ID_6	right__back__average	-1.028984944
3days	ID_7	right__back__average	-0.954060279
3days	ID_8	right__back__average	1.085135535
7days	ID_1	right__back__average	-0.944070368

7days	ID_2	right__back__average	0.427358733
7days	ID_3	right__back__average	-0.487637607
7days	ID_4	right__back__average	2.39815611
7days	ID_5	right__back__average	0.701435722
7days	ID_6	right__back__average	2.165795863
7days	ID_7	right__back__average	-2.410576257
7days	ID_8	right__back__average	0.774297871
7days	ID_9	right__back__average	-0.245413339
14days	ID_1	right__back__average	-1.060872574
14days	ID_2	right__back__average	-2.184781052
14days	ID_3	right__back__average	1.943203888
14days	ID_4	right__back__average	2.568507429
14days	ID_5	right__back__average	0.612213986
14days	ID_6	right__back__average	-3.290772364
14days	ID_7	right__back__average	-0.006108082
14days	ID_8	right__back__average	-0.037379197
14days	ID_9	right__back__average	1.848193881
14days	ID_10	right__back__average	2.666578077
14days	ID_11	right__back__average	2.549472541
21days	ID_1	right__back__average	-0.78674041
21days	ID_2	right__back__average	0.931724259
21days	ID_3	right__back__average	0.975036331
21days	ID_4	right__back__average	2.303445472
21days	ID_5	right__back__average	1.324666077
21days	ID_6	right__back__average	2.533424963
21days	ID_7	right__back__average	3.006823535
21days	ID_8	right__back__average	0.58944103
21days	ID_9	right__back__average	1.727858666
21days	ID_10	right__back__average	1.771106012
0days	ID_1	right__back__median	-1.830371539
0days	ID_2	right__back__median	-1.312863594
0days	ID_3	right__back__median	-3.319817877
0days	ID_4	right__back__median	-0.469532213
0days	ID_5	right__back__median	-1.245295153
0days	ID_6	right__back__median	0
0days	ID_7	right__back__median	2.680109103
0days	ID_8	right__back__median	6.497710752
0days	ID_9	right__back__median	0.790934626
0days	ID_10	right__back__median	2.968405315
0days	ID_11	right__back__median	-3.894430257
0days	ID_12	right__back__median	0.788309133
0days	ID_13	right__back__median	0.520071148
3days	ID_1	right__back__median	-6.962627438
3days	ID_2	right__back__median	-4.178970217
3days	ID_3	right__back__median	-4.405582381
3days	ID_4	right__back__median	-3.12863867
3days	ID_5	right__back__median	-3.626008271
3days	ID_6	right__back__median	-2.320210661

3days	ID_7	right__back__median	-0.367657838
3days	ID_8	right__back__median	2.149825638
7days	ID_1	right__back__median	-0.520040288
7days	ID_2	right__back__median	0.56389815
7days	ID_3	right__back__median	-0.903462467
7days	ID_4	right__back__median	2.806679172
7days	ID_5	right__back__median	0.640917201
7days	ID_6	right__back__median	4.375360868
7days	ID_7	right__back__median	0.258314772
7days	ID_8	right__back__median	1.74841411
7days	ID_9	right__back__median	1.949187482
14days	ID_1	right__back__median	-1.336873786
14days	ID_2	right__back__median	-3.613745458
14days	ID_3	right__back__median	2.698669552
14days	ID_4	right__back__median	3.631003844
14days	ID_5	right__back__median	2.291049403
14days	ID_6	right__back__median	-4.093526801
14days	ID_7	right__back__median	2.034673739
14days	ID_8	right__back__median	1.972429748
14days	ID_9	right__back__median	2.663750211
14days	ID_10	right__back__median	4.397476978
14days	ID_11	right__back__median	2.572437652
21days	ID_1	right__back__median	-0.792845149
21days	ID_2	right__back__median	0.734144003
21days	ID_3	right__back__median	0.862819433
21days	ID_4	right__back__median	4.263139462
21days	ID_5	right__back__median	2.315868584
21days	ID_6	right__back__median	3.80544907
21days	ID_7	right__back__median	3.04927329
21days	ID_8	right__back__median	2.234963897
21days	ID_9	right__back__median	3.362991192
21days	ID_10	right__back__median	1.786609545
0days	ID_1	right__back__protraction	-1.421076867
0days	ID_2	right__back__protraction	-0.500102876
0days	ID_3	right__back__protraction	-2.286100223
0days	ID_4	right__back__protraction	0
0days	ID_5	right__back__protraction	-0.220407759
0days	ID_6	right__back__protraction	0.881491698
0days	ID_7	right__back__protraction	2.694013764
0days	ID_8	right__back__protraction	2.882976574
0days	ID_9	right__back__protraction	-0.369787074
0days	ID_10	right__back__protraction	3.616231241
0days	ID_11	right__back__protraction	-0.380579943
0days	ID_12	right__back__protraction	1.778413854
0days	ID_13	right__back__protraction	1.553195225
3days	ID_1	right__back__protraction	-2.588404577
3days	ID_2	right__back__protraction	-3.125251707
3days	ID_3	right__back__protraction	-4.376133131

3days	ID_4	right__back__protraction	-2.631863487
3days	ID_5	right__back__protraction	0.606737611
3days	ID_6	right__back__protraction	-0.779698286
3days	ID_7	right__back__protraction	0.891683823
3days	ID_8	right__back__protraction	2.392053499
7days	ID_1	right__back__protraction	-1.466488182
7days	ID_2	right__back__protraction	0.32045122
7days	ID_3	right__back__protraction	-0.011322923
7days	ID_4	right__back__protraction	2.701702383
7days	ID_5	right__back__protraction	1.246791091
7days	ID_6	right__back__protraction	3.823594072
7days	ID_7	right__back__protraction	-0.242853724
7days	ID_8	right__back__protraction	1.711197726
7days	ID_9	right__back__protraction	0.846442875
14days	ID_1	right__back__protraction	-1.938068344
14days	ID_2	right__back__protraction	-1.865193
14days	ID_3	right__back__protraction	1.435711494
14days	ID_4	right__back__protraction	2.083280655
14days	ID_5	right__back__protraction	1.251088937
14days	ID_6	right__back__protraction	-0.011532146
14days	ID_7	right__back__protraction	0.883791713
14days	ID_8	right__back__protraction	1.507829887
14days	ID_9	right__back__protraction	3.836127713
14days	ID_10	right__back__protraction	2.288746274
14days	ID_11	right__back__protraction	1.804490211
21days	ID_1	right__back__protraction	0.275301351
21days	ID_2	right__back__protraction	2.943302486
21days	ID_3	right__back__protraction	3.205936847
21days	ID_4	right__back__protraction	3.620898837
21days	ID_5	right__back__protraction	3.365270743
21days	ID_6	right__back__protraction	2.508306541
21days	ID_7	right__back__protraction	3.022708311
21days	ID_8	right__back__protraction	2.153404345
21days	ID_9	right__back__protraction	1.172660773
21days	ID_10	right__back__protraction	2.371177014
0days	ID_1	right__back__retraction	0
0days	ID_2	right__back__retraction	1.416050016
0days	ID_3	right__back__retraction	-1.957660531
0days	ID_4	right__back__retraction	-1.984040499
0days	ID_5	right__back__retraction	-3.312804411
0days	ID_6	right__back__retraction	-1.960314377
0days	ID_7	right__back__retraction	1.678681237
0days	ID_8	right__back__retraction	7.073698638
0days	ID_9	right__back__retraction	0.849269889
0days	ID_10	right__back__retraction	0.812162463
0days	ID_11	right__back__retraction	-2.742840295
0days	ID_12	right__back__retraction	1.100014994
0days	ID_13	right__back__retraction	-0.055768991

3days	ID_1	right__back__retraction	-3.686972257
3days	ID_2	right__back__retraction	-2.869935908
3days	ID_3	right__back__retraction	-0.972749326
3days	ID_4	right__back__retraction	-2.255567001
3days	ID_5	right__back__retraction	-2.232078302
3days	ID_6	right__back__retraction	-0.681708899
3days	ID_7	right__back__retraction	-5.12461956
3days	ID_8	right__back__retraction	-1.350775104
7days	ID_1	right__back__retraction	-0.58724218
7days	ID_2	right__back__retraction	0.787624248
7days	ID_3	right__back__retraction	-0.183581407
7days	ID_4	right__back__retraction	1.530287351
7days	ID_5	right__back__retraction	-0.393741324
7days	ID_6	right__back__retraction	-2.962342971
7days	ID_7	right__back__retraction	-5.638722507
7days	ID_8	right__back__retraction	-2.386434933
7days	ID_9	right__back__retraction	-5.308127703
14days	ID_1	right__back__retraction	-0.207466585
14days	ID_2	right__back__retraction	-2.561717823
14days	ID_3	right__back__retraction	1.112462825
14days	ID_4	right__back__retraction	1.014349941
14days	ID_5	right__back__retraction	-3.386940034
14days	ID_6	right__back__retraction	-6.122774698
14days	ID_7	right__back__retraction	-4.125879163
14days	ID_8	right__back__retraction	-3.498621284
14days	ID_9	right__back__retraction	-1.131703461
14days	ID_10	right__back__retraction	-0.959433361
14days	ID_11	right__back__retraction	1.274775143
21days	ID_1	right__back__retraction	0.054960709
21days	ID_2	right__back__retraction	-0.958421751
21days	ID_3	right__back__retraction	-1.723538194
21days	ID_4	right__back__retraction	-0.415421186
21days	ID_5	right__back__retraction	-1.936323889
21days	ID_6	right__back__retraction	-0.430320046
21days	ID_7	right__back__retraction	1.544883197
21days	ID_8	right__back__retraction	-2.939745193
21days	ID_9	right__back__retraction	-1.126855587
21days	ID_10	right__back__retraction	0.324875024
0days	ID_1	right__front__average	-0.414956268
0days	ID_2	right__front__average	-0.129620843
0days	ID_3	right__front__average	0.021914097
0days	ID_4	right__front__average	0.504541113
0days	ID_5	right__front__average	-1.859685875
0days	ID_6	right__front__average	-0.652199165
0days	ID_7	right__front__average	1.289240568
0days	ID_8	right__front__average	1.10557979
0days	ID_9	right__front__average	0.415070213
0days	ID_10	right__front__average	-3.017275615

0days	ID_11	right__front__average	-0.70525656
0days	ID_12	right__front__average	0
0days	ID_13	right__front__average	0.936034643
3days	ID_1	right__front__average	-1.847480913
3days	ID_2	right__front__average	-1.006830148
3days	ID_3	right__front__average	-0.519916629
3days	ID_4	right__front__average	-2.375509092
3days	ID_5	right__front__average	-1.133696988
3days	ID_6	right__front__average	-1.479573856
3days	ID_7	right__front__average	-1.795672612
3days	ID_8	right__front__average	0.225308201
7days	ID_1	right__front__average	0.03472778
7days	ID_2	right__front__average	0.042579331
7days	ID_3	right__front__average	-2.374681633
7days	ID_4	right__front__average	-0.817392494
7days	ID_5	right__front__average	-2.633270102
7days	ID_6	right__front__average	-1.599808714
7days	ID_7	right__front__average	-1.862385246
7days	ID_8	right__front__average	-2.367818189
7days	ID_9	right__front__average	-1.35271496
14days	ID_1	right__front__average	1.3860377
14days	ID_2	right__front__average	-2.395939268
14days	ID_3	right__front__average	0.305387048
14days	ID_4	right__front__average	-1.726435765
14days	ID_5	right__front__average	-2.023496358
14days	ID_6	right__front__average	-3.476992686
14days	ID_7	right__front__average	-1.318233544
14days	ID_8	right__front__average	-1.365925182
14days	ID_9	right__front__average	-0.455525058
14days	ID_10	right__front__average	-0.109342186
14days	ID_11	right__front__average	0.568691943
21days	ID_1	right__front__average	-0.16368815
21days	ID_2	right__front__average	-3.077911527
21days	ID_3	right__front__average	-3.423564686
21days	ID_4	right__front__average	0.697291126
21days	ID_5	right__front__average	-3.322927206
21days	ID_6	right__front__average	-0.251843726
21days	ID_7	right__front__average	-1.335368763
21days	ID_8	right__front__average	0.031217492
21days	ID_9	right__front__average	-0.198973876
21days	ID_10	right__front__average	-0.798304955
0days	ID_1	right__front__median	-0.168571973
0days	ID_2	right__front__median	-0.826783694
0days	ID_3	right__front__median	-0.385938075
0days	ID_4	right__front__median	0.431279483
0days	ID_5	right__front__median	-1.101756862
0days	ID_6	right__front__median	0.5611322
0days	ID_7	right__front__median	1.315754519

0days	ID_8	right__front__median	0.366439149
0days	ID_9	right__front__median	1.503179119
0days	ID_10	right__front__median	-2.93875279
0days	ID_11	right__front__median	-1.669213039
0days	ID_12	right__front__median	0
0days	ID_13	right__front__median	1.570389115
3days	ID_1	right__front__median	-2.416762596
3days	ID_2	right__front__median	-1.391173985
3days	ID_3	right__front__median	-0.744887037
3days	ID_4	right__front__median	-3.806978384
3days	ID_5	right__front__median	-2.725821269
3days	ID_6	right__front__median	-3.085073207
3days	ID_7	right__front__median	-1.90808633
3days	ID_8	right__front__median	1.319839772
7days	ID_1	right__front__median	0.017742105
7days	ID_2	right__front__median	-0.909505354
7days	ID_3	right__front__median	-3.417498827
7days	ID_4	right__front__median	-1.868867582
7days	ID_5	right__front__median	-4.705078082
7days	ID_6	right__front__median	-3.174683055
7days	ID_7	right__front__median	-2.608414205
7days	ID_8	right__front__median	-3.375109376
7days	ID_9	right__front__median	-0.989122911
14days	ID_1	right__front__median	1.42538104
14days	ID_2	right__front__median	-4.247891766
14days	ID_3	right__front__median	-0.431757111
14days	ID_4	right__front__median	-2.746476822
14days	ID_5	right__front__median	-2.517615182
14days	ID_6	right__front__median	-4.421366903
14days	ID_7	right__front__median	-2.217291703
14days	ID_8	right__front__median	-2.385538486
14days	ID_9	right__front__median	-0.035296915
14days	ID_10	right__front__median	0.673202659
14days	ID_11	right__front__median	-0.211709649
21days	ID_1	right__front__median	-0.234139268
21days	ID_2	right__front__median	-4.916247242
21days	ID_3	right__front__median	-4.778671159
21days	ID_4	right__front__median	0.80778347
21days	ID_5	right__front__median	-5.618115113
21days	ID_6	right__front__median	-0.706095552
21days	ID_7	right__front__median	-1.9772455
21days	ID_8	right__front__median	0.147007758
21days	ID_9	right__front__median	0.13535427
21days	ID_10	right__front__median	-1.132010508
0days	ID_1	right__front__protraction	-1.512247084
0days	ID_2	right__front__protraction	0
0days	ID_3	right__front__protraction	-0.329057487
0days	ID_4	right__front__protraction	0.189957542

0days	ID_5	right__front__protraction	-1.519248755
0days	ID_6	right__front__protraction	-0.757627436
0days	ID_7	right__front__protraction	1.766036297
0days	ID_8	right__front__protraction	0.089914395
0days	ID_9	right__front__protraction	-0.782273392
0days	ID_10	right__front__protraction	-1.977281229
0days	ID_11	right__front__protraction	0.124241544
0days	ID_12	right__front__protraction	0.812229566
0days	ID_13	right__front__protraction	1.385868305
3days	ID_1	right__front__protraction	-0.012704658
3days	ID_2	right__front__protraction	-0.154354055
3days	ID_3	right__front__protraction	-0.407589008
3days	ID_4	right__front__protraction	-0.755500501
3days	ID_5	right__front__protraction	0.164153271
3days	ID_6	right__front__protraction	-0.439168329
3days	ID_7	right__front__protraction	-0.94982165
3days	ID_8	right__front__protraction	-1.08244552
7days	ID_1	right__front__protraction	-0.976820236
7days	ID_2	right__front__protraction	0.183013131
7days	ID_3	right__front__protraction	-1.375000616
7days	ID_4	right__front__protraction	0.225423235
7days	ID_5	right__front__protraction	-1.557079569
7days	ID_6	right__front__protraction	0.996728966
7days	ID_7	right__front__protraction	-1.045804756
7days	ID_8	right__front__protraction	0.608472236
7days	ID_9	right__front__protraction	-1.928753953
14days	ID_1	right__front__protraction	-0.236254125
14days	ID_2	right__front__protraction	-1.52548254
14days	ID_3	right__front__protraction	0.279243327
14days	ID_4	right__front__protraction	0.412871035
14days	ID_5	right__front__protraction	-0.935036379
14days	ID_6	right__front__protraction	-1.142357874
14days	ID_7	right__front__protraction	1.62375621
14days	ID_8	right__front__protraction	0.960321015
14days	ID_9	right__front__protraction	-1.384088641
14days	ID_10	right__front__protraction	1.544552256
14days	ID_11	right__front__protraction	2.948871686
21days	ID_1	right__front__protraction	-0.4977671
21days	ID_2	right__front__protraction	-1.257713203
21days	ID_3	right__front__protraction	-0.595169044
21days	ID_4	right__front__protraction	1.832973045
21days	ID_5	right__front__protraction	-0.476463466
21days	ID_6	right__front__protraction	0.927586227
21days	ID_7	right__front__protraction	-0.590027022
21days	ID_8	right__front__protraction	1.68678415
21days	ID_9	right__front__protraction	0.159135913
21days	ID_10	right__front__protraction	1.020922517
0days	ID_1	right__front__retraction	-0.93066662

0days	ID_2	right__front__retraction	0.44705624
0days	ID_3	right__front__retraction	-0.60513673
0days	ID_4	right__front__retraction	0.58771033
0days	ID_5	right__front__retraction	-3.259730399
0days	ID_6	right__front__retraction	-2.044834264
0days	ID_7	right__front__retraction	1.03871289
0days	ID_8	right__front__retraction	1.992417761
0days	ID_9	right__front__retraction	0
0days	ID_10	right__front__retraction	-2.937163791
0days	ID_11	right__front__retraction	0.203480725
0days	ID_12	right__front__retraction	-0.215854572
0days	ID_13	right__front__retraction	0.294718757
3days	ID_1	right__front__retraction	-2.038213048
3days	ID_2	right__front__retraction	-0.407148309
3days	ID_3	right__front__retraction	0.750361345
3days	ID_4	right__front__retraction	-2.18971475
3days	ID_5	right__front__retraction	-1.678337059
3days	ID_6	right__front__retraction	-0.37815693
3days	ID_7	right__front__retraction	-2.078306624
3days	ID_8	right__front__retraction	1.173070963
7days	ID_1	right__front__retraction	1.836241544
7days	ID_2	right__front__retraction	0.752301178
7days	ID_3	right__front__retraction	-1.538627901
7days	ID_4	right__front__retraction	-1.651799997
7days	ID_5	right__front__retraction	-1.623444265
7days	ID_6	right__front__retraction	-4.187294494
7days	ID_7	right__front__retraction	-0.550974868
7days	ID_8	right__front__retraction	-3.731654882
7days	ID_9	right__front__retraction	-0.706718797
14days	ID_1	right__front__retraction	2.816010627
14days	ID_2	right__front__retraction	-1.488736399
14days	ID_3	right__front__retraction	0.471879453
14days	ID_4	right__front__retraction	-2.837391993
14days	ID_5	right__front__retraction	-3.58770493
14days	ID_6	right__front__retraction	-4.199274051
14days	ID_7	right__front__retraction	-2.868433603
14days	ID_8	right__front__retraction	-2.479819801
14days	ID_9	right__front__retraction	0.870701604
14days	ID_10	right__front__retraction	-2.344768186
14days	ID_11	right__front__retraction	-0.964389038
21days	ID_1	right__front__retraction	0.92750539
21days	ID_2	right__front__retraction	-3.021517049
21days	ID_3	right__front__retraction	-4.12117692
21days	ID_4	right__front__retraction	-0.895782426
21days	ID_5	right__front__retraction	-3.664846613
21days	ID_6	right__front__retraction	-1.429468384
21days	ID_7	right__front__retraction	-1.27661404
21days	ID_8	right__front__retraction	-1.132579016

21days	ID_9	right__front__retraction	-1.847145473
21days	ID_10	right__front__retraction	-1.66798754

Suppl. Table 23: Summary of side perspective horizontal analysis

day	Measure	Mean	SD
0days	left__back__	0	2.354541209
0days	left__back__	0	3.227139569
0days	left__back__	0	1.69170068
0days	left__back__	0	2.846657111
0days	left__front__	0	1.253349506
0days	left__front__	0	1.570427129
0days	left__front__	0	1.264781286
0days	left__front__	0	1.743007692
0days	right__back_	0	2.344531176
0days	right__back_	0	2.77491306
0days	right__back_	0	1.770511725
0days	right__back_	0	2.700298056
0days	right__front_	0	1.203423662
0days	right__front_	0	1.305458505
0days	right__front_	0	1.12076202
0days	right__front_	0	1.5320941
3days	left__back__	-1.822015073	1.929090297
3days	left__back__	-4.080316287	3.126680302
3days	left__back__	-1.255989136	1.71114298
3days	left__back__	-1.564663883	1.511813249
3days	left__front__	-0.561834002	1.144458372
3days	left__front__	-1.018363925	1.131125044
3days	left__front__	0.42010209	1.405823096
3days	left__front__	-1.580369657	1.433498932
3days	right__back_	-2.147120341	1.757349752
3days	right__back_	-3.377323471	2.757335366
3days	right__back_	-1.684051431	2.344941218
3days	right__back_	-2.243822651	1.485169903
3days	right__front_	-1.306635422	0.824263826
3days	right__front_	-2.162424463	1.600281066
3days	right__front_	-0.423378668	0.447325136
3days	right__front_	-1.042742684	1.33491689
7days	left__back__	-2.317728529	0.643078789
7days	left__back__	-3.443448227	1.062367573
7days	left__back__	-2.07934681	0.764617288
7days	left__back__	-2.368477429	2.152067839
7days	left__front__	-1.002909718	1.17748234
7days	left__front__	-0.332171623	1.564770365
7days	left__front__	-0.315310416	0.934484209
7days	left__front__	-2.086637506	1.84924638
7days	right__back_	0.427358733	1.503626524
7days	right__back_	0.640917201	1.677492813
7days	right__back_	0.846442875	1.603551536
7days	right__back_	-0.58724218	2.560789847
7days	right__front_	-1.599808714	1.008113666

7days	right__front_	-2.608414205	1.510248011
7days	right__front_	-0.976820236	1.054519984
7days	right__front_	-1.538627901	1.919825549
14days	left__back__	-1.453731628	2.656271657
14days	left__back__	-1.659057825	3.988066508
14days	left__back__	-1.607006233	1.527604957
14days	left__back__	-1.44399568	2.82983814
14days	left__front__	-0.247411429	1.125586933
14days	left__front__	-0.120205915	1.368714832
14days	left__front__	0.617927104	1.07125209
14days	left__front__	-1.671537507	1.733422223
14days	right__back_	0.612213986	2.040695014
14days	right__back_	2.291049403	2.872608367
14days	right__back_	1.435711494	1.729372995
14days	right__back_	-1.131703461	2.439141909
14days	right__front_	-1.318233544	1.44321152
14days	right__front_	-2.217291703	1.953600325
14days	right__front_	0.279243327	1.441428049
14days	right__front_	-2.344768186	2.130770982
21days	left__back__	0.116947838	1.433482162
21days	left__back__	0.387687533	1.950397104
21days	left__back__	0.55295167	0.542495908
21days	left__back__	-0.318030745	2.525711836
21days	left__front__	-0.700592604	1.220282587
21days	left__front__	-0.791842726	1.835661276
21days	left__front__	1.135108796	1.069862785
21days	left__front__	-1.919413753	1.499065916
21days	right__back_	1.526262371	1.093320778
21days	right__back_	2.275416241	1.562512757
21days	right__back_	2.725804514	1.043927813
21days	right__back_	-0.694370898	1.274467572
21days	right__front_	-0.52507434	1.536426947
21days	right__front_	-0.91905303	2.394837165
21days	right__front_	-0.158663776	1.075149523
21days	right__front_	-1.548727962	1.471551016

Suppl. Table 24: Statistical test of bside perspective horizontal analysis

Measur	group1	group2	p.adj	sig	Test*
right__b	0days	3days	0.001853114	**	Repeat ANOVA + PH test
left__bac	0days	3days	0.008909729	**	Repeat ANOVA + PH test
right__b	0days	3days	0.016891748	*	Repeat ANOVA + PH test
left__bac	0days	14days	0.017744158	*	Repeat ANOVA + PH test
right__b	0days	3days	0.017941213	*	Repeat ANOVA + PH test
right__fr	0days	7days	0.029066542	*	Repeat ANOVA + PH test
left__bac	0days	7days	0.034047886	*	Repeat ANOVA + PH test
left__bac	0days	7days	0.034277897	*	Repeat ANOVA + PH test
right__b	0days	21days	0.040234352	*	Repeat ANOVA + PH test
left__bac	0days	7days	0.048678422	*	Repeat ANOVA + PH test
left__bac	0days	14days	0.057031105	*	Repeat ANOVA + PH test
right__b	0days	7days	0.075050598	*	Repeat ANOVA + PH test
right__b	0days	14days	0.075050598	*	Repeat ANOVA + PH test
right__b	0days	21days	0.092891088	*	Repeat ANOVA + PH test
right__fr	0days	3days	0.097701518	*	Repeat ANOVA + PH test
right__fr	0days	14days	0.097701518	*	Repeat ANOVA + PH test
right__fr	0days	21days	0.097701518	*	Repeat ANOVA + PH test
right__b	0days	3days	0.099814864	*	Repeat ANOVA + PH test
left__bac	0days	3days	0.148248421	ns	Repeat ANOVA + PH test
left__bac	0days	14days	0.148248421	ns	Repeat ANOVA + PH test
right__b	0days	21days	0.302434764	ns	Repeat ANOVA + PH test
right__fr	0days	21days	0.334110592	ns	Repeat ANOVA + PH test
left__fror	0days	7days	0.361568073	ns	Repeat ANOVA + PH test
left__fror	0days	21days	0.361568073	ns	Repeat ANOVA + PH test
left__bac	0days	21days	0.367291339	ns	Repeat ANOVA + PH test
right__b	0days	7days	0.375270127	ns	Repeat ANOVA + PH test
right__b	0days	14days	0.375270127	ns	Repeat ANOVA + PH test
left__bac	0days	7days	0.406980531	ns	Repeat ANOVA + PH test
left__bac	0days	21days	0.406980531	ns	Repeat ANOVA + PH test
left__fror	0days	3days	0.457508352	ns	Repeat ANOVA + PH test
left__fror	0days	14days	0.457508352	ns	Repeat ANOVA + PH test
right__fr	0days	14days	0.492138568	ns	Repeat ANOVA + PH test
left__bac	0days	3days	0.526400626	ns	Repeat ANOVA + PH test
left__bac	0days	14days	0.526400626	ns	Repeat ANOVA + PH test
left__bac	0days	3days	0.536024904	ns	Repeat ANOVA + PH test
right__fr	0days	7days	0.53778735	ns	Repeat ANOVA + PH test
left__bac	0days	21days	0.549397538	ns	Repeat ANOVA + PH test
left__bac	0days	21days	0.574923688	ns	Repeat ANOVA + PH test
right__fr	0days	3days	0.613412544	ns	Repeat ANOVA + PH test
right__fr	0days	7days	0.613412544	ns	Repeat ANOVA + PH test
right__b	0days	21days	0.620197338	ns	Repeat ANOVA + PH test
right__fr	0days	3days	0.655804103	ns	Repeat ANOVA + PH test
right__fr	0days	14days	0.655804103	ns	Repeat ANOVA + PH test
right__fr	0days	21days	0.655804103	ns	Repeat ANOVA + PH test
left__fror	0days	3days	1	ns	Repeat ANOVA + PH test

left__fror	0days	7days	1	ns	Repeat ANOVA + PH test
left__fror	0days	14days	1	ns	Repeat ANOVA + PH test
left__fror	0days	21days	1	ns	Repeat ANOVA + PH test
left__fror	0days	3days	1	ns	Repeat ANOVA + PH test
left__fror	0days	7days	1	ns	Repeat ANOVA + PH test
left__fror	0days	14days	1	ns	Repeat ANOVA + PH test
left__fror	0days	21days	1	ns	Repeat ANOVA + PH test
left__fror	0days	3days	1	ns	Repeat ANOVA + PH test
left__fror	0days	7days	1	ns	Repeat ANOVA + PH test
left__fror	0days	14days	1	ns	Repeat ANOVA + PH test
left__fror	0days	21days	1	ns	Repeat ANOVA + PH test
right__ba	0days	7days	1	ns	Repeat ANOVA + PH test
right__ba	0days	14days	1	ns	Repeat ANOVA + PH test
right__ba	0days	7days	1	ns	Repeat ANOVA + PH test
right__ba	0days	14days	1	ns	Repeat ANOVA + PH test
right__fr	0days	3days	1	ns	Repeat ANOVA + PH test
right__fr	0days	7days	1	ns	Repeat ANOVA + PH test
right__fr	0days	14days	1	ns	Repeat ANOVA + PH test
right__fr	0days	21days	1	ns	Repeat ANOVA + PH test

Suppl. Table 25: Raw data side perspective horizontal angles analysis

day	ID	Measure	Angle, °
0days	ID_1	avg_Angle__left__back__hip_ankle_toe	114.1670397
0days	ID_2	avg_Angle__left__back__hip_ankle_toe	120.1753257
0days	ID_3	avg_Angle__left__back__hip_ankle_toe	102.0163458
0days	ID_4	avg_Angle__left__back__hip_ankle_toe	111.8714042
0days	ID_5	avg_Angle__left__back__hip_ankle_toe	109.0708804
0days	ID_6	avg_Angle__left__back__hip_ankle_toe	130.7947873
0days	ID_7	avg_Angle__left__back__hip_ankle_toe	117.6747812
0days	ID_8	avg_Angle__left__back__hip_ankle_toe	130.0793998
0days	ID_9	avg_Angle__left__back__hip_ankle_toe	116.1412727
0days	ID_10	avg_Angle__left__back__hip_ankle_toe	117.8603151
0days	ID_11	avg_Angle__left__back__hip_ankle_toe	128.3921617
0days	ID_12	avg_Angle__left__back__hip_ankle_toe	125.9447653
0days	ID_13	avg_Angle__left__back__hip_ankle_toe	124.2589827
3days	ID_1	avg_Angle__left__back__hip_ankle_toe	117.7780998
3days	ID_2	avg_Angle__left__back__hip_ankle_toe	109.0184176
3days	ID_3	avg_Angle__left__back__hip_ankle_toe	106.8257991
3days	ID_4	avg_Angle__left__back__hip_ankle_toe	118.9717829
3days	ID_5	avg_Angle__left__back__hip_ankle_toe	121.5482061
3days	ID_6	avg_Angle__left__back__hip_ankle_toe	120.6246118
3days	ID_7	avg_Angle__left__back__hip_ankle_toe	104.2305844
3days	ID_8	avg_Angle__left__back__hip_ankle_toe	127.5396905
7days	ID_1	avg_Angle__left__back__hip_ankle_toe	109.4231579
7days	ID_2	avg_Angle__left__back__hip_ankle_toe	106.9625165
7days	ID_3	avg_Angle__left__back__hip_ankle_toe	109.1608043
7days	ID_4	avg_Angle__left__back__hip_ankle_toe	122.5989211
7days	ID_5	avg_Angle__left__back__hip_ankle_toe	109.2314524
7days	ID_6	avg_Angle__left__back__hip_ankle_toe	122.5098544
7days	ID_7	avg_Angle__left__back__hip_ankle_toe	107.4999364
7days	ID_8	avg_Angle__left__back__hip_ankle_toe	118.1545642
7days	ID_9	avg_Angle__left__back__hip_ankle_toe	111.0962299
14days	ID_1	avg_Angle__left__back__hip_ankle_toe	105.6096997
14days	ID_2	avg_Angle__left__back__hip_ankle_toe	106.5926892
14days	ID_3	avg_Angle__left__back__hip_ankle_toe	116.7620652
14days	ID_4	avg_Angle__left__back__hip_ankle_toe	107.810918
14days	ID_5	avg_Angle__left__back__hip_ankle_toe	107.584073
14days	ID_6	avg_Angle__left__back__hip_ankle_toe	109.015013
14days	ID_7	avg_Angle__left__back__hip_ankle_toe	121.4844352
14days	ID_8	avg_Angle__left__back__hip_ankle_toe	132.0615907
14days	ID_9	avg_Angle__left__back__hip_ankle_toe	118.3741866
14days	ID_10	avg_Angle__left__back__hip_ankle_toe	116.3048084
14days	ID_11	avg_Angle__left__back__hip_ankle_toe	116.3657691
21days	ID_1	avg_Angle__left__back__hip_ankle_toe	121.3778167
21days	ID_2	avg_Angle__left__back__hip_ankle_toe	120.9414171
21days	ID_3	avg_Angle__left__back__hip_ankle_toe	117.2057509
21days	ID_4	avg_Angle__left__back__hip_ankle_toe	109.018637
21days	ID_5	avg_Angle__left__back__hip_ankle_toe	110.2264305

21days	ID_6	avg_Angle__left__back__hip_ankle_toe	110.245508
21days	ID_7	avg_Angle__left__back__hip_ankle_toe	115.0626434
21days	ID_8	avg_Angle__left__back__hip_ankle_toe	132.4976511
21days	ID_9	avg_Angle__left__back__hip_ankle_toe	125.3111799
21days	ID_10	avg_Angle__left__back__hip_ankle_toe	130.1877934
0days	ID_1	avg_Angle__left__back__iliac_hip_ankle	135.0505341
0days	ID_2	avg_Angle__left__back__iliac_hip_ankle	135.5793202
0days	ID_3	avg_Angle__left__back__iliac_hip_ankle	144.4318321
0days	ID_4	avg_Angle__left__back__iliac_hip_ankle	135.7760854
0days	ID_5	avg_Angle__left__back__iliac_hip_ankle	133.371106
0days	ID_6	avg_Angle__left__back__iliac_hip_ankle	138.2339967
0days	ID_7	avg_Angle__left__back__iliac_hip_ankle	142.3933013
0days	ID_8	avg_Angle__left__back__iliac_hip_ankle	143.1065826
0days	ID_9	avg_Angle__left__back__iliac_hip_ankle	131.2486022
0days	ID_10	avg_Angle__left__back__iliac_hip_ankle	141.9465582
0days	ID_11	avg_Angle__left__back__iliac_hip_ankle	155.9406815
0days	ID_12	avg_Angle__left__back__iliac_hip_ankle	127.003597
0days	ID_13	avg_Angle__left__back__iliac_hip_ankle	130.4798667
3days	ID_1	avg_Angle__left__back__iliac_hip_ankle	131.4994529
3days	ID_2	avg_Angle__left__back__iliac_hip_ankle	137.002001
3days	ID_3	avg_Angle__left__back__iliac_hip_ankle	170.6036914
3days	ID_4	avg_Angle__left__back__iliac_hip_ankle	139.9021649
3days	ID_5	avg_Angle__left__back__iliac_hip_ankle	145.1201929
3days	ID_6	avg_Angle__left__back__iliac_hip_ankle	163.3106646
3days	ID_7	avg_Angle__left__back__iliac_hip_ankle	157.2863115
3days	ID_8	avg_Angle__left__back__iliac_hip_ankle	145.5540685
7days	ID_1	avg_Angle__left__back__iliac_hip_ankle	148.901809
7days	ID_2	avg_Angle__left__back__iliac_hip_ankle	138.5188622
7days	ID_3	avg_Angle__left__back__iliac_hip_ankle	141.9063018
7days	ID_4	avg_Angle__left__back__iliac_hip_ankle	145.9886662
7days	ID_5	avg_Angle__left__back__iliac_hip_ankle	148.6831878
7days	ID_6	avg_Angle__left__back__iliac_hip_ankle	145.1924703
7days	ID_7	avg_Angle__left__back__iliac_hip_ankle	146.1507297
7days	ID_8	avg_Angle__left__back__iliac_hip_ankle	147.2430243
7days	ID_9	avg_Angle__left__back__iliac_hip_ankle	139.1611706
14days	ID_1	avg_Angle__left__back__iliac_hip_ankle	139.9935782
14days	ID_2	avg_Angle__left__back__iliac_hip_ankle	138.3982793
14days	ID_3	avg_Angle__left__back__iliac_hip_ankle	171.7831316
14days	ID_4	avg_Angle__left__back__iliac_hip_ankle	136.8134784
14days	ID_5	avg_Angle__left__back__iliac_hip_ankle	137.8729391
14days	ID_6	avg_Angle__left__back__iliac_hip_ankle	144.0741428
14days	ID_7	avg_Angle__left__back__iliac_hip_ankle	138.5934962
14days	ID_8	avg_Angle__left__back__iliac_hip_ankle	147.2877546
14days	ID_9	avg_Angle__left__back__iliac_hip_ankle	150.289629
14days	ID_10	avg_Angle__left__back__iliac_hip_ankle	143.6005744
14days	ID_11	avg_Angle__left__back__iliac_hip_ankle	127.0132765
21days	ID_1	avg_Angle__left__back__iliac_hip_ankle	145.752007
21days	ID_2	avg_Angle__left__back__iliac_hip_ankle	134.3066689

21days	ID_3	avg_Angle__left__back__iliac_hip_ankle	131.6968323
21days	ID_4	avg_Angle__left__back__iliac_hip_ankle	134.7369823
21days	ID_5	avg_Angle__left__back__iliac_hip_ankle	132.8104309
21days	ID_6	avg_Angle__left__back__iliac_hip_ankle	138.8536897
21days	ID_7	avg_Angle__left__back__iliac_hip_ankle	138.2547258
21days	ID_8	avg_Angle__left__back__iliac_hip_ankle	135.402751
21days	ID_9	avg_Angle__left__back__iliac_hip_ankle	137.5116714
21days	ID_10	avg_Angle__left__back__iliac_hip_ankle	138.3122277
0days	ID_1	avg_Angle__left__front__elbow_wrist_toetip	144.2891192
0days	ID_2	avg_Angle__left__front__elbow_wrist_toetip	151.1045287
0days	ID_3	avg_Angle__left__front__elbow_wrist_toetip	141.0231684
0days	ID_4	avg_Angle__left__front__elbow_wrist_toetip	140.8208984
0days	ID_5	avg_Angle__left__front__elbow_wrist_toetip	149.9373826
0days	ID_6	avg_Angle__left__front__elbow_wrist_toetip	144.0921415
0days	ID_7	avg_Angle__left__front__elbow_wrist_toetip	142.3731221
0days	ID_8	avg_Angle__left__front__elbow_wrist_toetip	146.789637
0days	ID_9	avg_Angle__left__front__elbow_wrist_toetip	145.8770071
0days	ID_10	avg_Angle__left__front__elbow_wrist_toetip	140.9206528
0days	ID_11	avg_Angle__left__front__elbow_wrist_toetip	130.8326697
0days	ID_12	avg_Angle__left__front__elbow_wrist_toetip	149.3871516
0days	ID_13	avg_Angle__left__front__elbow_wrist_toetip	165.2511709
3days	ID_1	avg_Angle__left__front__elbow_wrist_toetip	146.8133437
3days	ID_2	avg_Angle__left__front__elbow_wrist_toetip	139.6066276
3days	ID_3	avg_Angle__left__front__elbow_wrist_toetip	137.106981
3days	ID_4	avg_Angle__left__front__elbow_wrist_toetip	134.1458412
3days	ID_5	avg_Angle__left__front__elbow_wrist_toetip	148.8868582
3days	ID_6	avg_Angle__left__front__elbow_wrist_toetip	121.7303035
3days	ID_7	avg_Angle__left__front__elbow_wrist_toetip	131.7931426
3days	ID_8	avg_Angle__left__front__elbow_wrist_toetip	137.0905538
7days	ID_1	avg_Angle__left__front__elbow_wrist_toetip	139.3027937
7days	ID_2	avg_Angle__left__front__elbow_wrist_toetip	152.1742211
7days	ID_3	avg_Angle__left__front__elbow_wrist_toetip	148.1289414
7days	ID_4	avg_Angle__left__front__elbow_wrist_toetip	142.5887226
7days	ID_5	avg_Angle__left__front__elbow_wrist_toetip	133.3288446
7days	ID_6	avg_Angle__left__front__elbow_wrist_toetip	139.6149882
7days	ID_7	avg_Angle__left__front__elbow_wrist_toetip	136.1857868
7days	ID_8	avg_Angle__left__front__elbow_wrist_toetip	153.7552099
7days	ID_9	avg_Angle__left__front__elbow_wrist_toetip	135.4245809
14days	ID_1	avg_Angle__left__front__elbow_wrist_toetip	121.5699832
14days	ID_2	avg_Angle__left__front__elbow_wrist_toetip	125.4997733
14days	ID_3	avg_Angle__left__front__elbow_wrist_toetip	130.359543
14days	ID_4	avg_Angle__left__front__elbow_wrist_toetip	136.1223004
14days	ID_5	avg_Angle__left__front__elbow_wrist_toetip	132.2485372
14days	ID_6	avg_Angle__left__front__elbow_wrist_toetip	137.4785996
14days	ID_7	avg_Angle__left__front__elbow_wrist_toetip	149.7871871
14days	ID_8	avg_Angle__left__front__elbow_wrist_toetip	149.0367611
14days	ID_9	avg_Angle__left__front__elbow_wrist_toetip	132.8074463
14days	ID_10	avg_Angle__left__front__elbow_wrist_toetip	133.3898257

14days	ID_11	avg_Angle__left__front__elbow_wrist_toetip	135.5794195
21days	ID_1	avg_Angle__left__front__elbow_wrist_toetip	134.826873
21days	ID_2	avg_Angle__left__front__elbow_wrist_toetip	124.349755
21days	ID_3	avg_Angle__left__front__elbow_wrist_toetip	148.4783237
21days	ID_4	avg_Angle__left__front__elbow_wrist_toetip	121.3859247
21days	ID_5	avg_Angle__left__front__elbow_wrist_toetip	151.7144917
21days	ID_6	avg_Angle__left__front__elbow_wrist_toetip	127.5613641
21days	ID_7	avg_Angle__left__front__elbow_wrist_toetip	126.4471065
21days	ID_8	avg_Angle__left__front__elbow_wrist_toetip	141.4164273
21days	ID_9	avg_Angle__left__front__elbow_wrist_toetip	144.2206726
21days	ID_10	avg_Angle__left__front__elbow_wrist_toetip	145.2322498
0days	ID_1	avg_Angle__left__front__shoulder_elbow_wrist	118.090206
0days	ID_2	avg_Angle__left__front__shoulder_elbow_wrist	115.5624276
0days	ID_3	avg_Angle__left__front__shoulder_elbow_wrist	137.0553127
0days	ID_4	avg_Angle__left__front__shoulder_elbow_wrist	121.8632327
0days	ID_5	avg_Angle__left__front__shoulder_elbow_wrist	115.7192397
0days	ID_6	avg_Angle__left__front__shoulder_elbow_wrist	120.8249607
0days	ID_7	avg_Angle__left__front__shoulder_elbow_wrist	118.5762272
0days	ID_8	avg_Angle__left__front__shoulder_elbow_wrist	118.7520815
0days	ID_9	avg_Angle__left__front__shoulder_elbow_wrist	116.9937495
0days	ID_10	avg_Angle__left__front__shoulder_elbow_wrist	118.5028724
0days	ID_11	avg_Angle__left__front__shoulder_elbow_wrist	129.1210117
0days	ID_12	avg_Angle__left__front__shoulder_elbow_wrist	114.4313034
0days	ID_13	avg_Angle__left__front__shoulder_elbow_wrist	121.3388142
3days	ID_1	avg_Angle__left__front__shoulder_elbow_wrist	124.0229817
3days	ID_2	avg_Angle__left__front__shoulder_elbow_wrist	126.3040495
3days	ID_3	avg_Angle__left__front__shoulder_elbow_wrist	118.4023969
3days	ID_4	avg_Angle__left__front__shoulder_elbow_wrist	124.0874927
3days	ID_5	avg_Angle__left__front__shoulder_elbow_wrist	123.6507557
3days	ID_6	avg_Angle__left__front__shoulder_elbow_wrist	127.5112357
3days	ID_7	avg_Angle__left__front__shoulder_elbow_wrist	121.8592033
3days	ID_8	avg_Angle__left__front__shoulder_elbow_wrist	121.9268735
7days	ID_1	avg_Angle__left__front__shoulder_elbow_wrist	126.7409911
7days	ID_2	avg_Angle__left__front__shoulder_elbow_wrist	108.3339656
7days	ID_3	avg_Angle__left__front__shoulder_elbow_wrist	124.1091533
7days	ID_4	avg_Angle__left__front__shoulder_elbow_wrist	122.2584768
7days	ID_5	avg_Angle__left__front__shoulder_elbow_wrist	127.9885279
7days	ID_6	avg_Angle__left__front__shoulder_elbow_wrist	117.9327268
7days	ID_7	avg_Angle__left__front__shoulder_elbow_wrist	123.8393014
7days	ID_8	avg_Angle__left__front__shoulder_elbow_wrist	116.0393596
7days	ID_9	avg_Angle__left__front__shoulder_elbow_wrist	121.0498651
14days	ID_1	avg_Angle__left__front__shoulder_elbow_wrist	136.2185214
14days	ID_2	avg_Angle__left__front__shoulder_elbow_wrist	138.3342963
14days	ID_3	avg_Angle__left__front__shoulder_elbow_wrist	124.3993537
14days	ID_4	avg_Angle__left__front__shoulder_elbow_wrist	118.1238956
14days	ID_5	avg_Angle__left__front__shoulder_elbow_wrist	119.8397393
14days	ID_6	avg_Angle__left__front__shoulder_elbow_wrist	123.1079442
14days	ID_7	avg_Angle__left__front__shoulder_elbow_wrist	114.9154838

14days	ID_8	avg_Angle__left__front__shoulder_elbow_wrist	112.221325
14days	ID_9	avg_Angle__left__front__shoulder_elbow_wrist	134.8577193
14days	ID_10	avg_Angle__left__front__shoulder_elbow_wrist	131.066123
14days	ID_11	avg_Angle__left__front__shoulder_elbow_wrist	128.6653096
21days	ID_1	avg_Angle__left__front__shoulder_elbow_wrist	118.5018086
21days	ID_2	avg_Angle__left__front__shoulder_elbow_wrist	134.9192923
21days	ID_3	avg_Angle__left__front__shoulder_elbow_wrist	119.6870298
21days	ID_4	avg_Angle__left__front__shoulder_elbow_wrist	133.1994913
21days	ID_5	avg_Angle__left__front__shoulder_elbow_wrist	124.0967548
21days	ID_6	avg_Angle__left__front__shoulder_elbow_wrist	138.6167141
21days	ID_7	avg_Angle__left__front__shoulder_elbow_wrist	129.9839995
21days	ID_8	avg_Angle__left__front__shoulder_elbow_wrist	117.7658956
21days	ID_9	avg_Angle__left__front__shoulder_elbow_wrist	110.2205771
21days	ID_10	avg_Angle__left__front__shoulder_elbow_wrist	119.8867237
0days	ID_1	avg_Angle__right__back__hip_ankle_toe	114.5769635
0days	ID_2	avg_Angle__right__back__hip_ankle_toe	122.2705118
0days	ID_3	avg_Angle__right__back__hip_ankle_toe	103.3324766
0days	ID_4	avg_Angle__right__back__hip_ankle_toe	112.8440145
0days	ID_5	avg_Angle__right__back__hip_ankle_toe	110.2712448
0days	ID_6	avg_Angle__right__back__hip_ankle_toe	121.8546027
0days	ID_7	avg_Angle__right__back__hip_ankle_toe	126.7599113
0days	ID_8	avg_Angle__right__back__hip_ankle_toe	121.4244838
0days	ID_9	avg_Angle__right__back__hip_ankle_toe	113.8967985
0days	ID_10	avg_Angle__right__back__hip_ankle_toe	110.1801702
0days	ID_11	avg_Angle__right__back__hip_ankle_toe	108.940755
0days	ID_12	avg_Angle__right__back__hip_ankle_toe	120.5667082
0days	ID_13	avg_Angle__right__back__hip_ankle_toe	119.9883246
3days	ID_1	avg_Angle__right__back__hip_ankle_toe	106.8085309
3days	ID_2	avg_Angle__right__back__hip_ankle_toe	103.6828796
3days	ID_3	avg_Angle__right__back__hip_ankle_toe	102.5763069
3days	ID_4	avg_Angle__right__back__hip_ankle_toe	107.0847789
3days	ID_5	avg_Angle__right__back__hip_ankle_toe	115.7164427
3days	ID_6	avg_Angle__right__back__hip_ankle_toe	101.30363
3days	ID_7	avg_Angle__right__back__hip_ankle_toe	115.9324685
3days	ID_8	avg_Angle__right__back__hip_ankle_toe	108.7504831
7days	ID_1	avg_Angle__right__back__hip_ankle_toe	105.1805545
7days	ID_2	avg_Angle__right__back__hip_ankle_toe	104.6534542
7days	ID_3	avg_Angle__right__back__hip_ankle_toe	106.6916234
7days	ID_4	avg_Angle__right__back__hip_ankle_toe	116.8184409
7days	ID_5	avg_Angle__right__back__hip_ankle_toe	122.1769937
7days	ID_6	avg_Angle__right__back__hip_ankle_toe	113.2392476
7days	ID_7	avg_Angle__right__back__hip_ankle_toe	122.0734282
7days	ID_8	avg_Angle__right__back__hip_ankle_toe	112.2490091
7days	ID_9	avg_Angle__right__back__hip_ankle_toe	115.0983191
14days	ID_1	avg_Angle__right__back__hip_ankle_toe	111.8480481
14days	ID_2	avg_Angle__right__back__hip_ankle_toe	110.8127988
14days	ID_3	avg_Angle__right__back__hip_ankle_toe	106.8557434
14days	ID_4	avg_Angle__right__back__hip_ankle_toe	111.8824012

14days	ID_5	avg_Angle__right__back__hip_ankle_toe	111.6102234
14days	ID_6	avg_Angle__right__back__hip_ankle_toe	106.1085687
14days	ID_7	avg_Angle__right__back__hip_ankle_toe	120.6597897
14days	ID_8	avg_Angle__right__back__hip_ankle_toe	123.1715971
14days	ID_9	avg_Angle__right__back__hip_ankle_toe	122.5984961
14days	ID_10	avg_Angle__right__back__hip_ankle_toe	115.5226258
14days	ID_11	avg_Angle__right__back__hip_ankle_toe	106.8107176
21days	ID_1	avg_Angle__right__back__hip_ankle_toe	115.293123
21days	ID_2	avg_Angle__right__back__hip_ankle_toe	124.3191046
21days	ID_3	avg_Angle__right__back__hip_ankle_toe	109.0419382
21days	ID_4	avg_Angle__right__back__hip_ankle_toe	109.2447171
21days	ID_5	avg_Angle__right__back__hip_ankle_toe	108.6220688
21days	ID_6	avg_Angle__right__back__hip_ankle_toe	113.6744101
21days	ID_7	avg_Angle__right__back__hip_ankle_toe	115.6251443
21days	ID_8	avg_Angle__right__back__hip_ankle_toe	113.520347
21days	ID_9	avg_Angle__right__back__hip_ankle_toe	117.1446924
21days	ID_10	avg_Angle__right__back__hip_ankle_toe	124.3530475
0days	ID_1	avg_Angle__right__back__iliac_hip_ankle	139.9973957
0days	ID_2	avg_Angle__right__back__iliac_hip_ankle	132.2151988
0days	ID_3	avg_Angle__right__back__iliac_hip_ankle	132.1324056
0days	ID_4	avg_Angle__right__back__iliac_hip_ankle	138.7875944
0days	ID_5	avg_Angle__right__back__iliac_hip_ankle	135.5329007
0days	ID_6	avg_Angle__right__back__iliac_hip_ankle	143.9298157
0days	ID_7	avg_Angle__right__back__iliac_hip_ankle	140.863859
0days	ID_8	avg_Angle__right__back__iliac_hip_ankle	144.9916763
0days	ID_9	avg_Angle__right__back__iliac_hip_ankle	138.9638264
0days	ID_10	avg_Angle__right__back__iliac_hip_ankle	139.7879085
0days	ID_11	avg_Angle__right__back__iliac_hip_ankle	145.3395096
0days	ID_12	avg_Angle__right__back__iliac_hip_ankle	136.4507223
0days	ID_13	avg_Angle__right__back__iliac_hip_ankle	135.6055551
3days	ID_1	avg_Angle__right__back__iliac_hip_ankle	145.0867908
3days	ID_2	avg_Angle__right__back__iliac_hip_ankle	138.6000723
3days	ID_3	avg_Angle__right__back__iliac_hip_ankle	158.7954739
3days	ID_4	avg_Angle__right__back__iliac_hip_ankle	152.3592993
3days	ID_5	avg_Angle__right__back__iliac_hip_ankle	152.4397201
3days	ID_6	avg_Angle__right__back__iliac_hip_ankle	157.2265762
3days	ID_7	avg_Angle__right__back__iliac_hip_ankle	158.5761679
3days	ID_8	avg_Angle__right__back__iliac_hip_ankle	146.042992
7days	ID_1	avg_Angle__right__back__iliac_hip_ankle	134.5954576
7days	ID_2	avg_Angle__right__back__iliac_hip_ankle	134.4924278
7days	ID_3	avg_Angle__right__back__iliac_hip_ankle	139.6648178
7days	ID_4	avg_Angle__right__back__iliac_hip_ankle	143.5968235
7days	ID_5	avg_Angle__right__back__iliac_hip_ankle	142.1148327
7days	ID_6	avg_Angle__right__back__iliac_hip_ankle	140.4937131
7days	ID_7	avg_Angle__right__back__iliac_hip_ankle	133.3226465
7days	ID_8	avg_Angle__right__back__iliac_hip_ankle	139.603305
7days	ID_9	avg_Angle__right__back__iliac_hip_ankle	130.7913714
14days	ID_1	avg_Angle__right__back__iliac_hip_ankle	136.2968398

14days	ID_2	avg_Angle__right__back__iliac_hip_ankle	136.3452176
14days	ID_3	avg_Angle__right__back__iliac_hip_ankle	145.6442664
14days	ID_4	avg_Angle__right__back__iliac_hip_ankle	136.4898882
14days	ID_5	avg_Angle__right__back__iliac_hip_ankle	135.8930869
14days	ID_6	avg_Angle__right__back__iliac_hip_ankle	134.8852585
14days	ID_7	avg_Angle__right__back__iliac_hip_ankle	146.1150925
14days	ID_8	avg_Angle__right__back__iliac_hip_ankle	136.7045303
14days	ID_9	avg_Angle__right__back__iliac_hip_ankle	135.7985651
14days	ID_10	avg_Angle__right__back__iliac_hip_ankle	126.191667
14days	ID_11	avg_Angle__right__back__iliac_hip_ankle	133.6199036
21days	ID_1	avg_Angle__right__back__iliac_hip_ankle	130.3417223
21days	ID_2	avg_Angle__right__back__iliac_hip_ankle	134.8075987
21days	ID_3	avg_Angle__right__back__iliac_hip_ankle	132.5816702
21days	ID_4	avg_Angle__right__back__iliac_hip_ankle	134.2766531
21days	ID_5	avg_Angle__right__back__iliac_hip_ankle	137.0984114
21days	ID_6	avg_Angle__right__back__iliac_hip_ankle	139.8085174
21days	ID_7	avg_Angle__right__back__iliac_hip_ankle	131.4147009
21days	ID_8	avg_Angle__right__back__iliac_hip_ankle	142.0365838
21days	ID_9	avg_Angle__right__back__iliac_hip_ankle	133.996252
21days	ID_10	avg_Angle__right__back__iliac_hip_ankle	132.5078168
0days	ID_1	avg_Angle__right__front__elbow_wrist_toetip	147.4149331
0days	ID_2	avg_Angle__right__front__elbow_wrist_toetip	144.1783268
0days	ID_3	avg_Angle__right__front__elbow_wrist_toetip	144.5530737
0days	ID_4	avg_Angle__right__front__elbow_wrist_toetip	148.8118398
0days	ID_5	avg_Angle__right__front__elbow_wrist_toetip	137.4973971
0days	ID_6	avg_Angle__right__front__elbow_wrist_toetip	143.5859217
0days	ID_7	avg_Angle__right__front__elbow_wrist_toetip	142.3324726
0days	ID_8	avg_Angle__right__front__elbow_wrist_toetip	149.4534672
0days	ID_9	avg_Angle__right__front__elbow_wrist_toetip	140.2925521
0days	ID_10	avg_Angle__right__front__elbow_wrist_toetip	139.6580109
0days	ID_11	avg_Angle__right__front__elbow_wrist_toetip	141.1675697
0days	ID_12	avg_Angle__right__front__elbow_wrist_toetip	138.9766468
0days	ID_13	avg_Angle__right__front__elbow_wrist_toetip	137.7000768
3days	ID_1	avg_Angle__right__front__elbow_wrist_toetip	131.7338568
3days	ID_2	avg_Angle__right__front__elbow_wrist_toetip	140.388301
3days	ID_3	avg_Angle__right__front__elbow_wrist_toetip	130.3957893
3days	ID_4	avg_Angle__right__front__elbow_wrist_toetip	134.4634459
3days	ID_5	avg_Angle__right__front__elbow_wrist_toetip	129.943651
3days	ID_6	avg_Angle__right__front__elbow_wrist_toetip	121.2009802
3days	ID_7	avg_Angle__right__front__elbow_wrist_toetip	128.0118273
3days	ID_8	avg_Angle__right__front__elbow_wrist_toetip	129.0815659
7days	ID_1	avg_Angle__right__front__elbow_wrist_toetip	126.7751667
7days	ID_2	avg_Angle__right__front__elbow_wrist_toetip	123.717096
7days	ID_3	avg_Angle__right__front__elbow_wrist_toetip	128.4997988
7days	ID_4	avg_Angle__right__front__elbow_wrist_toetip	136.6072312
7days	ID_5	avg_Angle__right__front__elbow_wrist_toetip	151.5066857
7days	ID_6	avg_Angle__right__front__elbow_wrist_toetip	132.9994875
7days	ID_7	avg_Angle__right__front__elbow_wrist_toetip	144.6194409

7days	ID_8	avg_Angle__right__front__elbow_wrist_toetip	122.4405135
7days	ID_9	avg_Angle__right__front__elbow_wrist_toetip	134.7909266
14days	ID_1	avg_Angle__right__front__elbow_wrist_toetip	131.1095135
14days	ID_2	avg_Angle__right__front__elbow_wrist_toetip	139.8249006
14days	ID_3	avg_Angle__right__front__elbow_wrist_toetip	118.1638702
14days	ID_4	avg_Angle__right__front__elbow_wrist_toetip	137.4691995
14days	ID_5	avg_Angle__right__front__elbow_wrist_toetip	133.8326746
14days	ID_6	avg_Angle__right__front__elbow_wrist_toetip	141.5698108
14days	ID_7	avg_Angle__right__front__elbow_wrist_toetip	143.4577418
14days	ID_8	avg_Angle__right__front__elbow_wrist_toetip	128.863032
14days	ID_9	avg_Angle__right__front__elbow_wrist_toetip	149.2347708
14days	ID_10	avg_Angle__right__front__elbow_wrist_toetip	143.3999389
14days	ID_11	avg_Angle__right__front__elbow_wrist_toetip	133.0763005
21days	ID_1	avg_Angle__right__front__elbow_wrist_toetip	123.6987701
21days	ID_2	avg_Angle__right__front__elbow_wrist_toetip	144.9612586
21days	ID_3	avg_Angle__right__front__elbow_wrist_toetip	127.0699557
21days	ID_4	avg_Angle__right__front__elbow_wrist_toetip	142.9652855
21days	ID_5	avg_Angle__right__front__elbow_wrist_toetip	125.3413558
21days	ID_6	avg_Angle__right__front__elbow_wrist_toetip	146.6411507
21days	ID_7	avg_Angle__right__front__elbow_wrist_toetip	150.4408418
21days	ID_8	avg_Angle__right__front__elbow_wrist_toetip	136.7602109
21days	ID_9	avg_Angle__right__front__elbow_wrist_toetip	126.0278079
21days	ID_10	avg_Angle__right__front__elbow_wrist_toetip	129.9179322
0days	ID_1	avg_Angle__right__front__shoulder_elbow_wrist	113.2772849
0days	ID_2	avg_Angle__right__front__shoulder_elbow_wrist	126.8813728
0days	ID_3	avg_Angle__right__front__shoulder_elbow_wrist	125.4043527
0days	ID_4	avg_Angle__right__front__shoulder_elbow_wrist	119.6655822
0days	ID_5	avg_Angle__right__front__shoulder_elbow_wrist	116.8499557
0days	ID_6	avg_Angle__right__front__shoulder_elbow_wrist	127.2919935
0days	ID_7	avg_Angle__right__front__shoulder_elbow_wrist	120.9959559
0days	ID_8	avg_Angle__right__front__shoulder_elbow_wrist	115.0245043
0days	ID_9	avg_Angle__right__front__shoulder_elbow_wrist	134.3973256
0days	ID_10	avg_Angle__right__front__shoulder_elbow_wrist	119.6953038
0days	ID_11	avg_Angle__right__front__shoulder_elbow_wrist	116.0094194
0days	ID_12	avg_Angle__right__front__shoulder_elbow_wrist	123.6923289
0days	ID_13	avg_Angle__right__front__shoulder_elbow_wrist	131.2565151
3days	ID_1	avg_Angle__right__front__shoulder_elbow_wrist	126.3122977
3days	ID_2	avg_Angle__right__front__shoulder_elbow_wrist	126.139292
3days	ID_3	avg_Angle__right__front__shoulder_elbow_wrist	122.6527397
3days	ID_4	avg_Angle__right__front__shoulder_elbow_wrist	130.1594753
3days	ID_5	avg_Angle__right__front__shoulder_elbow_wrist	134.1475915
3days	ID_6	avg_Angle__right__front__shoulder_elbow_wrist	128.6257931
3days	ID_7	avg_Angle__right__front__shoulder_elbow_wrist	128.3984498
3days	ID_8	avg_Angle__right__front__shoulder_elbow_wrist	133.7257222
7days	ID_1	avg_Angle__right__front__shoulder_elbow_wrist	118.7624668
7days	ID_2	avg_Angle__right__front__shoulder_elbow_wrist	126.7617471
7days	ID_3	avg_Angle__right__front__shoulder_elbow_wrist	133.1418351
7days	ID_4	avg_Angle__right__front__shoulder_elbow_wrist	125.7375807

7days	ID_5	avg_Angle__right__front__shoulder_elbow_wrist	126.1182715
7days	ID_6	avg_Angle__right__front__shoulder_elbow_wrist	123.7695133
7days	ID_7	avg_Angle__right__front__shoulder_elbow_wrist	114.7837253
7days	ID_8	avg_Angle__right__front__shoulder_elbow_wrist	132.59376
7days	ID_9	avg_Angle__right__front__shoulder_elbow_wrist	114.3278074
14days	ID_1	avg_Angle__right__front__shoulder_elbow_wrist	129.8586273
14days	ID_2	avg_Angle__right__front__shoulder_elbow_wrist	126.3839048
14days	ID_3	avg_Angle__right__front__shoulder_elbow_wrist	125.23928
14days	ID_4	avg_Angle__right__front__shoulder_elbow_wrist	121.9049694
14days	ID_5	avg_Angle__right__front__shoulder_elbow_wrist	123.0158817
14days	ID_6	avg_Angle__right__front__shoulder_elbow_wrist	124.9294654
14days	ID_7	avg_Angle__right__front__shoulder_elbow_wrist	130.5094206
14days	ID_8	avg_Angle__right__front__shoulder_elbow_wrist	124.4738622
14days	ID_9	avg_Angle__right__front__shoulder_elbow_wrist	116.8330872
14days	ID_10	avg_Angle__right__front__shoulder_elbow_wrist	127.9545522
14days	ID_11	avg_Angle__right__front__shoulder_elbow_wrist	139.1968385
21days	ID_1	avg_Angle__right__front__shoulder_elbow_wrist	128.3816376
21days	ID_2	avg_Angle__right__front__shoulder_elbow_wrist	112.0888589
21days	ID_3	avg_Angle__right__front__shoulder_elbow_wrist	119.7675932
21days	ID_4	avg_Angle__right__front__shoulder_elbow_wrist	121.3687758
21days	ID_5	avg_Angle__right__front__shoulder_elbow_wrist	136.0642249
21days	ID_6	avg_Angle__right__front__shoulder_elbow_wrist	122.9247985
21days	ID_7	avg_Angle__right__front__shoulder_elbow_wrist	119.0599052
21days	ID_8	avg_Angle__right__front__shoulder_elbow_wrist	128.6807356
21days	ID_9	avg_Angle__right__front__shoulder_elbow_wrist	135.3323797
21days	ID_10	avg_Angle__right__front__shoulder_elbow_wrist	137.6440652
0days	ID_1	max__left__back__hip_ankle_toe	91.72732898
0days	ID_2	max__left__back__hip_ankle_toe	103.415943
0days	ID_3	max__left__back__hip_ankle_toe	86.94896634
0days	ID_4	max__left__back__hip_ankle_toe	94.89330671
0days	ID_5	max__left__back__hip_ankle_toe	92.53341159
0days	ID_6	max__left__back__hip_ankle_toe	117.3569744
0days	ID_7	max__left__back__hip_ankle_toe	104.3963876
0days	ID_8	max__left__back__hip_ankle_toe	117.5357266
0days	ID_9	max__left__back__hip_ankle_toe	99.03623939
0days	ID_10	max__left__back__hip_ankle_toe	106.4866912
0days	ID_11	max__left__back__hip_ankle_toe	103.7768113
0days	ID_12	max__left__back__hip_ankle_toe	112.5409555
0days	ID_13	max__left__back__hip_ankle_toe	110.1309223
3days	ID_1	max__left__back__hip_ankle_toe	101.6614086
3days	ID_2	max__left__back__hip_ankle_toe	91.52570474
3days	ID_3	max__left__back__hip_ankle_toe	50.62944087
3days	ID_4	max__left__back__hip_ankle_toe	105.2300481
3days	ID_5	max__left__back__hip_ankle_toe	107.9986038
3days	ID_6	max__left__back__hip_ankle_toe	64.51797797
3days	ID_7	max__left__back__hip_ankle_toe	69.17091801
3days	ID_8	max__left__back__hip_ankle_toe	113.7393019
7days	ID_1	max__left__back__hip_ankle_toe	98.52715467

7days	ID_2	max__left__back__hip_ankle_toe	92.8590913
7days	ID_3	max__left__back__hip_ankle_toe	93.78106375
7days	ID_4	max__left__back__hip_ankle_toe	109.6313984
7days	ID_5	max__left__back__hip_ankle_toe	91.86109296
7days	ID_6	max__left__back__hip_ankle_toe	107.0616571
7days	ID_7	max__left__back__hip_ankle_toe	89.9443564
7days	ID_8	max__left__back__hip_ankle_toe	100.1166307
7days	ID_9	max__left__back__hip_ankle_toe	100.9042805
14days	ID_1	max__left__back__hip_ankle_toe	83.07903389
14days	ID_2	max__left__back__hip_ankle_toe	96.32599253
14days	ID_3	max__left__back__hip_ankle_toe	101.6801311
14days	ID_4	max__left__back__hip_ankle_toe	94.9717
14days	ID_5	max__left__back__hip_ankle_toe	92.57461962
14days	ID_6	max__left__back__hip_ankle_toe	94.93041555
14days	ID_7	max__left__back__hip_ankle_toe	103.0470832
14days	ID_8	max__left__back__hip_ankle_toe	99.14942948
14days	ID_9	max__left__back__hip_ankle_toe	99.2078674
14days	ID_10	max__left__back__hip_ankle_toe	102.8291154
14days	ID_11	max__left__back__hip_ankle_toe	101.9907584
21days	ID_1	max__left__back__hip_ankle_toe	110.083241
21days	ID_2	max__left__back__hip_ankle_toe	109.9518523
21days	ID_3	max__left__back__hip_ankle_toe	109.1246646
21days	ID_4	max__left__back__hip_ankle_toe	94.69368837
21days	ID_5	max__left__back__hip_ankle_toe	97.25821168
21days	ID_6	max__left__back__hip_ankle_toe	84.79785403
21days	ID_7	max__left__back__hip_ankle_toe	104.4087642
21days	ID_8	max__left__back__hip_ankle_toe	115.0738421
21days	ID_9	max__left__back__hip_ankle_toe	108.9990528
21days	ID_10	max__left__back__hip_ankle_toe	120.5220217
0days	ID_1	max__left__back__iliac_hip_ankle	115.3714243
0days	ID_2	max__left__back__iliac_hip_ankle	117.372634
0days	ID_3	max__left__back__iliac_hip_ankle	126.0277972
0days	ID_4	max__left__back__iliac_hip_ankle	115.9285448
0days	ID_5	max__left__back__iliac_hip_ankle	119.2465194
0days	ID_6	max__left__back__iliac_hip_ankle	117.4722069
0days	ID_7	max__left__back__iliac_hip_ankle	117.9216707
0days	ID_8	max__left__back__iliac_hip_ankle	119.4815222
0days	ID_9	max__left__back__iliac_hip_ankle	113.882047
0days	ID_10	max__left__back__iliac_hip_ankle	110.8659699
0days	ID_11	max__left__back__iliac_hip_ankle	113.6981679
0days	ID_12	max__left__back__iliac_hip_ankle	107.4834909
0days	ID_13	max__left__back__iliac_hip_ankle	111.6424051
3days	ID_1	max__left__back__iliac_hip_ankle	115.6355542
3days	ID_2	max__left__back__iliac_hip_ankle	119.2463184
3days	ID_3	max__left__back__iliac_hip_ankle	137.8699229
3days	ID_4	max__left__back__iliac_hip_ankle	112.5517247
3days	ID_5	max__left__back__iliac_hip_ankle	118.6173548
3days	ID_6	max__left__back__iliac_hip_ankle	138.6060308

3days	ID_7	max__left__back__iliac_hip_ankle	134.0105199
3days	ID_8	max__left__back__iliac_hip_ankle	114.0096058
7days	ID_1	max__left__back__iliac_hip_ankle	111.5305125
7days	ID_2	max__left__back__iliac_hip_ankle	117.4604646
7days	ID_3	max__left__back__iliac_hip_ankle	119.3846507
7days	ID_4	max__left__back__iliac_hip_ankle	119.5076853
7days	ID_5	max__left__back__iliac_hip_ankle	126.3379295
7days	ID_6	max__left__back__iliac_hip_ankle	111.7489541
7days	ID_7	max__left__back__iliac_hip_ankle	117.2496673
7days	ID_8	max__left__back__iliac_hip_ankle	118.186402
7days	ID_9	max__left__back__iliac_hip_ankle	112.3142177
14days	ID_1	max__left__back__iliac_hip_ankle	116.937847
14days	ID_2	max__left__back__iliac_hip_ankle	115.1037084
14days	ID_3	max__left__back__iliac_hip_ankle	115.4921607
14days	ID_4	max__left__back__iliac_hip_ankle	114.6037582
14days	ID_5	max__left__back__iliac_hip_ankle	114.0522261
14days	ID_6	max__left__back__iliac_hip_ankle	117.8522634
14days	ID_7	max__left__back__iliac_hip_ankle	114.5243285
14days	ID_8	max__left__back__iliac_hip_ankle	118.9617234
14days	ID_9	max__left__back__iliac_hip_ankle	126.5048296
14days	ID_10	max__left__back__iliac_hip_ankle	117.2050801
14days	ID_11	max__left__back__iliac_hip_ankle	111.5723757
21days	ID_1	max__left__back__iliac_hip_ankle	109.5141684
21days	ID_2	max__left__back__iliac_hip_ankle	112.4554472
21days	ID_3	max__left__back__iliac_hip_ankle	110.4079356
21days	ID_4	max__left__back__iliac_hip_ankle	107.3789559
21days	ID_5	max__left__back__iliac_hip_ankle	116.0243885
21days	ID_6	max__left__back__iliac_hip_ankle	115.9531074
21days	ID_7	max__left__back__iliac_hip_ankle	115.1965799
21days	ID_8	max__left__back__iliac_hip_ankle	107.166235
21days	ID_9	max__left__back__iliac_hip_ankle	106.5565077
21days	ID_10	max__left__back__iliac_hip_ankle	118.5481452
0days	ID_1	max__left__front__elbow_wrist_toetip	109.1394446
0days	ID_2	max__left__front__elbow_wrist_toetip	115.0539533
0days	ID_3	max__left__front__elbow_wrist_toetip	117.5839455
0days	ID_4	max__left__front__elbow_wrist_toetip	112.1229604
0days	ID_5	max__left__front__elbow_wrist_toetip	110.4515599
0days	ID_6	max__left__front__elbow_wrist_toetip	111.7664072
0days	ID_7	max__left__front__elbow_wrist_toetip	109.6098502
0days	ID_8	max__left__front__elbow_wrist_toetip	114.7311377
0days	ID_9	max__left__front__elbow_wrist_toetip	117.5478112
0days	ID_10	max__left__front__elbow_wrist_toetip	106.8709378
0days	ID_11	max__left__front__elbow_wrist_toetip	96.15509457
0days	ID_12	max__left__front__elbow_wrist_toetip	115.4105023
0days	ID_13	max__left__front__elbow_wrist_toetip	114.237395
3days	ID_1	max__left__front__elbow_wrist_toetip	107.7074702
3days	ID_2	max__left__front__elbow_wrist_toetip	103.5673784
3days	ID_3	max__left__front__elbow_wrist_toetip	88.77930008

3days	ID_4	max__left__front__elbow_wrist_toetip	101.5046599
3days	ID_5	max__left__front__elbow_wrist_toetip	111.288825
3days	ID_6	max__left__front__elbow_wrist_toetip	86.92063815
3days	ID_7	max__left__front__elbow_wrist_toetip	88.82415752
3days	ID_8	max__left__front__elbow_wrist_toetip	106.0736816
7days	ID_1	max__left__front__elbow_wrist_toetip	89.66225482
7days	ID_2	max__left__front__elbow_wrist_toetip	99.98182634
7days	ID_3	max__left__front__elbow_wrist_toetip	101.1681076
7days	ID_4	max__left__front__elbow_wrist_toetip	109.8275479
7days	ID_5	max__left__front__elbow_wrist_toetip	99.6299151
7days	ID_6	max__left__front__elbow_wrist_toetip	108.8489702
7days	ID_7	max__left__front__elbow_wrist_toetip	89.39504579
7days	ID_8	max__left__front__elbow_wrist_toetip	110.7440502
7days	ID_9	max__left__front__elbow_wrist_toetip	93.27650942
14days	ID_1	max__left__front__elbow_wrist_toetip	89.50101884
14days	ID_2	max__left__front__elbow_wrist_toetip	92.82770689
14days	ID_3	max__left__front__elbow_wrist_toetip	74.48369071
14days	ID_4	max__left__front__elbow_wrist_toetip	93.15068127
14days	ID_5	max__left__front__elbow_wrist_toetip	92.3054446
14days	ID_6	max__left__front__elbow_wrist_toetip	103.1200699
14days	ID_7	max__left__front__elbow_wrist_toetip	111.6858828
14days	ID_8	max__left__front__elbow_wrist_toetip	114.2046072
14days	ID_9	max__left__front__elbow_wrist_toetip	103.6076443
14days	ID_10	max__left__front__elbow_wrist_toetip	93.22818414
14days	ID_11	max__left__front__elbow_wrist_toetip	102.6981546
21days	ID_1	max__left__front__elbow_wrist_toetip	106.3413062
21days	ID_2	max__left__front__elbow_wrist_toetip	89.01349576
21days	ID_3	max__left__front__elbow_wrist_toetip	108.3413232
21days	ID_4	max__left__front__elbow_wrist_toetip	91.94315286
21days	ID_5	max__left__front__elbow_wrist_toetip	104.1403033
21days	ID_6	max__left__front__elbow_wrist_toetip	92.71269221
21days	ID_7	max__left__front__elbow_wrist_toetip	93.69806701
21days	ID_8	max__left__front__elbow_wrist_toetip	104.3914548
21days	ID_9	max__left__front__elbow_wrist_toetip	112.6317496
21days	ID_10	max__left__front__elbow_wrist_toetip	108.3729804
0days	ID_1	max__left__front__shoulder_elbow_wrist	84.30849219
0days	ID_2	max__left__front__shoulder_elbow_wrist	86.62929107
0days	ID_3	max__left__front__shoulder_elbow_wrist	114.6183676
0days	ID_4	max__left__front__shoulder_elbow_wrist	88.00997075
0days	ID_5	max__left__front__shoulder_elbow_wrist	84.53577601
0days	ID_6	max__left__front__shoulder_elbow_wrist	92.35080845
0days	ID_7	max__left__front__shoulder_elbow_wrist	95.63154558
0days	ID_8	max__left__front__shoulder_elbow_wrist	88.54447942
0days	ID_9	max__left__front__shoulder_elbow_wrist	89.92213854
0days	ID_10	max__left__front__shoulder_elbow_wrist	88.9051869
0days	ID_11	max__left__front__shoulder_elbow_wrist	89.83989865
0days	ID_12	max__left__front__shoulder_elbow_wrist	88.97322094
0days	ID_13	max__left__front__shoulder_elbow_wrist	88.75442785

3days	ID_1	max__left__front__shoulder_elbow_wrist	87.90184741
3days	ID_2	max__left__front__shoulder_elbow_wrist	93.36346025
3days	ID_3	max__left__front__shoulder_elbow_wrist	84.58947978
3days	ID_4	max__left__front__shoulder_elbow_wrist	90.75839225
3days	ID_5	max__left__front__shoulder_elbow_wrist	94.3020503
3days	ID_6	max__left__front__shoulder_elbow_wrist	90.77141301
3days	ID_7	max__left__front__shoulder_elbow_wrist	102.7489449
3days	ID_8	max__left__front__shoulder_elbow_wrist	90.32943105
7days	ID_1	max__left__front__shoulder_elbow_wrist	85.3607612
7days	ID_2	max__left__front__shoulder_elbow_wrist	81.52548133
7days	ID_3	max__left__front__shoulder_elbow_wrist	90.66974203
7days	ID_4	max__left__front__shoulder_elbow_wrist	93.92620484
7days	ID_5	max__left__front__shoulder_elbow_wrist	97.96018927
7days	ID_6	max__left__front__shoulder_elbow_wrist	85.74692944
7days	ID_7	max__left__front__shoulder_elbow_wrist	94.25048263
7days	ID_8	max__left__front__shoulder_elbow_wrist	88.3251087
7days	ID_9	max__left__front__shoulder_elbow_wrist	98.89196048
14days	ID_1	max__left__front__shoulder_elbow_wrist	101.2726377
14days	ID_2	max__left__front__shoulder_elbow_wrist	102.3203625
14days	ID_3	max__left__front__shoulder_elbow_wrist	88.79720305
14days	ID_4	max__left__front__shoulder_elbow_wrist	91.51977852
14days	ID_5	max__left__front__shoulder_elbow_wrist	95.31226494
14days	ID_6	max__left__front__shoulder_elbow_wrist	89.89538177
14days	ID_7	max__left__front__shoulder_elbow_wrist	83.54411405
14days	ID_8	max__left__front__shoulder_elbow_wrist	80.75655096
14days	ID_9	max__left__front__shoulder_elbow_wrist	103.6051296
14days	ID_10	max__left__front__shoulder_elbow_wrist	100.5696788
14days	ID_11	max__left__front__shoulder_elbow_wrist	87.70256677
21days	ID_1	max__left__front__shoulder_elbow_wrist	83.46066055
21days	ID_2	max__left__front__shoulder_elbow_wrist	96.88246865
21days	ID_3	max__left__front__shoulder_elbow_wrist	83.30474903
21days	ID_4	max__left__front__shoulder_elbow_wrist	101.5530932
21days	ID_5	max__left__front__shoulder_elbow_wrist	90.38312205
21days	ID_6	max__left__front__shoulder_elbow_wrist	105.9018232
21days	ID_7	max__left__front__shoulder_elbow_wrist	97.570341
21days	ID_8	max__left__front__shoulder_elbow_wrist	87.07982696
21days	ID_9	max__left__front__shoulder_elbow_wrist	80.401434
21days	ID_10	max__left__front__shoulder_elbow_wrist	86.63374923
0days	ID_1	max__right__back__hip_ankle_toe	98.09740996
0days	ID_2	max__right__back__hip_ankle_toe	107.1280945
0days	ID_3	max__right__back__hip_ankle_toe	91.38798124
0days	ID_4	max__right__back__hip_ankle_toe	89.79730061
0days	ID_5	max__right__back__hip_ankle_toe	92.44508567
0days	ID_6	max__right__back__hip_ankle_toe	107.5178033
0days	ID_7	max__right__back__hip_ankle_toe	109.9635415
0days	ID_8	max__right__back__hip_ankle_toe	101.4994907
0days	ID_9	max__right__back__hip_ankle_toe	96.96533204
0days	ID_10	max__right__back__hip_ankle_toe	98.60137476

0days	ID_11	max__right__back__hip_ankle_toe	99.60377412
0days	ID_12	max__right__back__hip_ankle_toe	108.8444601
0days	ID_13	max__right__back__hip_ankle_toe	108.9121
3days	ID_1	max__right__back__hip_ankle_toe	89.9122847
3days	ID_2	max__right__back__hip_ankle_toe	83.29081951
3days	ID_3	max__right__back__hip_ankle_toe	71.29628097
3days	ID_4	max__right__back__hip_ankle_toe	92.12811429
3days	ID_5	max__right__back__hip_ankle_toe	98.33487308
3days	ID_6	max__right__back__hip_ankle_toe	56.93142735
3days	ID_7	max__right__back__hip_ankle_toe	79.44998109
3days	ID_8	max__right__back__hip_ankle_toe	87.01503671
7days	ID_1	max__right__back__hip_ankle_toe	91.26276361
7days	ID_2	max__right__back__hip_ankle_toe	87.81608679
7days	ID_3	max__right__back__hip_ankle_toe	91.70726729
7days	ID_4	max__right__back__hip_ankle_toe	98.27124061
7days	ID_5	max__right__back__hip_ankle_toe	106.57002
7days	ID_6	max__right__back__hip_ankle_toe	97.42542199
7days	ID_7	max__right__back__hip_ankle_toe	104.8321287
7days	ID_8	max__right__back__hip_ankle_toe	93.23755083
7days	ID_9	max__right__back__hip_ankle_toe	100.0327779
14days	ID_1	max__right__back__hip_ankle_toe	90.50324738
14days	ID_2	max__right__back__hip_ankle_toe	90.68399971
14days	ID_3	max__right__back__hip_ankle_toe	93.96665162
14days	ID_4	max__right__back__hip_ankle_toe	99.00391277
14days	ID_5	max__right__back__hip_ankle_toe	99.47641336
14days	ID_6	max__right__back__hip_ankle_toe	84.58263895
14days	ID_7	max__right__back__hip_ankle_toe	102.3983019
14days	ID_8	max__right__back__hip_ankle_toe	109.8576089
14days	ID_9	max__right__back__hip_ankle_toe	102.7613329
14days	ID_10	max__right__back__hip_ankle_toe	103.8333401
14days	ID_11	max__right__back__hip_ankle_toe	93.45188218
21days	ID_1	max__right__back__hip_ankle_toe	101.6829115
21days	ID_2	max__right__back__hip_ankle_toe	95.87819758
21days	ID_3	max__right__back__hip_ankle_toe	96.80301542
21days	ID_4	max__right__back__hip_ankle_toe	92.78694543
21days	ID_5	max__right__back__hip_ankle_toe	95.79613646
21days	ID_6	max__right__back__hip_ankle_toe	85.8144255
21days	ID_7	max__right__back__hip_ankle_toe	102.0594265
21days	ID_8	max__right__back__hip_ankle_toe	98.60975728
21days	ID_9	max__right__back__hip_ankle_toe	103.0829294
21days	ID_10	max__right__back__hip_ankle_toe	107.7974327
0days	ID_1	max__right__back__iliac_hip_ankle	112.0990845
0days	ID_2	max__right__back__iliac_hip_ankle	108.7072396
0days	ID_3	max__right__back__iliac_hip_ankle	119.5516735
0days	ID_4	max__right__back__iliac_hip_ankle	120.3724578
0days	ID_5	max__right__back__iliac_hip_ankle	114.6983898
0days	ID_6	max__right__back__iliac_hip_ankle	119.9536656
0days	ID_7	max__right__back__iliac_hip_ankle	118.0108086

0days	ID_8	max__right__back__iliac_hip_ankle	116.2949838
0days	ID_9	max__right__back__iliac_hip_ankle	117.0934756
0days	ID_10	max__right__back__iliac_hip_ankle	115.2232001
0days	ID_11	max__right__back__iliac_hip_ankle	110.0888078
0days	ID_12	max__right__back__iliac_hip_ankle	113.8600195
0days	ID_13	max__right__back__iliac_hip_ankle	111.0195792
3days	ID_1	max__right__back__iliac_hip_ankle	115.5956252
3days	ID_2	max__right__back__iliac_hip_ankle	120.4523037
3days	ID_3	max__right__back__iliac_hip_ankle	124.113007
3days	ID_4	max__right__back__iliac_hip_ankle	127.5679809
3days	ID_5	max__right__back__iliac_hip_ankle	128.0599037
3days	ID_6	max__right__back__iliac_hip_ankle	125.0521204
3days	ID_7	max__right__back__iliac_hip_ankle	116.140444
3days	ID_8	max__right__back__iliac_hip_ankle	127.9030432
7days	ID_1	max__right__back__iliac_hip_ankle	104.8485708
7days	ID_2	max__right__back__iliac_hip_ankle	112.7933078
7days	ID_3	max__right__back__iliac_hip_ankle	113.5135571
7days	ID_4	max__right__back__iliac_hip_ankle	121.1774945
7days	ID_5	max__right__back__iliac_hip_ankle	118.1878291
7days	ID_6	max__right__back__iliac_hip_ankle	115.6278345
7days	ID_7	max__right__back__iliac_hip_ankle	106.1001075
7days	ID_8	max__right__back__iliac_hip_ankle	115.2921169
7days	ID_9	max__right__back__iliac_hip_ankle	111.2469024
14days	ID_1	max__right__back__iliac_hip_ankle	113.3525857
14days	ID_2	max__right__back__iliac_hip_ankle	119.1741782
14days	ID_3	max__right__back__iliac_hip_ankle	101.7573607
14days	ID_4	max__right__back__iliac_hip_ankle	113.7868875
14days	ID_5	max__right__back__iliac_hip_ankle	115.1471597
14days	ID_6	max__right__back__iliac_hip_ankle	115.6366594
14days	ID_7	max__right__back__iliac_hip_ankle	123.2281414
14days	ID_8	max__right__back__iliac_hip_ankle	106.4285606
14days	ID_9	max__right__back__iliac_hip_ankle	112.7082898
14days	ID_10	max__right__back__iliac_hip_ankle	110.6225826
14days	ID_11	max__right__back__iliac_hip_ankle	110.02597
21days	ID_1	max__right__back__iliac_hip_ankle	108.6244495
21days	ID_2	max__right__back__iliac_hip_ankle	107.0100279
21days	ID_3	max__right__back__iliac_hip_ankle	111.9578181
21days	ID_4	max__right__back__iliac_hip_ankle	115.4965786
21days	ID_5	max__right__back__iliac_hip_ankle	121.9876146
21days	ID_6	max__right__back__iliac_hip_ankle	118.4272161
21days	ID_7	max__right__back__iliac_hip_ankle	121.6298435
21days	ID_8	max__right__back__iliac_hip_ankle	116.6923442
21days	ID_9	max__right__back__iliac_hip_ankle	108.3975982
21days	ID_10	max__right__back__iliac_hip_ankle	107.3501603
0days	ID_1	max__right__front__elbow_wrist_toetip	101.5465915
0days	ID_2	max__right__front__elbow_wrist_toetip	107.5680155
0days	ID_3	max__right__front__elbow_wrist_toetip	116.2310477
0days	ID_4	max__right__front__elbow_wrist_toetip	114.1121752

0days	ID_5	max__right__front__elbow_wrist_toetip	96.68553235
0days	ID_6	max__right__front__elbow_wrist_toetip	109.2646057
0days	ID_7	max__right__front__elbow_wrist_toetip	107.061423
0days	ID_8	max__right__front__elbow_wrist_toetip	113.1218669
0days	ID_9	max__right__front__elbow_wrist_toetip	115.7744057
0days	ID_10	max__right__front__elbow_wrist_toetip	88.75873999
0days	ID_11	max__right__front__elbow_wrist_toetip	86.57834199
0days	ID_12	max__right__front__elbow_wrist_toetip	96.54917803
0days	ID_13	max__right__front__elbow_wrist_toetip	104.4515338
3days	ID_1	max__right__front__elbow_wrist_toetip	98.54918166
3days	ID_2	max__right__front__elbow_wrist_toetip	99.12041589
3days	ID_3	max__right__front__elbow_wrist_toetip	82.8112308
3days	ID_4	max__right__front__elbow_wrist_toetip	92.36834177
3days	ID_5	max__right__front__elbow_wrist_toetip	96.04177349
3days	ID_6	max__right__front__elbow_wrist_toetip	82.71672841
3days	ID_7	max__right__front__elbow_wrist_toetip	92.41954918
3days	ID_8	max__right__front__elbow_wrist_toetip	100.5168292
7days	ID_1	max__right__front__elbow_wrist_toetip	82.28177006
7days	ID_2	max__right__front__elbow_wrist_toetip	77.81775026
7days	ID_3	max__right__front__elbow_wrist_toetip	96.65081218
7days	ID_4	max__right__front__elbow_wrist_toetip	100.5536034
7days	ID_5	max__right__front__elbow_wrist_toetip	109.0033982
7days	ID_6	max__right__front__elbow_wrist_toetip	93.83449558
7days	ID_7	max__right__front__elbow_wrist_toetip	107.8572129
7days	ID_8	max__right__front__elbow_wrist_toetip	91.21495971
7days	ID_9	max__right__front__elbow_wrist_toetip	105.9094185
14days	ID_1	max__right__front__elbow_wrist_toetip	100.277387
14days	ID_2	max__right__front__elbow_wrist_toetip	103.7613447
14days	ID_3	max__right__front__elbow_wrist_toetip	68.21320466
14days	ID_4	max__right__front__elbow_wrist_toetip	101.5115169
14days	ID_5	max__right__front__elbow_wrist_toetip	98.65636688
14days	ID_6	max__right__front__elbow_wrist_toetip	99.92760486
14days	ID_7	max__right__front__elbow_wrist_toetip	112.1650696
14days	ID_8	max__right__front__elbow_wrist_toetip	93.97238681
14days	ID_9	max__right__front__elbow_wrist_toetip	109.3471815
14days	ID_10	max__right__front__elbow_wrist_toetip	104.1243676
14days	ID_11	max__right__front__elbow_wrist_toetip	96.04925953
21days	ID_1	max__right__front__elbow_wrist_toetip	84.35084646
21days	ID_2	max__right__front__elbow_wrist_toetip	107.2040119
21days	ID_3	max__right__front__elbow_wrist_toetip	89.27738323
21days	ID_4	max__right__front__elbow_wrist_toetip	108.8625753
21days	ID_5	max__right__front__elbow_wrist_toetip	92.95017037
21days	ID_6	max__right__front__elbow_wrist_toetip	107.0054988
21days	ID_7	max__right__front__elbow_wrist_toetip	110.2696775
21days	ID_8	max__right__front__elbow_wrist_toetip	105.3672396
21days	ID_9	max__right__front__elbow_wrist_toetip	93.63894942
21days	ID_10	max__right__front__elbow_wrist_toetip	105.2930821
0days	ID_1	max__right__front__shoulder_elbow_wrist	92.78129787

0days	ID_2	max__right__front__shoulder_elbow_wrist	97.63453396
0days	ID_3	max__right__front__shoulder_elbow_wrist	97.41524957
0days	ID_4	max__right__front__shoulder_elbow_wrist	100.5802426
0days	ID_5	max__right__front__shoulder_elbow_wrist	93.68928193
0days	ID_6	max__right__front__shoulder_elbow_wrist	104.9450912
0days	ID_7	max__right__front__shoulder_elbow_wrist	86.43819261
0days	ID_8	max__right__front__shoulder_elbow_wrist	96.18107262
0days	ID_9	max__right__front__shoulder_elbow_wrist	104.4692796
0days	ID_10	max__right__front__shoulder_elbow_wrist	98.35556161
0days	ID_11	max__right__front__shoulder_elbow_wrist	98.79397534
0days	ID_12	max__right__front__shoulder_elbow_wrist	95.66104579
0days	ID_13	max__right__front__shoulder_elbow_wrist	102.9404682
3days	ID_1	max__right__front__shoulder_elbow_wrist	99.92582509
3days	ID_2	max__right__front__shoulder_elbow_wrist	106.6574159
3days	ID_3	max__right__front__shoulder_elbow_wrist	95.97237495
3days	ID_4	max__right__front__shoulder_elbow_wrist	101.77282
3days	ID_5	max__right__front__shoulder_elbow_wrist	105.8384717
3days	ID_6	max__right__front__shoulder_elbow_wrist	99.98260491
3days	ID_7	max__right__front__shoulder_elbow_wrist	82.91539274
3days	ID_8	max__right__front__shoulder_elbow_wrist	95.89536911
7days	ID_1	max__right__front__shoulder_elbow_wrist	91.74715648
7days	ID_2	max__right__front__shoulder_elbow_wrist	99.7368484
7days	ID_3	max__right__front__shoulder_elbow_wrist	104.1167479
7days	ID_4	max__right__front__shoulder_elbow_wrist	100.4532928
7days	ID_5	max__right__front__shoulder_elbow_wrist	88.8668416
7days	ID_6	max__right__front__shoulder_elbow_wrist	99.89928787
7days	ID_7	max__right__front__shoulder_elbow_wrist	84.19134761
7days	ID_8	max__right__front__shoulder_elbow_wrist	104.7109843
7days	ID_9	max__right__front__shoulder_elbow_wrist	85.80438216
14days	ID_1	max__right__front__shoulder_elbow_wrist	98.13646818
14days	ID_2	max__right__front__shoulder_elbow_wrist	92.27651552
14days	ID_3	max__right__front__shoulder_elbow_wrist	95.8072057
14days	ID_4	max__right__front__shoulder_elbow_wrist	79.09497643
14days	ID_5	max__right__front__shoulder_elbow_wrist	77.0886452
14days	ID_6	max__right__front__shoulder_elbow_wrist	101.5909612
14days	ID_7	max__right__front__shoulder_elbow_wrist	103.7774518
14days	ID_8	max__right__front__shoulder_elbow_wrist	101.623618
14days	ID_9	max__right__front__shoulder_elbow_wrist	89.51785551
14days	ID_10	max__right__front__shoulder_elbow_wrist	93.877852
14days	ID_11	max__right__front__shoulder_elbow_wrist	109.7220009
21days	ID_1	max__right__front__shoulder_elbow_wrist	96.22340739
21days	ID_2	max__right__front__shoulder_elbow_wrist	83.83375309
21days	ID_3	max__right__front__shoulder_elbow_wrist	91.94362043
21days	ID_4	max__right__front__shoulder_elbow_wrist	83.9856519
21days	ID_5	max__right__front__shoulder_elbow_wrist	106.0741007
21days	ID_6	max__right__front__shoulder_elbow_wrist	86.65861773
21days	ID_7	max__right__front__shoulder_elbow_wrist	90.2682807
21days	ID_8	max__right__front__shoulder_elbow_wrist	101.844095

21days	ID_9	max__right__front__shoulder_elbow_wrist	97.15777285
21days	ID_10	max__right__front__shoulder_elbow_wrist	96.1437489
0days	ID_1	min__left__back__hip_ankle_toe	147.0412207
0days	ID_2	min__left__back__hip_ankle_toe	145.978783
0days	ID_3	min__left__back__hip_ankle_toe	120.1431936
0days	ID_4	min__left__back__hip_ankle_toe	139.3240292
0days	ID_5	min__left__back__hip_ankle_toe	139.2348709
0days	ID_6	min__left__back__hip_ankle_toe	149.1721694
0days	ID_7	min__left__back__hip_ankle_toe	138.6449589
0days	ID_8	min__left__back__hip_ankle_toe	150.3085489
0days	ID_9	min__left__back__hip_ankle_toe	132.692295
0days	ID_10	min__left__back__hip_ankle_toe	140.0301931
0days	ID_11	min__left__back__hip_ankle_toe	155.1288184
0days	ID_12	min__left__back__hip_ankle_toe	147.809568
0days	ID_13	min__left__back__hip_ankle_toe	151.6148064
3days	ID_1	min__left__back__hip_ankle_toe	145.7085498
3days	ID_2	min__left__back__hip_ankle_toe	132.4933488
3days	ID_3	min__left__back__hip_ankle_toe	136.9114195
3days	ID_4	min__left__back__hip_ankle_toe	143.8136867
3days	ID_5	min__left__back__hip_ankle_toe	149.3277455
3days	ID_6	min__left__back__hip_ankle_toe	148.5041428
3days	ID_7	min__left__back__hip_ankle_toe	121.7562151
3days	ID_8	min__left__back__hip_ankle_toe	151.7270164
7days	ID_1	min__left__back__hip_ankle_toe	143.9241946
7days	ID_2	min__left__back__hip_ankle_toe	151.217144
7days	ID_3	min__left__back__hip_ankle_toe	138.5545709
7days	ID_4	min__left__back__hip_ankle_toe	149.3910072
7days	ID_5	min__left__back__hip_ankle_toe	137.2258423
7days	ID_6	min__left__back__hip_ankle_toe	151.2679264
7days	ID_7	min__left__back__hip_ankle_toe	130.0495142
7days	ID_8	min__left__back__hip_ankle_toe	151.5681176
7days	ID_9	min__left__back__hip_ankle_toe	135.8193939
14days	ID_1	min__left__back__hip_ankle_toe	123.3397087
14days	ID_2	min__left__back__hip_ankle_toe	125.2653653
14days	ID_3	min__left__back__hip_ankle_toe	148.2992027
14days	ID_4	min__left__back__hip_ankle_toe	125.0830137
14days	ID_5	min__left__back__hip_ankle_toe	123.9229151
14days	ID_6	min__left__back__hip_ankle_toe	133.9349623
14days	ID_7	min__left__back__hip_ankle_toe	146.3442794
14days	ID_8	min__left__back__hip_ankle_toe	152.5210301
14days	ID_9	min__left__back__hip_ankle_toe	139.9180621
14days	ID_10	min__left__back__hip_ankle_toe	134.163788
14days	ID_11	min__left__back__hip_ankle_toe	150.7651374
21days	ID_1	min__left__back__hip_ankle_toe	147.0323842
21days	ID_2	min__left__back__hip_ankle_toe	142.5949188
21days	ID_3	min__left__back__hip_ankle_toe	139.6556164
21days	ID_4	min__left__back__hip_ankle_toe	126.163333
21days	ID_5	min__left__back__hip_ankle_toe	138.2883367

21days	ID_6	min__left__back__hip_ankle_toe	126.9477694
21days	ID_7	min__left__back__hip_ankle_toe	130.5556982
21days	ID_8	min__left__back__hip_ankle_toe	151.9466522
21days	ID_9	min__left__back__hip_ankle_toe	149.1913322
21days	ID_10	min__left__back__hip_ankle_toe	145.8561784
0days	ID_1	min__left__back__iliac_hip_ankle	164.6210857
0days	ID_2	min__left__back__iliac_hip_ankle	163.6421312
0days	ID_3	min__left__back__iliac_hip_ankle	165.8535725
0days	ID_4	min__left__back__iliac_hip_ankle	163.5440885
0days	ID_5	min__left__back__iliac_hip_ankle	168.5864692
0days	ID_6	min__left__back__iliac_hip_ankle	165.7297628
0days	ID_7	min__left__back__iliac_hip_ankle	163.6402368
0days	ID_8	min__left__back__iliac_hip_ankle	170.6709701
0days	ID_9	min__left__back__iliac_hip_ankle	155.8743763
0days	ID_10	min__left__back__iliac_hip_ankle	170.6238285
0days	ID_11	min__left__back__iliac_hip_ankle	178.8308243
0days	ID_12	min__left__back__iliac_hip_ankle	161.273217
0days	ID_13	min__left__back__iliac_hip_ankle	161.2293732
3days	ID_1	min__left__back__iliac_hip_ankle	171.7858811
3days	ID_2	min__left__back__iliac_hip_ankle	168.7570161
3days	ID_3	min__left__back__iliac_hip_ankle	184.1085158
3days	ID_4	min__left__back__iliac_hip_ankle	168.6934658
3days	ID_5	min__left__back__iliac_hip_ankle	166.8368813
3days	ID_6	min__left__back__iliac_hip_ankle	176.4547118
3days	ID_7	min__left__back__iliac_hip_ankle	176.0680471
3days	ID_8	min__left__back__iliac_hip_ankle	174.815604
7days	ID_1	min__left__back__iliac_hip_ankle	179.8671498
7days	ID_2	min__left__back__iliac_hip_ankle	177.4249699
7days	ID_3	min__left__back__iliac_hip_ankle	173.041342
7days	ID_4	min__left__back__iliac_hip_ankle	167.9842361
7days	ID_5	min__left__back__iliac_hip_ankle	167.4127881
7days	ID_6	min__left__back__iliac_hip_ankle	176.5534784
7days	ID_7	min__left__back__iliac_hip_ankle	169.0594087
7days	ID_8	min__left__back__iliac_hip_ankle	173.0691147
7days	ID_9	min__left__back__iliac_hip_ankle	169.5393214
14days	ID_1	min__left__back__iliac_hip_ankle	170.028497
14days	ID_2	min__left__back__iliac_hip_ankle	173.4257797
14days	ID_3	min__left__back__iliac_hip_ankle	185.9926747
14days	ID_4	min__left__back__iliac_hip_ankle	171.4087567
14days	ID_5	min__left__back__iliac_hip_ankle	173.3833956
14days	ID_6	min__left__back__iliac_hip_ankle	166.4887985
14days	ID_7	min__left__back__iliac_hip_ankle	164.1716742
14days	ID_8	min__left__back__iliac_hip_ankle	170.9004482
14days	ID_9	min__left__back__iliac_hip_ankle	170.788648
14days	ID_10	min__left__back__iliac_hip_ankle	175.9657905
14days	ID_11	min__left__back__iliac_hip_ankle	167.2471454
21days	ID_1	min__left__back__iliac_hip_ankle	173.9259527
21days	ID_2	min__left__back__iliac_hip_ankle	166.0741409

21days	ID_3	min__left__back__iliac_hip_ankle	171.9912105
21days	ID_4	min__left__back__iliac_hip_ankle	167.556084
21days	ID_5	min__left__back__iliac_hip_ankle	168.0364067
21days	ID_6	min__left__back__iliac_hip_ankle	163.2132321
21days	ID_7	min__left__back__iliac_hip_ankle	162.280375
21days	ID_8	min__left__back__iliac_hip_ankle	166.7748447
21days	ID_9	min__left__back__iliac_hip_ankle	167.0039304
21days	ID_10	min__left__back__iliac_hip_ankle	166.1222
0days	ID_1	min__left__front__elbow_wrist_toetip	190.6963804
0days	ID_2	min__left__front__elbow_wrist_toetip	184.949143
0days	ID_3	min__left__front__elbow_wrist_toetip	166.4973578
0days	ID_4	min__left__front__elbow_wrist_toetip	186.4425396
0days	ID_5	min__left__front__elbow_wrist_toetip	183.1381513
0days	ID_6	min__left__front__elbow_wrist_toetip	189.2530872
0days	ID_7	min__left__front__elbow_wrist_toetip	174.9726881
0days	ID_8	min__left__front__elbow_wrist_toetip	186.6502383
0days	ID_9	min__left__front__elbow_wrist_toetip	179.6208628
0days	ID_10	min__left__front__elbow_wrist_toetip	175.4246851
0days	ID_11	min__left__front__elbow_wrist_toetip	177.9955273
0days	ID_12	min__left__front__elbow_wrist_toetip	185.889003
0days	ID_13	min__left__front__elbow_wrist_toetip	199.5761567
3days	ID_1	min__left__front__elbow_wrist_toetip	182.773857
3days	ID_2	min__left__front__elbow_wrist_toetip	178.1527578
3days	ID_3	min__left__front__elbow_wrist_toetip	167.2368804
3days	ID_4	min__left__front__elbow_wrist_toetip	168.1945912
3days	ID_5	min__left__front__elbow_wrist_toetip	185.2736523
3days	ID_6	min__left__front__elbow_wrist_toetip	162.7330485
3days	ID_7	min__left__front__elbow_wrist_toetip	165.3021763
3days	ID_8	min__left__front__elbow_wrist_toetip	181.0537716
7days	ID_1	min__left__front__elbow_wrist_toetip	167.6104371
7days	ID_2	min__left__front__elbow_wrist_toetip	178.8125769
7days	ID_3	min__left__front__elbow_wrist_toetip	182.3836035
7days	ID_4	min__left__front__elbow_wrist_toetip	186.3958372
7days	ID_5	min__left__front__elbow_wrist_toetip	165.5964541
7days	ID_6	min__left__front__elbow_wrist_toetip	186.8359075
7days	ID_7	min__left__front__elbow_wrist_toetip	175.577155
7days	ID_8	min__left__front__elbow_wrist_toetip	180.1725916
7days	ID_9	min__left__front__elbow_wrist_toetip	167.2945748
14days	ID_1	min__left__front__elbow_wrist_toetip	157.5877504
14days	ID_2	min__left__front__elbow_wrist_toetip	161.0129519
14days	ID_3	min__left__front__elbow_wrist_toetip	166.1516207
14days	ID_4	min__left__front__elbow_wrist_toetip	162.1183818
14days	ID_5	min__left__front__elbow_wrist_toetip	158.9915776
14days	ID_6	min__left__front__elbow_wrist_toetip	179.6207762
14days	ID_7	min__left__front__elbow_wrist_toetip	182.464194
14days	ID_8	min__left__front__elbow_wrist_toetip	192.8429763
14days	ID_9	min__left__front__elbow_wrist_toetip	158.2858851
14days	ID_10	min__left__front__elbow_wrist_toetip	158.505806

14days	ID_11	min__left__front__elbow_wrist_toetip	169.6498049
21days	ID_1	min__left__front__elbow_wrist_toetip	173.5109729
21days	ID_2	min__left__front__elbow_wrist_toetip	155.099008
21days	ID_3	min__left__front__elbow_wrist_toetip	194.0440285
21days	ID_4	min__left__front__elbow_wrist_toetip	157.2325801
21days	ID_5	min__left__front__elbow_wrist_toetip	181.7846292
21days	ID_6	min__left__front__elbow_wrist_toetip	155.1752468
21days	ID_7	min__left__front__elbow_wrist_toetip	162.8179563
21days	ID_8	min__left__front__elbow_wrist_toetip	188.0455322
21days	ID_9	min__left__front__elbow_wrist_toetip	186.4701767
21days	ID_10	min__left__front__elbow_wrist_toetip	189.4067468
0days	ID_1	min__left__front__shoulder_elbow_wrist	159.4007057
0days	ID_2	min__left__front__shoulder_elbow_wrist	159.0398387
0days	ID_3	min__left__front__shoulder_elbow_wrist	169.0967875
0days	ID_4	min__left__front__shoulder_elbow_wrist	159.1771127
0days	ID_5	min__left__front__shoulder_elbow_wrist	160.6846703
0days	ID_6	min__left__front__shoulder_elbow_wrist	168.3004372
0days	ID_7	min__left__front__shoulder_elbow_wrist	154.3385826
0days	ID_8	min__left__front__shoulder_elbow_wrist	158.5790006
0days	ID_9	min__left__front__shoulder_elbow_wrist	157.3644562
0days	ID_10	min__left__front__shoulder_elbow_wrist	157.2528986
0days	ID_11	min__left__front__shoulder_elbow_wrist	178.1307668
0days	ID_12	min__left__front__shoulder_elbow_wrist	160.2604429
0days	ID_13	min__left__front__shoulder_elbow_wrist	161.6050236
3days	ID_1	min__left__front__shoulder_elbow_wrist	160.3175702
3days	ID_2	min__left__front__shoulder_elbow_wrist	168.6898251
3days	ID_3	min__left__front__shoulder_elbow_wrist	174.4881478
3days	ID_4	min__left__front__shoulder_elbow_wrist	171.0912211
3days	ID_5	min__left__front__shoulder_elbow_wrist	170.3889173
3days	ID_6	min__left__front__shoulder_elbow_wrist	173.6895015
3days	ID_7	min__left__front__shoulder_elbow_wrist	168.9631246
3days	ID_8	min__left__front__shoulder_elbow_wrist	163.0139214
7days	ID_1	min__left__front__shoulder_elbow_wrist	179.27291
7days	ID_2	min__left__front__shoulder_elbow_wrist	161.1427312
7days	ID_3	min__left__front__shoulder_elbow_wrist	163.5644764
7days	ID_4	min__left__front__shoulder_elbow_wrist	164.8556868
7days	ID_5	min__left__front__shoulder_elbow_wrist	174.7093452
7days	ID_6	min__left__front__shoulder_elbow_wrist	165.2443637
7days	ID_7	min__left__front__shoulder_elbow_wrist	172.8900061
7days	ID_8	min__left__front__shoulder_elbow_wrist	162.6271194
7days	ID_9	min__left__front__shoulder_elbow_wrist	175.5311295
14days	ID_1	min__left__front__shoulder_elbow_wrist	176.4368758
14days	ID_2	min__left__front__shoulder_elbow_wrist	175.7049537
14days	ID_3	min__left__front__shoulder_elbow_wrist	179.6366167
14days	ID_4	min__left__front__shoulder_elbow_wrist	169.305756
14days	ID_5	min__left__front__shoulder_elbow_wrist	173.8749867
14days	ID_6	min__left__front__shoulder_elbow_wrist	165.832295
14days	ID_7	min__left__front__shoulder_elbow_wrist	156.5270369

14days	ID_8	min__left__front__shoulder_elbow_wrist	160.4084698
14days	ID_9	min__left__front__shoulder_elbow_wrist	176.1660423
14days	ID_10	min__left__front__shoulder_elbow_wrist	171.5972762
14days	ID_11	min__left__front__shoulder_elbow_wrist	164.6457876
21days	ID_1	min__left__front__shoulder_elbow_wrist	175.6742946
21days	ID_2	min__left__front__shoulder_elbow_wrist	168.382931
21days	ID_3	min__left__front__shoulder_elbow_wrist	156.4274699
21days	ID_4	min__left__front__shoulder_elbow_wrist	173.549149
21days	ID_5	min__left__front__shoulder_elbow_wrist	170.8004995
21days	ID_6	min__left__front__shoulder_elbow_wrist	180.6563487
21days	ID_7	min__left__front__shoulder_elbow_wrist	169.3810777
21days	ID_8	min__left__front__shoulder_elbow_wrist	159.6027987
21days	ID_9	min__left__front__shoulder_elbow_wrist	162.9043
21days	ID_10	min__left__front__shoulder_elbow_wrist	161.3284819
0days	ID_1	min__right__back__hip_ankle_toe	137.566607
0days	ID_2	min__right__back__hip_ankle_toe	144.436746
0days	ID_3	min__right__back__hip_ankle_toe	118.2785234
0days	ID_4	min__right__back__hip_ankle_toe	127.8559332
0days	ID_5	min__right__back__hip_ankle_toe	124.2762428
0days	ID_6	min__right__back__hip_ankle_toe	145.8065366
0days	ID_7	min__right__back__hip_ankle_toe	145.7821659
0days	ID_8	min__right__back__hip_ankle_toe	152.1876887
0days	ID_9	min__right__back__hip_ankle_toe	139.9501295
0days	ID_10	min__right__back__hip_ankle_toe	127.748874
0days	ID_11	min__right__back__hip_ankle_toe	126.9095843
0days	ID_12	min__right__back__hip_ankle_toe	138.013383
0days	ID_13	min__right__back__hip_ankle_toe	155.2297325
3days	ID_1	min__right__back__hip_ankle_toe	132.0886164
3days	ID_2	min__right__back__hip_ankle_toe	125.5735534
3days	ID_3	min__right__back__hip_ankle_toe	116.0563943
3days	ID_4	min__right__back__hip_ankle_toe	127.6385145
3days	ID_5	min__right__back__hip_ankle_toe	136.7274602
3days	ID_6	min__right__back__hip_ankle_toe	120.4866862
3days	ID_7	min__right__back__hip_ankle_toe	144.2561887
3days	ID_8	min__right__back__hip_ankle_toe	132.637952
7days	ID_1	min__right__back__hip_ankle_toe	126.2641575
7days	ID_2	min__right__back__hip_ankle_toe	122.3565674
7days	ID_3	min__right__back__hip_ankle_toe	119.9246068
7days	ID_4	min__right__back__hip_ankle_toe	137.2615942
7days	ID_5	min__right__back__hip_ankle_toe	146.8664482
7days	ID_6	min__right__back__hip_ankle_toe	134.900534
7days	ID_7	min__right__back__hip_ankle_toe	155.1670414
7days	ID_8	min__right__back__hip_ankle_toe	134.4494994
7days	ID_9	min__right__back__hip_ankle_toe	152.1363313
14days	ID_1	min__right__back__hip_ankle_toe	139.1109345
14days	ID_2	min__right__back__hip_ankle_toe	151.4425914
14days	ID_3	min__right__back__hip_ankle_toe	126.8615427
14days	ID_4	min__right__back__hip_ankle_toe	147.9064403

14days	ID_5	min__right__back__hip_ankle_toe	148.7383313
14days	ID_6	min__right__back__hip_ankle_toe	124.8138493
14days	ID_7	min__right__back__hip_ankle_toe	140.035204
14days	ID_8	min__right__back__hip_ankle_toe	269.3681928
14days	ID_9	min__right__back__hip_ankle_toe	145.3913629
14days	ID_10	min__right__back__hip_ankle_toe	143.9447768
14days	ID_11	min__right__back__hip_ankle_toe	128.1831943
21days	ID_1	min__right__back__hip_ankle_toe	141.3016094
21days	ID_2	min__right__back__hip_ankle_toe	150.3090076
21days	ID_3	min__right__back__hip_ankle_toe	126.6448869
21days	ID_4	min__right__back__hip_ankle_toe	147.2348186
21days	ID_5	min__right__back__hip_ankle_toe	129.7379315
21days	ID_6	min__right__back__hip_ankle_toe	155.0685307
21days	ID_7	min__right__back__hip_ankle_toe	145.6272112
21days	ID_8	min__right__back__hip_ankle_toe	140.070641
21days	ID_9	min__right__back__hip_ankle_toe	139.5810897
21days	ID_10	min__right__back__hip_ankle_toe	141.8285164
0days	ID_1	min__right__back__iliac_hip_ankle	165.2021713
0days	ID_2	min__right__back__iliac_hip_ankle	157.1508637
0days	ID_3	min__right__back__iliac_hip_ankle	149.9793554
0days	ID_4	min__right__back__iliac_hip_ankle	164.5641548
0days	ID_5	min__right__back__iliac_hip_ankle	164.3973745
0days	ID_6	min__right__back__iliac_hip_ankle	163.0924444
0days	ID_7	min__right__back__iliac_hip_ankle	162.1550654
0days	ID_8	min__right__back__iliac_hip_ankle	160.7031448
0days	ID_9	min__right__back__iliac_hip_ankle	171.5267003
0days	ID_10	min__right__back__iliac_hip_ankle	175.4941822
0days	ID_11	min__right__back__iliac_hip_ankle	172.1369545
0days	ID_12	min__right__back__iliac_hip_ankle	162.7766401
0days	ID_13	min__right__back__iliac_hip_ankle	166.4335421
3days	ID_1	min__right__back__iliac_hip_ankle	184.5324493
3days	ID_2	min__right__back__iliac_hip_ankle	170.2128867
3days	ID_3	min__right__back__iliac_hip_ankle	177.9152088
3days	ID_4	min__right__back__iliac_hip_ankle	175.8283244
3days	ID_5	min__right__back__iliac_hip_ankle	166.5011262
3days	ID_6	min__right__back__iliac_hip_ankle	176.135997
3days	ID_7	min__right__back__iliac_hip_ankle	176.8437227
3days	ID_8	min__right__back__iliac_hip_ankle	166.1998313
7days	ID_1	min__right__back__iliac_hip_ankle	179.0141384
7days	ID_2	min__right__back__iliac_hip_ankle	174.9241482
7days	ID_3	min__right__back__iliac_hip_ankle	186.82825
7days	ID_4	min__right__back__iliac_hip_ankle	165.2977495
7days	ID_5	min__right__back__iliac_hip_ankle	170.3762344
7days	ID_6	min__right__back__iliac_hip_ankle	161.1185885
7days	ID_7	min__right__back__iliac_hip_ankle	161.6724177
7days	ID_8	min__right__back__iliac_hip_ankle	163.4165674
7days	ID_9	min__right__back__iliac_hip_ankle	170.4908928
14days	ID_1	min__right__back__iliac_hip_ankle	164.4972315

14days	ID_2	min__right__back__iliac_hip_ankle	171.1924813
14days	ID_3	min__right__back__iliac_hip_ankle	181.4079536
14days	ID_4	min__right__back__iliac_hip_ankle	175.177989
14days	ID_5	min__right__back__iliac_hip_ankle	175.5167888
14days	ID_6	min__right__back__iliac_hip_ankle	165.4213545
14days	ID_7	min__right__back__iliac_hip_ankle	165.6178041
14days	ID_8	min__right__back__iliac_hip_ankle	161.8336829
14days	ID_9	min__right__back__iliac_hip_ankle	157.739733
14days	ID_10	min__right__back__iliac_hip_ankle	168.4328513
14days	ID_11	min__right__back__iliac_hip_ankle	162.4237469
21days	ID_1	min__right__back__iliac_hip_ankle	164.9995803
21days	ID_2	min__right__back__iliac_hip_ankle	166.820456
21days	ID_3	min__right__back__iliac_hip_ankle	166.6415109
21days	ID_4	min__right__back__iliac_hip_ankle	169.8237028
21days	ID_5	min__right__back__iliac_hip_ankle	164.0759398
21days	ID_6	min__right__back__iliac_hip_ankle	170.1621808
21days	ID_7	min__right__back__iliac_hip_ankle	167.8193744
21days	ID_8	min__right__back__iliac_hip_ankle	163.2236519
21days	ID_9	min__right__back__iliac_hip_ankle	165.9912849
21days	ID_10	min__right__back__iliac_hip_ankle	160.0504547
0days	ID_1	min__right__front__elbow_wrist_toetip	170.0339887
0days	ID_2	min__right__front__elbow_wrist_toetip	166.890535
0days	ID_3	min__right__front__elbow_wrist_toetip	186.9580726
0days	ID_4	min__right__front__elbow_wrist_toetip	170.0988295
0days	ID_5	min__right__front__elbow_wrist_toetip	189.7303798
0days	ID_6	min__right__front__elbow_wrist_toetip	167.2936008
0days	ID_7	min__right__front__elbow_wrist_toetip	184.0455259
0days	ID_8	min__right__front__elbow_wrist_toetip	179.692531
0days	ID_9	min__right__front__elbow_wrist_toetip	167.26677
0days	ID_10	min__right__front__elbow_wrist_toetip	174.2524409
0days	ID_11	min__right__front__elbow_wrist_toetip	169.205196
0days	ID_12	min__right__front__elbow_wrist_toetip	168.0820087
0days	ID_13	min__right__front__elbow_wrist_toetip	162.0875121
3days	ID_1	min__right__front__elbow_wrist_toetip	167.2473128
3days	ID_2	min__right__front__elbow_wrist_toetip	166.1233948
3days	ID_3	min__right__front__elbow_wrist_toetip	165.790433
3days	ID_4	min__right__front__elbow_wrist_toetip	167.2423151
3days	ID_5	min__right__front__elbow_wrist_toetip	163.0803778
3days	ID_6	min__right__front__elbow_wrist_toetip	166.4967531
3days	ID_7	min__right__front__elbow_wrist_toetip	166.8737621
3days	ID_8	min__right__front__elbow_wrist_toetip	168.8544368
7days	ID_1	min__right__front__elbow_wrist_toetip	164.5990524
7days	ID_2	min__right__front__elbow_wrist_toetip	160.0227807
7days	ID_3	min__right__front__elbow_wrist_toetip	161.5073815
7days	ID_4	min__right__front__elbow_wrist_toetip	169.8251834
7days	ID_5	min__right__front__elbow_wrist_toetip	185.6517438
7days	ID_6	min__right__front__elbow_wrist_toetip	174.5290069
7days	ID_7	min__right__front__elbow_wrist_toetip	191.3853582

7days	ID_8	min__right__front__elbow_wrist_toetip	170.9888852
7days	ID_9	min__right__front__elbow_wrist_toetip	183.5572218
14days	ID_1	min__right__front__elbow_wrist_toetip	171.1387231
14days	ID_2	min__right__front__elbow_wrist_toetip	175.0013657
14days	ID_3	min__right__front__elbow_wrist_toetip	158.9738122
14days	ID_4	min__right__front__elbow_wrist_toetip	172.0452112
14days	ID_5	min__right__front__elbow_wrist_toetip	172.3047269
14days	ID_6	min__right__front__elbow_wrist_toetip	170.7034391
14days	ID_7	min__right__front__elbow_wrist_toetip	165.0177694
14days	ID_8	min__right__front__elbow_wrist_toetip	159.43749
14days	ID_9	min__right__front__elbow_wrist_toetip	188.2935208
14days	ID_10	min__right__front__elbow_wrist_toetip	169.8941946
14days	ID_11	min__right__front__elbow_wrist_toetip	160.6890114
21days	ID_1	min__right__front__elbow_wrist_toetip	167.8940692
21days	ID_2	min__right__front__elbow_wrist_toetip	189.3744234
21days	ID_3	min__right__front__elbow_wrist_toetip	168.1499567
21days	ID_4	min__right__front__elbow_wrist_toetip	176.5429955
21days	ID_5	min__right__front__elbow_wrist_toetip	158.7068834
21days	ID_6	min__right__front__elbow_wrist_toetip	180.7328527
21days	ID_7	min__right__front__elbow_wrist_toetip	182.5258043
21days	ID_8	min__right__front__elbow_wrist_toetip	164.3850086
21days	ID_9	min__right__front__elbow_wrist_toetip	177.4029136
21days	ID_10	min__right__front__elbow_wrist_toetip	167.3078975
0days	ID_1	min__right__front__shoulder_elbow_wrist	167.5594076
0days	ID_2	min__right__front__shoulder_elbow_wrist	165.4411877
0days	ID_3	min__right__front__shoulder_elbow_wrist	151.7757242
0days	ID_4	min__right__front__shoulder_elbow_wrist	161.2687298
0days	ID_5	min__right__front__shoulder_elbow_wrist	172.4710485
0days	ID_6	min__right__front__shoulder_elbow_wrist	172.0836837
0days	ID_7	min__right__front__shoulder_elbow_wrist	159.4804423
0days	ID_8	min__right__front__shoulder_elbow_wrist	158.3577267
0days	ID_9	min__right__front__shoulder_elbow_wrist	180.7920302
0days	ID_10	min__right__front__shoulder_elbow_wrist	176.5775949
0days	ID_11	min__right__front__shoulder_elbow_wrist	166.2460127
0days	ID_12	min__right__front__shoulder_elbow_wrist	173.117989
0days	ID_13	min__right__front__shoulder_elbow_wrist	177.5191236
3days	ID_1	min__right__front__shoulder_elbow_wrist	171.9144366
3days	ID_2	min__right__front__shoulder_elbow_wrist	162.5037427
3days	ID_3	min__right__front__shoulder_elbow_wrist	171.4350699
3days	ID_4	min__right__front__shoulder_elbow_wrist	174.6256185
3days	ID_5	min__right__front__shoulder_elbow_wrist	175.6781465
3days	ID_6	min__right__front__shoulder_elbow_wrist	179.0694642
3days	ID_7	min__right__front__shoulder_elbow_wrist	162.7436089
3days	ID_8	min__right__front__shoulder_elbow_wrist	172.4220438
7days	ID_1	min__right__front__shoulder_elbow_wrist	163.5977541
7days	ID_2	min__right__front__shoulder_elbow_wrist	183.8641945
7days	ID_3	min__right__front__shoulder_elbow_wrist	165.8388769
7days	ID_4	min__right__front__shoulder_elbow_wrist	163.5583693

7days	ID_5	min__right__front__shoulder_elbow_wrist	163.9256761
7days	ID_6	min__right__front__shoulder_elbow_wrist	175.0210027
7days	ID_7	min__right__front__shoulder_elbow_wrist	157.5719481
7days	ID_8	min__right__front__shoulder_elbow_wrist	180.2347552
7days	ID_9	min__right__front__shoulder_elbow_wrist	167.8046076
14days	ID_1	min__right__front__shoulder_elbow_wrist	170.4457194
14days	ID_2	min__right__front__shoulder_elbow_wrist	175.2760213
14days	ID_3	min__right__front__shoulder_elbow_wrist	187.446569
14days	ID_4	min__right__front__shoulder_elbow_wrist	165.9493805
14days	ID_5	min__right__front__shoulder_elbow_wrist	163.8274377
14days	ID_6	min__right__front__shoulder_elbow_wrist	163.0330161
14days	ID_7	min__right__front__shoulder_elbow_wrist	173.0932763
14days	ID_8	min__right__front__shoulder_elbow_wrist	177.0766709
14days	ID_9	min__right__front__shoulder_elbow_wrist	154.6229821
14days	ID_10	min__right__front__shoulder_elbow_wrist	174.7191183
14days	ID_11	min__right__front__shoulder_elbow_wrist	180.0441825
21days	ID_1	min__right__front__shoulder_elbow_wrist	181.0484775
21days	ID_2	min__right__front__shoulder_elbow_wrist	160.619939
21days	ID_3	min__right__front__shoulder_elbow_wrist	170.9794967
21days	ID_4	min__right__front__shoulder_elbow_wrist	167.0312112
21days	ID_5	min__right__front__shoulder_elbow_wrist	177.8685191
21days	ID_6	min__right__front__shoulder_elbow_wrist	166.3579638
21days	ID_7	min__right__front__shoulder_elbow_wrist	162.4931435
21days	ID_8	min__right__front__shoulder_elbow_wrist	168.1769528
21days	ID_9	min__right__front__shoulder_elbow_wrist	174.1670074
21days	ID_10	min__right__front__shoulder_elbow_wrist	169.8967262

Suppl. Table 26: Summary of side perspective horizontal angles analysis

Day	Measure	Mean	SD
0days	avg_Angle__left__back__hip_ankle_toe	119.111343	8.65844201
0days	avg_Angle__left__back__iliac_hip_ankle	138.043236	7.57301528
0days	avg_Angle__left__front__elbow_wrist_toetip	145.592204	7.90188404
0days	avg_Angle__left__front__shoulder_elbow_wrist	120.525495	6.21804693
0days	avg_Angle__right__back__hip_ankle_toe	115.91592	6.76303026
0days	avg_Angle__right__back__iliac_hip_ankle	138.815259	4.3619968
0days	avg_Angle__right__front__elbow_wrist_toetip	142.740176	4.03154536
0days	avg_Angle__right__front__shoulder_elbow_wrist	122.341684	6.47474687
0days	max__left__back__hip_ankle_toe	103.136897	9.78782328
0days	max__left__back__iliac_hip_ankle	115.876492	4.64422425
0days	max__left__front__elbow_wrist_toetip	111.590846	5.6784448
0days	max__left__front__shoulder_elbow_wrist	90.8479695	7.73242322
0days	max__right__back__hip_ankle_toe	100.827981	7.11727027
0days	max__right__back__iliac_hip_ankle	115.151799	3.8553309
0days	max__right__front__elbow_wrist_toetip	104.438727	9.88292302
0days	max__right__front__shoulder_elbow_wrist	97.6834841	5.07734702
0days	min__left__back__hip_ankle_toe	142.85565	9.3320989
0days	min__left__back__iliac_hip_ankle	165.701534	5.62838471
0days	min__left__front__elbow_wrist_toetip	183.161986	8.3702189
0days	min__left__front__shoulder_elbow_wrist	161.786979	6.40634549
0days	min__right__back__hip_ankle_toe	137.234011	11.4226258
0days	min__right__back__iliac_hip_ankle	164.277892	6.59481822
0days	min__right__front__elbow_wrist_toetip	173.510569	8.74332525
0days	min__right__front__shoulder_elbow_wrist	167.899285	8.53651527
3days	avg_Angle__left__back__hip_ankle_toe	115.817149	8.18087786
3days	avg_Angle__left__back__iliac_hip_ankle	148.784818	13.6262678
3days	avg_Angle__left__front__elbow_wrist_toetip	137.146706	8.55788941
3days	avg_Angle__left__front__shoulder_elbow_wrist	123.470624	2.82184551
3days	avg_Angle__right__back__hip_ankle_toe	107.73194	5.57556526
3days	avg_Angle__right__back__iliac_hip_ankle	151.140887	7.30944456
3days	avg_Angle__right__front__elbow_wrist_toetip	130.652427	5.47486517
3days	avg_Angle__right__front__shoulder_elbow_wrist	128.77017	3.89044541
3days	max__left__back__hip_ankle_toe	88.0591755	23.4851485
3days	max__left__back__iliac_hip_ankle	123.818379	11.0716034
3days	max__left__front__elbow_wrist_toetip	99.3332639	9.68828038
3days	max__left__front__shoulder_elbow_wrist	91.8456274	5.34602804
3days	max__right__back__hip_ankle_toe	82.2948522	13.1346753
3days	max__right__back__iliac_hip_ankle	123.110554	5.13016493
3days	max__right__front__elbow_wrist_toetip	93.0680063	7.01288227
3days	max__right__front__shoulder_elbow_wrist	98.6200343	7.47640818
3days	min__left__back__hip_ankle_toe	141.280266	10.2091984
3days	min__left__back__iliac_hip_ankle	173.440015	5.63790546
3days	min__left__front__elbow_wrist_toetip	173.840092	8.88748092
3days	min__left__front__shoulder_elbow_wrist	168.830279	4.92077719
3days	min__right__back__hip_ankle_toe	129.433171	9.00274326
3days	min__right__back__iliac_hip_ankle	174.271193	6.2459468

3days	min__right__front__elbow_wrist_toetip	166.463598	1.65275189
3days	min__right__front__shoulder_elbow_wrist	171.299016	5.88986056
7days	avg_Angle__left__back__hip_ankle_toe	112.959715	6.33633335
7days	avg_Angle__left__back__iliac_hip_ankle	144.638469	3.88348317
7days	avg_Angle__left__front__elbow_wrist_toetip	142.278232	7.45072884
7days	avg_Angle__left__front__shoulder_elbow_wrist	120.921374	6.07764475
7days	avg_Angle__right__back__hip_ankle_toe	113.13123	6.67643384
7days	avg_Angle__right__back__iliac_hip_ankle	137.630599	4.42047671
7days	avg_Angle__right__front__elbow_wrist_toetip	133.550705	9.66743436
7days	avg_Angle__right__front__shoulder_elbow_wrist	123.999634	6.87872875
7days	max__left__back__hip_ankle_toe	98.2985251	6.8611388
7days	max__left__back__iliac_hip_ankle	117.080054	4.74659798
7days	max__left__front__elbow_wrist_toetip	100.281581	8.33402003
7days	max__left__front__shoulder_elbow_wrist	90.7396511	5.97570012
7days	max__right__back__hip_ankle_toe	96.7950286	6.35387056
7days	max__right__back__iliac_hip_ankle	113.198636	5.27995414
7days	max__right__front__elbow_wrist_toetip	96.1248245	11.0412133
7days	max__right__front__shoulder_elbow_wrist	95.5029877	7.91283351
7days	min__left__back__hip_ankle_toe	143.22419	8.08484991
7days	min__left__back__iliac_hip_ankle	172.661312	4.50303778
7days	min__left__front__elbow_wrist_toetip	176.742126	8.22486184
7days	min__left__front__shoulder_elbow_wrist	168.870863	6.69834259
7days	min__right__back__hip_ankle_toe	136.591864	12.6848151
7days	min__right__back__iliac_hip_ankle	170.348776	8.6722293
7days	min__right__front__elbow_wrist_toetip	173.562957	11.147965
7days	min__right__front__shoulder_elbow_wrist	169.046354	8.73186362
14days	avg_Angle__left__back__hip_ankle_toe	114.360477	8.05024414
14days	avg_Angle__left__back__iliac_hip_ankle	143.247298	11.2720928
14days	avg_Angle__left__front__elbow_wrist_toetip	134.898125	8.5465791
14days	avg_Angle__left__front__shoulder_elbow_wrist	125.61361	8.88687637
14days	avg_Angle__right__back__hip_ankle_toe	113.443728	6.25543739
14days	avg_Angle__right__back__iliac_hip_ankle	136.725847	5.41883874
14days	avg_Angle__right__front__elbow_wrist_toetip	136.363796	8.59137686
14days	avg_Angle__right__front__shoulder_elbow_wrist	126.390899	5.70462638
14days	max__left__back__hip_ankle_toe	97.2532861	5.90232909
14days	max__left__back__iliac_hip_ankle	116.619118	3.86336044
14days	max__left__front__elbow_wrist_toetip	97.3466441	11.2055642
14days	max__left__front__shoulder_elbow_wrist	93.2086971	7.91715736
14days	max__right__back__hip_ankle_toe	97.3199391	7.35833156
14days	max__right__back__iliac_hip_ankle	112.897125	5.82312227
14days	max__right__front__elbow_wrist_toetip	98.9096082	11.4889138
14days	max__right__front__shoulder_elbow_wrist	94.7739591	10.017674
14days	min__left__back__hip_ankle_toe	136.687042	11.4349251
14days	min__left__back__iliac_hip_ankle	171.800146	5.7981734
14days	min__left__front__elbow_wrist_toetip	167.930157	11.9373203
14days	min__left__front__shoulder_elbow_wrist	170.012372	7.36523572
14days	min__right__back__hip_ankle_toe	151.436038	40.1940476
14days	min__right__back__iliac_hip_ankle	168.114692	7.0499522

14days	min__right__front__elbow_wrist_toetip	169.409024	8.43672161
14days	min__right__front__shoulder_elbow_wrist	171.412216	9.12569363
21days	avg_Angle__left__back__hip_ankle_toe	119.207483	8.35426229
21days	avg_Angle__left__back__iliac_hip_ankle	136.763799	3.99494863
21days	avg_Angle__left__front__elbow_wrist_toetip	136.563319	11.0291336
21days	avg_Angle__left__front__shoulder_elbow_wrist	124.687829	9.08652152
21days	avg_Angle__right__back__hip_ankle_toe	115.083859	5.70163392
21days	avg_Angle__right__back__iliac_hip_ankle	134.886993	3.72815045
21days	avg_Angle__right__front__elbow_wrist_toetip	135.382457	10.1569051
21days	avg_Angle__right__front__shoulder_elbow_wrist	126.131297	8.48583852
21days	max__left__back__hip_ankle_toe	105.491319	10.5303718
21days	max__left__back__iliac_hip_ankle	111.920147	4.32213586
21days	max__left__front__elbow_wrist_toetip	101.158653	8.43863629
21days	max__left__front__shoulder_elbow_wrist	91.3171268	8.64998268
21days	max__right__back__hip_ankle_toe	98.0311178	6.13448138
21days	max__right__back__iliac_hip_ankle	113.757365	5.85045605
21days	max__right__front__elbow_wrist_toetip	100.421943	9.36935615
21days	max__right__front__shoulder_elbow_wrist	93.4133049	7.44664291
21days	min__left__back__hip_ankle_toe	139.823222	9.25821405
21days	min__left__back__iliac_hip_ankle	167.297838	3.52247117
21days	min__left__front__elbow_wrist_toetip	174.358688	15.5251392
21days	min__left__front__shoulder_elbow_wrist	167.870735	7.70650868
21days	min__right__back__hip_ankle_toe	141.740424	8.66628733
21days	min__right__back__iliac_hip_ankle	165.960814	3.05745037
21days	min__right__front__elbow_wrist_toetip	173.302281	9.4879407
21days	min__right__front__shoulder_elbow_wrist	169.863944	6.42798948

Suppl. Table 27: Statistical test of bside perspective horizontal angle analysis

Measure	group1	group2	p.adj	sig	Test*
avg_Angle__left__back__hip_ankle_toe	0days	3days	1	ns	Rep ANOVA with PH test
avg_Angle__left__back__hip_ankle_toe	0days	7days	1	ns	Rep ANOVA with PH test
avg_Angle__left__back__hip_ankle_toe	0days	14days	1	ns	Rep ANOVA with PH test
avg_Angle__left__back__hip_ankle_toe	0days	21days	1	ns	Rep ANOVA with PH test
avg_Angle__left__back__iliac_hip_ankle	0days	3days	0.78	ns	Rep ANOVA with PH test
avg_Angle__left__back__iliac_hip_ankle	0days	7days	1	ns	Rep ANOVA with PH test
avg_Angle__left__back__iliac_hip_ankle	0days	14days	1	ns	Rep ANOVA with PH test
avg_Angle__left__back__iliac_hip_ankle	0days	21days	1	ns	Rep ANOVA with PH test
avg_Angle__left__front__ellbow_wrist_toetip	0days	3days	0.734	ns	Rep ANOVA with PH test
avg_Angle__left__front__ellbow_wrist_toetip	0days	7days	0.734	ns	Rep ANOVA with PH test
avg_Angle__left__front__ellbow_wrist_toetip	0days	14days	0.628	ns	Rep ANOVA with PH test
avg_Angle__left__front__ellbow_wrist_toetip	0days	21days	0.734	ns	Rep ANOVA with PH test
avg_Angle__left__front__shoulder_ellbow_wris	0days	3days	1	ns	Rep ANOVA with PH test
avg_Angle__left__front__shoulder_ellbow_wris	0days	7days	1	ns	Rep ANOVA with PH test
avg_Angle__left__front__shoulder_ellbow_wris	0days	14days	1	ns	Rep ANOVA with PH test
avg_Angle__left__front__shoulder_ellbow_wris	0days	21days	1	ns	Rep ANOVA with PH test
avg_Angle__right__back__hip_ankle_toe	0days	3days	1	ns	Rep ANOVA with PH test
avg_Angle__right__back__hip_ankle_toe	0days	7days	1	ns	Rep ANOVA with PH test
avg_Angle__right__back__hip_ankle_toe	0days	14days	1	ns	Rep ANOVA with PH test
avg_Angle__right__back__hip_ankle_toe	0days	21days	1	ns	Rep ANOVA with PH test
avg_Angle__right__back__iliac_hip_ankle	0days	3days	0.548	ns	Rep ANOVA with PH test
avg_Angle__right__back__iliac_hip_ankle	0days	7days	1	ns	Rep ANOVA with PH test
avg_Angle__right__back__iliac_hip_ankle	0days	14days	1	ns	Rep ANOVA with PH test
avg_Angle__right__back__iliac_hip_ankle	0days	21days	1	ns	Rep ANOVA with PH test
avg_Angle__right__front__ellbow_wrist_toetip	0days	3days	0.579	ns	Rep ANOVA with PH test
avg_Angle__right__front__ellbow_wrist_toetip	0days	7days	0.752	ns	Rep ANOVA with PH test
avg_Angle__right__front__ellbow_wrist_toetip	0days	14days	0.752	ns	Rep ANOVA with PH test
avg_Angle__right__front__ellbow_wrist_toetip	0days	21days	0.752	ns	Rep ANOVA with PH test
avg_Angle__right__front__shoulder_ellbow_wr	0days	3days	1	ns	Rep ANOVA with PH test
avg_Angle__right__front__shoulder_ellbow_wr	0days	7days	1	ns	Rep ANOVA with PH test
avg_Angle__right__front__shoulder_ellbow_wr	0days	14days	1	ns	Rep ANOVA with PH test
avg_Angle__right__front__shoulder_ellbow_wr	0days	21days	1	ns	Rep ANOVA with PH test
max__left__back__hip_ankle_toe	0days	3days	0.276	ns	Rep ANOVA with PH test
max__left__back__hip_ankle_toe	0days	7days	1	ns	Rep ANOVA with PH test
max__left__back__hip_ankle_toe	0days	14days	1	ns	Rep ANOVA with PH test
max__left__back__hip_ankle_toe	0days	21days	1	ns	Rep ANOVA with PH test
max__left__back__iliac_hip_ankle	0days	3days	1	ns	Rep ANOVA with PH test
max__left__back__iliac_hip_ankle	0days	7days	1	ns	Rep ANOVA with PH test
max__left__back__iliac_hip_ankle	0days	14days	1	ns	Rep ANOVA with PH test
max__left__back__iliac_hip_ankle	0days	21days	1	ns	Rep ANOVA with PH test
max__left__front__ellbow_wrist_toetip	0days	3days	0.418	ns	Rep ANOVA with PH test
max__left__front__ellbow_wrist_toetip	0days	7days	0.418	ns	Rep ANOVA with PH test
max__left__front__ellbow_wrist_toetip	0days	14days	0.238	ns	Rep ANOVA with PH test
max__left__front__ellbow_wrist_toetip	0days	21days	0.418	ns	Rep ANOVA with PH test
max__left__front__shoulder_ellbow_wrist	0days	3days	1	ns	Rep ANOVA with PH test
max__left__front__shoulder_ellbow_wrist	0days	7days	1	ns	Rep ANOVA with PH test

max__left__front__shoulder_ellbow_wrist	0days	14days	1	ns	Rep ANOVA with PH test
max__left__front__shoulder_ellbow_wrist	0days	21days	1	ns	Rep ANOVA with PH test
max__right__back__hip_ankle_toe	0days	3days	0.102	ns	Rep ANOVA with PH test
max__right__back__hip_ankle_toe	0days	7days	1	ns	Rep ANOVA with PH test
max__right__back__hip_ankle_toe	0days	14days	1	ns	Rep ANOVA with PH test
max__right__back__hip_ankle_toe	0days	21days	1	ns	Rep ANOVA with PH test
max__right__back__iliac_hip_ankle	0days	3days	1	ns	Rep ANOVA with PH test
max__right__back__iliac_hip_ankle	0days	7days	1	ns	Rep ANOVA with PH test
max__right__back__iliac_hip_ankle	0days	14days	1	ns	Rep ANOVA with PH test
max__right__back__iliac_hip_ankle	0days	21days	1	ns	Rep ANOVA with PH test
max__right__front__ellbow_wrist_toetip	0days	3days	0.68	ns	Rep ANOVA with PH test
max__right__front__ellbow_wrist_toetip	0days	7days	0.896	ns	Rep ANOVA with PH test
max__right__front__ellbow_wrist_toetip	0days	14days	0.929	ns	Rep ANOVA with PH test
max__right__front__ellbow_wrist_toetip	0days	21days	0.929	ns	Rep ANOVA with PH test
max__right__front__shoulder_ellbow_wrist	0days	3days	1	ns	Rep ANOVA with PH test
max__right__front__shoulder_ellbow_wrist	0days	7days	1	ns	Rep ANOVA with PH test
max__right__front__shoulder_ellbow_wrist	0days	14days	1	ns	Rep ANOVA with PH test
max__right__front__shoulder_ellbow_wrist	0days	21days	1	ns	Rep ANOVA with PH test
min__left__back__hip_ankle_toe	0days	3days	1	ns	Rep ANOVA with PH test
min__left__back__hip_ankle_toe	0days	7days	1	ns	Rep ANOVA with PH test
min__left__back__hip_ankle_toe	0days	14days	1	ns	Rep ANOVA with PH test
min__left__back__hip_ankle_toe	0days	21days	1	ns	Rep ANOVA with PH test
min__left__back__iliac_hip_ankle	0days	3days	1	ns	Rep ANOVA with PH test
min__left__back__iliac_hip_ankle	0days	7days	1	ns	Rep ANOVA with PH test
min__left__back__iliac_hip_ankle	0days	14days	1	ns	Rep ANOVA with PH test
min__left__back__iliac_hip_ankle	0days	21days	1	ns	Rep ANOVA with PH test
min__left__front__ellbow_wrist_toetip	0days	3days	0.769	ns	Rep ANOVA with PH test
min__left__front__ellbow_wrist_toetip	0days	7days	0.769	ns	Rep ANOVA with PH test
min__left__front__ellbow_wrist_toetip	0days	14days	0.176	ns	Rep ANOVA with PH test
min__left__front__ellbow_wrist_toetip	0days	21days	0.769	ns	Rep ANOVA with PH test
min__left__front__shoulder_ellbow_wrist	0days	3days	1	ns	Rep ANOVA with PH test
min__left__front__shoulder_ellbow_wrist	0days	7days	1	ns	Rep ANOVA with PH test
min__left__front__shoulder_ellbow_wrist	0days	14days	1	ns	Rep ANOVA with PH test
min__left__front__shoulder_ellbow_wrist	0days	21days	1	ns	Rep ANOVA with PH test
min__right__back__hip_ankle_toe	0days	3days	1	ns	Rep ANOVA with PH test
min__right__back__hip_ankle_toe	0days	7days	1	ns	Rep ANOVA with PH test
min__right__back__hip_ankle_toe	0days	14days	0.241	ns	Rep ANOVA with PH test
min__right__back__hip_ankle_toe	0days	21days	1	ns	Rep ANOVA with PH test
min__right__back__iliac_hip_ankle	0days	3days	0.912	ns	Rep ANOVA with PH test
min__right__back__iliac_hip_ankle	0days	7days	1	ns	Rep ANOVA with PH test
min__right__back__iliac_hip_ankle	0days	14days	1	ns	Rep ANOVA with PH test
min__right__back__iliac_hip_ankle	0days	21days	1	ns	Rep ANOVA with PH test
min__right__front__ellbow_wrist_toetip	0days	3days	1	ns	Rep ANOVA with PH test
min__right__front__ellbow_wrist_toetip	0days	7days	1	ns	Rep ANOVA with PH test
min__right__front__ellbow_wrist_toetip	0days	14days	1	ns	Rep ANOVA with PH test
min__right__front__ellbow_wrist_toetip	0days	21days	1	ns	Rep ANOVA with PH test
min__right__front__shoulder_ellbow_wrist	0days	3days	1	ns	Rep ANOVA with PH test
min__right__front__shoulder_ellbow_wrist	0days	7days	1	ns	Rep ANOVA with PH test

min__right__front__shoulder_ellbow_wrist	0days	14days	1	ns	Rep ANOVA with PH test
min__right__front__shoulder_ellbow_wrist	0days	21days	1	ns	Rep ANOVA with PH test

Suppl. Table 28: Parameter importance based on random forest classification baseline, 3, 7, 14, 21 dpi

Ranking	Importance (Gini)	Name Parameter	Groups
1	1.404140601	l-back-toe__Average_Height	baseline, 3,7,14,21 dpi
2	1.098874678	left__back__protraction	baseline, 3,7,14,21 dpi
3	1.007898844	l-front-toe-tip__Movement	baseline, 3,7,14,21 dpi
4	0.958852155	right__back__median	baseline, 3,7,14,21 dpi
5	0.94089747	right__back__movement_per_ste	baseline, 3,7,14,21 dpi
6	0.925255406	left__front__protraction	baseline, 3,7,14,21 dpi
7	0.922614498	left__back__average	baseline, 3,7,14,21 dpi
8	0.888160511	l-wrist__Average_Height	baseline, 3,7,14,21 dpi
9	0.860963463	l-back-ankle__Movement	baseline, 3,7,14,21 dpi
10	0.844080488	right__back__protraction	baseline, 3,7,14,21 dpi
11	0.73386252	l-wrist__Movement	baseline, 3,7,14,21 dpi
12	0.725433395	right__front__protraction	baseline, 3,7,14,21 dpi
13	0.718656082	FRBL_RATIO	baseline, 3,7,14,21 dpi
14	0.682319128	l-back-toe__Movement	baseline, 3,7,14,21 dpi
15	0.639498284	right__back__average	baseline, 3,7,14,21 dpi
16	0.622255378	left__front__retraction	baseline, 3,7,14,21 dpi
17	0.610304695	r-wrist__Movement	baseline, 3,7,14,21 dpi
18	0.604568189	left__back__retraction	baseline, 3,7,14,21 dpi
19	0.60391649	right__back__seconds	baseline, 3,7,14,21 dpi
20	0.573511732	r-wrist__Average_Height	baseline, 3,7,14,21 dpi

Suppl. Table 29: Parameter importance based on random forest classification baseline and 3 dpi

Ranking	Importance (Gini)	Name Parameter	Groups
1	0.343571429	l-hip__Average_Height	baseline, 3 dpi
2	0.236904762	r-front-toe-tip__Average_Height	baseline, 3 dpi
3	0.221428571	l-hip__Movement	baseline, 3 dpi
4	0.215714286	right__back__median	baseline, 3 dpi
5	0.210952381	right__back__seconds	baseline, 3 dpi
6	0.187142857	r-wrist__Average_Height	baseline, 3 dpi
7	0.157142857	r-hip__Average_Height	baseline, 3 dpi
8	0.15	r-hip__Movement	baseline, 3 dpi
9	0.145714286	right__back__average	baseline, 3 dpi
10	0.139285714	FRBL_RATIO	baseline, 3 dpi
11	0.105714286	l-front-toe-tip__Movement	baseline, 3 dpi
12	0.102857143	left__front__seconds	baseline, 3 dpi
13	0.10047619	left__back__average	baseline, 3 dpi
14	0.100238095	l-back-toe__Average_Height	baseline, 3 dpi
15	0.098571429	r-wrist__Movement	baseline, 3 dpi
16	0.098571429	right__front__average	baseline, 3 dpi
17	0.097142857	l-front-toe-tip__Average_Height	baseline, 3 dpi
18	0.096785714	left__back__seconds	baseline, 3 dpi
19	0.078095238	l-back-toe__Movement	baseline, 3 dpi
20	0.066666667	l-wrist__Movement	baseline, 3 dpi

Suppl. Table 30: Parameter importance based on random forest classification baseline and 21 dpi

Ranking	Importance (Gini)	Name Parameter	Groups
1	0.698500309	left__front__retraction	baseline, 21 dpi
2	0.328743558	left__front__median	baseline, 21 dpi
3	0.242116574	right__front__seconds	baseline, 21 dpi
4	0.217580396	l-tail-base__Average_Height	baseline, 21 dpi
5	0.171238404	l-tail-base__Movement	baseline, 21 dpi
6	0.166821274	right__front__retraction	baseline, 21 dpi
7	0.166770254	right__front__average	baseline, 21 dpi
8	0.147867965	r-back-ankle__Movement	baseline, 21 dpi
9	0.14038961	left__front__average	baseline, 21 dpi
10	0.138056586	left__front__protraction	baseline, 21 dpi
11	0.136403319	l-front-toe-tip__Movement	baseline, 21 dpi
12	0.122979283	right__back__seconds	baseline, 21 dpi
13	0.122492785	left__front__seconds	baseline, 21 dpi
14	0.118863636	r-iliac-crest__Average_Height	baseline, 21 dpi
15	0.1180906	left__front__retraction	baseline, 21 dpi
16	0.109763451	l-hip__Average_Height	baseline, 21 dpi
17	0.10025974	left__back__seconds	baseline, 21 dpi
18	0.098092146	left__back__median	baseline, 21 dpi
19	0.093073593	avg_stance_time	baseline, 21 dpi
20	0.088682746	avg_swing_time	baseline, 21 dpi

Suppl. Table 31: Error rate between intact and injured mice during ladder rung test

Measure	Day	median	mean	sd
l-back-toe	intact	0	5.07111936	7.338168353
l-back-toe	stroked	12.5	14.3187065	13.7133233
l-front-toe-tip	intact	0	8.54711306	11.48099774
l-front-toe-tip	stroked	14.2857143	15.5634395	13.1484941
r-back-toe	intact	0	1.66624501	4.431341378
r-back-toe	stroked	0	6.90764791	10.88975155
r-front-toe-tip	intact	0	5.77959198	7.420014048
r-front-toe-tip	stroked	0	6.99085418	9.900592938

Suppl. Table 32: Statistics for error rate between intact and injured mice during ladder rung test

Measure	group1	group2	p.adj	sig	Test*
l-back-toe	intact	stroked	3.31E-05	****	Dunn Test
l-front-toe-tip	intact	stroked	0.00092729	***	Dunn Test
r-back-toe	intact	stroked	0.00097815	***	Dunn Test
r-front-toe-tip	intact	stroked	0.77167211	ns	Dunn Test

Suppl. Table 33: Error rate in a time course during ladder rung test

Measure	Day	median	mean	sd
l-front-toe-tip	0days	0	9.07653292	11.59493899
l-front-toe-tip	3days	14.2857143	14.8750304	12.4414783
l-front-toe-tip	7days	16.6666667	19.0929903	14.82716005
l-front-toe-tip	14days	12.5	16.6249612	17.05103827
l-front-toe-tip	21days	12.5	14.4451062	15.43371401
l-back-toe	0days	0	5.22108844	7.162169313
l-back-toe	3days	12.5	14.6640165	13.73748383
l-back-toe	7days	10	12.2643206	15.0284742
l-back-toe	14days	9.09090909	10.4341769	11.42036526
l-back-toe	21days	0	6.88517964	10.68409771
r-back-toe	0days	0	1.75754611	4.534867628
r-back-toe	3days	0	6.95922814	10.89317723
r-back-toe	7days	0	3.69699123	8.373892334
r-back-toe	14days	0	4.01435574	7.569811652
r-back-toe	21days	0	4.31710495	8.36361438
r-front-toe-tip	0days	0	4.77024959	6.140608234
r-front-toe-tip	3days	0	7.29295811	10.08561303
r-front-toe-tip	7days	0	3.77267426	7.092408012
r-front-toe-tip	14days	0	8.01656476	10.54868099
r-front-toe-tip	21days	0	5.61199553	11.92702074

Suppl. Table 34:Statistics throughout a time course during ladder rung test

parameter	group1	group2	p.adj	p.adj.signif	Test*
l-back-toe	0days	3days	1.73E-05	****	Repeated ANOVA with post hoc test
l-back-toe	0days	7days	0.00067035	***	Repeated ANOVA with post hoc test
l-back-toe	0days	14days	0.01370947	*	Repeated ANOVA with post hoc test
l-back-toe	0days	21days	0.39747262	ns	Repeated ANOVA with post hoc test
l-front-toe-tip	0days	3days	0.01174657	*	Repeated ANOVA with post hoc test
l-front-toe-tip	0days	7days	8.95E-07	****	Repeated ANOVA with post hoc test
l-front-toe-tip	0days	14days	0.00025968	***	Repeated ANOVA with post hoc test
l-front-toe-tip	0days	21days	0.01174657	*	Repeated ANOVA with post hoc test
r-back-toe	0days	3days	0.04348641	*	Repeated ANOVA with post hoc test
r-back-toe	0days	7days	0.52810778	ns	Repeated ANOVA with post hoc test
r-back-toe	0days	14days	0.52810778	ns	Repeated ANOVA with post hoc test
r-back-toe	0days	21days	0.52810778	ns	Repeated ANOVA with post hoc test
r-front-toe-tip	0days	3days	0.65330132	ns	Repeated ANOVA with post hoc test
r-front-toe-tip	0days	7days	1	ns	Repeated ANOVA with post hoc test
r-front-toe-tip	0days	14days	0.34823814	ns	Repeated ANOVA with post hoc test
r-front-toe-tip	0days	21days	1	ns	Repeated ANOVA with post hoc test

Suppl. Table 35: Raw data during conventional tests

Behavior test	Score	Parameter	Mouse_ID	Day
ladder rung	0.013924	FL	1	baseline
ladder rung	0.034935	FL	2	baseline
ladder rung	0.054539	FL	3	baseline
ladder rung	0.03522	FL	4	baseline
ladder rung	0.04778985	FL	5	baseline
ladder rung	0.04300255	FL	6	baseline
ladder rung	0.04352911	FL	7	baseline
ladder rung	0.04876631	FL	8	baseline
ladder rung	0.04440803	FL	9	baseline
ladder rung	0.06496047	FL	10	baseline
ladder rung	0.06659583	FL	11	baseline
ladder rung	0.04204352	FL	12	baseline
ladder rung	0.184202	FL	1	3
ladder rung	0.249242	FL	2	3
ladder rung	0.21934	FL	3	3
ladder rung	0.2294	FL	4	3
ladder rung	0.2885575	FL	5	3
ladder rung	0.2779464	FL	6	3
ladder rung	0.2447014	FL	7	3
ladder rung	0.2359469	FL	8	3
ladder rung	0.2811619	FL	9	3
ladder rung	0.1750913	FL	10	3
ladder rung	0.2881345	FL	11	3
ladder rung	0.2974798	FL	12	3
ladder rung	0.1924652	FL	1	7
ladder rung	0.1758639	FL	2	7
ladder rung	0.234539	FL	3	7
ladder rung	0.2678063	FL	4	7
ladder rung	0.1683676	FL	5	7
ladder rung	0.1737422	FL	6	7
ladder rung	0.2573249	FL	7	7
ladder rung	0.2104021	FL	8	7
ladder rung	0.2819787	FL	9	7
ladder rung	0.2490475	FL	10	7
ladder rung	0.2944221	FL	11	7
ladder rung	0.1558081	FL	12	7
ladder rung	0.2152912	FL	1	14
ladder rung	0.2604266	FL	2	14
ladder rung	0.1762194	FL	3	14
ladder rung	0.2038705	FL	4	14
ladder rung	0.2518033	FL	5	14
ladder rung	0.1612487	FL	6	14
ladder rung	0.2015572	FL	7	14
ladder rung	0.2098318	FL	8	14
ladder rung	0.1715338	FL	9	14
ladder rung	0.1924448	FL	10	14
ladder rung	0.1960025	FL	11	14
ladder rung	0.1385358	FL	12	14
ladder rung	0.11456	FL	1	21
ladder rung	0.14304	FL	2	21

ladder rung	0.12314	FL	3	21
ladder rung	0.110394	FL	4	21
ladder rung	0.1703651	FL	5	21
ladder rung	0.2166202	FL	6	21
ladder rung	0.1061859	FL	7	21
ladder rung	0.1828388	FL	8	21
ladder rung	0.1609431	FL	9	21
ladder rung	0.2353897	FL	10	21
ladder rung	0.1891668	FL	11	21
ladder rung	0.2170997	FL	12	21
ladder rung	0.0437	ladder rung	1	baseline
ladder rung	0.066394	ladder rung	2	baseline
ladder rung	0.02452	ladder rung	3	baseline
ladder rung	0.0195602	ladder rung	4	baseline
ladder rung	0.03563	ladder rung	5	baseline
ladder rung	0.02546	ladder rung	6	baseline
ladder rung	0.013825	ladder rung	7	baseline
ladder rung	0.03994745	ladder rung	8	baseline
ladder rung	0.01769902	ladder rung	9	baseline
ladder rung	0.0375225	ladder rung	10	baseline
ladder rung	0.02544737	ladder rung	11	baseline
ladder rung	0.05332957	ladder rung	12	baseline
ladder rung	0.209953	ladder rung	1	3
ladder rung	0.1724	ladder rung	2	3
ladder rung	0.15355	ladder rung	3	3
ladder rung	0.13385	ladder rung	4	3
ladder rung	0.25836	ladder rung	5	3
ladder rung	0.1836	ladder rung	6	3
ladder rung	0.16754	ladder rung	7	3
ladder rung	0.1636239	ladder rung	8	3
ladder rung	0.2400068	ladder rung	9	3
ladder rung	0.1358733	ladder rung	10	3
ladder rung	0.157042	ladder rung	11	3
ladder rung	0.09770476	ladder rung	12	3
ladder rung	0.18425	ladder rung	1	7
ladder rung	0.14425	ladder rung	2	7
ladder rung	0.1856	ladder rung	3	7
ladder rung	0.17285	ladder rung	4	7
ladder rung	0.24258	ladder rung	5	7
ladder rung	0.143536	ladder rung	6	7
ladder rung	0.15342	ladder rung	7	7
ladder rung	0.2172824	ladder rung	8	7
ladder rung	0.1547405	ladder rung	9	7
ladder rung	0.199411	ladder rung	10	7
ladder rung	0.1812098	ladder rung	11	7
ladder rung	0.2150024	ladder rung	12	7
ladder rung	0.10353	ladder rung	1	14
ladder rung	0.11429	ladder rung	2	14
ladder rung	0.1434835	ladder rung	3	14
ladder rung	0.114935	ladder rung	4	14
ladder rung	0.1842	ladder rung	5	14
ladder rung	0.094852	ladder rung	6	14

ladder rung	0.14356	ladder rung	7	14
ladder rung	0.201433	ladder rung	8	14
ladder rung	0.1061027	ladder rung	9	14
ladder rung	0.1566955	ladder rung	10	14
ladder rung	0.1511585	ladder rung	11	14
ladder rung	0.1738028	ladder rung	12	14
ladder rung	0.0535	ladder rung	1	21
ladder rung	0.0245	ladder rung	2	21
ladder rung	0.13285	ladder rung	3	21
ladder rung	0.18646	ladder rung	4	21
ladder rung	0.09435	ladder rung	5	21
ladder rung	0.0843536	ladder rung	6	21
ladder rung	0.0483535	ladder rung	7	21
ladder rung	0.04401902	ladder rung	8	21
ladder rung	0.1562648	ladder rung	9	21
ladder rung	0.15005045	ladder rung	10	21
ladder rung	0.05684677	ladder rung	11	21
ladder rung	0.09030958	ladder rung	12	21
Neurolog Score	0	Neurolog Score	1	baseline
Neurolog Score	0	Neurolog Score	2	baseline
Neurolog Score	0.5	Neurolog Score	3	baseline
Neurolog Score	0	Neurolog Score	4	baseline
Neurolog Score	0	Neurolog Score	5	baseline
Neurolog Score	0	Neurolog Score	6	baseline
Neurolog Score	0	Neurolog Score	7	baseline
Neurolog Score	3.5	Neurolog Score	1	0
Neurolog Score	4	Neurolog Score	2	0
Neurolog Score	3.5	Neurolog Score	3	0
Neurolog Score	4.5	Neurolog Score	4	0
Neurolog Score	4	Neurolog Score	5	0
Neurolog Score	4	Neurolog Score	6	0
Neurolog Score	4	Neurolog Score	7	0
Neurolog Score	1.5	Neurolog Score	1	3
Neurolog Score	2	Neurolog Score	2	3
Neurolog Score	1.5	Neurolog Score	3	3
Neurolog Score	2	Neurolog Score	4	3
Neurolog Score	1.5	Neurolog Score	5	3
Neurolog Score	2	Neurolog Score	6	3
Neurolog Score	1.5	Neurolog Score	7	3
Neurolog Score	1.5	Neurolog Score	1	7
Neurolog Score	0	Neurolog Score	2	7
Neurolog Score	0.5	Neurolog Score	3	7
Neurolog Score	0	Neurolog Score	4	7
Neurolog Score	1	Neurolog Score	5	7
Neurolog Score	1	Neurolog Score	6	7
Neurolog Score	1	Neurolog Score	7	7
Neurolog Score	0	Neurolog Score	1	14
Neurolog Score	0	Neurolog Score	2	14
Neurolog Score	0.5	Neurolog Score	3	14
Neurolog Score	0.5	Neurolog Score	4	14
Neurolog Score	0.5	Neurolog Score	5	14
Neurolog Score	0.5	Neurolog Score	6	14

Neurolog Score	0.5	Neurolog Score	7	14
Neurolog Score	0	Neurolog Score	1	21
Neurolog Score	0	Neurolog Score	2	21
Neurolog Score	0	Neurolog Score	3	21
Neurolog Score	0.5	Neurolog Score	4	21
Neurolog Score	0.5	Neurolog Score	5	21
Neurolog Score	0	Neurolog Score	6	21
Neurolog Score	0.5	Neurolog Score	7	21
Cylinder	0.125	Dragging	1	baseline
Cylinder	0.090909	Dragging	2	baseline
Cylinder	0	Dragging	3	baseline
Cylinder	0	Dragging	4	baseline
Cylinder	0	Dragging	5	baseline
Cylinder	0.0625	Dragging	6	baseline
Cylinder	0	Dragging	7	baseline
Cylinder	0	Dragging	8	baseline
Cylinder	0	Dragging	9	baseline
Cylinder	0.03125	Dragging	10	baseline
Cylinder	0	Dragging	11	baseline
Cylinder	0	Dragging	12	baseline
Cylinder	0.333333	Dragging	1	3
Cylinder	0.342857	Dragging	2	3
Cylinder	0.215385	Dragging	3	3
Cylinder	0.310526	Dragging	4	3
Cylinder	0.45	Dragging	5	3
Cylinder	0.46	Dragging	6	3
Cylinder	0.357143	Dragging	7	3
Cylinder	0.333333	Dragging	8	3
Cylinder	0.2142857	Dragging	9	3
Cylinder	0.125	Dragging	10	3
Cylinder	0.5	Dragging	11	3
Cylinder	0.2173913	Dragging	12	3
Cylinder	0.28	Dragging	1	7
Cylinder	0.367	Dragging	2	7
Cylinder	0.254	Dragging	3	7
Cylinder	0.2842	Dragging	4	7
Cylinder	0.28	Dragging	5	7
Cylinder	0.33	Dragging	6	7
Cylinder	0.615385	Dragging	7	7
Cylinder	0.35	Dragging	8	7
Cylinder	0.353	Dragging	9	7
Cylinder	0.2453	Dragging	10	7
Cylinder	0.368	Dragging	11	7
Cylinder	0.3524	Dragging	12	7
Cylinder	0.2	Dragging	1	14
Cylinder	0.29	Dragging	2	14
Cylinder	0.22	Dragging	3	14
Cylinder	0.2	Dragging	4	14
Cylinder	0.2	Dragging	5	14
Cylinder	0.57	Dragging	6	14
Cylinder	0.4	Dragging	7	14
Cylinder	0.36	Dragging	8	14

Cylinder	0.15	Dragging	9	14
Cylinder	0.356	Dragging	10	14
Cylinder	0.294	Dragging	11	14
Cylinder	0.2256	Dragging	12	14
Cylinder	0.15	Dragging	1	21
Cylinder	0.166	Dragging	2	21
Cylinder	0.25	Dragging	3	21
Cylinder	0.25	Dragging	4	21
Cylinder	0.4	Dragging	5	21
Cylinder	0.3	Dragging	6	21
Cylinder	0.291667	Dragging	7	21
Cylinder	0.381818	Dragging	8	21
Cylinder	0.3333333	Dragging	9	21
Cylinder	0.25	Dragging	10	21
Cylinder	0.5	Dragging	11	21
Cylinder	0.4	Dragging	12	21
Cylinder	0.5	Asymmetry	1	baseline
Cylinder	0.3	Asymmetry	2	baseline
Cylinder	0.4	Asymmetry	3	baseline
Cylinder	0.7	Asymmetry	4	baseline
Cylinder	0.25	Asymmetry	5	baseline
Cylinder	0	Asymmetry	6	baseline
Cylinder	0.146	Asymmetry	7	baseline
Cylinder	0.14	Asymmetry	8	baseline
Cylinder	0.27	Asymmetry	9	baseline
Cylinder	0.43	Asymmetry	10	baseline
Cylinder	0	Asymmetry	11	baseline
Cylinder	0.090909	Asymmetry	12	baseline
Cylinder	0.5	Asymmetry	1	3
Cylinder	0.85	Asymmetry	2	3
Cylinder	0.9	Asymmetry	3	3
Cylinder	0.6	Asymmetry	4	3
Cylinder	0.4744	Asymmetry	5	3
Cylinder	0.64674	Asymmetry	6	3
Cylinder	0.67	Asymmetry	7	3
Cylinder	0.79	Asymmetry	8	3
Cylinder	0.77	Asymmetry	9	3
Cylinder	0.85	Asymmetry	10	3
Cylinder	0.667	Asymmetry	11	3
Cylinder	0.37	Asymmetry	12	3
Cylinder	0.736	Asymmetry	1	7
Cylinder	0.665	Asymmetry	2	7
Cylinder	0.6755	Asymmetry	3	7
Cylinder	0.7055	Asymmetry	4	7
Cylinder	0.68	Asymmetry	5	7
Cylinder	0.57	Asymmetry	6	7
Cylinder	0.68	Asymmetry	7	7
Cylinder	0.7	Asymmetry	8	7
Cylinder	0.574	Asymmetry	9	7
Cylinder	0.79	Asymmetry	10	7
Cylinder	0.4	Asymmetry	11	7
Cylinder	0.4577	Asymmetry	12	7

Cylinder	0.75	Asymmetry	1	14
Cylinder	0.67	Asymmetry	2	14
Cylinder	0.766	Asymmetry	3	14
Cylinder	0.799	Asymmetry	4	14
Cylinder	0.55	Asymmetry	5	14
Cylinder	0.56	Asymmetry	6	14
Cylinder	0.77	Asymmetry	7	14
Cylinder	0.55	Asymmetry	8	14
Cylinder	0.842	Asymmetry	9	14
Cylinder	0.95	Asymmetry	10	14
Cylinder	0.5	Asymmetry	11	14
Cylinder	0.855	Asymmetry	12	14
Cylinder	0.9	Asymmetry	1	21
Cylinder	0.75	Asymmetry	2	21
Cylinder	0.9	Asymmetry	3	21
Cylinder	0.82307692	Asymmetry	4	21
Cylinder	0.58	Asymmetry	5	21
Cylinder	0.5	Asymmetry	6	21
Cylinder	0.56	Asymmetry	7	21
Cylinder	0.7	Asymmetry	8	21
Cylinder	0.8	Asymmetry	9	21
Cylinder	0.9	Asymmetry	10	21
Cylinder	0.35	Asymmetry	11	21
Cylinder	0.76	Asymmetry	12	21
Rotarod	0.00689655	Rotarod_Lat	1	baseline
Rotarod	0.00655022	Rotarod_Lat	2	baseline
Rotarod	0.00562852	Rotarod_Lat	3	baseline
Rotarod	0.00993377	Rotarod_Lat	4	baseline
Rotarod	0.01132075	Rotarod_Lat	5	baseline
Rotarod	0.00689655	Rotarod_Lat	6	baseline
Rotarod	0.00806452	Rotarod_Lat	7	baseline
Rotarod	0.015625	Rotarod_Lat	8	baseline
Rotarod	0.01345291	Rotarod_Lat	9	baseline
Rotarod	0.00849858	Rotarod_Lat	10	baseline
Rotarod	0.01003344	Rotarod_Lat	11	baseline
Rotarod	0.01079137	Rotarod_Lat	12	baseline
Rotarod	0.01041667	Rotarod_Lat	1	3
Rotarod	0.00898204	Rotarod_Lat	2	3
Rotarod	0.04761905	Rotarod_Lat	3	3
Rotarod	0.01315789	Rotarod_Lat	4	3
Rotarod	0.01376147	Rotarod_Lat	5	3
Rotarod	0.01204819	Rotarod_Lat	6	3
Rotarod	0.00828729	Rotarod_Lat	7	3
Rotarod	0.02362205	Rotarod_Lat	8	3
Rotarod	0.01538462	Rotarod_Lat	9	3
Rotarod	0.00898204	Rotarod_Lat	10	3
Rotarod	0.02912621	Rotarod_Lat	11	3
Rotarod	0.02857143	Rotarod_Lat	12	3
Rotarod	0.01158301	Rotarod_Lat	1	7
Rotarod	0.01195219	Rotarod_Lat	2	7
Rotarod	0.00704225	Rotarod_Lat	3	7
Rotarod	0.01530612	Rotarod_Lat	4	7

Rotarod	0.01219512	Rotarod_Lat	5	7
Rotarod	0.01010101	Rotarod_Lat	6	7
Rotarod	0.0078329	Rotarod_Lat	7	7
Rotarod	0.01158301	Rotarod_Lat	8	7
Rotarod	0.00707547	Rotarod_Lat	9	7
Rotarod	0.00977199	Rotarod_Lat	10	7
Rotarod	0.00900901	Rotarod_Lat	11	7
Rotarod	0.01578947	Rotarod_Lat	12	7
Rotarod	0.01075269	Rotarod_Lat	1	14
Rotarod	0.00923077	Rotarod_Lat	2	14
Rotarod	0.00573614	Rotarod_Lat	3	14
Rotarod	0.01145038	Rotarod_Lat	4	14
Rotarod	0.00815217	Rotarod_Lat	5	14
Rotarod	0.00683371	Rotarod_Lat	6	14
Rotarod	0.00674157	Rotarod_Lat	7	14
Rotarod	0.01578947	Rotarod_Lat	8	14
Rotarod	0.02173913	Rotarod_Lat	9	14
Rotarod	0.0096463	Rotarod_Lat	10	14
Rotarod	0.00869565	Rotarod_Lat	11	14
Rotarod	0.01578947	Rotarod_Lat	12	14
Rotarod	0.01016949	Rotarod_Lat	1	21
Rotarod	0.00466563	Rotarod_Lat	2	21
Rotarod	0.00717703	Rotarod_Lat	3	21
Rotarod	0.00806452	Rotarod_Lat	4	21
Rotarod	0.00717703	Rotarod_Lat	5	21
Rotarod	0.00895522	Rotarod_Lat	6	21
Rotarod	0.00791557	Rotarod_Lat	7	21
Rotarod	0.00777202	Rotarod_Lat	8	21
Rotarod	0.01045296	Rotarod_Lat	9	21
Rotarod	0.00714286	Rotarod_Lat	10	21
Rotarod	0.01045296	Rotarod_Lat	11	21
Rotarod	0.01428571	Rotarod_Lat	12	21
Single Pellet Grasping	0	DragDrop	1	baseline
Single Pellet Grasping	10	DragDrop	1	4
Single Pellet Grasping	8	DragDrop	1	7
Single Pellet Grasping	14	DragDrop	1	14
Single Pellet Grasping	8	DragDrop	1	21
Single Pellet Grasping	11	DragDrop	1	28
Single Pellet Grasping	0	DragDrop	2	baseline
Single Pellet Grasping	21	DragDrop	2	4
Single Pellet Grasping	17	DragDrop	2	7
Single Pellet Grasping	18	DragDrop	2	14
Single Pellet Grasping	16	DragDrop	2	21
Single Pellet Grasping	18	DragDrop	2	28
Single Pellet Grasping	0	DragDrop	3	baseline
Single Pellet Grasping	20	DragDrop	3	4
Single Pellet Grasping	20	DragDrop	3	7
Single Pellet Grasping	14	DragDrop	3	14
Single Pellet Grasping	16	DragDrop	3	21
Single Pellet Grasping	18	DragDrop	3	28
Single Pellet Grasping	0	DragDrop	4	baseline
Single Pellet Grasping	20	DragDrop	4	4

Single Pellet Grasping	17	DragDrop	4	7
Single Pellet Grasping	13	DragDrop	4	14
Single Pellet Grasping	NA	DragDrop	4	21
Single Pellet Grasping	NA	DragDrop	4	28
Single Pellet Grasping	0	DragDrop	5	baseline
Single Pellet Grasping	18	DragDrop	5	4
Single Pellet Grasping	15	DragDrop	5	7
Single Pellet Grasping	19	DragDrop	5	14
Single Pellet Grasping	17	DragDrop	5	21
Single Pellet Grasping	12	DragDrop	5	28
Single Pellet Grasping	0	DragDrop	6	baseline
Single Pellet Grasping	16	DragDrop	6	4
Single Pellet Grasping	19	DragDrop	6	7
Single Pellet Grasping	16	DragDrop	6	14
Single Pellet Grasping	17	DragDrop	6	21
Single Pellet Grasping	16	DragDrop	6	28
Single Pellet Grasping	23.33333	Fail	1	baseline
Single Pellet Grasping	40	Fail	1	4
Single Pellet Grasping	16.66667	Fail	1	7
Single Pellet Grasping	26.66667	Fail	1	14
Single Pellet Grasping	20	Fail	1	21
Single Pellet Grasping	18.33333	Fail	1	28
Single Pellet Grasping	30	Fail	2	baseline
Single Pellet Grasping	48.33333	Fail	2	4
Single Pellet Grasping	35	Fail	2	7
Single Pellet Grasping	33.33333	Fail	2	14
Single Pellet Grasping	43.33333	Fail	2	21
Single Pellet Grasping	33.33333	Fail	2	28
Single Pellet Grasping	50	Fail	3	baseline
Single Pellet Grasping	43.47826	Fail	3	4
Single Pellet Grasping	43.33333	Fail	3	7
Single Pellet Grasping	23.33333	Fail	3	14
Single Pellet Grasping	30	Fail	3	21
Single Pellet Grasping	30	Fail	3	28
Single Pellet Grasping	20	Fail	4	baseline
Single Pellet Grasping	33.33333	Fail	4	4
Single Pellet Grasping	30.35714	Fail	4	7
Single Pellet Grasping	25	Fail	4	14
Single Pellet Grasping	NA	Fail	4	21
Single Pellet Grasping	NA	Fail	4	28
Single Pellet Grasping	20	Fail	5	baseline
Single Pellet Grasping	52	Fail	5	4
Single Pellet Grasping	35	Fail	5	7
Single Pellet Grasping	38.33333	Fail	5	14
Single Pellet Grasping	41.66667	Fail	5	21
Single Pellet Grasping	22.22222	Fail	5	28
Single Pellet Grasping	36.66667	Fail	6	baseline
Single Pellet Grasping	40	Fail	6	4
Single Pellet Grasping	45	Fail	6	7
Single Pellet Grasping	30	Fail	6	14
Single Pellet Grasping	35	Fail	6	21
Single Pellet Grasping	33.33333	Fail	6	28

Suppl. Table 36: Summary of conventional tests

Behavior test	Parameter	Day	Value	Median	sd
Cylinder	Dragging	baseline	0.02580492	0	0.043465278
Cylinder	Dragging	3	0.3216045	0.333333	0.113574086
Cylinder	Dragging	7	0.33994042	0.34	
Cylinder	Dragging	14	0.2888	0.2578	0.11782193
Cylinder	Dragging	21	0.30606819	0.2958335	0.102420194
Cylinder	Asymmetry	baseline	0.26890908	0.26	0.211431335
Cylinder	Asymmetry	3	0.67401167	0.6685	0.166382983
Cylinder	Asymmetry	7	0.63614167	0.67775	0.114654481
Cylinder	Asymmetry	14	0.7135	0.758	0.145089001
Cylinder	Asymmetry	21	0.71025641	0.755	0.177478706
ladder rung	FL	baseline	0.04497614	0.04396857	0.013998926
ladder rung	FL	3	0.24760031	0.2469717	0.040909479
ladder rung	FL	7	0.22181397	0.22247055	0.048428787
ladder rung	FL	14	0.19823047	0.19877985	0.034868174
ladder rung	FL	21	0.16414528	0.1656541	0.045218736
ladder rung	ladder rung	baseline	0.03358626	0.030545	0.015616676
ladder rung	ladder rung	3	0.17279198	0.16558195	0.045312186
ladder rung	ladder rung	7	0.18284434	0.1827299	0.031428618
ladder rung	ladder rung	14	0.14067025	0.14352175	0.034509077
ladder rung	ladder rung	21	0.09348814	0.08733159	0.051885317
Neurolog Score	Neurolog Scc	baseline	0.07142857	0	0.188982237
Neurolog Score	Neurolog Scc	0	3.92857143	4	0.34503278
Neurolog Score	Neurolog Scc	3	1.71428571	1.5	0.267261242
Neurolog Score	Neurolog Scc	7	0.71428571	1	0.56694671
Neurolog Score	Neurolog Scc	14	0.35714286	0.5	0.243975018
Neurolog Score	Neurolog Scc	21	0.21428571	0	0.267261242
Rotarod	Rotarod_Lat	baseline	0.00947435	0.00921618	0.002998449
Rotarod	Rotarod_Lat	3	0.01832991	0.01345968	0.011837166
Rotarod	Rotarod_Lat	7	0.01077013	0.01084201	0.002880008
Rotarod	Rotarod_Lat	14	0.01087979	0.00943854	0.004698944
Rotarod	Rotarod_Lat	21	0.00868592	0.00799004	0.002426897
Single Pellet Grasping	Fail	baseline	30	26.666665	11.73787867
Single Pellet Grasping	Fail	4	42.8574867	41.73913	6.648244732
Single Pellet Grasping	Fail	7	34.22619	35	10.23248587
Single Pellet Grasping	Fail	14	29.4444433	28.333335	5.643743373
Single Pellet Grasping	Fail	21	34	35	9.472181346
Single Pellet Grasping	Fail	28	27.444442	30	6.822258514
Single Pellet Grasping	DragDrop	baseline	0	0	0
Single Pellet Grasping	DragDrop	4	17.5	19	4.086563348
Single Pellet Grasping	DragDrop	7	16	17	4.289522118
Single Pellet Grasping	DragDrop	14	15.6666667	15	2.422120283
Single Pellet Grasping	DragDrop	21	14.8	16	3.834057903
Single Pellet Grasping	DragDrop	28	15	16	3.31662479

Suppl. Table 37: Statistical test of conventional tests

Behavior test	Parameter	group1	group2	p.adj.signif	Stat test
Neurolog Score	Neurolog Scc	baseline	0	****	Repeat. ANOVA with PH
Neurolog Score	Neurolog Scc	baseline	3	****	Repeat. ANOVA with PH
Neurolog Score	Neurolog Scc	baseline	7	*	Repeat. ANOVA with PH
Neurolog Score	Neurolog Scc	baseline	14	ns	Repeat. ANOVA with PH
Neurolog Score	Neurolog Scc	baseline	21	ns	Repeat. ANOVA with PH
Rotarod	Rotarod_Lat	baseline	3	**	Repeat. ANOVA with PH
Rotarod	Rotarod_Lat	baseline	7	ns	Repeat. ANOVA with PH
Rotarod	Rotarod_Lat	baseline	14	ns	Repeat. ANOVA with PH
Rotarod	Rotarod_Lat	baseline	21	ns	Repeat. ANOVA with PH
Cylinder	Dragging	baseline	3	****	Repeat. ANOVA with PH
Cylinder	Dragging	baseline	7	****	Repeat. ANOVA with PH
Cylinder	Dragging	baseline	14	****	Repeat. ANOVA with PH
Cylinder	Dragging	baseline	21	****	Repeat. ANOVA with PH
Cylinder	Asymmetry	baseline	3	****	Repeat. ANOVA with PH
Cylinder	Asymmetry	baseline	7	****	Repeat. ANOVA with PH
Cylinder	Asymmetry	baseline	14	****	Repeat. ANOVA with PH
Cylinder	Asymmetry	baseline	21	****	Repeat. ANOVA with PH
ladder rung	FL	baseline	3	****	Repeat. ANOVA with PH
ladder rung	FL	baseline	7	****	Repeat. ANOVA with PH
ladder rung	FL	baseline	14	****	Repeat. ANOVA with PH
ladder rung	FL	baseline	21	****	Repeat. ANOVA with PH
ladder rung	ladder rung	baseline	3	****	Repeat. ANOVA with PH
ladder rung	ladder rung	baseline	7	****	Repeat. ANOVA with PH
ladder rung	ladder rung	baseline	14	****	Repeat. ANOVA with PH
ladder rung	ladder rung	baseline	21	**	Repeat. ANOVA with PH
Single Pellet Grasping	Fail	baseline	4	ns	Repeat. ANOVA with PH
Single Pellet Grasping	Fail	baseline	7	ns	Repeat. ANOVA with PH
Single Pellet Grasping	Fail	baseline	14	ns	Repeat. ANOVA with PH
Single Pellet Grasping	Fail	baseline	21	ns	Repeat. ANOVA with PH
Single Pellet Grasping	Fail	baseline	28	ns	Repeat. ANOVA with PH
Single Pellet Grasping	DragDrop	baseline	4	****	Repeat. ANOVA with PH
Single Pellet Grasping	DragDrop	baseline	7	****	Repeat. ANOVA with PH
Single Pellet Grasping	DragDrop	baseline	14	****	Repeat. ANOVA with PH
Single Pellet Grasping	DragDrop	baseline	21	****	Repeat. ANOVA with PH
Single Pellet Grasping	DragDrop	baseline	28	****	Repeat. ANOVA with PH
